# Integrated proteomics and metabolomics analysis of transgenic and gene-stacked maize line seeds

**DOI:** 10.1080/21645698.2021.1934351

**Published:** 2021-06-07

**Authors:** Weixiao Liu, Haiming Zhao, Chaohua Miao, Wujun Jin

**Affiliations:** a Biotechnology Research Institute, Chinese Agricultural and Academic Sciences, Beijing, P.R. China; b State Key Laboratory of Agrobiotechnology and National Maize Improvement Center, Department of Plant Genetics and Breeding, China Agricultural University, Beijing, P. R. China

**Keywords:** Maize seeds, unintended effects, iTRAQ-based quantitative proteomics, widely targeted metabolomics, genetically modified, gene stacking

## Abstract

Unintended effects of genetically modified (GM) crops may pose safety issues. Omics techniques provide researchers with useful tools to assess such unintended effects. Proteomics and metabolomics analyses were performed for three GM maize varieties, 2A-7, CC-2, and 2A-7×CC-2 stacked transgenic maize, and the corresponding non-GM parent Zheng58.

Proteomics revealed 120, 271 and 135 maize differentially expressed proteins (DEPs) in the 2A-7/Zheng58, CC-2/Zheng58 and 2A-7×CC-2/Zheng58 comparisons, respectively. Kyoto Encyclopedia of Genes and Genomes (KEGG) pathway enrichment analysis showed that most DEPs participated in metabolic pathways and the biosynthesis of secondary metabolite. Metabolomics revealed 179, 135 and 131 differentially accumulated metabolites (DAMs) in the 2A-7/Zheng58, CC-2/Zheng58 and 2A-7×CC-2/Zheng58 comparisons, respectively. Based on KEGG enrichment analysis, most DAMs are involved in the biosynthesis of secondary metabolite and metabolic pathways. According to integrated proteomics and metabolomics analysis, the introduction of exogenous EPSPS did not affect the expression levels of six other enzymes or the abundance of seven metabolites involved in the shikimic acid pathway in CC-2 and 2A-7×CC-2 seeds. Six co-DEPs annotated by integrated proteomics and metabolomics pathway analysis were further analyzed by qRT-PCR.

This study successfully employed integrated proteomic and metabolomic technology to assess unintended changes in maize varieties. The results suggest that GM and gene stacking do not cause significantly unintended effects.

## 1. Introduction

The commercialization of genetically modified (GM) crops began in 1996.^1^ From 1996 to 2018, land planted with GM crops increased from 1.7 to 191.7 million hectares.^2^ Stacked GM crops, which combine two or more traits, have multiple benefits and satisfy the need for planting diversity. The planting area of stacked GM crops accounts for approximately 40% of the global GM crop production area.^2^ Although the commercialization of GM crops has many advantages, such as considerable economic benefits and reduced chemical pesticide pollution, more attention is being given to their food and environmental safety.^3,4^ Genetic modification might lead to not only the insertion of exogenous DNA fragments but also the rearrangement or deletion of some endogenous genes,^5^ therefore interfering with certain biochemical pathways and possibly producing new allergens or toxins. Thus, safety assessments of GM crops must be comprehensively carried out.^6–9^

Different from previous directional detection methods, such as PCR and ELISA, the newly developed nondirectional detection methods, such as omics (e.g., genomics, transcriptomics, proteomics, metabolomics), developed in recent years enable carrying out comprehensive safety assessment and provide detailed insight into any unintended changes in the GM crops studied.^10,11^ Although genomics and transcriptomics provide high coverage of gene sequence and expression data, which are distant from affecting the levels of nutrients, antinutrients and other factors that contribute to food and feed quality and safety, proteomics and metabolomics are closer to such endpoint phenotypes.^12^ The use of isobaric tags with relative and absolute quantitation (iTRAQ)-based proteomics is a high-throughput method with high accuracy and repeatability that has been widely used in the safety evaluation of GM crops such as rice, potatoes and soybeans.^13–18^ Our previous study also showed that this method can successfully assess unintended changes in GM maize.^19^ In addition to proteins, primary metabolism profoundly influences crop growth, development and reproduction.^20,21^ Metabolites such as carbohydrates, amino acids and organic acids accumulate in seeds, largely affecting crop quality traits.^22^ Recent advances in metabolite profiling technology make it possible to comprehensively compare the differences in metabolites of crops affected by the growth environment,^12^ genetic engineering and conventional cross-breeding.^23–25^

In this study, proteomics and metabolomics were used to identify the unintended changes in seeds of the GM maize lines 2A-7 and CC-2 and the stacked line 2A-7×CC-2. Based on the proteomics and metabolomics data, the changes in protein expression and metabolites were evaluated between three GM maize lines, 2A-7, CC-2, and 2A-7×CC-2, and their non-GM parent, Zheng58.

## 2. Materials and Methods

### 2.1 Plant Materials

Seeds of the GM maize lines 2A-7 (carrying *cry1Ab* and *cry2Ab* genes), CC-2 (carrying *maroACC* gene) and the stacked line 2A-7×CC-2 (containing *cry1Ab, cry2Ab* and *maroACC* genes), as well as their non-GM parent, Zheng58, were studied. The GM maize line and non-GM parent seeds were provided by the State Key Laboratory of Agrobiotechnology and National Maize Improvement Center, Department of Plant Genetics and Breeding, China Agricultural University (CAU). All maize seeds collected were obtained from the same natural growth environment and stored at −80°C. Full and uniform maize seeds were selected as experimental materials.

### 2.2 DNA Extraction

Maize seed genomic DNA was extracted using the Quickly Genome DNA Extraction Kit (Tiangen, Beijing, China).

### 2.3 PCR-based Detection of Transgenic Maize

Event-specific PCR was used to detect specific events in GM maize lines. The sequences of the event-specific primers used and the sizes of the amplified DNA fragments are listed in Supplementary Table S1. PCR consisted of denaturing at 95°C for 1 min and 30 cycles of denaturing at 95°C for 15 s followed by annealing at 58°C and extension at 68°C for 30 s.

### 2.4 Protein Preparation

Three biological replicates of seeds of the different maize lines were used for protein profiling in this study. Maize seed grains of each line were ground in liquid N_2_. Total proteins were extracted with lysis buffer. The protein concentration was determined by the Bradford method.^26^

### 2.5 Trypsin Digestion and iTRAQ Labeling

The extracted proteins (100 µg) were digested with 4 µg of trypsin overnight at 37°C. Protein reduction, blocking of cysteine residues, and digestion were performed according to the iTRAQ manufacturer’s protocol (SCIEX, Framingham, MA, USA). The digested peptides were labeled with individual iTRAQ reagents, following the standard iTRAQ protocol for the 8-plex kit. The tags used were 113 Da for 2A-7, 114 Da for CC-2, 115 Da for 2A-7× CC-2, and 116 Da for Zheng58. The labeled samples were mixed in equal amounts and lyophilized.

### 2.6 LC and MS/MS (Liquid Chromatography and Tandem Mass Spectrometry) Analysis

The peptide mixture was redissolved in solution A (98% ddH_2_O, 2% acetonitrile and 0.1% formic acid) and then fractionated by a TripleTOF 5600 Plus instrument (SCIEX, Framingham, MA, USA). Then, 100 μg of the mixture was desalted and fractionated using a Durashell-C18 reversed-phase column. Next, solution B (98% acetonitrile, 2% ddH_2_O and 0.1% formic acid) was added. After separation, the fractions were resuspended in 20 μL of solution C (0.1% formic acid and 2% methanol in water), separated by an Eksigent nanoLC instrument (SCIEX, Framingham MA, USA) and analyzed by on-line electrospray tandem mass spectrometry.

### 2.7 Analysis of Proteomic Data

The original files generated by the Q-Exactive system were analyzed using ProteinPilotTM V4.5 (Thermo Fisher Scientific, Waltham, MA, USA), and protein identification was performed using the Mascot search engine (Matrix Science, London, UK; version 2.3.02) against the UniProt *Zea mays* (maize) database supplemented with 3 foreign proteins, namely, Cry2Ab, Cry1Ab and EPSPS (5-enolpyruvulshikimate-3-phosphate synthase enzyme, *maroACC* gene coding product). All the identified proteins were matched with at least one unique peptide at ≥ 95% confidence.^14,27^ Proteins that had a fold change ≥ 2 or ≤ 0.5 and *P* value ≤ 0.05^15,28,29^ among the comparison groups were considered DEPs. The principal components (PCs) analysis was performed by the statistics function prcomp within R (www.r-project.org). A heatmap of the identified proteins was generated by hierarchical clustering. Functional classification of the DEPs was performed by Gene Ontology (GO) annotation and enrichment using the Gene Ontology database (http://www.geneontology.org/). KEGG pathway annotation and enrichment of the DEPs were carried out using the KEGG database (http://www.genome.jp/kegg/).

### 2.8 Metabolite Preparation

Six biological replicates of seeds of the different maize lines were used for metabolite profiling in this study. Maize seed grains of each line were ground in liquid N_2_. The total metabolites were extracted with 70% aqueous methanol from 100 mg of seed powder overnight at 4°C. Following centrifugation at 10,000 g for 10 min, the extracts were absorbed by a CNWBOND Carbon-GCB SPE Cartridge (ANPEL, Shanghai, China) and filtered before UPLC (ultra performance liquid chromatography)-MS/MS analysis.

### 2.9 UPLC Conditions

The extracted metabolites were analyzed using a UPLC-ESI-MS/MS system (UPLC, Shim-pack UFLC SHIMADZU CBM30A system; MS, Applied Biosystems 4500 Q TRAP). The analytical conditions were as follows: UPLC column, Agilent SB-C18 (1.8 µm, 2.1 mm×100 mm). The mobile phase consisted of solvent A (98% ddH_2_O, 0.1% formic acid) and solvent B (acetonitrile). Sample measurements were performed with a gradient program that employed the starting conditions of 95% A and 5% B. Within 9 min, a linear gradient to 5% A and 95% B was programmed, and a composition of 5% A and 95% B was maintained for 1 min. Subsequently, a composition of 95% A and 5.0% B was adjusted within 1.10 min and kept for 2.9 min. The column oven was set to 40°C, and the injection volume was 4 μL. The effluent was alternatively connected to an electron spray ionization-triple quadrupole-linear ion trap (ESI-QTRAP)-MS.

### 2.10 ESI-QTRAP-MS/MS

The ESI source operation parameters were as follows: ion source, turbo spray; source temperature 550°C; ion spray voltage (IS), 5500 V (positive ion mode)/-4500 V (negative ion mode); the ion source gas I (GSI), gas II (GSII), and curtain gas (CUR) were set at 50, 60, and 30.0 psi, respectively; and the collision gas (CAD) was high. Instrument tuning and mass calibration were performed with 10 and 100 μmol/L polypropylene glycol solutions in QQQ (triple quadrupole) and LIT (linear ion trap) modes, respectively. QQQ scans were acquired as MRM (multiple reaction monitoring) experiments with the collision gas (nitrogen) set to 5 psi. The DP (declustering potential) and CE (collision energy) of individual MRM transitions were determined with further DP and CE optimization. A specific set of MRM transitions was monitored for each period according to the metabolites eluted within this period.

### 2.11 Metabolite Data Analysis

Before data analysis, quality control (QC) analysis was conducted to confirm the reliability of the data. A QC sample was prepared by the mixture of sample extracts and inserted into every two samples to monitor the changes over repeated analyses. Orthogonal partial least squares-discriminant analysis (OPLS-DA) was used to maximize the metabolome differences by removing the irrelevant differences between the GM and non-GM parent samples. On the basis of the OPLS-DA results, the metabolites of different maize samples were preliminarily screened from the variable importance in projection (VIP) values of the obtained multivariate OPLS-DA model. The *P*-value and fold change values of the univariate analysis were combined to further screen differential metabolites. Metabolites with VIP ≥ 1 and fold change ≥ 2 or fold change ≤ 0.5 were considered differential metabolites for group discrimination.^30^ A heatmap based on the hierarchical cluster analysis method was generated in R software (www.r-project.org). KEGG pathway annotation of the DAMs was carried out using the KEGG database (http://www.genome.jp/kegg/).^31,32^

### 2.12 *qRT-PCR (Quantitative Real-time* PCR)

A total of approximately 1.0 g of seeds ground in liquid N_2_ per maize line was used for total RNA extraction with Ambion PureLink plant RNA reagent (Invitrogen, CA, USA). RNA integrity was analyzed by agarose gel electrophoresis and reverse transcribed with M-MLV reverse transcriptase (Invitrogen, CA, USA). The sequences of gene-specific primers used for quantitative real-time PCR (qPCR) are listed in Supplementary Table S2. qRT-PCR was performed using the SYBR Green qRT-PCR Kit (Bio-Rad, CA, USA) with three biological replicates. All reactions were conducted on a CFX96 real-time PCR system (Bio-Rad). The PCR conditions consisted of denaturing at 95°C for 1 min and 40 cycles of denaturing at 95°C for 15 s, followed by annealing and extension at 60°C for 30 s. The qRT-PCR data were analyzed using the 2^−ΔΔCT^ relative quantification method.^33^ Expression of *GAPDH (glyceraldehyde-3-phosphate dehydrogenase gene)* was measured as an internal control.

### 2.13 ELISA (Enzyme-Linked Immunosorbent Assay)

Maize seeds were ground with liquid N_2_. Proteins were extracted with lysis buffer. The contents of Cry1Ab, Cry2Ab and EPSPS were assessed using ELISA kits (Agdia, IN, USA).

## 3. Results

### 3.1 Confirmation of GM Maize Lines

Seeds were used to study the proteomic and metabolomic differences between 3 GM maize lines (2A-7, CC-2 and 2A-7×CC-2) and their non-GM parent, Zheng58. Event-specific PCR was carried out for the identification of transformants. The target DNA fragment was only obtained from GM maize lines (Supplementary Figure S1) and then sequenced for further verification.

### 3.2 Protein Profiling of Maize Seeds

**Figure 1.**
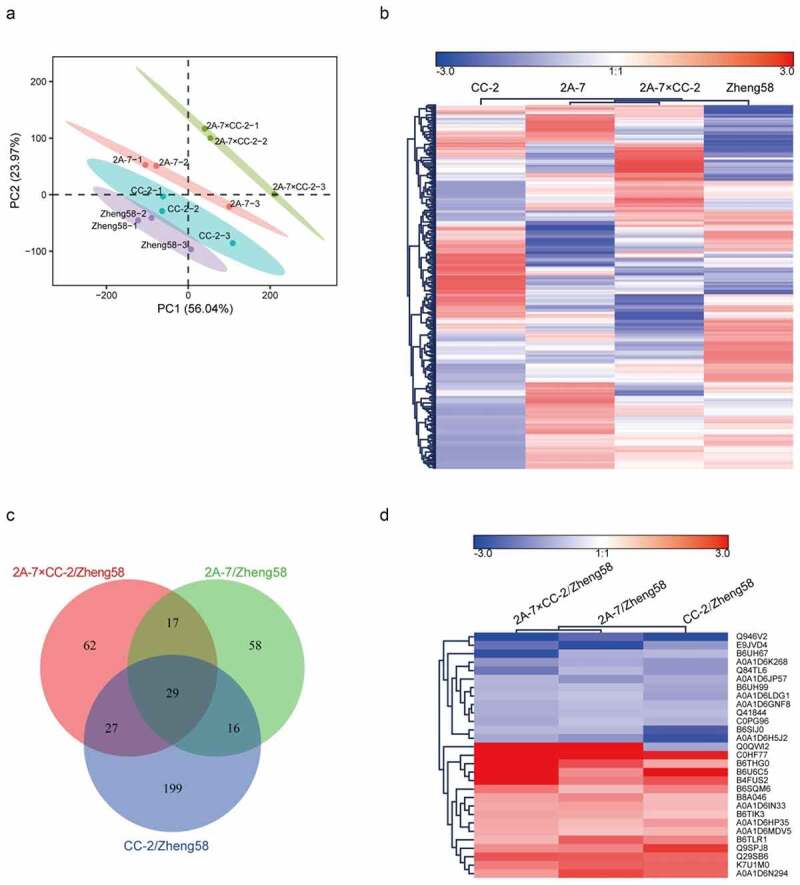
**Protein expression pattern analysis**. (a) Principal components (PCs) analyses of protein levels in seeds of 4 maize lines. Score plot of the first two PCs with the explained variance. (b) Cluster map comparing the protein expression patterns of the 4 studied maize lines. (c) Venn diagram showing the number of overlapping DEPs across pairwise comparisons among 3 groups of GM/non-GM maize seeds. (d) Cluster map comparing the DEP regulation patterns. Red indicates relatively high expression, blue indicates relatively low expression, and white indicates the same expression levels in the two lines. All the MS data were normalized and then applied for cluster analysis

In total, 3600, 3508 and 3419 proteins were identified from three independent iTRAQ replicates, for a total of 6874 proteins (Supplementary Table S3). Among the identified proteins, 2569 simultaneously appeared in three replicates. The principal components (PCs) analysis obtained PC1(56.04%) and PC2(23.97%) two main components. Under the influence of the inter-batch effect, the repeated samples did not cluster together well, but the samples in the batch tended to gather (Fig. 1a). Cluster analysis of the identified proteins showed that the protein expression patterns of 2A-7 and 2A-7×CC-2 shared the highest similarity among the 4 studied maize lines. The protein expression patterns of 2A-7×CC-2 and Zheng58 shared higher similarity than those of 2A-7 and CC-2 (Fig. 1b).

### 3.3 Identification of Maize DEPs and co-DEPs in Seeds

Proteins with a fold change greater than 2.0-fold or lower than 0.5-fold (*P* value ≤ 0.05) were identified as DEPs. The numbers of DEPs in the different comparison groups are summarized in Table 1. There were 120 maize DEPs identified in the 2A-7/Zheng58 comparison group, including 57 upregulated proteins and 63 downregulated proteins (Supplementary Table S4). Two hundred seventy-one maize DEPs were identified by comparison of CC-2 with Zheng58, 48 of which were upregulated and 223 of which were downregulated (Supplementary Table S5). There were 135 maize DEPs identified in the 2A-7×CC-2/Zheng58 comparison group, including 58 upregulated proteins and 77 downregulated proteins (Supplementary Table S6). Among these DEPs, 29 were simultaneously identified in the three comparison groups and named co-DEPs (Fig. 1c). Except for co-DEP Q0QW12, the regulatory trend of the other 28 co-DEPs was consistent among the three comparison groups (Fig. 1d).

**Table 1.** Number of identified maize DEPs

Comparison group	Upregulated	Downregulated	All sig diff
2A-7/Zheng58	57	63	120
CC-2/Zheng58	48	223	271
2A-7×CC-2/Zheng58	58	77	135

### 3.4 Analysis of the Maize DEPs Identified

Gene Ontology (GO) annotation and enrichment were performed using the Gene Ontology database to reveal the functions of the identified DEPs, including molecular functions, biological processes, and cellular components. The DEPs of 2A-7/Zheng58 were classified into 34 functional groups (Supplementary Figure S2A). The DEPs in the biological processes category are mainly involved in the response to temperature stimulus and defense response (Supplementary Figure S2B). The CC-2/Zheng58 DEPs were annotated into 43 functional groups (Supplementary Figure S3A). In the biological processes category, the DEPs participate in the response to stress and oxidation reduction (Supplementary Figure S3B). The DEPs for 2A-7×CC-2/Zheng58 were sorted into 35 functional groups (Supplementary Figure S4A), and the biological processes category of the DEPs are mainly involved in oxidation reduction and homeostatic processes (Supplementary Figure S4B).

**Figure 2.**
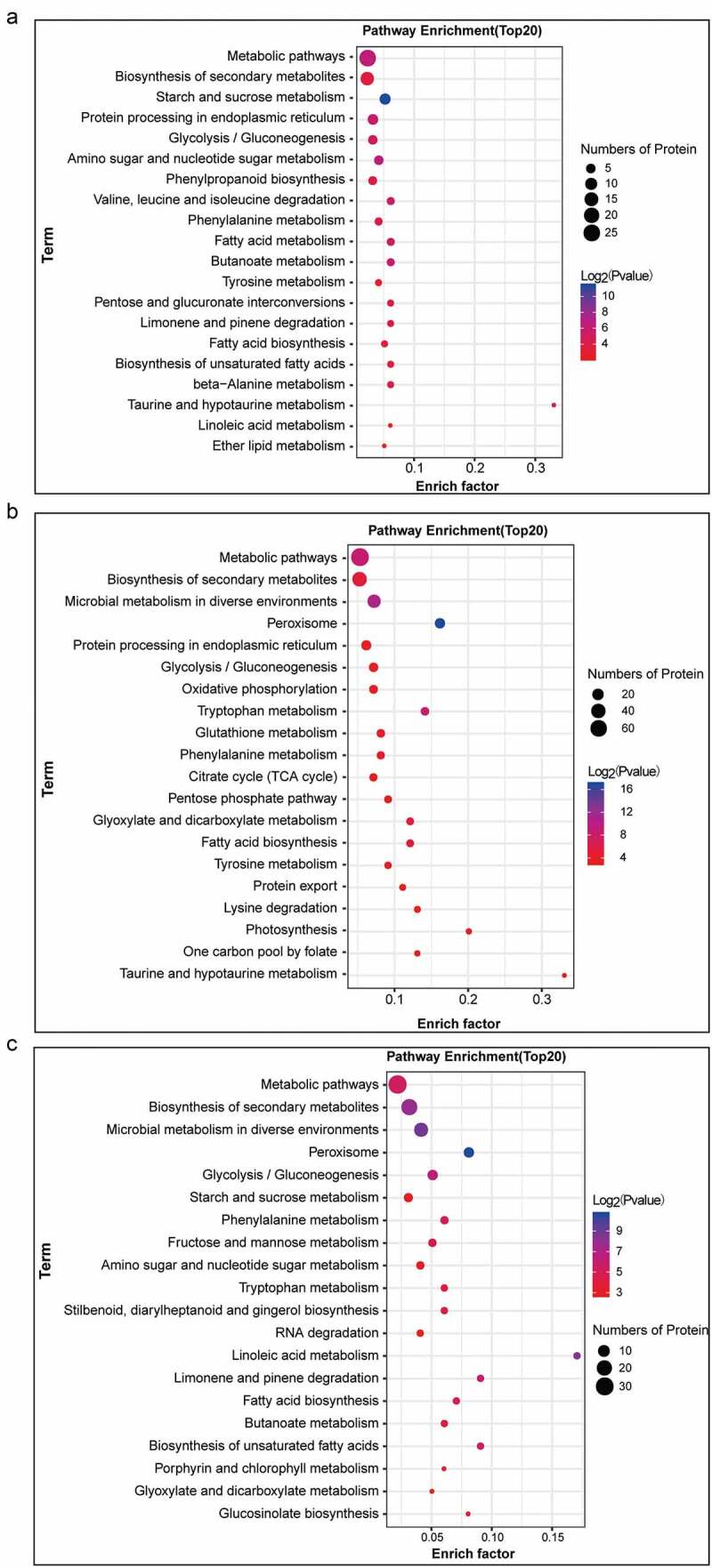
KEGG pathway enrichment analysis of DEPs in 2A-7/Zheng58 (a), CC-2/Zheng58 (b), and 2A-7×CC-2/Zheng58 (c)

KEGG pathway annotation and enrichment analysis of the identified DEPs was carried out with the KEGG pathway database. The DEPs identified in the 2A-7/Zheng58 comparison group are mainly involved in metabolic pathways (ko01100) and biosynthesis of secondary metabolites (ko01110), followed by starch and sucrose metabolism (ko00500) and protein processing in the endoplasmic reticulum (ko04141) (Fig. 2a). The DEPs obtained from CC-2/Zheng58 and 2A-7×CC-2/Zheng58 comparisons mainly participate in metabolic pathways (ko01100) and biosynthesis of secondary metabolites (ko01110), followed by microbial metabolism in diverse environments (ko01120) (Fig. 2b and 2c).

### 3.5 Metabolite Profiling of Maize Seeds

**Figure 3.**
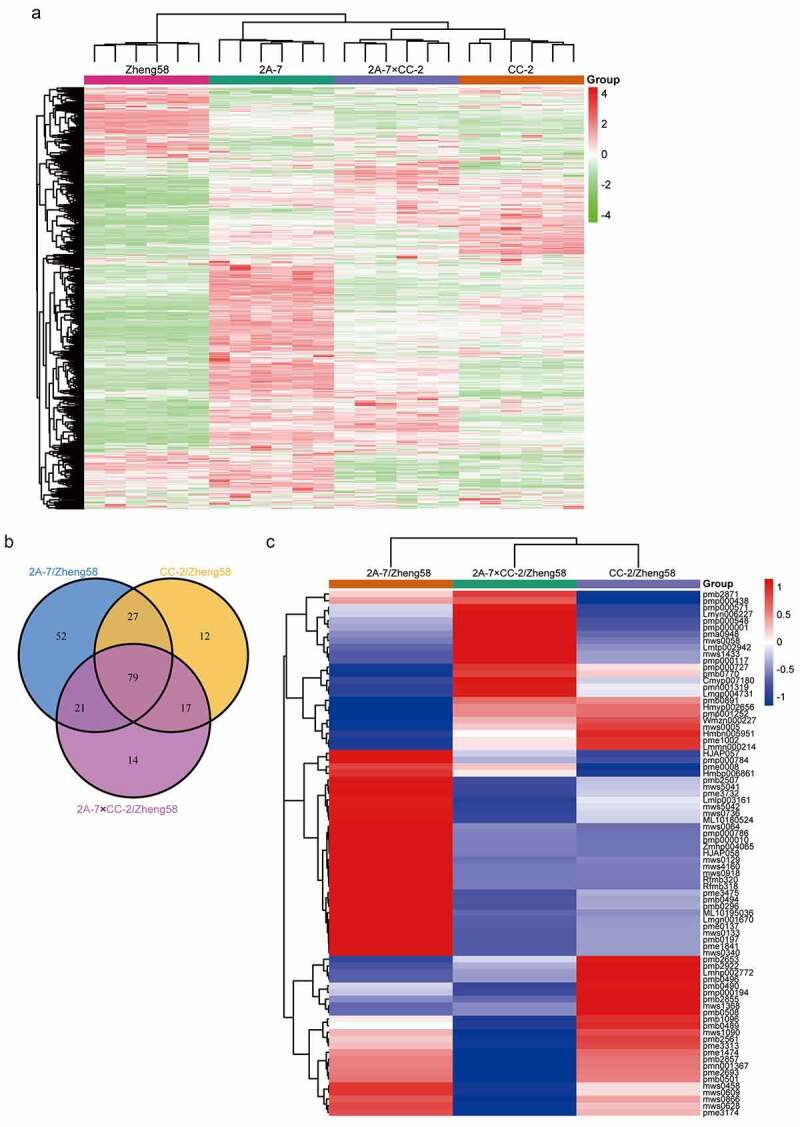
**Metabolite abundance pattern analysis**. (a) Cluster map comparing the metabolite abundance patterns of the 4 studied maize lines. Red indicates relatively high abundance, green indicates relatively lower abundance, and white indicates the same levels in the two lines. (b) Venn diagram showing the number of overlapping DAMs across pairwise comparisons among 3 groups of GM/non-GM maize seeds. (c) Cluster map comparing the DAM regulation patterns. Red indicates relatively high abundance, green indicates relatively lower abundance, and white indicates the same levels in the two lines. All the MS data were normalized and then used in cluster analysis

There were 616 metabolites successfully detected in maize seeds by widely targeted metabolomics (Supplementary Table S7). The metabolites detected are diverse, and they were classified into 32 classes, including amino acids and derivatives, phenolic acids, organic acids, nucleotides and derivatives, sugar alcohols, etc. Cluster analysis of the identified metabolites showed that the metabolite profiles of 2A-7×CC-2 and CC-2 share the highest similarity among the 4 studied maize lines. The metabolite profiles of 2A-7×CC-2 and 2A-7 share higher similarity than those of 2A-7 and Zheng58 (Fig. 3a).

### 3.6 Identification of DAMs and co-DAMs in Maize Seeds

Metabolites with changes in abundance greater than 2.0-fold or lower than 0.5-fold (VIP ≥ 1) were identified as DAMs. The numbers of DAMs in the different comparison groups are summarized in Table 2. There were 179 DAMs identified in the 2A-7/Zheng58 comparison group, including 164 increased and 15 decreased metabolites (Supplementary Table S8). In total, 135 DAMs were identified by comparison of CC-2 with Zheng58, 109 of which were increased and 26 decreased (Supplementary Table S9). There were 131 DAMs identified in the 2A-7×CC-2/Zheng58 comparison group, including 106 increased and 25 decreased metabolites (Supplementary Table S10). Among these DAMs, 79 were identified in three comparison groups at the same time and named co-DAMs (Fig. 3b). The regulatory trend of these co-DAMs is illustrated in Fig. 3c.

**Table 2.** Number of identified DAMs

Comparison group	Upregulated	Downregulated	All sig diff
2A-7/Zheng58	164	15	179
CC-2/Zheng58	109	26	135
2A-7×CC-2/Zheng58	106	25	131

### 3.7 Analysis of the Identified DAMs

**Figure 4.**
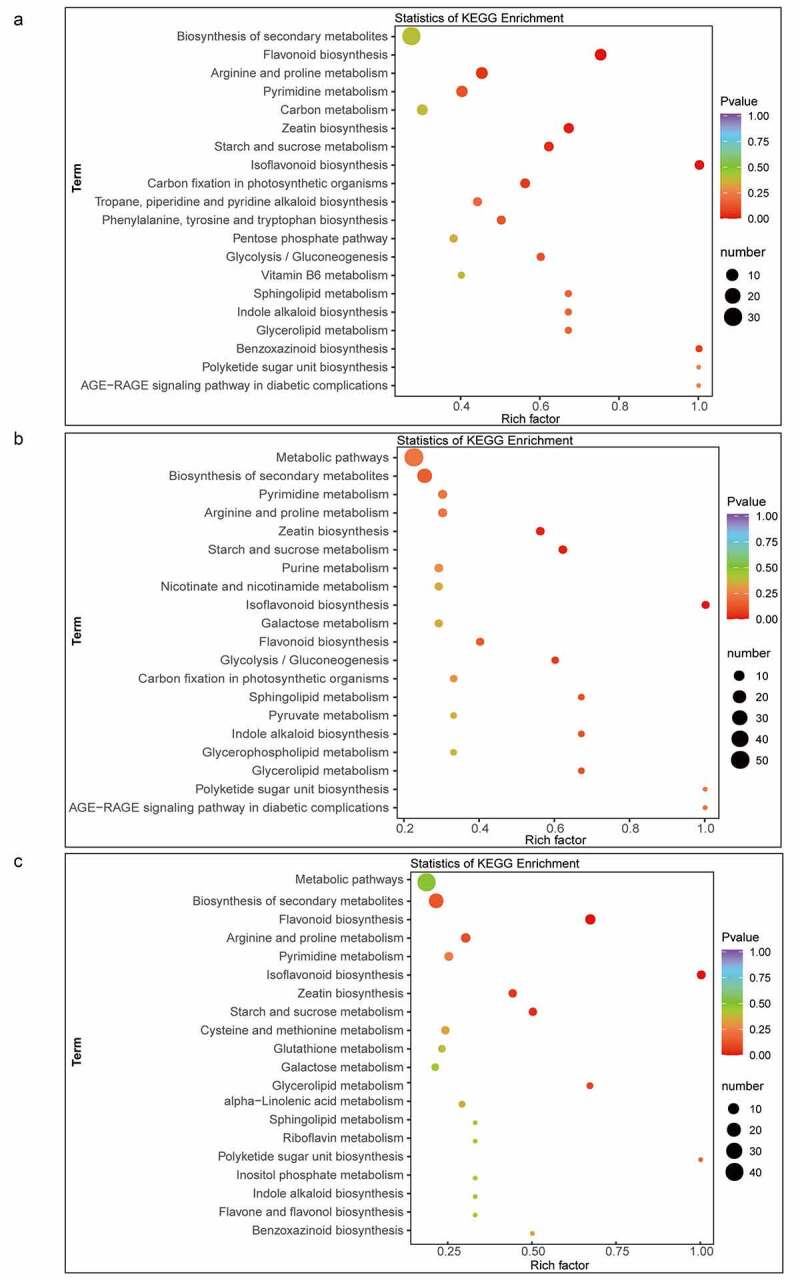
KEGG pathway enrichment analysis of DAMs in 2A-7/Zheng58 (a), CC-2/Zheng58 (b), and 2A-7×CC-2/Zheng58 (c)

KEGG pathway enrichment analysis of the identified DAMs was carried out with the KEGG pathway database. The DAMs identified in the 2A-7/Zheng58 comparison group are mainly involved in the biosynthesis of secondary metabolites (ko01110), followed by flavonoid biosynthesis (ko00941) and arginine and proline metabolism (ko00330) (Fig. 4a). The DAMs obtained from the CC-2/Zheng58 comparison mainly participate in metabolic pathways (ko01100) and biosynthesis of secondary metabolites (ko01110), followed by pyrimidine metabolism (ko00240) (Fig. 4b). The DAMs detected from the 2A-7×CC-2/Zheng58 comparison group is primarily associated with metabolic pathways (ko01100) and biosynthesis of secondary metabolites (ko01110), followed by flavonoid biosynthesis (ko00941) (Fig. 4c).

### 3.8 Integrated Proteomics and Metabolomics Analyses

Integrated proteomics and metabolomics analyses showed that DEPs are involved in 68, 113 and 80 KEGG pathways in the 2A-7/Zheng58, CC-2/Zheng58 and 2A-7×CC-2/Zheng58 comparison groups. DAMs are involved in 67, 67 and 48 KEGG pathways in 2A-7/Zheng58, CC-2/Zheng58 and 2A-7×CC-2/Zheng58 comparisons. These identified DEPs and DAMs participate in 30, 48 and 22 common KEGG (co-KEGG) pathways in the three comparison groups (Table 3). Further data analysis showed involvement in KEGG pathways for 6 co-DEPs and 30 co-DAMs (Tables 4 and Tables 5). The introduction of exogenous EPSPS did not affect the expression levels of six other enzymes, DAHP synthase, 3-dehydroquinate synthase, 3-dehydroquinate dehydratase, shikimate dehydrogenase, shikimate kinase and chorismate synthase, or cause the abundance of seven metabolites, 3-dehydroquinate, 3-dehydroshikimate, shikimate, shikimate-3-phosphate, 5-O-(1-carboxyvinyl)-3-phosphoshikimate, chorismate and prephenate, involved in the shikimic acid pathway in seeds of the GM maize lines CC-2 and 2A-7×CC-2 (Supplementary Figure S5 and S6).

**Table 3.** Number of common metabolic pathways based on integrated proteomics and metabolomics analyses

No. of KEGG pathways	Comparison group		
	2A-7/Zheng58	CC-2/Zheng58	2A-7×CC-2/Zheng58
DEP	68	113	80
DAM	67	67	48
co-KEGG pathways	30	48	22

**Table 4.** Co-DEPs identified and annotated to KEGG pathways

No.	Accession	Name	Diff_state	KEGG pathways
1	E9JVD4	Aldose reductase	Down	Microbial metabolism in diverse environments; Biosynthesis of secondary metabolites; Butanoate metabolism; Fructose and mannose metabolism; Metabolic pathways; Glycerolipid metabolism; Glycolysis/gluconeogenesis; Linoleic acid metabolism
2	Q0QWI2	Sorbitol dehydrogenase	Up	Metabolic pathways; Fructose and mannose metabolism
3	B6THG0	Peroxidase	Up	Metabolic pathways; Phenylalanine metabolism; Biosynthesis of secondary metabolites; Phenylpropanoid biosynthesis
4	B8A046	Phenylalanine ammonia-lyase	Up	Biosynthesis of secondary metabolites; Phenylalanine metabolism; Phenylpropanoid biosynthesis; Nitrogen metabolism; Metabolic pathways
5	A0A1D6HP35	Glutathione reductase	Up	Glutathione metabolism
6	Q9SPJ8	Cell wall invertase	Up	Starch and sucrose metabolism; Metabolic pathways; Galactose metabolism

**Table 5.** coDAMs detected and annotated to KEGG pathways

No.	Index	Name	Diff_state	KEGG pathways
1	pme2693	N-Acetylputrescine	up	Arginine and proline metabolism; Metabolic pathways
2	mws0005	Tryptamine	up	Tryptophan metabolism; Biosynthesis of secondary metabolites; Metabolic pathways
3	mws1090	Glucose-1-phosphate	up	Pentose and glucuronate interconversions; Galactose metabolism; Starch and sucrose metabolism; Biosynthesis of secondary metabolites; Metabolic pathways; Amino sugar and nucleotide sugar metabolism; Glycolysis/gluconeogenesis; Glycerolipid metabolism
4	Lmyn006227	Galangin (3,5,7-Trihydroxyflavone)	up	Flavonoid biosynthesis
5	mws0628	4-Hydroxybenzaldehyde	down	Metabolic pathways
6	pme1474	5ʹ-Deoxy-5ʹ-(methylthio)adenosine	up	Metabolic pathways; Zeatin biosynthesis
7	mws0866	D-Glucose 6-phosphate	up	Starch and sucrose metabolism; Biosynthesis of secondary metabolites; Metabolic pathways
8	pmb0501	Agmatine	up	Metabolic pathways; Arginine and proline metabolism
9	mws0609	Guanosine 3ʹ,5ʹ-cyclic monophosphate	up	Purine metabolism; Metabolic pathways
10	pmb2507	2-Deoxyribose-1-phosphate	up	Pyrimidine metabolism; Pentose phosphate pathway; Metabolic pathways
11	pmb0508	p-Coumaroylagmatine	up	Arginine and proline metabolism; Metabolic pathways
12	pme3174	Cytidine 5ʹ-monophosphate(Cytidylic acid)	up	Pyrimidine metabolism; Metabolic pathways
13	Lmlp003161	N-Feruloylputrescine	up	Arginine and proline metabolism; Metabolic pathways
14	mws0458	Vanillin	down	Biosynthesis of secondary metabolites; Metabolic pathways
15	pmb0496	N-Feruloylagmatine	up	Arginine and proline metabolism; Metabolic pathways
16	pme3313	D-Fructose 6-phosphate	up	Galactose metabolism; Starch and sucrose metabolism; Carbon fixation in photosynthetic organisms; Biosynthesis of secondary metabolites; Metabolic pathways
17	pme1841	Cadaverine	up	Biosynthesis of secondary metabolites; Metabolic pathways; Glutathione metabolism; Lysine degradation; Tropane, piperidine and pyridine alkaloid biosynthesis
18	pmp000571	Apigenin	up	Biosynthesis of secondary metabolites; Metabolic pathways; Flavonoid biosynthesis
19	pmb0490	p-Coumaroylputrescine	up	Metabolic pathways; Arginine and proline metabolism
20	pme3732	Cytidine	up	Pyrimidine metabolism; Metabolic pathways
21	pme3475	Butin	up	Flavonoid biosynthesis
22	pmb0891	Cis-Zeatin-7-N-glucoside	down	Zeatin biosynthesis; Biosynthesis of secondary metabolites
23	mws0133	Nicotinamide	up	Nicotinate and nicotinamide metabolism; Metabolic pathways
24	ML10195036	3-Dehydrosphinganine	up	Sphingolipid metabolism; Metabolic pathways
25	pme0008	L-Citrulline	up	Biosynthesis of secondary metabolites Metabolic pathways
26	mws0064	Eriodictyol (5,7,3ʹ,4ʹ-Tetrahydroxyflavanone)	up	Flavonoid biosynthesis; Biosynthesis of secondary metabolites; Metabolic pathways
27	pmb1096	Indole	up	Tryptophan metabolism; Phenylalanine, tyrosine and tryptophan biosynthesis; Biosynthesis of secondary metabolites; Metabolic pathways; Benzoxazinoid biosynthesis
28	Lmgn001670	Salicylic acid	down	Biosynthesis of secondary metabolites; Metabolic pathways; Phenylalanine metabolism
29	pmb2922	Uridine 5ʹ-diphospho-D-glucose	up	Amino sugar and nucleotide sugar metabolism; Ascorbate and aldarate metabolism; Biosynthesis of secondary metabolites; Galactose metabolism; Glycerolipid metabolism; Metabolic pathways; Pentose and glucuronate interconversions; Pyrimidine metabolism; Starch and sucrose metabolism; Zeatin biosynthesis
30	pme1002	L-Tyramine	up	Biosynthesis of secondary metabolites; Isoquinoline alkaloid biosynthesis; Metabolic pathways; Tyrosine metabolism

### 3.9 qRT-PCR Analysis of the co-DEPs Involved in the KEGG Pathway

**Figure 5.**
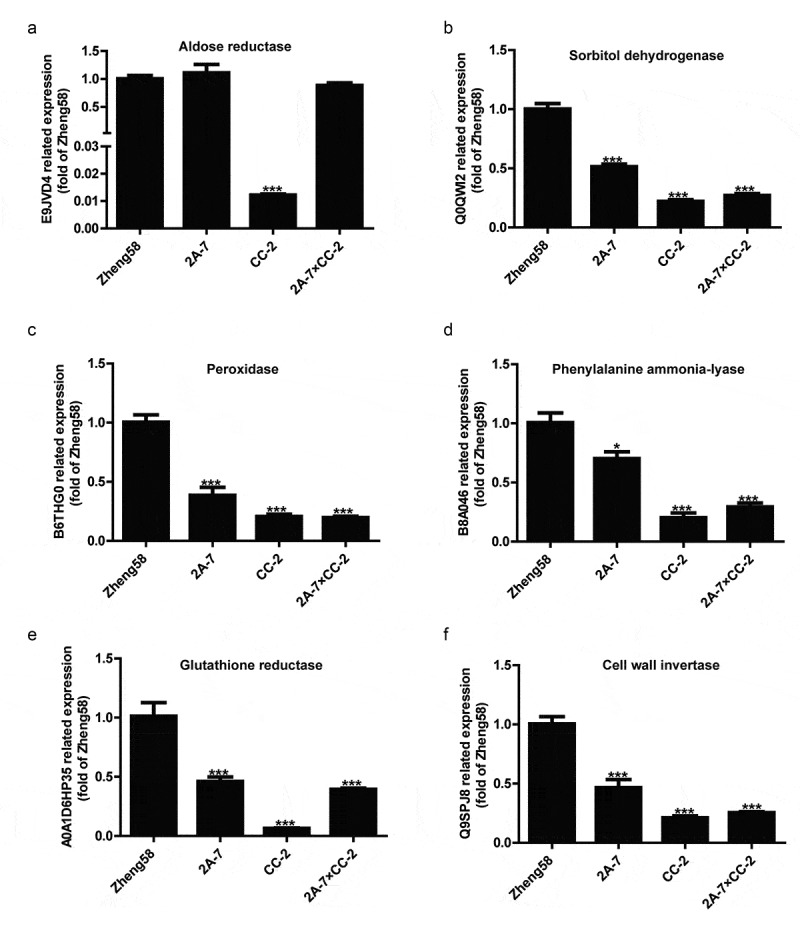
**qRT-PCR analysis of the gene expression patterns of selected co-DEPs among 3 comparison groups of GM/non-GM maize seeds**. (a) Aldose reductase, (b) sorbitol dehydrogenase, (c) peroxidase, (d) phenylalanine ammonia-lyase, (e) glutathione reductase, and (f) cell wall invertase. Error bars represent the standard deviation (SD) among the three replicates. The asterisks represent significant differences compared with A3525, as indicated by the t-test (* *p* < .05, ** *p* < .01 and *** *p* < .001)

Six co-DEPs involved in the KEGG pathways, aldose reductase, sorbitol dehydrogenase, peroxidase, phenylalanine ammonia-lyase, glutathione reductase and cell wall invertase, were selected for qRT-PCR analysis to detect their transcriptional levels. Aldose reductase was downregulated in the seeds of GM maize line CC-2 but not significantly changed in the seeds of the other two GM maize lines 2A-7 and 2A-7×CC-2. The other five co-DEPs were downregulated in the seeds of all three GM maize lines (Fig. 5).

### 3.10 Exogenous Protein Detection by iTRAQ and ELISA in Seeds of GM Maize Lines

Three exogenous proteins, Cry1Ab, Cry2Ab and EPSPS, were detected as DEPs. ELISA data showed that the content of Cry1Ab was 1.81 µg/g and 2.02 µg/g and the content of Cry2A was 0.31 µg/g and 0.31 µg/g in GM maize seeds 2A-7 and 2A-7×CC-2, respectively. The content of EPSPS was 20.00 µg/g and 28.70 µg/g in GM maize seeds CC-2 and 2A-7× CC-2, respectively (Table 6).

**Table 6.** Exogenous DEPs detected by iTRAQ and ELISA

Comparison group	Exogenous proteins	Fold change	P value	Protein content (µg/g)
2A-7/Zheng58	Cry1Ab	20.3964	0.0004	1.81 ± 0.07
	Cry2Ab	9.9649	0.0017	0.31 ± 0.01
CC-2/Zheng58	EPSPS	32.9403	0.0003	20.00 ± 1.19
2A-7×CC-2/Zheng58	Cry1Ab	23.1555	0.0004	2.02 ± 0.02
	Cry2Ab	14.1882	0.0026	0.31 ± 0.01
	EPSPS	24.4700	0.0011	28.70 ± 1.49

## 4. Discussion

Safety assessment of GM crops is performed to demonstrate the substantive equivalence of the GM crops and their non-GM parents. In the past, a large number of safety evaluations based on oimcs analysis of GM crops have either applied nonconsumpted tissues^34–37^ or focused more on the effects of environmental factors.^38–41^ Maize seeds, as edible plant parts for food and feed with abundant protein and metabolite types and quantities, must undergo safety evaluation. Integrated proteomics and metabolomics analyses can identify proteins and metabolites involved in the same pathways, which can better reveal the biological processes of crops after genetic modification. In this study, quantitative proteomics and widely targeted metabolomics analyses were performed for three GM maize lines, 2A-7 and CC-2, and the stacked transgenic line 2A-7×CC-2, and their corresponding non-GM parent, Zheng58, grown in the same environment. There were 120, 271 and 135 maize DEPs detected in the 2A-7/Zheng58, CC-2/Zheng58 and 2A-7×CC-2/Zheng58 comparisons, respectively. In total, 179, 135 and 131 DAMs were selected in the 2A-7/Zheng58, CC-2/Zheng58 and 2A-7×CC-2/Zheng58 comparisons, respectively. Compared with GM maize 2A-7 and CC-2, gene-stacked maize 2A-7× CC-2 did not affect the protein and metabolite profiles more significantly. This result was consistent with previous publications.^42,43^

In these GM maize lines, the *bt* genes *cry1Ab* and *cry2Ab* are exogenous to plants, and the insecticidal proteins Cry1Ab and Cry2Ab are not involved in known metabolic activity in plants. Herbicide tolerance is conferred by introducing the *maroACC* gene, which encodes the EPSPS enzyme related to the shikimate pathway. The introduction of the herbicide resistance gene *maroACC* resulted in an increase in the number of DEPs in CC-2 and 2A-7×CC-2 maize seeds. The *maroACC* gene encodes EPSPS, a 3-phosphoshikimate 1-carboxyvinyltransferase and the key enzyme in the shikimic acid pathway, which is a metabolic pathway for the biosynthesis of aromatic amino acids in microorganisms and plants.^44–46^ Nevertheless, integrated proteomics and metabolomics analyses showed that the introduction of exogenous EPSPS did not affect the expression levels of six other enzymes or the abundance of seven metabolites involved in the shikimic acid pathway in seeds of the GM maize lines CC-2 and 2A-7×CC-2 (Supplementary Figure S5 and S6). Insertion of the *maroACC* gene may interfere with other biological pathways than the shikimic acid pathway.

Integrated proteomics and metabolomics analyses identified 6 co-DEPs annotated in the KEGG pathways, aldose reductase, sorbitol dehydrogenase, peroxidase, phenylalanine ammonia-lyase, glutathione reductase and cell wall invertase, which were selected for qRT-PCR to assess gene expression. Aldose reductase showed similar downregulated patterns at the protein and transcription levels in the CC-2/Zheng58 comparison group but showed no change at the transcription level in the other two comparison groups. The other five co-DEPs showed opposite regulation patterns at the translation and transcription levels. These results are consistent with previously reported results for GM crops,^28,47^ which might be due to the expression levels of proteins depending on the synergy of multiple biological processes, such as transcription, posttranscriptional modification, translation and posttranslational modification. Several endoplasmic reticulum-associated proteins, such as heat shock proteins Hsp40, Hsp70, sHSP and protein disulfide-isomerase (PDIs), were identified as DEPs. These proteins work as molecular chaperones or catalyze the rearrangement of -S-S- bonds in proteins to prevent protein denaturation, restore protein conformation and biological activity, and degrade misfolded proteins, which might be the mechanism underlying the self-protection stimulated by the expression of exogenous genes in GM maize seeds.

## 5. Conclusions

Integrated iTRAQ quantitative proteomics and widely targeted metabolomics were used to evaluate changes in the protein and metabolite profiles of maize seeds caused by both transgenic modifications and gene stacking. Pathway enrichment showed that these DEPs and DAMs mainly participate in metabolic pathways and biosynthesis of secondary metabolites. Except for EPSPS, shikimate pathway-related proteins and metabolites were not identified as DEPs and DAMs, respectively. Gene stacking did not affect protein and metabolite profiles more significantly.

## Supplementary Material

Supplemental Material

## References

[1] Estruch JJ, Carozzi NB, Desai N, Duck NB, Warren GW, Koziel MG. Transgenic plants: an emerging approach to pest control. Nat Biotechnol. 1997;15(2):137–41. doi:10.1038/Nbt0297-137. 9035137

[2] http://www.isaaa.org/gmapprovaldatabase/default.asp

[3] Conner AJ, Jacobs JM. Genetic engineering of crops as potential source of genetic hazard in the human diet. Mutat Res. 1999;443(1–2):223–34. doi:10.1016/S1383-5742(99)00020-4. 10415441

[4] Conner AJ, Jacobs JM. Food risks from transgenic crops in perspective. Nutrition. 2000;16(7–8):709–11. doi:10.1016/S0899-9007(00)00331-2. 10906607

[5] Fiaz. S, X. W, Afifa Y, Alharthi B, Ali H. Apomixis and stratgeies for induce apomixis to preserve hybrid seed vigor for multiple generations. GM Foods and Crop. 2020. doi:10.1080/21645698.2020.1808423. PMC7553744 32877304

[6] Ren YF, Lv J, Wang H, Li L, Peng Y, Qu L-J. A comparative proteomics approach to detect unintended effects in transgenic Arabidopsis. J Genetics Genomics. 2009;36(10):629–39. doi:10.1016/S1673-8527(08)60155-1. 19840761

[7] Cellini F, Chesson A, Colquhoun I, Constable A, Davies HV, Engel KH, Gatehouse AMR, Kärenlampi S, Kok EJ, Leguay-J-J, *et al*. Unintended effects and their detection in genetically modified crops. Food Chem Toxicol. 2004;42(7):1089–125. doi:10.1016/j.fct.2004.02.003. 15123383

[8] Li X, He XY, Luo YB, Xiao GY, Jiang XB, Huang KL. Comparative analysis of nutritional composition between herbicide-tolerant rice with bar gene and its non-transgenic counterpart. J Food Compos Anal. 2008;21(7):535–39. doi:10.1016/j.jfca.2008.06.001.

[9] Han JH, Yang YX, Chen SR, Wang Z, Yang XL, Wang GD, Men JH. Comparison of nutrient composition of parental rice and rice genetically modified with cowpea trypsin inhibitor in China. J Food Compos Anal. 2005;18(4):297–302. doi:10.1016/j.jfca.2004.11.001.

[10] Ricroch AE, Berge JB, Kuntz M. Evaluation of genetically engineered crops using transcriptomic, proteomic, and metabolomic profiling techniques. Plant Physiol. 2011;155(4):1752–61. doi:10.1104/pp.111.173609. 21350035 PMC3091128

[11] Barros E, Lezar S, Anttonen MJ, van Dijk JP, Röhlig RM, Kok EJ, Engel K-H. Comparison of two GM maize varieties with a near-isogenic non-GM variety using transcriptomics, proteomics and metabolomics. Plant Biotechnol J. 2010;8(4):436–51. doi:10.1111/j.1467-7652.2009.00487.x. 20132517

[12] Bedair M, Glenn KC. Evaluation of the use of untargeted metabolomics in the safety assessment of genetically modified crops. Metabolomics. 2020;16(10). doi:10.1007/s11306-020-01733-8. PMC7547035 33037482

[13] Qin J, Gu F, Liu D, Yin C, Zhao S, Chen H, Zhang J, Yang C, Zhan X, Zhang M, *et al*. Proteomic analysis of elite soybean Jidou17 and its parents using iTRAQ-based quantitative approaches. Proteome Sci. 2013;11(1):12. doi:10.1186/1477-5956-11-12. 23531047 PMC3622570

[14] Qin J, Zhang J, Wang F, Wang J, Zheng Z, Yin C, Chen H, Shi A, Zhang B, Chen P, *et al*. iTRAQ protein profile analysis of developmental dynamics in soybean [Glycine max (L.) Merr.] leaves. Plos One. 2017;12(9):e0181910. doi:10.1371/journal.pone.0181910. 28953898 PMC5617144

[15] Zeng WY, Sun Z, Cai Z, Chen H, Lai Z, Yang S, Tang X. Proteomic analysis by iTRAQ-MRM of soybean resistance to Lamprosema indicate. Bmc Genomics. 2017;18(1). doi:10.1186/s12864-017-3825-0. PMC5461738 28587595

[16] Ji W, Cong R, Li S, Li R, Qin Z, Li Y, Zhou X, Chen S, Li J. Comparative proteomic analysis of soybean leaves and roots by iTRAQ provides insights into response mechanisms to short-term salt stress. Front Plant Sci. 2016:7. doi:10.3389/fpls.2016.00573. PMC4850148 27200046

[17] Lim S, Borza T, Peters RD, Coffin RH, Al-Mughrabi KI, Pinto DM, Wang-Pruski G. Proteomics analysis suggests broad functional changes in potato leaves triggered by phosphites and a complex indirect mode of action against Phytophthora infestans. J Proteomics. 2013;93:207–23. doi:10.1016/j.jprot.2013.03.010. 23542353

[18] Qian D, Tian L, Qu L. Proteomic analysis of endoplasmic reticulum stress responses in rice seeds. Sci Rep. 2015;5(1):14255. doi:10.1038/srep14255. 26395408 PMC4585792

[19] Liu WX, Zhang Z LL, Jin WJ, Jin WJ, Jin W. iTRAQ-based quantitative proteomic analysis of transgenic and nontransgenicmaize seeds. J Food Compos Anal. 2020;92:103564. doi:10.1016/j.jfca.2020.103564.

[20] Schauer N, Semel Y, Roessner U, Gur A, Balbo I, Carrari F, Pleban T, Perez-Melis A, Bruedigam C, Kopka J, *et al*. Comprehensive metabolic profiling and phenotyping of interspecific introgression lines for tomato improvement. Nat Biotechnol. 2006;24(4):447–54. doi:10.1038/nbt1192. 16531992

[21] Schauer N, Semel Y, Balbo I, Steinfath M, Repsilber D, Selbig J, Pleban T, Zamir D, Fernie AR. Mode of inheritance of primary metabolic traits in tomato. Plant Cell. 2008;20(3):509–23. doi:10.1105/tpc.107.056523. 18364465 PMC2329927

[22] Alonso-Blanco C, Aarts MGM, Bentsink L, Keurentjes JJB, Reymond M, Vreugdenhil D, Koornneef M. What has natural variation taught us about plant development, physiology, and adaptation? Plant Cell. 2009;21(7):1877–96. doi:10.1105/tpc.109.068114. 19574434 PMC2729614

[23] Chen W, Gong L, Guo Z, Wang W, Zhang H, Liu X, Yu S, Xiong L, Luo J. A novel integrated method for large-scale detection, identification, and quantification of widely targeted metabolites: application in the study of rice metabolomics. Mol Plant. 2013;6(6):1769–80. doi:10.1093/mp/sst080. 23702596

[24] Zhang X, Zhang R, Li L, Yang Y, Ding Y, Guan H, Wang X, Zhang A, Wen H. Negligible transcriptome and metabolome alterations in RNAi insecticidal maize against Monolepta hieroglyphica. Plant Cell Rep. 2020;39(11):1539–47. doi:10.1007/s00299-020-02582-4. 32869121 PMC7554010

[25] Liu QS, Yang X, Tzin V, Peng Y, Romeis J, Li Y. Plant breeding involving genetic engineering does not result in unacceptable unintended effects in rice relative to conventional cross-breeding. Plant J. 2020;103(6):2236–49. doi:10.1111/tpj.14895. 32593184 PMC7540705

[26] Bradford MM. A rapid and sensitive method for the quantitation of microgram quantities of protein utilizing the principle of protein-dye binding. Anal Biochem. 1976;72(1––2):248–54. doi:10.1016/0003-2697(76)90527-3. 942051

[27] Baldrianova J, Černý M, Novák J, Jedelský PL, Divíšková E, Brzobohatý B. Arabidopsis proteome responses to the smoke-derived growth regulator karrikin. J Proteomics. 2015;120:7–20. doi:10.1016/j.jprot.2015.02.011. 25746380

[28] Wang LM, Wang X, Jin X, Jia R, Huang Q, Tan Y, Guo A. Comparative proteomics of Bt-transgenic and non-transgenic cotton leaves. Proteome Sci. 2015;13(1). doi:10.1186/s12953-015-0071-8. PMC4422549 25949214

[29] Liu YB, Zhang YX, Song SQ, Li JS, Stewart CN, Wei W, Zhao YJ, Wang WQ. A proteomic analysis of seeds from Bt-transgenic Brassica napus and hybrids with wild B. juncea. Sci Rep. 2015;5. doi: 10.1038/Srep15480. PMC4614387 26486652

[30] Yuan H, Zeng X, Shi J, Xu Q, Wang Y, Jabu D, Sang Z, Nyima T. Time-course comparative metabolite profiling under osmotic stress in tolerant and sensitive Tibetan hulless barley. Biomed Res Int. 2018;2018:9415409. doi:10.1155/2018/9415409. 30671479 PMC6323448

[31] Kanehisa M, Goto S. KEGG: kyoto encyclopedia of genes and genomes. Nucleic Acids Res. 2000;28(1):27–30. doi:10.1093/nar/28.1.27. 10592173 PMC102409

[32] Ogata H, Goto S, Sato K, Fujibuchi W, Bono H, Kanehisa M. KEGG: kyoto Encyclopedia of Genes and Genomes. Nucleic Acids Res. 1999;27(1):29–34. doi:10.1093/nar/27.1.29. 9847135 PMC148090

[33] Livak KJ, Schmittgen TD. Analysis of relative gene expression data using real-time quantitative PCR and the 2(T)(-Delta Delta C) method. Methods. 2001;25(4):402–08. doi:10.1006/meth.2001.1262. 11846609

[34] Plischke A, Choi YH, Brakefield PM, Klinkhamer PGL, Bruinsma M. Metabolomic plasticity in GM and Non-GM potato leaves in response to Aphid herbivory and virus infection. J Agr Food Chem. 2012;60:1488–93. doi:10.1021/jf204864y. 22243672 PMC3279958

[35] Zhou J, Zhang L, Chang Y, Lu X, Zhu Z, Xu G. Alteration of leaf metabolism in Bt-transgenic Rice (Oryza sativa L.) and its wild type under insecticide stress. J Proteome Res. 2012;11(8):4351–60. doi:10.1021/pr300495x. 22768924

[36] Christ B, Hochstrasser R, Guyer L, Francisco R, Aubry S, Hörtensteiner S, Weng J-K. Non-specific activities of the major herbicide-resistance gene BAR. Nat Plants. 2017;3(12):937–45. doi:10.1038/s41477-017-0061-1. 29180815 PMC6342461

[37] Hao WY, Li FW, Yan W, Li CC, Hao DY. Comparative metabolic profiling of four transgenic maize lines and two non-transgenic maize lines using high-performance liquid chromatography mass spectrometry. Acta Physiol Plant. 2017;39(8). doi:10.1007/s11306-020-01733-8.

[38] Chang YW, Zhao C, Zhu Z, Wu Z, Zhou J, Zhao Y, Lu X, Xu G. Metabolic profiling based on LC/MS to evaluate unintended effects of transgenic rice with cry1Ac and sck genes. Plant Mol Biol. 2012;78(4–5):477–87. doi:10.1007/s11103-012-9876-3. 22271304

[39] Frank T, Rohlig RM, Davies HV, Barros E, Engel KH. Metabolite profiling of maize kernels-genetic modification versus environmental influence. J Agr Food Chem. 2012;60(12):3005–12. doi:10.1021/jf204167t. 22375597

[40] Chen MJ, Rao RSP, Zhang YM, Zhong C, Thelen JJ. Metabolite variation in hybrid corn grain from a large-scale multisite study. Crop J. 2016;4(3):177–87. doi:10.1016/j.cj.2016.03.004.

[41] Tang WJ, Hazebroek J, Zhong C, Harp T, Vlahakis C, Baumhover B, Asiago V. Effect of genetics, environment, and phenotype on the metabolome of maize hybrids using GC/MS and LC/MS. J Agr Food Chem. 2017;65(25):5215–25. doi:10.1021/acs.jafc.7b00456. 28574696

[42] Wang XJ, Zhang X, Yang JT, Wang ZX. Effect on transcriptome and metabolome of stacked transgenic maize containing insecticidal cry and glyphosate tolerance epsps genes. Plant J. 2018;93(6):1007–16. doi:10.1111/tpj.13825. 29356248

[43] Weber N, Halpin C, Hannah LC, Jez JM, Kough J, Parrott W. Editor’s choice: crop genome plasticity and its relevance to food and feed safety of genetically engineered breeding stacks. Plant Physiol. 2012;160(4):1842–53. doi:10.1104/pp.112.204271. 23060369 PMC3510115

[44] Zabalza A, Orcaray L, Fernandez-Escalada M, Zulet-Gonzalez A, Royuela M. The pattern of shikimate pathway and phenylpropanoids after inhibition by glyphosate or quinate feeding in pea roots. Pestic Biochem Phys. 2017;141:96–102. doi:10.1016/j.pestbp.2016.12.005. 28911748

[45] Herrmann KM. The shikimate pathway - early steps in the biosynthesis of aromatic-compounds. Plant Cell. 1995;7(7):907–19. doi:10.2307/3870046. 12242393 PMC160886

[46] Mir R, Jallu S, Singh TP. The shikimate pathway: review of amino acid sequence, function and three-dimensional structures of the enzymes. Crit Rev Microbiol. 2015;41(2):172–89. doi:10.3109/1040841X.2013.813901. 23919299

[47] Tan YH, Tong Z, Yang Q, Sun Y, Jin X, Peng CZ, Guo AP, Wang XC. Proteomic analysis of phytase transgenic and non-transgenic maize seeds. Sci Rep. 2017;7. doi:10.1038/S41598-017-09557-8. PMC5569035 28835691

