# Supplementary material for: Integrated proteomics and metabolomics analysis of transgenic and gene-stacked maize line seeds
Source: GM Crops Food. 2021 Jun 7;12(1):361–75. doi: 10.1080/21645698.2021.1934351 (PMC8189116; doi:10.1080/21645698.2021.1934351)
Supplement: Supplemental Material [file KGMC_A_1934351_SM4891.docx]

**Title:** Integrated proteomics and metabolomics analysis of transgenic and gene stacking maize line seeds

**Running Title:** Integrated proteomics and metabolomics analysis of transgenic maize seeds

Weixiao Liu^a*^, Haiming Zhao^b^, Chaohua Miao^a^ and Wujun Jin^a*^

^a^ Biotechnology Research Institute, Chinese Agricultural and Academic Sciences, Beijing 100081, P.R. China

^b^ State Key Laboratory of Agrobiotechnology and National Maize Improvement Center, Department of Plant Genetics and Breeding, China Agricultural University, Beijing 100193, P. R. China

*Correspondence author:

Weixiao Liu Ph.D. and Wujun Jin Ph.D.

Biotechnology Research Institute, Chinese Agriculture Academy of Sciences

No.12 Zhongguancun South St., Haidian District, Beijing 100081, P.R. China

Tel.: 86-10-82109852


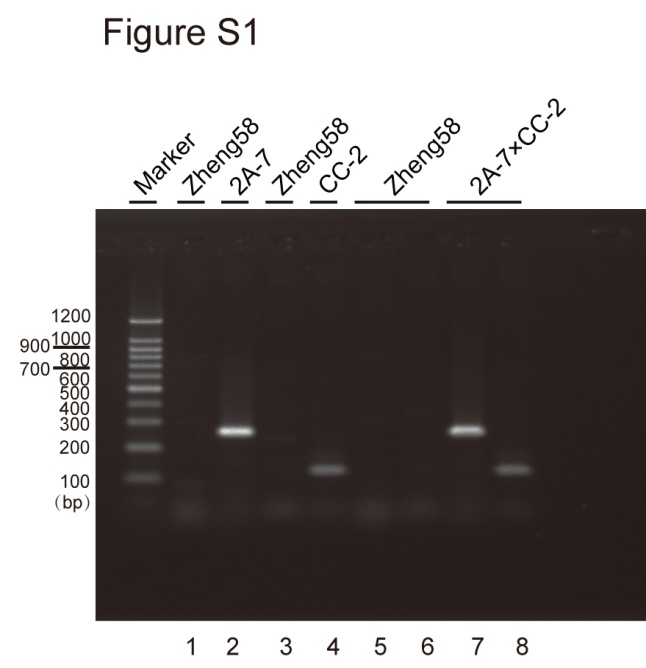


**Figure S1.** Event-specific PCR detection of the GM maize lines studied. M, 100bp DNA ladder.


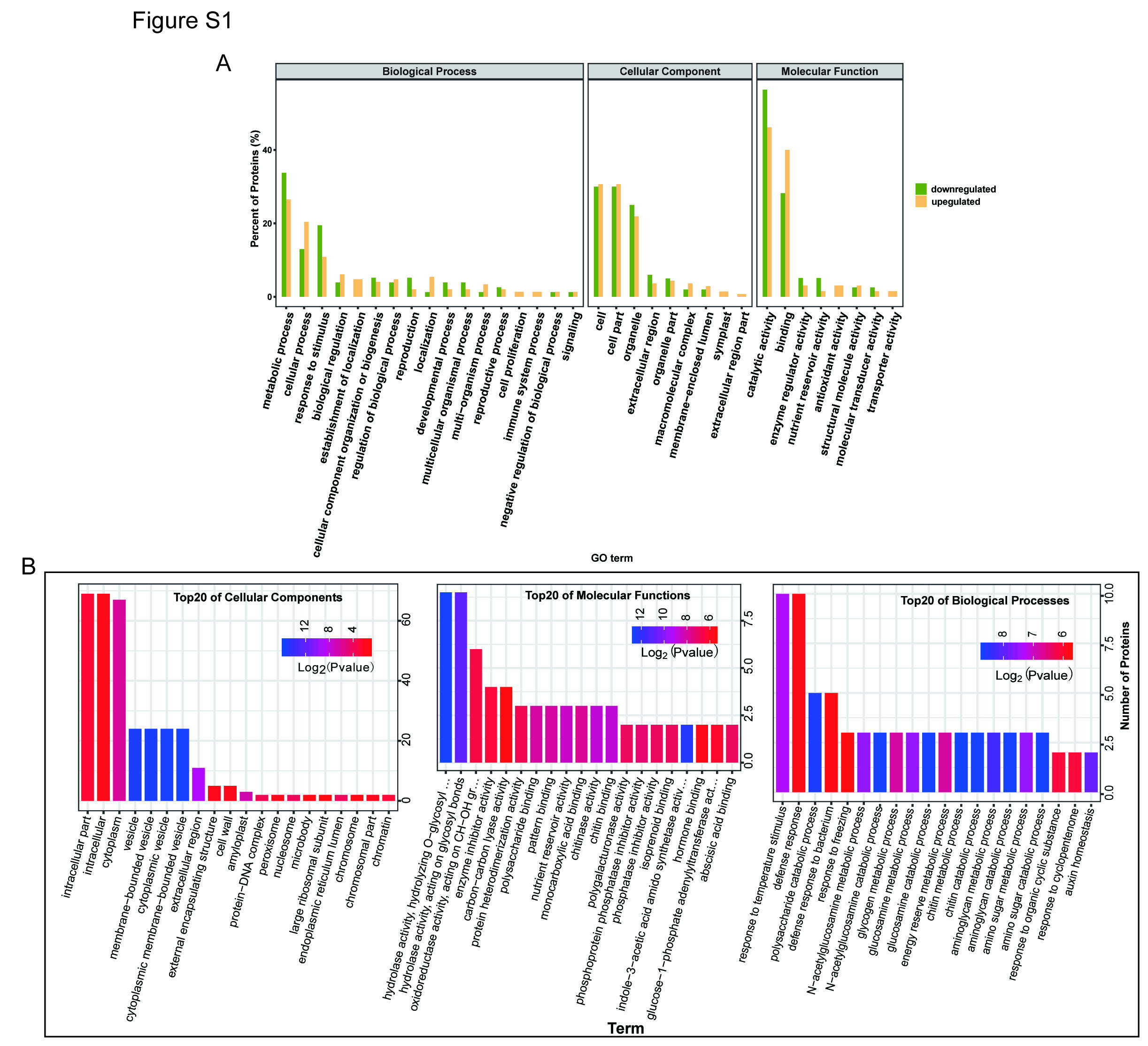


**Figure S2.** GO annotation (A) and enrichment analysis (B) of the identified DEPs in the 2A-7/Zheng58 comparison group. DEPs were annotated into 3 main categories, namely, biological process, cellular component and molecular function, to determine the functions of the identified DEPs between the GM maize line 2A-7 and the non-GM parent Zheng58. Yellow bars represent upregulated proteins, and green bars represent downregulated proteins


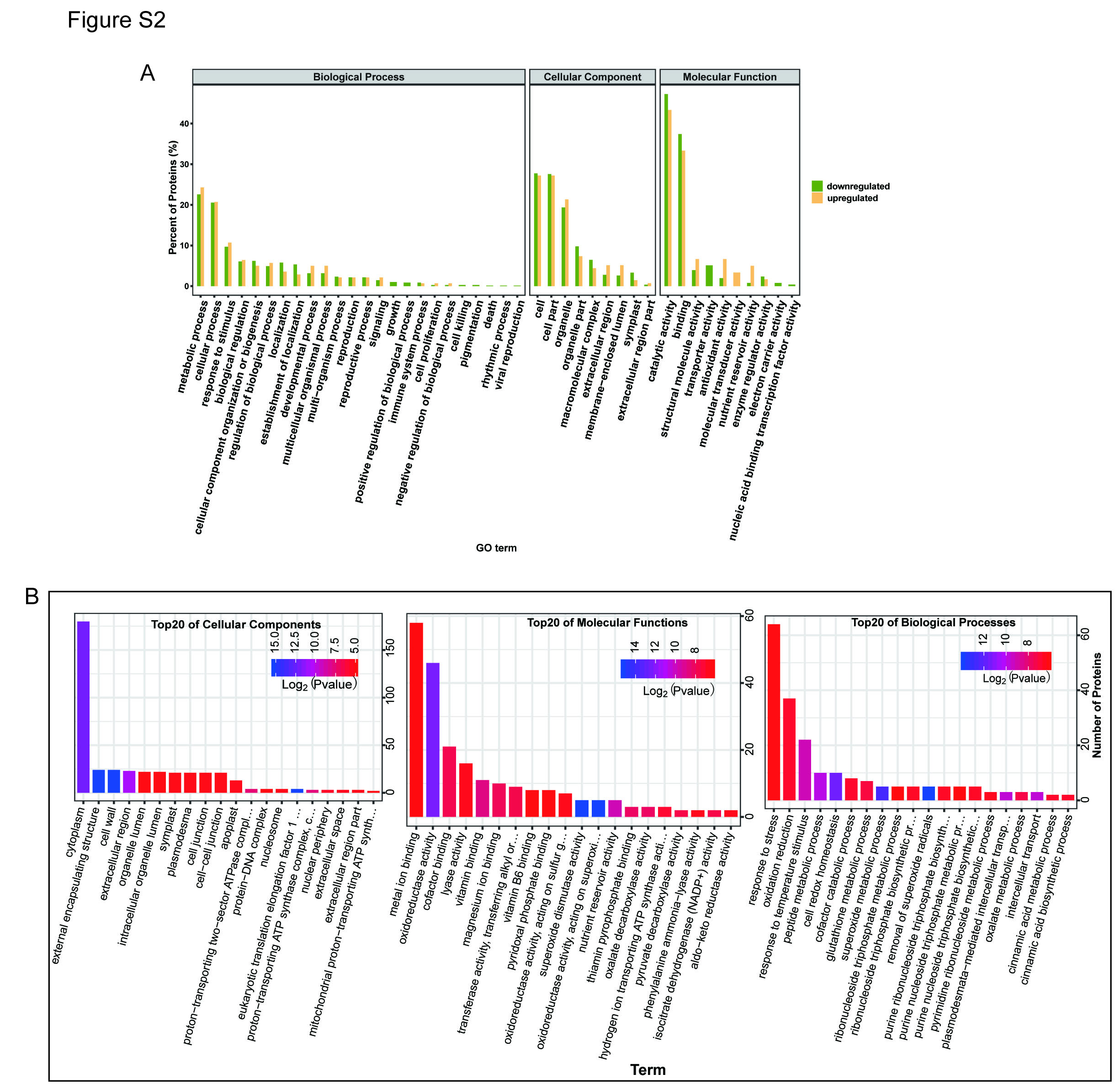


**Figure S3.** GO annotation (A) and enrichment analysis (B) of the identified DEPs in the CC-2/Zheng58 comparison group. DEPs were annotated into 3 main categories, namely, biological process, cellular component and molecular function, to determine the functions of the identified DEPs between the GM maize line CC-2 and the non-GM parent Zheng58. Yellow indicates upregulated proteins, and green indicates downregulated proteins.


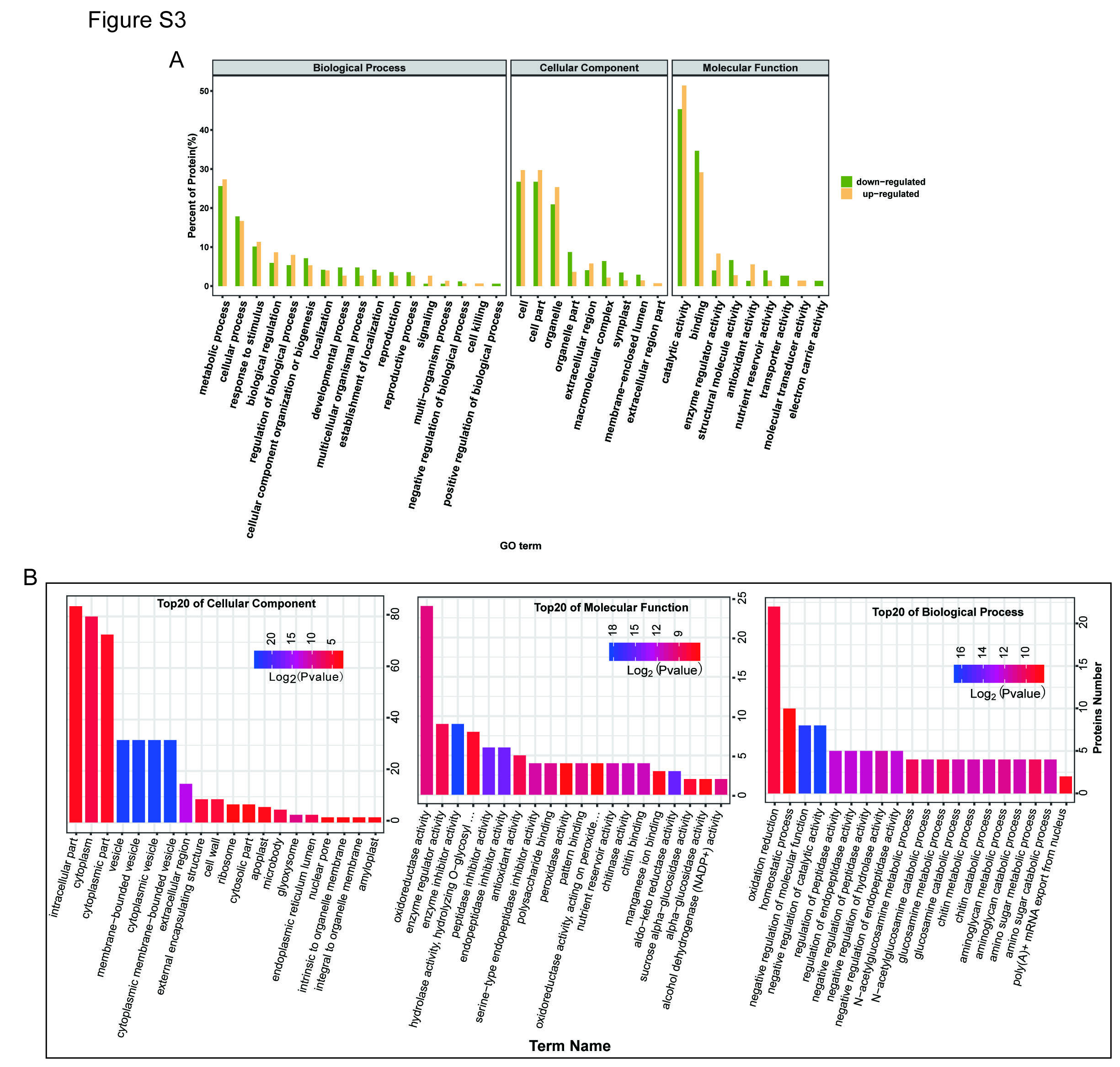


**Figure S4.** GO annotation (A) and enrichment analysis (B) of the identified DEPs in the 2A-7×CC-2/Zheng58 comparison group. DEPs were annotated into 3 main categories, namely, biological process, cellular component and molecular function, to determine the functions of the identified DEPs between the GM maize line 2A-7×CC-2 and the non-GM parent Zheng58. Yellow indicates upregulated proteins, and green indicates downregulated proteins.


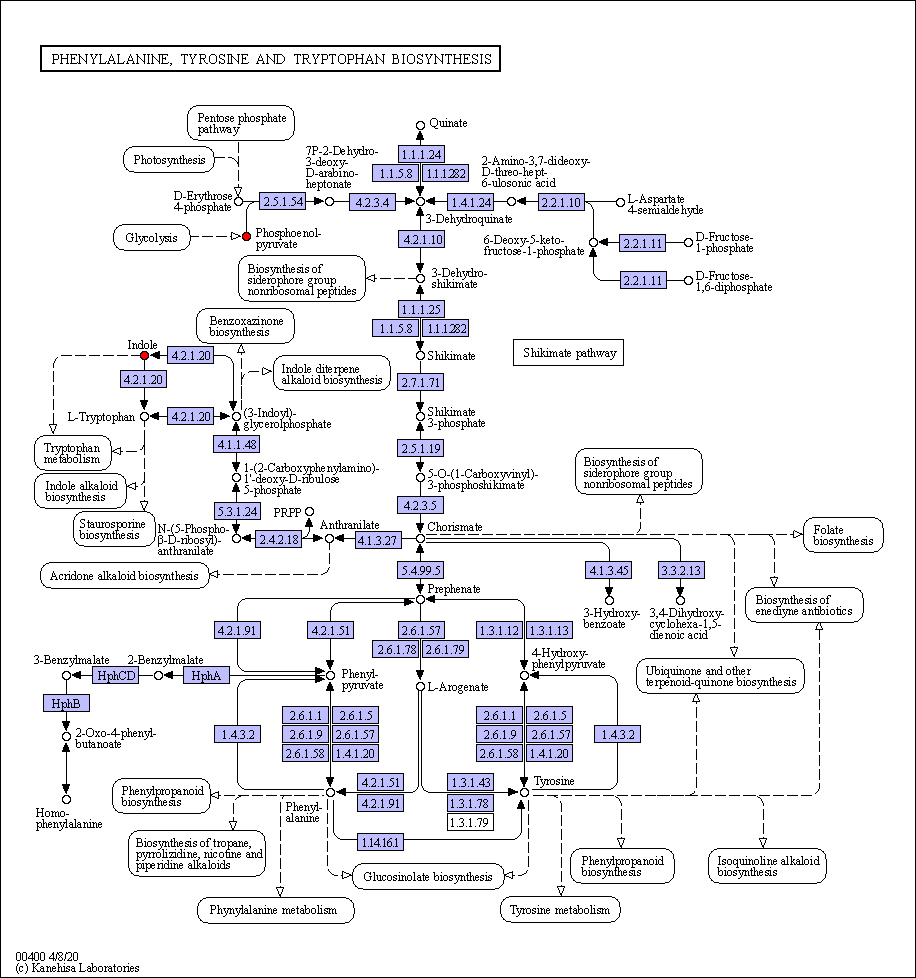


**Figure S5.** DEPs and DAMs involved in the phenylalanine, tyrosine and tryptophan biosynthesis pathways in the CC-2/Zheng58 comparison group. Red dots are upregulated metabolites, green dots are downregulated metabolites, pink rectangles are upregulated proteins, and yellow rectangles are downregulated proteins.


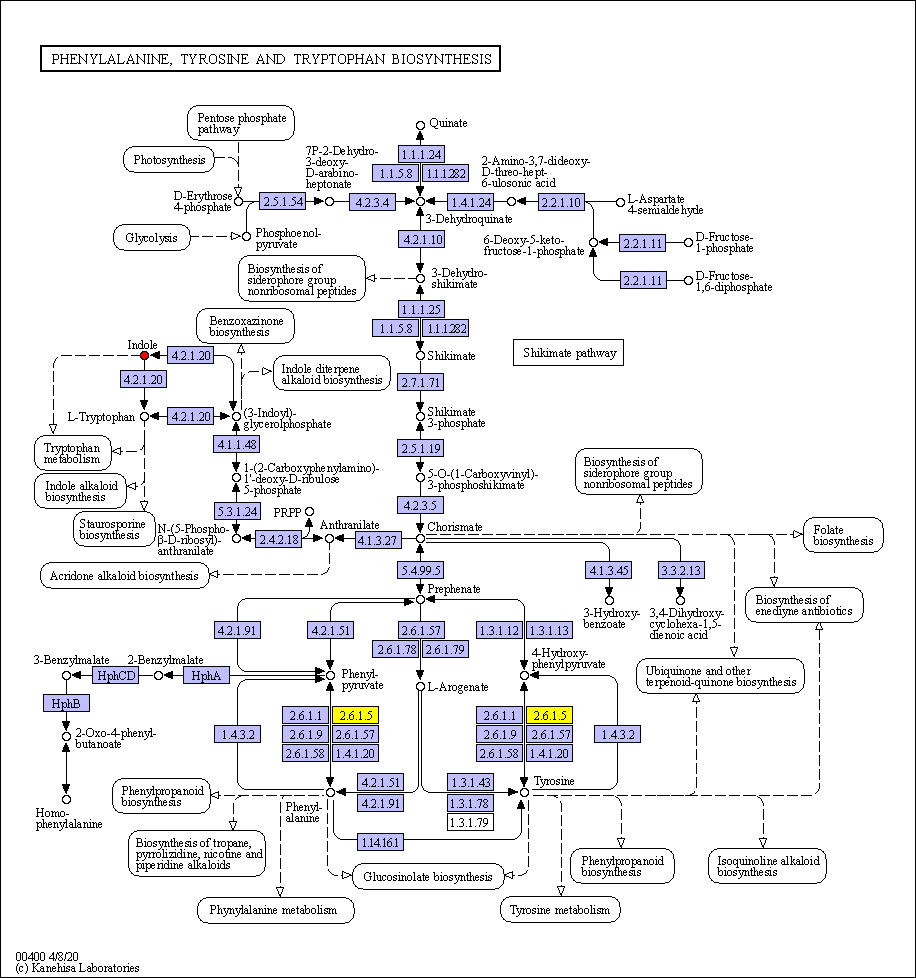


**Figure S6.** DEPs and DAMs involved in the phenylalanine, tyrosine and tryptophan biosynthesis pathways in the 2A-7×CC-2/Zheng58 comparison group. Red dots are upregulated metabolites, green dots are downregulated metabolites, pink rectangles are upregulated proteins, and yellow rectangles are downregulated proteins.

**Table S1** Primers for event-specific PCR analysis of transgenic maize.

| **Maize line** | **Primer** | **Sequence of primer (5’-3’)** | **Size of fragment (bp)** |
| --- | --- | --- | --- |
| 2A-7 | 2A-7 F | CGGTCGATGAACGTGAACAAG | 232 |
|  | 2A-7 R | CAGTACATTAAAAACGTCCGCAAT |  |
| CC-2 | CC-2 F | 5′- GTTTATGGTTCTCCCCGTGTA -3′ | 115 |
|  | CC-2 R | 5′- TCGGGGGATCTGGATTTTAGT -3′ |  |
| 2A-7×CC-2 | 2A-7 F | CGGTCGATGAACGTGAACAAG | 232 |
|  | 2A-7 R | CAGTACATTAAAAACGTCCGCAAT |  |
|  | CC-2 F | 5′- GTTTATGGTTCTCCCCGTGTA -3′ | 115 |
|  | CC-2 R | 5′- TCGGGGGATCTGGATTTTAGT -3′ |  |

**Table S2** Primers for qRT-PCR detection of co-DEPs used in KEGG analysis.

| Accession | Name | Primer | Sequence (5'-3') |
| --- | --- | --- | --- |
|  | GAPDH | GAPDH 1F | AACATCGGTATCAACGGCTTCG |
|  |  | GAPDH 1R: | TGGGTGGAGTCGTACTTGAGC |
| E9JVD4 | Aldose reductase | E9JVD4 1F | GCGGTCAGCGAATGTTCCAC |
|  |  | E9JVD4 1R | CCAGCGGAGAGTAAGCAGTAAC |
| Q0QWI2 | Sorbitol dehydrogenase | Q0QWI2 2F | CTGGCGGACGAGGTGGAG |
|  |  | Q0QWI2 2R | CATCCCGACCAGGCACACC |
| B6THG0 | Peroxidase | B6THG0 2F | CCCAAGGCGGAGTCCATCG |
|  |  | B6THG0 2R | GAAGCAGTCGTGGAAGTGGAG |
| B8A046 | Phenylalanine ammonia-lyase | B8A046 1F | CTCGCCATCGCCAACATCG |
|  |  | B8A046 1R | TGCCCTTGAAGCCGTAGTCC |
| A0A1D6HP35 | Glutathione reductase | A0A1D6HP35 1F | CGCTGCTGAAGAGTTCGTGAC |
|  |  | A0A1D6HP35 1R | AGTTCGTCTTTGGCTTGGATGTC |
| Q9SPJ8 | Cell wall invertase | Q9SPJ8 2F | AAGGACGGTGTGGCTGGAC |
|  |  | Q9SPJ8 2R | TTACCCTGTTCCTGAGAGTGACC |

**Table S3** List of all identified proteins in maize seeds.

| **Accession** | **Name** | **Protein Mass** | **Protein Length** |
| --- | --- | --- | --- |
| B6TGZ8 | Uncharacterized protein | 3616.1 | 32 |
| B6SM71 | Uncharacterized protein | 5404.2 | 46 |
| B8XVR5 | Ubiquitin-like protein ATG12 | 6121.1 | 54 |
| B6U454 | Uncharacterized protein | 6894 | 56 |
| B6UBC3 | Uncharacterized protein | 6255.5 | 56 |
| B7ZXJ4 | Uncharacterized protein | 6969.1 | 60 |
| B6SNR4 | Ubiquinol-cytochrome c reductase complex 6.7 kDa protein | 6661.6 | 61 |
| B6TXT3 | Uncharacterized protein | 6774.7 | 61 |
| C0HHN5 | Uncharacterized protein | 6979.4 | 61 |
| K7V2H9 | Uncharacterized protein | 7375.5 | 61 |
| B6T191 | Translation machinery associated TMA7 | 6717.8 | 62 |
| B6UDE4 | Uncharacterized protein | 6660.7 | 62 |
| B6UIM8 | 40S ribosomal protein S30 | 7056.3 | 62 |
| B6SPG4 | Ubiquinol-cytochrome c reductase complex 8.0 kDa protein | 7172.2 | 63 |
| B6SPL8 | Ubiquinol-cytochrome c reductase complex 8.0 kDa protein | 7180.2 | 63 |
| B6TH28 | Putative membrane lipoprotein | 6809.7 | 63 |
| B6U7Y4 | H/ACA ribonucleoprotein complex subunit 3-like protein | 7510.6 | 64 |
| B6UDY9 | H/ACA ribonucleoprotein complex subunit 3-like protein | 7455.6 | 64 |
| B6SJE6 | Flower-specific gamma-thionin | 7253.3 | 65 |
| B6SQB3 | Uncharacterized protein | 6213.2 | 65 |
| A0A1D6MSS7 | Uncharacterized protein | 6843.9 | 66 |
| B6SQE1 | COX VIIa-like protein | 7667 | 67 |
| B6TA09 | Uncharacterized protein | 7459.4 | 67 |
| B6TQ39 | Uncharacterized protein | 7503.6 | 67 |
| A0A1D6JPY7 | 40S ribosomal protein S28 | 7825.1 | 68 |
| B6UGT6 | Uncharacterized protein | 7944.1 | 68 |
| A0A1D6M294 | Inhibitor I family protein | 7568.6 | 69 |
| B6SGW4 | Uncharacterized protein | 7427.5 | 69 |
| B6SIV4 | 60S ribosomal protein L38 | 8037.4 | 69 |
| B6SMB0 | Cytochrome b-c1 complex subunit 6 | 8299.4 | 69 |
| B6SNL8 | 60S ribosomal protein L38 | 8062.5 | 69 |
| B6T9V8 | 60S ribosomal protein L38 | 8074.5 | 69 |
| B6TLS6 | Cytochrome b-c1 complex subunit 6 | 8331.5 | 69 |
| B6UE19 | 60S ribosomal protein L38 | 7965.4 | 69 |
| B6SLR8 | Subtilisin-chymotrypsin inhibitor CI-1C | 7804.8 | 70 |
| B6SNA6 | Subtilisin-chymotrypsin inhibitor CI-1B | 7504.6 | 70 |
| B6SGY0 | Subtilisin-chymotrypsin inhibitor CI-1B | 7561.6 | 71 |
| Q2XWZ4 | Protease inhibitor | 7518.6 | 71 |
| B4FET5 | Cytochrome b-c1 complex subunit 8 protein | 8468 | 72 |
| B6SQH6 | Import inner membrane translocase subunit Tim8 | 8234.6 | 72 |
| B6T320 | Uncharacterized protein | 8474.8 | 72 |
| B8QVS8 | Proteinase inhibitor | 7547.6 | 72 |
| K7TTS3 | Cox19-like CHCH family protein | 7901 | 72 |
| B6SGF5 | Uncharacterized protein | 7476.2 | 73 |
| B6T013 | Subtilisin-chymotrypsin inhibitor CI-1B | 7603.7 | 73 |
| B6U463 | Subtilisin-chymotrypsin inhibitor-2A | 7568.6 | 73 |
| B6SJA5 | Little protein 1 | 8108.8 | 74 |
| B7ZXS2 | Small nuclear ribonucleoprotein G | 8032.3 | 74 |
| B6TML5 | Mitochondrial import inner membrane translocase subunit Tim10 | 8796.2 | 75 |
| A0A317YIJ5 | Uncharacterized protein | 8678.4 | 76 |
| B6SP40 | Uncharacterized protein | 8356.5 | 76 |
| B6SIN8 | Metallothionein2 | 7705.5 | 77 |
| B6T232 | Uncharacterized protein | 8520.7 | 77 |
| B6TQW4 | Cytochrome c oxidase polypeptide Vc | 8612.9 | 77 |
| B6SHY0 | ATOZI1 | 8561.7 | 78 |
| B6SJS8 | Defensin-like protein 6 | 8387.8 | 78 |
| B4FKM7 | Uncharacterized protein | 8678.9 | 79 |
| B6SJ49 | Flower-specific gamma-thionin | 8870.4 | 79 |
| B6SJR3 | Mitochondrial import receptor subunit TOM7-1 | 8544 | 79 |
| B6SL97 | Defensin-like protein 1 | 8611.2 | 79 |
| A0A1D6EBT5 | DNA-binding protein S1FA | 8867.6 | 80 |
| B4FGA5 | Small nuclear ribonucleoprotein G | 8890.3 | 80 |
| B6SK31 | Small nuclear ribonucleoprotein G | 8934.4 | 80 |
| B6T1C3 | DNA-binding protein S1FA2 | 8851.5 | 80 |
| B6TET3 | Uncharacterized protein | 9026.5 | 80 |
| C0HIC4 | 40S ribosomal protein S21 | 8877.9 | 80 |
| B6SGF7 | Uncharacterized protein | 8580.4 | 81 |
| C0PL52 | Uncharacterized protein | 9123.5 | 81 |
| A6XER2 | Defensin-like protein 2 | 9057.5 | 82 |
| B6T6M4 | Uncharacterized protein | 8068.9 | 82 |
| B6SPL7 | Copper chaperone | 8738 | 84 |
| C5JA66 | Basal endosperm transfer layer10 | 9252.8 | 84 |
| A0A1D6NH20 | Seed maturation protein PM41 | 8689.2 | 85 |
| B6SMX5 | Low-molecular-weight cysteine-rich protein LCR70 | 9230.7 | 85 |
| K7VBL1 | Uncharacterized protein | 8893.8 | 85 |
| Q9ZQX9 | 40S ribosomal protein S27 | 9542.1 | 86 |
| A0A076YHT4 | Maternally expressed protein 9 (Fragment) | 9530 | 87 |
| B4FQ12 | Splicing factor subunit | 10117.2 | 87 |
| B4FYN0 | mitochondrial import inner membrane translocase subunit TIM13 | 9290.3 | 87 |
| B6T1H8 | B12D protein | 9773.3 | 87 |
| B6UDZ3 | Bowman-Birk type wound-induced proteinase inhibitor WIP1 | 9311.9 | 87 |
| A0A1D6N294 | Uncharacterized protein | 9376.7 | 88 |
| B6UE21 | Cyclin-dependent kinases regulatory subunit | 10408.9 | 88 |
| B6T113 | Bowman-Birk type wound-induced proteinase inhibitor WIP1 | 9498.1 | 89 |
| B6T2T4 | Nonspecific lipid-transfer protein | 8878.1 | 89 |
| B6UB43 | Acyl-CoA-binding protein | 10153.2 | 89 |
| B4FHU9 | Succinate dehydrogenase subunit 7A mitochondrial | 9843.4 | 90 |
| B6TM38 | Uncharacterized protein | 9889.4 | 90 |
| K7UD42 | Uncharacterized protein | 9708.2 | 90 |
| B6SGU6 | Uncharacterized protein | 9678.1 | 91 |
| B6SI37 | Embryo specific protein1 | 9669.5 | 91 |
| B6SNV1 | Uncharacterized protein | 9708.2 | 91 |
| B6SQD4 | Catalytic/ oxidoreductase, acting on NADH or NADPH | 10127.5 | 91 |
| B6SRG5 | Acyl-CoA-binding protein | 9965.2 | 91 |
| B6SYN4 | Uncharacterized protein | 9735.2 | 91 |
| B6TLX2 | Fiber protein Fb15 | 10654.2 | 91 |
| B6TTL1 | Ubiquitin-like protein ATG12 | 10239.7 | 91 |
| P46517 | Late embryogenesis abundant protein EMB564 | 9683.5 | 91 |
| A0A1D6LVQ2 | HVA22-like protein | 11078.9 | 92 |
| A0A1D6QJZ6 | Plant UBX domain-containing protein 11 | 10354 | 92 |
| B6SP75 | Uncharacterized protein | 9549.5 | 92 |
| B6TIA2 | 60S ribosomal protein L37a | 10257 | 92 |
| B6U092 | Uncharacterized protein | 9333.2 | 92 |
| B6UH62 | NudC domain-containing protein 2 | 10539.7 | 92 |
| K7V4C9 | Actin-related protein 2/3 complex subunit 5 | 10439.9 | 92 |
| B4FU14 | mitochondrial import inner membrane translocase subunit Tim9 | 10866.4 | 93 |
| B6SI77 | F1N21.17 | 11033.3 | 93 |
| B6SYW6 | Uncharacterized protein | 9794.3 | 93 |
| B6U5W6 | Mitochondrial import inner membrane translocase subunit Tim9 | 10792.3 | 93 |
| K7UVP7 | Cysteine and histidine-rich domain-containing protein RAR1 | 10675 | 93 |
| B4FXV1 | Uncharacterized protein | 9942.2 | 94 |
| B6SXG6 | Putative carbohydrate esterase | 10592.5 | 94 |
| B6T3V3 | Mitochondrial import receptor subunit TOM22 | 9765.2 | 94 |
| B6TGV6 | Nonspecific lipid-transfer protein | 9597.1 | 94 |
| B6UB58 | Ubiquitin-like protein | 10720 | 94 |
| K7VA33 | Uncharacterized protein | 10597.5 | 94 |
| B4FCE3 | Sm-like protein LSM6A | 10076.4 | 95 |
| B4FMW5 | 60S ribosomal protein L37a-2 | 10599.5 | 95 |
| B6SIB6 | BolA-like protein | 10012.3 | 95 |
| B6U2X6 | Mitochondrial import receptor subunit TOM22 | 9852.3 | 95 |
| B6UI15 | Subtilisin-chymotrypsin inhibitor CI-1B | 10279.9 | 95 |
| Q6ST18 | Heat shock factor binding protein 2 | 10247.3 | 95 |
| Q9ZRF5 | Acidic ribosomal protein P2a-4 (Fragment) | 9617.5 | 95 |
| A0A1D6H9Y7 | ATPase inhibitor | 10674.9 | 96 |
| B4G0X9 | Uncharacterized protein | 10317.7 | 96 |
| B6SHU7 | Uncharacterized protein | 10359.6 | 96 |
| B6SIK6 | Putative signal peptidase complex subunit 1 | 10453.3 | 96 |
| B6SJA3 | Signal peptidase complex subunit 1 | 10565.4 | 96 |
| B6TZN5 | Uncharacterized protein | 10596 | 96 |
| C4J7U8 | Uncharacterized protein | 10216.9 | 96 |
| A0A1D6E708 | Enhancer of rudimentary-like protein | 11415 | 97 |
| A0A1D6NGW8 | Vacuolar protein sorting protein 25 | 10663.1 | 97 |
| B4FNN2 | Early nodulin 93 | 10214.8 | 97 |
| B6SGF4 | Nonspecific lipid-transfer protein | 9894.6 | 97 |
| B6TAA7 | Chaperonin | 10577.2 | 97 |
| B6TUI2 | ATPase inhibitor | 10676.9 | 97 |
| B4FE30 | 10 kDa chaperonin | 10543.3 | 98 |
| B4FPJ2 | U6 snRNA-associated Sm-like protein LSm3 | 11235.8 | 98 |
| B6SLX1 | Chaperonin | 10507.2 | 98 |
| B6SP11 | Non-specific lipid-transfer protein 2 | 9809.3 | 98 |
| B6SXA8 | Ubiquitin-fold modifier 1 | 10139.7 | 98 |
| B6U7P4 | Lipid transfer protein1 | 10021.7 | 98 |
| B6UE79 | Ubiquitin-fold modifier 1 | 10165.7 | 98 |
| B6UH23 | B12D protein | 11044.6 | 98 |
| B8A1K8 | Ferredoxin | 10484.4 | 98 |
| A0A1D6F1Q5 | Uncharacterized protein | 11024.9 | 99 |
| A0A1D6F5A7 | Putative non-specific lipid-transfer protein 2 | 10302.9 | 99 |
| B4FNC9 | Small ubiquitin-related modifier | 10931.1 | 99 |
| B4FNR5 | U6 snRNA-associated Sm-like protein LSm8 | 10889.3 | 99 |
| B6T147 | 40S ribosomal protein S29 | 11284 | 99 |
| B6TLX0 | NADH-ubiquinone oxidoreductase 10.5 kDa subunit | 11101.7 | 99 |
| A0A1D6HE40 | Replication protein A 32 kDa subunit A | 10740 | 100 |
| A0A1D6KYH1 | Gibberellin-regulated protein 1 | 10601.4 | 100 |
| A3FMA4 | Bowman-Birk type trypsin inhibitor | 10406 | 100 |
| B6T157 | Uncharacterized protein | 11067 | 100 |
| B6T406 | Gibberellin-regulated protein 1 | 10591.4 | 100 |
| B6UII5 | Nonspecific lipid-transfer protein AKCS9 | 10159.9 | 100 |
| C0PET1 | Uncharacterized protein | 11985 | 101 |
| A0A1D6IMH3 | Retrovirus-related Pol polyprotein LINE-1 | 11180.6 | 102 |
| B4FHI8 | Hypoxia-responsive family protein | 11122.5 | 102 |
| B6TBN5 | 60S acidic ribosomal protein P1 | 10160.2 | 102 |
| Q93V45 | Wound-induced protease inhibitor | 10985.8 | 102 |
| Q946H3 | Wound-induced protease inhibitor | 10996.8 | 102 |
| A0A1D6JPY6 | 40S ribosomal protein S28 | 12069.2 | 103 |
| B6SRG1 | Uncharacterized protein | 11109.8 | 103 |
| B6TGI8 | Signal recognition particle 9 kDa protein | 12062.7 | 103 |
| B6UCK6 | Abscisic stress ripening protein 1 | 11673.9 | 103 |
| A0A1D6M725 | 60S ribosomal protein L30-2 | 11636.7 | 104 |
| B6SLR4 | Small nuclear ribonucleoprotein Sm D2 | 12019.1 | 104 |
| B6TF18 | Membrane steroid-binding protein 1 | 10996.2 | 104 |
| B6TJK3 | NADH dehydrogenase [ubiquinone] 1 beta subcomplex subunit 10-A | 12501.2 | 104 |
| B6UDZ9 | PVR3-like protein | 10605 | 104 |
| K7TM20 | Small nuclear ribonucleoprotein Sm D2 | 11933 | 104 |
| B4FFH9 | Small nuclear ribonucleoprotein Sm D2 | 12048.1 | 105 |
| B6SQE2 | CDGSH iron-sulfur domain-containing protein NEET | 10943.6 | 105 |
| K7VEL2 | Transcription elongation factor 1 homolog | 11758.6 | 105 |
| A0A1D6IKG5 | 40S ribosomal protein S9-2 | 11605.3 | 106 |
| B6SNY4 | Uncharacterized protein | 11390.2 | 106 |
| B6T666 | Transcription initiation factor IIA subunit 2 | 11852.5 | 106 |
| B6TLG0 | Uncharacterized protein | 12208.8 | 106 |
| B6TNG4 | Pollen-specific protein like | 11671.3 | 106 |
| B6UDB5 | Uncharacterized protein | 11606.6 | 106 |
| B6SHX0 | 5a2 protein | 11855.1 | 107 |
| B6SLM7 | Uncharacterized protein | 11013.1 | 107 |
| B6SNM4 | Protein transport protein Sec61 subunit beta | 10614 | 107 |
| B6ST85 | Glycine-rich cell wall structural protein | 11016.3 | 107 |
| B6TQE5 | Mitochondrial pyruvate carrier | 12072 | 107 |
| B6U636 | Catalytic/ oxidoreductase, acting on NADH or NADPH | 11482.9 | 107 |
| B6UFF4 | Pop3 peptide | 11458.2 | 107 |
| C5JA67 | BETL-9 protein | 11841 | 107 |
| A0A1D6L6Y7 | Uncharacterized protein | 11499.3 | 108 |
| A0A1D6LHK0 | Protein transport protein Sec61 subunit beta | 10673 | 108 |
| B4FG22 | 40S ribosomal protein S25-1 | 12022 | 108 |
| B4FLH6 | NADH dehydrogenase [ubiquinone] 1 beta subcomplex subunit 7 | 11960.9 | 108 |
| B4FXK8 | 40S ribosomal protein S25-1 | 12084 | 108 |
| B6UF37 | Uncharacterized protein | 12279.1 | 108 |
| B6UIG0 | Uncharacterized protein | 12039.1 | 108 |
| B6T361 | 60S acidic ribosomal protein P1 | 11041.3 | 109 |
| B8QW69 | Non-specific lipid-transfer protein (Fragment) | 10554.7 | 109 |
| P52855 | 60S acidic ribosomal protein P1 | 11096.4 | 109 |
| A0A1D6GL83 | Protease Do-like 7 | 12242.8 | 110 |
| B4FDM3 | DNA-directed RNA polymerase subunit | 12608.6 | 110 |
| B4FPE4 | V-type proton ATPase subunit G | 12234.6 | 110 |
| B4FVZ9 | V-type proton ATPase subunit G | 12307.7 | 110 |
| B4FWA7 | Mitochondrial pyruvate carrier | 12293 | 110 |
| B6T1H9 | Uncharacterized protein | 12692.9 | 110 |
| B6UD55 | NADH dehydrogenase [ubiquinone] 1 alpha subcomplex subunit 8-B | 12427.1 | 110 |
| B6UF11 | Ribosomal protein L37 | 12420.3 | 110 |
| B6UF59 | NADH dehydrogenase [ubiquinone] 1 alpha subcomplex subunit 8-B | 12441.2 | 110 |
| C0P568 | NADH dehydrogenase [ubiquinone] iron-sulfur protein 6 mitochondrial | 11714.5 | 110 |
| C0P8X9 | Uncharacterized protein | 12218.9 | 110 |
| C0PBC6 | NADH dehydrogenase [ubiquinone] 1 alpha subcomplex subunit 8-B | 12400.1 | 110 |
| Q4FZ53 | Cysteine proteinase inhibitor | 12311.9 | 110 |
| B4FFB8 | 5a2 protein | 12046 | 111 |
| B6SMY5 | Uncharacterized protein | 12413.1 | 111 |
| B6SZZ7 | Pi starvation-induced protein | 12028.4 | 111 |
| B4FNZ2 | Tubulin-specific chaperone A | 12509.1 | 112 |
| B4FYS2 | Cytochrome c-2 | 12210.9 | 112 |
| B6SKR4 | Cytochrome c | 12151.8 | 112 |
| B6ST82 | Uncharacterized protein | 11458.8 | 112 |
| B6TGS7 | Cytochrome c | 12209.9 | 112 |
| B6UGJ4 | Seed maturation protein | 11642.9 | 112 |
| C0P8D8 | Uncharacterized protein | 12307.8 | 112 |
| C0PK55 | Cytochrome c-2 | 12166.8 | 112 |
| C4IYY6 | 60S acidic ribosomal protein P2A | 11352.6 | 112 |
| Q5XLE1 | Peptidylprolyl isomerase | 12184.9 | 112 |
| Q9ATW9 | 60S ribosomal protein L33-B | 12885.9 | 112 |
| A0A1D6M1L7 | Uncharacterized protein | 13501 | 113 |
| B6T8E4 | Embryo specific protein5 | 12083.1 | 113 |
| A0A1D6HJU2 | Uncharacterized protein | 12871.3 | 114 |
| A0A1D6I6R9 | Uncharacterized protein | 11994 | 114 |
| B4FX04 | Dolichyl-diphosphooligosaccharide--protein glycosyltransferase subunit DAD1 | 12308.5 | 114 |
| B6SHX4 | NADH ubiquinone oxidoreductase B22-like subunit | 13353.8 | 114 |
| B6T1J9 | NADH ubiquinone oxidoreductase B22-like subunit | 13362.8 | 114 |
| B6T3H5 | DNA-directed RNA polymerase subunit | 13193.8 | 114 |
| B6UA28 | F8M12.18 protein | 12190.8 | 114 |
| B4FLV6 | Protein translation factor SUI1 homolog 2 | 12721.4 | 115 |
| B4FX16 | Macrophage migration inhibitory factor | 12162.8 | 115 |
| B6SIX5 | Protein translation factor SUI1 | 12764.5 | 115 |
| B6T5L1 | Complex I subunit | 12666.3 | 115 |
| B6TUZ6 | Macrophage migration inhibitory factor | 12008.6 | 115 |
| B6UHE2 | Defensin-like protein 6 | 12294 | 115 |
| Q19VG5 | GASA-like protein | 11912.9 | 115 |
| Q6LBH9 | Ferredoxin-thioredoxin reductase (Fragment) | 12957.6 | 115 |
| A0A317Y0Q6 | Mitochondrial import inner membrane translocase subunit PAM16 like 2 | 13142.9 | 116 |
| B6SID3 | Uncharacterized protein | 11845.2 | 116 |
| Q4FZ50 | Cysteine proteinase inhibitor | 12329.8 | 116 |
| B4F9G9 | Uncharacterized protein | 13680.4 | 117 |
| B4FHH3 | Uncharacterized protein | 13456.1 | 117 |
| B6U6I9 | Glycine-rich cell wall structural protein | 12201.7 | 117 |
| Q2XX15 | Non-specific lipid-transfer protein | 11348.7 | 117 |
| A0A1D6GHT6 | 40S ribosomal protein S27 | 13424.7 | 118 |
| A0A1D6HD72 | DNA-directed RNA polymerases II IV and V subunit 11 | 13837.6 | 118 |
| B6T5U0 | F1F0-ATPase inhibitor protein | 12532.1 | 118 |
| B6TGD4 | F1F0-ATPase inhibitor protein | 12386.8 | 118 |
| B6TLU5 | Lipid binding protein | 12148.4 | 118 |
| B6U9T1 | Uncharacterized protein | 12284.1 | 118 |
| Q45NP8 | Mannose-binding lectin | 13051.5 | 118 |
| A0A1D6F439 | Uveal autoantigen with coiled-coil domains and ankyrin repeats isoform 4 | 13334.6 | 119 |
| A0A1D6I126 | Uncharacterized protein | 12016.4 | 119 |
| B6U3J5 | Autophagy-related protein | 13822.8 | 119 |
| B6UGU1 | 60S ribosomal protein L34-3 | 13635 | 119 |
| Q2XX22 | Non-specific lipid-transfer protein | 11666.1 | 119 |
| A0A1D6FKZ1 | 60S acidic ribosomal protein P2A | 12272.7 | 120 |
| A0A317Y8M9 | Uncharacterized protein | 12926.5 | 120 |
| B4FJS1 | DNA-directed RNA polymerase II subunit J | 14120.8 | 120 |
| B4G1B3 | 60S acidic ribosomal protein P3 | 12134.2 | 120 |
| B6TBR8 | Seed specific protein Bn15D1B | 12216.6 | 120 |
| B6TYJ4 | Thioredoxin | 12975 | 120 |
| C0HJ97 | Eukaryotic translation initiation factor 5A | 13144.9 | 120 |
| O24413 | 60S acidic ribosomal protein P3 | 12219.3 | 120 |
| Q2XX14 | Non-specific lipid-transfer protein | 11751.2 | 120 |
| B6T089 | Non-specific lipid-transfer protein | 11740.2 | 121 |
| B6U278 | Uncharacterized protein | 12757.3 | 121 |
| B6U4A6 | Glycine-rich cell wall structural protein | 12505.9 | 121 |
| B8QW34 | Non-specific lipid-transfer protein | 11752.2 | 121 |
| C4J0R0 | Bet1-like SNARE 1-1 | 13733.3 | 121 |
| Q2XX16 | Non-specific lipid-transfer protein | 11806.2 | 121 |
| A0A096QRS2 | RING-box protein 1a | 13618.3 | 122 |
| A0A317YJL0 | Uncharacterized protein | 13840.9 | 122 |
| B4FJB5 | Hydrogen-transporting ATP synthase, rotational mechanism | 14042.2 | 122 |
| B6SIP2 | Hydrogen-transporting ATP synthase, rotational mechanism | 13959.1 | 122 |
| B6TQ98 | Peroxisomal multifunctional enzyme type 2 | 13222.3 | 122 |
| Q4W1F6 | Thioredoxin | 13039.1 | 122 |
| A0A1D6L8E2 | Cytochrome b-c1 complex subunit 7 | 14329.4 | 123 |
| B4FC89 | Uncharacterized protein | 12959.8 | 123 |
| B6SJJ1 | 60S ribosomal protein L31 | 14038.1 | 123 |
| B6SX73 | 60S ribosomal protein L35-1 | 14300.1 | 123 |
| B6TMP9 | Cytochrome b-c1 complex subunit 7 | 14481.6 | 123 |
| B6U1Q4 | COX VIIa-like protein | 13852.7 | 123 |
| C0P5V1 | RING-box protein 1a | 13751.4 | 123 |
| A0A1D6E7Y4 | Plant UBX domain-containing protein 1 | 14066.3 | 124 |
| B6SNJ5 | 60S ribosomal protein L31 | 14206.3 | 124 |
| B6TQC5 | Peptidyl-prolyl cis-trans isomerase | 13347.8 | 124 |
| B6UI14 | Lactoylglutathione lyase | 12987.7 | 124 |
| B6T2Z3 | Uncharacterized protein | 14053.7 | 125 |
| B6TTM6 | Uncharacterized protein | 13970 | 125 |
| B6SJN6 | Programmed cell death protein 5 | 14357 | 126 |
| B6T6T3 | 40S ribosomal protein S20 | 13872.1 | 126 |
| B6TEX6 | Cytochrome b-c1 complex subunit 7 | 14684.9 | 126 |
| B6TU95 | Uncharacterized protein | 13690 | 126 |
| B6U6V0 | Uncharacterized protein | 13858.6 | 126 |
| I2EBS8 | Thioredoxin | 13609.4 | 126 |
| K7VGJ2 | Ubiquitin carrier protein 7 | 13732.4 | 126 |
| K7WCU4 | Uncharacterized protein | 14080.7 | 126 |
| Q9AR47 | Prefoldin subunit 4 | 14689.2 | 126 |
| A0A1D6JXE5 | LIM domain-containing protein WLIM2b | 14455.6 | 127 |
| B6SIP0 | NHP2-like protein 1 | 13759.9 | 127 |
| B6TE40 | 60S ribosomal protein L22-2 | 14044.2 | 127 |
| B6TEM6 | Acyl carrier protein | 13698.5 | 127 |
| B6THW6 | Huntingtin interacting protein K | 13413.9 | 127 |
| Q4FZ46 | Cysteine proteinase inhibitor | 13661.7 | 127 |
| A0A1D6HXQ1 | Adenylate kinase isoenzyme 6-like protein | 13993.5 | 128 |
| B4FDT0 | 40S ribosomal protein S20-1 | 14002.4 | 128 |
| B4FI70 | Dynein light chain | 14295.1 | 128 |
| B6SRH2 | GIR1 | 13519.9 | 128 |
| Q9SWH6 | Uncharacterized protein (Fragment) | 14052.7 | 128 |
| A0A1D6G673 | Small ubiquitin-related modifier 2 | 14459.4 | 129 |
| A0A1D6LDG1 | Seed maturation protein | 13510.8 | 129 |
| B4FAP8 | B-cell receptor-associated 31-like | 14789.6 | 129 |
| B4FKE8 | Pi starvation-induced protein | 13914.7 | 129 |
| B6SIP7 | Prefoldin subunit 1 | 14841.7 | 129 |
| B6SJ07 | Non-specific lipid-transfer protein | 12294.9 | 129 |
| B6SJF7 | 14 kDa zinc-binding protein | 14229.3 | 129 |
| B6T4Y5 | Prefoldin subunit 1 | 14889.8 | 129 |
| B6THR4 | Thioredoxin-like 4 | 14353.2 | 129 |
| Q4FZ49 | Cysteine proteinase inhibitor | 13895.9 | 129 |
| A0A1D6GPD5 | Uncharacterized protein | 14314.9 | 130 |
| A0A1D6NY32 | Uncharacterized protein | 13730.4 | 130 |
| B4FN06 | Double-stranded DNA-binding family protein | 14648.4 | 130 |
| B6SJ13 | 40S ribosomal protein S15a-1 | 14790.2 | 130 |
| B6SYD0 | 60S ribosomal protein L22-2 | 14530.7 | 130 |
| B6TEL5 | V-type proton ATPase subunit F | 14473.4 | 130 |
| B6TUK9 | Basic blue protein | 13402.3 | 130 |
| B6UBZ4 | Uncharacterized protein | 14455.2 | 130 |
| A0A317YEE2 | Acyl carrier protein | 13998.9 | 131 |
| B4FK69 | Acyl carrier protein | 14440.2 | 131 |
| B4FLZ5 | Multifunctional methyltransferase subunit TRM112-like protein | 14510.5 | 131 |
| B6SK60 | Uncharacterized protein | 14542.4 | 131 |
| B6T5S0 | Prefoldin subunit 6 | 14808 | 131 |
| B6T7X9 | Profilin | 14073 | 131 |
| B6THA1 | Glutaredoxin homolog1 | 13689.5 | 131 |
| B6U8D6 | Uncharacterized protein | 14538.5 | 131 |
| B6UCI7 | Uncharacterized protein | 14137 | 131 |
| C0PJM7 | Signal recognition particle 14 kDa protein | 14834.2 | 131 |
| Q9FR39 | Profilin-5 | 14114.1 | 131 |
| B4FHZ4 | NADH dehydrogenase [ubiquinone] 1 alpha subcomplex subunit 6 | 15141.2 | 132 |
| B4FKA5 | Small nuclear ribonucleoprotein Sm D3 | 14340.7 | 132 |
| B4FNH4 | Small nuclear ribonucleoprotein Sm D3 | 14280.6 | 132 |
| B6SJY6 | HVA22-like protein | 14938.2 | 132 |
| B6SN61 | Grx_C2.1-glutaredoxin subgroup I | 13932 | 132 |
| B6TQT0 | 60S ribosomal protein L18A | 13962.9 | 132 |
| B6TSH8 | Chemocyanin | 13645.5 | 132 |
| B6TVB9 | Erwinia induced protein 2 | 15224 | 132 |
| C0P5A7 | Growth-regulating-factor-inteacting factor2 | 14013.5 | 132 |
| C4JBF9 | Prefoldin 6 | 14895.1 | 132 |
| A0A317YDY0 | Uncharacterized protein | 14662.5 | 133 |
| B6SX67 | 60S ribosomal protein L32 | 15785.6 | 133 |
| B6T1H7 | Glutaredoxin-like protein | 14715.1 | 133 |
| B6T5B3 | Uncharacterized protein | 15009.3 | 133 |
| B6T7Z2 | Cytochrome B5 isoform D | 14674.6 | 133 |
| B6U998 | 60S ribosomal protein L22-2 | 15123.2 | 133 |
| C0HGE1 | Extensin-like protein | 13430.7 | 133 |
| Q8H6X5 | Thioredoxin | 14638.5 | 133 |
| A0A0A1PKK5 | Cysteine proteinase inhibitor | 14782.9 | 134 |
| B4FBN1 | Chloroplast chaperonin 10 | 14429.4 | 134 |
| B4FFV2 | SWIB/MDM2 domain superfamily protein | 14005.2 | 134 |
| B4FUA9 | 60 ribosomal protein L14 | 15361.1 | 134 |
| B6T375 | Thioredoxin | 14732.8 | 134 |
| B6TMP6 | 60 ribosomal protein L14 | 15376.1 | 134 |
| B6UAH3 | Uncharacterized protein | 13902.9 | 134 |
| B6UGU7 | VQ motif family protein | 13786.4 | 134 |
| C0HHA0 | Uncharacterized protein | 14881.9 | 134 |
| Q30KV7 | Cysteine proteinase inhibitor | 14852 | 134 |
| Q30KW0 | Cysteine proteinase inhibitor | 14882 | 134 |
| W5RZV8 | Cysteine proteinase inhibitor | 14791.9 | 134 |
| A0A317YB59 | 7,8-dihydroneopterin aldolase | 14645.6 | 135 |
| B4FAE4 | CHL-CPN10 | 14435.5 | 135 |
| B4FIA6 | Histone H2A | 13902.9 | 135 |
| B4FKH7 | Cytochrome B5 isoform D | 14860.8 | 135 |
| B4FQM4 | SNARE-like superfamily protein | 15436.6 | 135 |
| B4G237 | Histone H2A | 14044 | 135 |
| B6T2C6 | Peptide-methionine (R)-S-oxide reductase | 14652.3 | 135 |
| B6T828 | Grx_C4-glutaredoxin subgroup I | 14569.6 | 135 |
| Q4A1K0 | Cysteine proteinase inhibitor | 14882 | 135 |
| A0A1D6P5N1 | Thioredoxin | 14877.1 | 136 |
| B6T3F2 | Histone H2A | 14415.6 | 136 |
| B6UGZ5 | Uncharacterized protein | 13397 | 136 |
| C0PFG6 | Uncharacterized protein | 14326.1 | 136 |
| K7TYE5 | Histone H2A | 14147.2 | 136 |
| A0A1D6ISX9 | Non-specific lipid-transfer protein | 14060 | 137 |
| B4FEE3 | 60S ribosomal protein L27 | 15636.5 | 137 |
| B4FJ27 | 40S ribosomal protein S24 | 15733.5 | 137 |
| B4FTA3 | 60S ribosomal protein L27 | 15606.5 | 137 |
| B6SIX3 | 40S ribosomal protein S24 | 15733.5 | 137 |
| B6T1R4 | Nucleic acid-binding OB-fold-like protein | 15136.2 | 137 |
| B6TW21 | Uncharacterized protein | 15420.3 | 137 |
| B6TZE4 | Microsomal glutathione S-transferase 3 | 15551.3 | 137 |
| C0PNT2 | 40S ribosomal protein S24 | 15657.4 | 137 |
| A0A1D6KL85 | HVA22-like protein | 15638 | 138 |
| B4FJ69 | Uncharacterized protein | 15643.6 | 138 |
| B6SL93 | Early response to dehydration 15-like protein | 15289 | 138 |
| B6T6D1 | Uncharacterized protein | 15193.3 | 138 |
| B6U4S6 | Thioredoxin | 15600.1 | 138 |
| B8A1M2 | Uncharacterized protein | 15072.9 | 138 |
| C0PFJ9 | Seven transmembrane domain protein | 15776.7 | 138 |
| K7UNW7 | Embryonic protein DC-8 | 14164.5 | 138 |
| P93631 | HMG transcription factor | 15007.2 | 138 |
| A0A1D6IV85 | Glycine-rich protein2 | 14658.6 | 139 |
| A0A1D6J1A0 | Non-specific lipid-transfer protein | 14279.2 | 139 |
| A0A1D6KQP9 | Cytochrome b5 | 15810 | 139 |
| A0A1D6PYT7 | Ribosomal protein S25 | 15713.4 | 139 |
| A0A317YJF2 | Uncharacterized protein YsfE | 15024.7 | 139 |
| B4FEJ6 | Actin depolymerizing factor3 | 15899.8 | 139 |
| B4FSW2 | Actin-depolymerizing factor 10 | 15913.8 | 139 |
| B6SGF3 | Glyoxalase family protein superfamily | 15082.7 | 139 |
| B6SM43 | Cytochrome b5 | 14845.8 | 139 |
| B6T175 | Alba DNA/RNA-binding protein | 15029.1 | 139 |
| C4JC68 | Uncharacterized protein | 14699.7 | 139 |
| B4FDG2 | Acyl carrier protein | 14986.9 | 140 |
| B4FGL4 | 60S ribosomal protein L23 | 15030.7 | 140 |
| B4FRN9 | 60S ribosomal protein L23 | 15060.8 | 140 |
| B6SHY8 | 40S ribosomal protein S12 | 14994.3 | 140 |
| B6TCB8 | 40S ribosomal protein S12 | 14993.3 | 140 |
| B6TW30 | Acyl carrier protein | 14981.9 | 140 |
| K7TFT9 | Acyl carrier protein | 15056 | 140 |
| A0A317Y8M6 | Non-specific lipid transfer protein-like 1 | 13799.7 | 141 |
| A0A1D6L7K8 | PITH domain-containing protein | 16301.4 | 142 |
| A0A1D6M5V0 | 60S ribosomal protein L36 | 16074.5 | 142 |
| A0A1D6M723 | 60S ribosomal protein L30-2 | 16075.9 | 142 |
| B4FGM4 | Endothelial differentiation-related factor 1 | 15647.9 | 142 |
| B4FID1 | 40S ribosomal protein S17-1 | 16458 | 142 |
| B4FJM6 | Lactoylglutathione lyase / glyoxalase I family protein | 15232.9 | 142 |
| B6SMH0 | Trypsin/factor XIIA inhibitor | 14917.2 | 142 |
| B6T4C6 | Mitosis protein dim1 | 16558.9 | 142 |
| B6T6W0 | 40S ribosomal protein S23 | 15718.4 | 142 |
| B6T8B3 | Endothelial differentiation-related factor 1 | 15678 | 142 |
| B6UE30 | Uncharacterized protein | 16048.1 | 142 |
| C0HEB4 | AP complex subunit sigma | 17051.7 | 142 |
| C4XVE1 | Translocon-associated protein subunit alpha | 15803 | 142 |
| K7VDJ2 | 40S ribosomal protein S17-4 | 16442.9 | 142 |
| Q9M7N5 | MFP1 attachment factor 1 | 14465.8 | 142 |
| A0A1D6P8A0 | Protein SPIRAL1 | 14310.9 | 143 |
| B4FQI3 | NADH dehydrogenase [ubiquinone] 1 alpha subcomplex subunit 13-A | 15973.4 | 143 |
| B4FTQ9 | AP complex subunit sigma | 16907.5 | 143 |
| B6T2N1 | SWIB/MDM2 domain superfamily protein | 15315.9 | 143 |
| B6T5N9 | Actin-depolymerizing factor 6 | 16636.6 | 143 |
| B6TMU3 | Uncharacterized protein | 16472.3 | 143 |
| B6UHZ7 | Uncharacterized protein | 15349.2 | 143 |
| C4IYX9 | Uncharacterized protein | 16134.5 | 143 |
| A0A1D6LFE5 | 40S ribosomal protein S29 | 15760.4 | 144 |
| A0A1D6LW48 | Elongin C | 16414.3 | 144 |
| B6TGW1 | Uncharacterized protein | 16052.2 | 144 |
| B6UHH1 | Uncharacterized protein | 15811.5 | 144 |
| C4J6C7 | Nucleic acid-binding OB-fold-like protein | 16345.3 | 144 |
| Q8LNY9 | His-containing phosphotransfer protein | 16109.3 | 144 |
| A0A1D6GGL6 | SWIb domain-containing protein | 15385.9 | 145 |
| A0A1D6JVL3 | B-cell receptor-associated 31-like | 16649.8 | 145 |
| B4F7Z7 | G10 family protein | 17360.8 | 145 |
| B4FD90 | 40S ribosomal protein S19 | 16265.6 | 145 |
| B4FTP0 | Actin-depolymerizing factor 6 | 16832.9 | 145 |
| B6SJ81 | Actin-depolymerizing factor 6 | 16834.9 | 145 |
| B6T0Z9 | 60S ribosomal protein L27a-3 | 16035.9 | 145 |
| B6T4H7 | 60S ribosomal protein L27a-3 | 16078 | 145 |
| B6T8X1 | G10 family protein | 17302.7 | 145 |
| B6TP17 | Enzyme of the cupin superfamily | 16042.2 | 145 |
| Q41802 | Defence-related protein | 15542.5 | 145 |
| A0A1D6ECX7 | 60S ribosomal protein L37a-2 | 16536.8 | 146 |
| B4FK58 | Signal recognition particle 19 kDa protein | 16059.6 | 146 |
| B6SHT0 | OB-fold nucleic acid binding domain containing protein | 15496.6 | 146 |
| B6SN23 | Negatively light-regulated protein | 15927.5 | 146 |
| B6SQA2 | 60S ribosomal protein L28 | 16352.9 | 146 |
| B6SRG8 | DNA-directed RNA polymerases I, II, and III 17.1 kDa polypeptide | 16554.6 | 146 |
| B6TB46 | Prefoldin subunit 2 | 16365.7 | 146 |
| B6TFZ9 | Amino acid selective channel protein | 15469.7 | 146 |
| C0PAS9 | Alba DNA/RNA-binding protein | 15872.8 | 146 |
| C4IYC2 | U6 snRNA-associated Sm-like protein LSm4 | 15627.7 | 146 |
| K7VQ98 | Class I heat shock protein 3 | 15846 | 146 |
| A0A1D6EPM1 | Carbon-sulfur lyases | 15902.6 | 147 |
| A0A1D6KMR1 | Histidine-containing phosphotransfer protein 1 | 16750.1 | 147 |
| B6T2D0 | Cytochrome b5 isoform 2 | 16177.2 | 147 |
| B6TX36 | Fasciclin-like arabinogalactan protein 8 | 14405 | 147 |
| A0A1D6JTE3 | Superoxide dismutase | 15501.3 | 148 |
| A0A1D6LXS9 | Signal recognition particle 9 kDa protein | 17057.5 | 148 |
| B4FC97 | Ubiquitin-conjugating enzyme E2 11 | 16486.9 | 148 |
| B4FFR3 | Outer envelope pore protein 16-3 chloroplastic/mitochondrial | 15617.8 | 148 |
| B4FNQ8 | Calmodulin | 16638.7 | 148 |
| B4FSG6 | Ubiquitin-conjugating enzyme E2 29 | 16510.9 | 148 |
| B6SK03 | Ubiquitin-conjugating enzyme E2 variant 1C | 16771 | 148 |
| B6T1J3 | 40S ribosomal protein S13 | 16868.8 | 148 |
| B6T3C4 | Ubiquitin-conjugating enzyme E2-17 kDa | 16410.7 | 148 |
| B6T4U8 | Calmodulin | 16555.5 | 148 |
| B6TG61 | 40S ribosomal protein S16 | 16749.6 | 148 |
| C0HI43 | DUF1279 family protein | 16034.5 | 148 |
| C0P3Z8 | Ubiquitin-conjugating enzyme E2 variant 1C | 16762.9 | 148 |
| C0P8C8 | Ferredoxin | 15491.1 | 148 |
| K7U7L9 | Putative vesicle-associated membrane protein family protein | 16586.7 | 148 |
| Q1A5Y5 | Phytochrome B1 (Fragment) | 15568.8 | 148 |
| Q6R9A4 | Ribosomal protein S7 | 17308.1 | 148 |
| A0A1D6FSD1 | 40S ribosomal protein S26 | 16644.1 | 149 |
| A0A317Y448 | Nucleoside diphosphate kinase 1 | 16540.8 | 149 |
| B6SNQ7 | 40S ribosomal protein S16-3 | 16891.8 | 149 |
| B6TVN0 | 40S ribosomal protein S16 | 16904 | 149 |
| B6UDP2 | Signal peptidase complex subunit 3 | 16879.3 | 149 |
| C0HHC4 | Nucleoside diphosphate kinase | 16830.3 | 149 |
| C4J6D2 | Uncharacterized protein | 14979 | 149 |
| Q43699 | Calmodulin | 16831.5 | 149 |
| A0A1D6ER29 | Copper transporter 5 | 16034.7 | 150 |
| A0A1D6P793 | Plasma membrane | 16932.1 | 150 |
| B4FDH5 | Histone H2B | 16173.8 | 150 |
| B6T522 | 40S ribosomal protein S14 | 16358.6 | 150 |
| B6TR40 | EARLY flowering 4 protein | 15304.8 | 150 |
| B6TVU2 | Prefoldin subunit 5 | 16387.8 | 150 |
| Q5EUD4 | Protein disulfide isomerase | 16835.3 | 150 |
| Q6JAD2 | Ferredoxin | 16028 | 150 |
| A0A1D6FPV2 | 40S ribosomal protein S12 | 16263.9 | 151 |
| A0A1D6LKT3 | Early nodulin-related | 16009.3 | 151 |
| A6YSM3 | PL3K2 | 16520.4 | 151 |
| B4FML8 | 40S ribosomal protein S13 | 17143.1 | 151 |
| B6UDR1 | Peptidyl-prolyl cis-trans isomerase | 15779.8 | 151 |
| A0A1D6E5Q7 | Kunitz trypsin inhibitor 1 | 15863.8 | 152 |
| A0A1D6IYD1 | Uncharacterized protein | 17155.4 | 152 |
| A0A1D6PR86 | Hevein-like preproprotein | 16008.9 | 152 |
| B4FIB4 | Histone deacetylase complex subunit SAP18 | 17128.7 | 152 |
| B4FTK1 | Ubiquitin-conjugating enzyme E2 2 | 17336.4 | 152 |
| B4FUS2 | 40S ribosomal protein S18 | 17694.5 | 152 |
| B4G286 | 40S ribosomal protein S18 | 17708.6 | 152 |
| B6SGR7 | Ubiquitin-conjugating enzyme E2 2 | 17339.4 | 152 |
| B6SIH3 | 16.9 kDa class I heat shock protein 1 | 17159.3 | 152 |
| B6SJC7 | Histone deacetylase complex subunit SAP18 | 17188.7 | 152 |
| B6SX57 | 60S ribosomal protein L23a | 16923 | 152 |
| B6T878 | Histone H2B | 16198.8 | 152 |
| B6TH74 | Histone H2A | 15909.4 | 152 |
| B6TJN9 | Histone deacetylase complex subunit SAP18 | 17234.8 | 152 |
| B6TRF5 | Win1 | 16131.4 | 152 |
| B6TTC8 | 16.9 kDa class I heat shock protein 1 | 17173.3 | 152 |
| B6UCM9 | Pleckstrin homology domain-containing protein 1 | 17210.3 | 152 |
| B6UF85 | ML domain protein | 16309.6 | 152 |
| B6UH99 | Late embryogeneis abundant protein Lea14-A | 16088.3 | 152 |
| C0P8H0 | Ferredoxin-thioredoxin reductase, catalytic chain | 16739.9 | 152 |
| K7TSG6 | Rhodanese-like domain-containing protein 19 mitochondrial | 16213.3 | 152 |
| P93801 | Superoxide dismutase [Cu-Zn] | 15088.6 | 152 |
| Q43701 | Heat shock protein 17.2 | 17162.2 | 152 |
| A0A1D6J436 | HR-like lesion-inducing protein-related | 17048.3 | 153 |
| A0A1D6LQK5 | Ubiquitin3 | 17058.6 | 153 |
| B4FM69 | 40S ribosomal protein S15a-1 | 17350.3 | 153 |
| B4FPT7 | Polyubiquitin 2 | 17101.5 | 153 |
| B6SIC6 | Putative ubiquitin-conjugating enzyme family | 17187.7 | 153 |
| B6T5E1 | Induced stolen tip protein TUB8 | 15281.4 | 153 |
| B6TM85 | Tankyrase 2 | 15814.5 | 153 |
| B6TUC4 | Glycine-rich RNA-binding protein 2 | 15655.3 | 153 |
| B6U085 | Endothelial differentiation-related factor 1 | 16209.6 | 153 |
| B6UBK0 | Nicotiana lesion-inducing like | 17396.6 | 153 |
| C4J8Y5 | Uncharacterized protein | 17648.3 | 153 |
| D2JSQ3 | ATP synthase subunit 8 | 17809.5 | 153 |
| E1U816 | ERTC | 16087.4 | 153 |
| B4FPA1 | Uncharacterized protein | 17336.8 | 154 |
| B4FPP4 | Protein AE7-like 1 | 17228.4 | 154 |
| B4FUJ3 | Uncharacterized protein | 16872.8 | 154 |
| B6SIX0 | 16.9 kDa class I heat shock protein 1 | 17058.1 | 154 |
| B6SJE9 | 17.0 kDa class II heat shock protein | 17046.6 | 154 |
| B6SNT9 | Thioesterase superfamily member 2 | 16254.6 | 154 |
| B6SQM0 | Major pollen allergen Car b 1 isoforms 1A and 1B | 16783.1 | 154 |
| B6TM82 | ML domain protein | 16752.2 | 154 |
| B6UFQ3 | Thioesterase superfamily member 2 | 16254.6 | 154 |
| B6UGR5 | 16.9 kDa class I heat shock protein 1 | 17102.2 | 154 |
| B8QVA0 | Hageman factor inhibitor | 16173.6 | 154 |
| Q19VG6 | Major latex protein 22 | 17111.3 | 154 |
| B4FUK2 | Peptidylprolyl isomerase | 16374.8 | 155 |
| B6SIL9 | 40S ribosomal protein S27a | 17697.6 | 155 |
| B6TGS0 | Uncharacterized protein | 16883.5 | 155 |
| B6TTU1 | Late embryogenesis abundant protein | 16784.7 | 155 |
| B6UHJ9 | 60S ribosomal protein L23a-1 | 17239.4 | 155 |
| C0HF09 | Copper-transporting atpase paa1 | 16153.1 | 155 |
| C0P2N0 | Heavy metal-associated isoprenylated plant protein 27 | 17230.9 | 155 |
| K7W249 | Cysteine proteinase inhibitor | 16042.1 | 155 |
| Q2XWV3 | Hageman factor inhibitor | 16273.6 | 155 |
| Q41753 | Ubiquitin fusion protein | 17731.5 | 155 |
| Q6LCT7 | Ubiquitin fusion protein | 17681.6 | 155 |
| Q8S2Y3 | Glycine-rich RNA binding protein (Fragment) | 15950.1 | 155 |
| A0A1D6FT90 | PLASMODESMATA CALLOSE-BINDING PROTEIN 2 | 16751 | 156 |
| B4FJ41 | Oleosin | 15793.1 | 156 |
| B6SGM7 | Uncharacterized protein | 15798.9 | 156 |
| B6SJD3 | Acylphosphatase | 17031.2 | 156 |
| B6SJU3 | Histone H2A | 16194.9 | 156 |
| B6T6E9 | LSM7-like | 16932.3 | 156 |
| B6TWV7 | Protein UXT | 18229.8 | 156 |
| B6U1V8 | Glycine-rich RNA-binding protein 2 | 15374.4 | 156 |
| B6U9L4 | ML domain protein | 16745.3 | 156 |
| C4J0J7 | Uncharacterized protein | 18259.8 | 156 |
| A0A1D6HI24 | 17.4 kDa class I heat shock protein 3 | 16686.6 | 157 |
| A0A1D6MLR1 | Ribosomal protein L26 | 17499.1 | 157 |
| A0A1D6Q4T8 | Protein RRP6-like 2 | 17262.3 | 157 |
| B4FA79 | Uncharacterized protein | 16431 | 157 |
| B6SHA8 | Histone H3 | 17296.9 | 157 |
| B6SRH1 | NADH-ubiquinone oxidoreductase 18 kDa subunit | 17251.6 | 157 |
| B6TD78 | 16.9 kDa class I heat shock protein 3 | 17663.7 | 157 |
| B6TPG2 | 60S ribosomal protein L26-1 | 17531.2 | 157 |
| B6TUB1 | NADH-ubiquinone oxidoreductase 18 kDa subunit | 17359.8 | 157 |
| B6UIA7 | Oleosin Bn-V | 16439.9 | 157 |
| Q5NKN3 | DNA-binding protein MNB1B | 17145.8 | 157 |
| Q8LK76 | Centromeric histone H3 | 17606.3 | 157 |
| A0A0F6P1Q3 | 17.7 kDa class I heat shock protein | 17745.7 | 158 |
| A0A1D6MDL1 | 40S ribosomal protein S12 | 17172.9 | 158 |
| A0A1D6Q6G4 | Expansin-B4 | 16193.3 | 158 |
| B4FFU5 | Uncharacterized protein | 16973.4 | 158 |
| B4FV52 | Uncharacterized protein | 17744.4 | 158 |
| B6SNS1 | NudC domain-containing protein 2 | 18128.3 | 158 |
| B6TBJ2 | Uncharacterized protein | 16885.8 | 158 |
| B6TGV8 | Uncharacterized protein | 16498.8 | 158 |
| B6TI93 | 40S ribosomal protein S23 | 17222.9 | 158 |
| C0PLX5 | Uncharacterized protein | 17815.8 | 158 |
| Q2XX74 | Pathogenesis-related protein 1 (Fragment) | 16655.2 | 158 |
| A0A1D6HBQ7 | Uncharacterized protein | 15343.7 | 159 |
| A0A1D6IZ49 | Seed maturation protein | 16123.6 | 159 |
| B4F976 | 17.4 kDa class I heat shock protein 3 | 17880 | 159 |
| B4FCV1 | NADH dehydrogenase [ubiquinone] 1 alpha subcomplex subunit 12 | 18534.8 | 159 |
| B4FUR6 | Glycine cleavage system H protein | 17325.2 | 159 |
| B6SHE9 | Insect intestinal mucin IIM22 | 16158 | 159 |
| B6SHN1 | Oleosin | 16914.2 | 159 |
| B6T2A5 | 40S ribosomal protein S11 | 17689.7 | 159 |
| B6T832 | Histone H2A | 16486.2 | 159 |
| B6T8H3 | 50S ribosomal protein L24 | 17704.6 | 159 |
| C4J240 | NADH dehydrogenase [ubiquinone] 1 alpha subcomplex subunit 12 | 18508.8 | 159 |
| B4FDE1 | Eukaryotic translation initiation factor 5A | 17442.4 | 160 |
| B6SIL7 | Eukaryotic translation initiation factor 5A | 17496.5 | 160 |
| B6TJF0 | Universal stress protein | 16911.3 | 160 |
| B6U7Z4 | Ubiquitin-conjugating enzyme E2 I | 18057.5 | 160 |
| C4JBL9 | Glycine cleavage system H protein | 17323.2 | 160 |
| Q29SB6 | Pathogenesis-related protein 10 | 16942.2 | 160 |
| A0A1D6LE54 | Succinate dehydrogenase subunit 6 mitochondrial | 17979.6 | 161 |
| A0A1D6NHF2 | Uncharacterized protein | 17255.5 | 161 |
| B4FKM4 | Protein mago nashi-like protein | 18506.7 | 161 |
| B4FNF4 | B-cell receptor-associated 31-like | 17744.7 | 161 |
| B6SQM6 | Pathogenesis-related protein 10 | 17026.3 | 161 |
| B6T4B0 | Uncharacterized protein | 17521.8 | 161 |
| B6T4J0 | Ubiquitin-conjugating enzyme E2 variant 1 | 18349.7 | 161 |
| B6TCL0 | Ferredoxin-thioredoxin reductase, variable chain | 17595.3 | 161 |
| B6THZ6 | Ubiquitin-conjugating enzyme E2 W | 18220.5 | 161 |
| A0A1D6HL18 | ER6 protein | 17189.9 | 162 |
| A0A317Y134 | Uncharacterized protein | 17977.2 | 162 |
| B4FN24 | Peroxiredoxin-2B | 17322.7 | 162 |
| B6T8B9 | ER6 protein | 17191.9 | 162 |
| B6TLK8 | 17.4 kDa class I heat shock protein 3 | 17943.8 | 162 |
| B6TR21 | Ferredoxin | 17381.8 | 162 |
| A0A1D6I7Y2 | Peptidylprolyl isomerase | 17448 | 163 |
| B4FS53 | Heat-and acid-stable phosphoprotein | 19028 | 163 |
| B4FT59 | 17.4 kDa class I heat shock protein | 18022 | 163 |
| B6SJI7 | Splicing factor, arginine/serine-rich 7 | 18680.5 | 163 |
| B6SJS7 | Nascent polypeptide-associated complex subunit beta | 17975.3 | 163 |
| B6SNE2 | Heat-and acid-stable phosphoprotein | 19034 | 163 |
| B6ST87 | Protein mago nashi | 18532.8 | 163 |
| B6T652 | Selenoprotein | 18448 | 163 |
| B6TBD4 | Nascent polypeptide-associated complex subunit beta | 17961.2 | 163 |
| B6TIB6 | GNAT transcription factor | 17816.2 | 163 |
| B6TR42 | Cold shock protein-1 | 15748.2 | 163 |
| A0A1D6KH07 | Uncharacterized protein | 17872.1 | 164 |
| A0A1D6NEI0 | Late embryogenesis abundant protein, group 3 | 16335.9 | 164 |
| B4FKK0 | Uncharacterized protein | 18313 | 164 |
| B4G087 | Adenine nucleotide alpha hydrolase-like superfamily protein | 17878.3 | 164 |
| B6T0Q2 | Histone H2A | 16994.9 | 164 |
| B6T326 | Peptidyl-prolyl cis-trans isomerase | 18012.4 | 164 |
| B6T3J3 | Histone H2A | 16996.9 | 164 |
| B6T766 | 60S ribosomal protein L21 | 18775.8 | 164 |
| B6TAN6 | Transcription regulator | 17353.2 | 164 |
| B6TYX3 | USP family protein | 17779.2 | 164 |
| C4J494 | 17.8 kDa class II heat shock protein | 17799.3 | 164 |
| C4J5F8 | 60S ribosomal protein L21-1 | 18848.9 | 164 |
| O24588 | Isocitrate lyase (Fragment) | 17552.7 | 164 |
| A0A1D6FPC0 | Acidic ribosomal protein P2b (Rpp2b) | 17508.7 | 165 |
| A0A317Y3U6 | Uncharacterized protein | 18907.5 | 165 |
| B4F9K4 | 17.5 kDa class II heat shock protein | 17868.5 | 165 |
| B4FFW5 | Adenine nucleotide alpha hydrolase-like superfamily protein | 17898.6 | 165 |
| B4FIE5 | Nascent polypeptide-associated complex subunit beta | 17767.9 | 165 |
| B4FJP1 | ATPase E1 | 18577.6 | 165 |
| B4FL49 | Nascent polypeptide-associated complex subunit beta | 17739.9 | 165 |
| B4FSM7 | Hemoglobin1 | 18278.2 | 165 |
| B6SNQ1 | Universal stress protein | 17438.9 | 165 |
| B6T9V0 | HSP20-like chaperones superfamily protein | 18642.7 | 165 |
| B6TVC7 | Ferredoxin | 17836.4 | 165 |
| B6TVI3 | 60S ribosomal protein L21 | 18849.8 | 165 |
| B6UHW9 | Oleosin Zm-II | 16771.3 | 165 |
| B8QX94 | Thaumatin-like protein (Fragment) | 16435.3 | 165 |
| C0HF03 | V-type proton ATPase proteolipid subunit | 16624.5 | 165 |
| C4J5G4 | Glycine-rich protein | 15920.3 | 165 |
| C4JAT0 | Uncharacterized protein | 17840.5 | 165 |
| K7V763 | Cytochrome c oxidase subunit 5b-2 mitochondrial | 17889.8 | 165 |
| A0A1D6G8D4 | Adenine nucleotide alpha hydrolase-like superfamily protein | 18833.7 | 166 |
| A0A1D6GJ80 | Ubiquitin-conjugating enzyme E2 variant 1A | 18928.3 | 166 |
| A0A1D6IIL4 | WPP domain-containing protein 2 | 16694 | 166 |
| A0A1R3MBN3 | Cyanate hydratase | 18526.1 | 166 |
| A0A1X7YF18 | Uncharacterized protein | 18120.7 | 166 |
| B4FA26 | AP complex subunit sigma | 19086 | 166 |
| B4FHT6 | Ran BP2/NZF zinc finger-like superfamily protein | 17804.2 | 166 |
| B4FRM7 | 60S ribosomal protein L12-3 | 17717.5 | 166 |
| B4FT80 | Nucleic acid-binding OB-fold-like protein | 18259.7 | 166 |
| B4G1C1 | Putative desiccation-related protein LEA14 | 17984.4 | 166 |
| B6T1H5 | 60S ribosomal protein L12-3 | 17768.5 | 166 |
| B6T6F6 | Ribosome biogenesis protein RLP24 | 19950.1 | 166 |
| B6T6G1 | 60S ribosomal protein L12 | 17701.4 | 166 |
| B6UF31 | Cytochrome c oxidase subunit | 18456.7 | 166 |
| A0A1D6LZ57 | USP family protein | 18037.5 | 167 |
| A0A1D6M5V2 | 60S ribosomal protein L36 | 19240 | 167 |
| A0A1D6NQJ6 | Uncharacterized protein | 16851.9 | 167 |
| B4FIR4 | Glutaredoxin-C5 chloroplastic | 17975.6 | 167 |
| B4FIS2 | Signal peptidase complex subunit 3 | 18880.5 | 167 |
| B6SIF5 | Translationally controlled tumor1 | 18742 | 167 |
| B6SIR7 | Uncharacterized protein | 17779.3 | 167 |
| B6T4Y4 | Grx_S12-glutaredoxin subgroup I | 18074.7 | 167 |
| B6T5R2 | Mitochondrial fission 1 protein | 18389.9 | 167 |
| B6T7L5 | THAP domain-containing protein 4 | 18126.4 | 167 |
| B6TP18 | 50S ribosomal protein L12-2 | 17127.9 | 167 |
| B6U3S1 | Uncharacterized protein | 16823.8 | 167 |
| C0P9S6 | Thioredoxin Y1 chloroplastic | 18536.3 | 167 |
| Q2XXD2 | Pathogenesis-related maize seed protein | 18447.8 | 167 |
| A0A1D6NAP0 | Os02g0306125-like protein | 18659.8 | 168 |
| A0A1D6NHF1 | Uncharacterized protein | 17975.3 | 168 |
| A3KLI0 | RAB17 protein | 17061.6 | 168 |
| A3KLI1 | Dehydrin DHN1 | 17075.6 | 168 |
| A7RDP0 | Lea2 | 17077.6 | 168 |
| B6SMX7 | 4-hydroxy-4-methyl-2-oxoglutarate aldolase | 17952.5 | 168 |
| B6SU31 | Glutathione peroxidase | 18457.7 | 168 |
| B6TN41 | 4-hydroxy-4-methyl-2-oxoglutarate aldolase | 18116.8 | 168 |
| P12950 | Dehydrin DHN1 | 17160.7 | 168 |
| Q6JAH6 | Glutathione peroxidase | 18459.8 | 168 |
| Q8LK64 | Glutathione peroxidase | 18619.9 | 168 |
| A0A1D6NG29 | Ubiquitin-conjugating enzyme E2 36 | 19102.9 | 169 |
| A0A1D6NZF7 | Uncharacterized protein | 18516.4 | 169 |
| A0A317YG84 | NADH dehydrogenase [ubiquinone] iron-sulfur protein 5-B | 19425.7 | 169 |
| B6T6A4 | Uncharacterized protein | 18247.7 | 169 |
| B6TDH3 | Protein MODIFIER OF SNC1 11 | 17533 | 169 |
| C4J8J2 | Protein TAPETUM DETERMINANT 1 | 17385.4 | 169 |
| K7TQ73 | Thioesterase family protein | 17438.6 | 169 |
| O22656 | Ubiquitin carrier protein 7 | 18970.2 | 169 |
| O24560 | Ubiquitin carrier protein 7 | 19151.4 | 169 |
| A0A1D6HKR8 | Putative RNA-binding protein ARP1 | 17892.8 | 170 |
| A0A1D6IU95 | DNA-directed RNA polymerases II IV and V subunit 10 | 19468.4 | 170 |
| B4FS99 | ATP synthase subunit d, mitochondrial | 19927.4 | 170 |
| B6SJ93 | Plasma membrane associated protein | 18074 | 170 |
| B6T7T9 | Uncharacterized protein | 18486.8 | 170 |
| B6TKU6 | 50S ribosomal protein L14 | 18307.5 | 170 |
| B6TRK0 | Mitochondrial fission 1 protein | 18834.2 | 170 |
| C0P5Z6 | DUF538 family protein | 18746.2 | 170 |
| A0A1D6GYU3 | Rho-related protein from plants 9 | 18685.4 | 171 |
| A0A1D6IB46 | 60S ribosomal protein L14-1 | 19304.5 | 171 |
| A0A1D6LPH1 | 40S ribosomal protein S14-3 | 18762.5 | 171 |
| A0A1D6QTH3 | Histone deacetylase complex subunit SAP18 | 19412.3 | 171 |
| A0A1Q0YGV1 | Ribosomal protein L17b | 19506.3 | 171 |
| A0A317YK16 | 60S ribosomal protein L17 | 19411.1 | 171 |
| B4F9E8 | 17.4 kDa class III heat shock protein | 18347.6 | 171 |
| B4FHE4 | 60S ribosomal protein L17-2 | 19392.1 | 171 |
| B6T1L4 | 60S ribosomal protein L17 | 19397.1 | 171 |
| B6T4K8 | Maternal effect embryo arrest 59 | 18506.2 | 171 |
| B6T8I3 | Dirigent protein | 17557.6 | 171 |
| B6TLW3 | Fiber protein Fb19 | 18098.6 | 171 |
| E2IPC6 | Chloroplast thioredoxin M-type 2 | 18374.3 | 171 |
| K7U3E1 | Histone H3.2 | 19085 | 171 |
| A0A1D6GIN0 | Plant/MUD21-2 protein | 17928.5 | 172 |
| A0A1D6HHV1 | Formate dehydrogenase chloroplastic/mitochondrial | 18955.5 | 172 |
| B4FY36 | Caltractin1 | 19772.9 | 172 |
| B4FZZ2 | Peptidyl-prolyl cis-trans isomerase | 18362 | 172 |
| B6SNN5 | MFT2-Corn MFT-like protein | 18922.6 | 172 |
| B6T381 | EF hand family protein | 19407.3 | 172 |
| B6TMH2 | Grx_S14-glutaredoxin subgroup II | 18669.5 | 172 |
| B6UIB1 | MFT2-Corn MFT-like protein | 18888.6 | 172 |
| C0PK59 | Uncharacterized protein | 19760.8 | 172 |
| C4JBV6 | Uncharacterized protein | 18270.5 | 172 |
| Q6R9F0 | Ribosomal protein S1 | 19482.9 | 172 |
| A0A1D6K0T8 | NADH ubiquinone oxidoreductase1 | 19382.9 | 173 |
| A0A1D6K371 | Nascent polypeptide-associated complex alpha subunit-like protein | 19111 | 173 |
| A8WES5 | Protein FLOWERING LOCUS T | 19312.9 | 173 |
| B6SIE4 | DUF538 family protein | 18320.8 | 173 |
| B6SJN4 | Uncharacterized protein | 18779.7 | 173 |
| B6SLS7 | Uncharacterized protein | 17647 | 173 |
| B6T319 | RS21-C6 protein | 19028.2 | 173 |
| B6T3Q3 | Adenine nucleotide alpha hydrolase-like superfamily protein | 18763.6 | 173 |
| B6TZR1 | NADH-ubiquinone oxidoreductase 13 kDa-B subunit | 19408.9 | 173 |
| C0PC75 | Thioredoxin | 18739.7 | 173 |
| A0A1D6GTY0 | Bifunctional inhibitor/lipid-transfer protein/seed storage 2S albumin superfamily protein | 19020.3 | 174 |
| A0A317Y7C5 | 40S ribosomal protein S4 | 20244.6 | 174 |
| B6TRK2 | Glucan endo-1,3-beta-glucosidase 3 | 17304.3 | 174 |
| B6TTP4 | Stress-inducible membrane pore protein | 17814.7 | 174 |
| B6U7P6 | Blue copper protein | 17691.9 | 174 |
| B6UH30 | PEBP (Phosphatidylethanolamine-binding protein) family protein | 18729 | 174 |
| C0P3Z2 | WPP domain-containing protein 2 | 17343.8 | 174 |
| C0PJ68 | 40S ribosomal protein S12 | 18759.9 | 174 |
| K7VC71 | Uncharacterized protein | 19378.1 | 174 |
| Q2XX96 | Pathogenesis-related protein 5 | 17504.4 | 174 |
| Q94F75 | GNAT transcription factor | 20289.3 | 174 |
| A0A1D6F696 | NADH-ubiquinone reductase complex 1 MLRQ subunit | 18766.3 | 175 |
| A0A1D6LDM1 | Protein mago nashi-like protein | 19718 | 175 |
| B6SJ01 | Uncharacterized protein | 18779.3 | 175 |
| B6SRE7 | Peptidyl-prolyl cis-trans isomerase | 18586 | 175 |
| B6T814 | Calcineurin subunit B | 20063.6 | 175 |
| B6TX84 | Calcineurin subunit B | 20063.6 | 175 |
| C0HHQ5 | Transmembrane protein 85 | 18894.7 | 175 |
| B4FLE3 | HSP20-like chaperones superfamily protein | 19824.6 | 176 |
| B4FST4 | SKP1-like protein 1A | 19254.3 | 176 |
| B4G233 | Dirigent protein | 18259.5 | 176 |
| B6SI42 | Oleosin | 17469.8 | 176 |
| B6TT37 | Blue copper protein | 17425.1 | 176 |
| B6UGN9 | Oleosin | 17425.7 | 176 |
| C0P6L6 | Dirigent protein | 18327.9 | 176 |
| C4J950 | Uncharacterized protein | 19551.2 | 176 |
| Q53AV6 | Putative zinc finger protein ZF2 | 18263.7 | 176 |
| Q9MAY9 | Coatomer subunit zeta-1 | 19458.1 | 176 |
| A0A1D6PZ79 | EMB2733/ESP3 (EMBRYO DEFECTIVE 2733) | 20060.2 | 177 |
| B6SHU1 | Vacuolar protein sorting protein 25 | 20623.5 | 177 |
| B6T8D0 | N-terminal acetyltransferase complex ARD1 subunit A | 19840.3 | 177 |
| B6T9S0 | Thioredoxin X chloroplastic | 19089.8 | 177 |
| C0P6C4 | Uncharacterized protein | 18918.6 | 177 |
| K7U3I3 | Ethylene response protein | 19928.6 | 177 |
| K7UZD3 | Pop3 peptide | 19674 | 177 |
| A0A1D6FSJ2 | 60S ribosomal protein L24 | 20840.5 | 178 |
| A0A1D6M656 | NF-YB-like protein | 18861.9 | 178 |
| A0A1D6MPT6 | U6 snRNA-associated Sm-like protein LSm4 | 19229.7 | 178 |
| B4FAB0 | Cupredoxin superfamily protein | 17447.1 | 178 |
| B4FU90 | 60S ribosomal protein L18a | 21378.9 | 178 |
| B6T1D8 | 60S ribosomal protein L18a | 21473 | 178 |
| B6T7X0 | Acyl-CoA N-acyltransferase (NAT) superfamily protein | 19668.5 | 178 |
| B6U3H8 | Lipid transfer protein | 16671.9 | 178 |
| B6UAV8 | 60S ribosomal protein L18a | 21380.9 | 178 |
| B6UFN0 | Outer envelope pore protein 16-2 chloroplastic | 18159.2 | 178 |
| Q00LN5 | Mucronate mutant 16 kDa gamma zein | 18542.3 | 178 |
| Q946W0 | 15kD beta zein | 19106.4 | 178 |
| Q9ZTT0 | 6-phosphogluconate dehydrogenase isoenzyme B (Fragment) | 18943.5 | 178 |
| A0A1D6HWL3 | Cytidine/deoxycytidylate deaminase family protein | 19213.6 | 179 |
| A0A1D6HXQ0 | Adenylate kinase isoenzyme 6 homolog | 19921.1 | 179 |
| B4FQQ1 | Reactive Intermediate Deaminase A chloroplastic | 18888.4 | 179 |
| B4FW06 | 40S ribosomal protein S10-1 | 19938.3 | 179 |
| B6SGY5 | RNA binding protein | 17660.9 | 179 |
| B6T8E7 | 40S ribosomal protein S10 | 19980.5 | 179 |
| B6TFE5 | Uncharacterized protein | 18707.4 | 179 |
| B6UAQ7 | Pectinesterase inhibitor domain containing protein | 18509.5 | 179 |
| B6UAW2 | Zinc-binding protein | 19137.7 | 179 |
| J7LC26 | Ribosomal protein S10 | 20060.5 | 179 |
| K7TU64 | Transmembrane protein 18 | 20109.1 | 179 |
| A0A1D6HA44 | Uncharacterized protein | 18702 | 180 |
| A8WES3 | ZCN11 | 19187.9 | 180 |
| B4FS03 | Adenine phosphoribosyltransferase 1 | 19348.4 | 180 |
| B4FVF9 | CCAAT-DR1 transcription factor | 19069.1 | 180 |
| B4FVQ8 | Adenine nucleotide alpha hydrolase-like superfamily protein | 19904.7 | 180 |
| B6T551 | Nudix hydrolase 13 | 20164.4 | 180 |
| B6T920 | Adenine phosphoribosyltransferase 1 | 19396.4 | 180 |
| B6TG00 | Copper ion binding protein | 17899.1 | 180 |
| B6TQX0 | Uncharacterized protein | 19226.8 | 180 |
| B6UCS0 | Signal peptidase I | 20088.6 | 180 |
| B8A1P4 | Signal peptidase I | 20159.7 | 180 |
| C0P3F6 | Thioesterase superfamily protein | 19164.6 | 180 |
| Q41888 | Prolamin PPROL 17 | 19417.7 | 180 |
| A0A1D6H4T8 | Uncharacterized protein | 19862.6 | 181 |
| A0A1D6HA45 | Uncharacterized protein | 18785.2 | 181 |
| B4FMJ0 | ADP-ribosylation factor 1 | 20579.5 | 181 |
| B4FP40 | ADP-ribosylation factor | 20665.5 | 181 |
| B6SJ67 | Lipid transfer protein | 18767.2 | 181 |
| B6SUK1 | Ligatin | 20329.5 | 181 |
| B6TQP9 | Lipid transfer protein | 18769.2 | 181 |
| B6UIH3 | Threonine endopeptidase | 19518 | 181 |
| C0PH28 | 40S ribosomal protein S10-1 | 20226.7 | 181 |
| K7V5D2 | Bundle sheath strands specific1 | 20428.9 | 181 |
| Q38JE3 | TIL-2-Zea mays Temperature-induced lipocalin-2 | 20904.5 | 181 |
| A0A1D6GMM7 | Cytochrome b5 isoform E | 20327.3 | 182 |
| A0A1D6HJB6 | Uncharacterized protein | 20005.7 | 182 |
| A0A1D6Q3A1 | NADPH-dependent pterin aldehyde reductase | 18848.4 | 182 |
| A0A317Y380 | Uncharacterized protein | 18770.5 | 182 |
| B4FJ97 | Uncharacterized protein | 20115.6 | 182 |
| B4FM64 | ADP-ribosylation factor 3 | 20356 | 182 |
| B4FRH1 | Thioredoxin M1 chloroplastic | 19560.4 | 182 |
| B4FV31 | 60S ribosomal protein L11-1 | 20897.1 | 182 |
| B6SID7 | Late embryogenesis abundant protein, group 3 | 18599.1 | 182 |
| B6T2I0 | 60S ribosomal protein L11-1 | 20791.9 | 182 |
| B6T8R3 | 40S ribosomal protein S10 | 20303.7 | 182 |
| B6TXN6 | 40S ribosomal protein S26 | 19940.7 | 182 |
| B6UH71 | Coatomer subunit zeta-1 | 19970.4 | 182 |
| B7ZY36 | Uncharacterized protein | 18709.2 | 182 |
| C0PAV3 | Ubiquitin-fold modifier-conjugating enzyme 1 | 20319.1 | 182 |
| K7TT54 | Uncharacterized protein | 19446.4 | 182 |
| A0A1D6J5D3 | S-adenosyl-L-methionine-dependent methyltransferase superfamily protein | 20135.8 | 183 |
| A0A1D6K5R3 | Protein SPIRAL1 | 18832.9 | 183 |
| A0A317Y3X3 | Lipid transfer protein EARLI 1 | 17432.8 | 183 |
| B4FHR1 | Uncharacterized protein | 20961.9 | 183 |
| B4FIK1 | Ubiquitin-conjugating enzyme E2 4 | 20871.3 | 183 |
| B4FVZ7 | AIG2-like protein | 20721.4 | 183 |
| B6TGL6 | Uncharacterized protein | 18491.1 | 183 |
| B6TJ90 | Multifunctional fusion protein | 20075 | 183 |
| B6TWE3 | Uncharacterized protein | 19129.7 | 183 |
| B6U5I8 | 2Fe-2S ferredoxin | 19965.3 | 183 |
| B6UD91 | Pectinesterase inhibitor domain containing protein | 18962.3 | 183 |
| B6UHH4 | NEDD8-conjugating enzyme Ubc12-like | 20658.5 | 183 |
| B6UIC1 | 50S ribosomal protein L12-1 | 18858.3 | 183 |
| C0PNT5 | Uncharacterized protein | 20644.5 | 183 |
| K7UV01 | HMG-Y-related protein A | 19083.6 | 183 |
| Q548E8 | 16 kDa gamma zein | 19558.5 | 183 |
| A0A096RZ13 | SnRK1-interacting protein 1 | 20247.8 | 184 |
| A0A1D6HYS3 | Phosphoglucosamine mutase family protein | 20192.2 | 184 |
| A0A1D6J7S7 | Uncharacterized protein | 19155.6 | 184 |
| A0A1D6JX12 | PYM protein | 20035.4 | 184 |
| A0A1D6MWV4 | Osmotin-like protein | 18681.2 | 184 |
| B6TDA1 | Splicing factor, arginine/serine-rich 7 | 20972.9 | 184 |
| B6TIS1 | SnRK1-interacting protein 1 | 20148.7 | 184 |
| C0P8F5 | Cyclic phosphodiesterase | 20759.2 | 184 |
| Q6PNA0 | Putative RUB1 conjugating enzyme | 20655.6 | 184 |
| A0A0D5BX12 | Methionine sulfoxide reductase | 20360.4 | 185 |
| A0A1D6NQP9 | Non-specific lipid-transfer protein | 19267.1 | 185 |
| B4FWF5 | Histone deacetylase 6 | 20101.2 | 185 |
| B4FZ61 | Trafficking protein particle complex subunit | 20745.5 | 185 |
| B6SIW3 | PME/invertase inhibitor-like protein | 19439.2 | 185 |
| B6T1X5 | Grx_S15.1-glutaredoxin subgroup II | 20004.5 | 185 |
| B6TQP7 | Lipid transfer protein | 18899.3 | 185 |
| B6TTI9 | Cytochrome b5 | 20491.1 | 185 |
| Q946V1 | Hageman factor inhibitor | 19626.5 | 185 |
| A0A317YF25 | Guanine deaminase | 20328 | 186 |
| A0A317YHF1 | Universal stress protein A-like protein | 19487 | 186 |
| A1YLY2 | Cytidine/deoxycytidylate deaminase family protein | 20264.9 | 186 |
| B4FPE0 | Cytidine/deoxycytidylate deaminase family protein | 20170.8 | 186 |
| B6SIZ2 | Oleosin | 18332.7 | 186 |
| B6T2W6 | RNA-binding protein cabeza | 19301.8 | 186 |
| B6T8Z7 | Cytidine/deoxycytidylate deaminase family protein | 20207.8 | 186 |
| B6UCA0 | HVA22-like protein | 21837.7 | 186 |
| C0PMQ1 | Lactoylglutathione lyase | 21060.6 | 186 |
| B4FR02 | PITH domain-containing protein | 20748.2 | 187 |
| B6SJ08 | 60S ribosomal protein L18-3 | 21042.6 | 187 |
| B6SNF5 | OLE-5 | 19420.2 | 187 |
| B6SSG7 | Dehydration responsive element binding protein | 19277.4 | 187 |
| B6T9W1 | Wound/stress protein | 19692.2 | 187 |
| B6TF86 | Hydroxyacylglutathione hydrolase 3 | 20246.8 | 187 |
| B6TPZ0 | Histone H3 | 20767.1 | 187 |
| B6U062 | Early nodulin 20 | 19390.6 | 187 |
| B6UHJ5 | Mal d 1-associated protein | 20735.5 | 187 |
| K7U5W7 | Wound/stress protein | 19606.1 | 187 |
| K7V2R4 | Lachrymatory factor synthase | 19445.5 | 187 |
| A0A1D6MX78 | Prefoldin subunit 3 | 20984.8 | 188 |
| A0A317YGV5 | 40S ribosomal protein S25-2 | 20930.1 | 188 |
| B4FKM9 | 60S ribosome subunit biogenesis protein NIP7 homolog | 20914.8 | 188 |
| B6SQ48 | Peripheral-type benzodiazepine receptor | 18376.1 | 188 |
| B6SQG1 | Nudix hydrolase 13 | 21014.3 | 188 |
| B6SUM0 | Vacuolar protein sorting-associated protein 29 | 20880 | 188 |
| B6T8W5 | Bifunctional coenzyme A synthase | 20186.7 | 188 |
| C0PK92 | Abscisic acid receptor PYL2 | 20560 | 188 |
| K7VZ76 | DUF679 domain membrane protein 2 | 19920.7 | 188 |
| A0A1D6J8F5 | Small nuclear ribonucleoprotein Sm D1 | 20612.8 | 189 |
| A0A1D6JFA5 | Acyl-CoA N-acyltransferase (NAT) superfamily protein | 21580.5 | 189 |
| A0A1D6JK23 | Extensin-like protein | 16781.8 | 189 |
| A0A1D6MBF3 | 60S ribosomal protein L28-1 | 21419.8 | 189 |
| A0A317Y0U8 | Uncharacterized protein | 20179.8 | 189 |
| B6T277 | Autophagy-related protein 101 | 22480.6 | 189 |
| B6TH32 | Uncharacterized protein | 20767.4 | 189 |
| B6TKE7 | Prefoldin subunit 3 | 20963.7 | 189 |
| B7ZZ71 | Cobalt ion binding | 20473.9 | 189 |
| C0PD54 | Molybdopterin synthase catalytic subunit | 20531.8 | 189 |
| A0A1D6F932 | 40S ribosomal protein S15-4 | 21230.7 | 190 |
| A0A1D6KMJ3 | Late embryogenesis abundant (LEA) hydroxyproline-rich glycoprotein family | 20459.1 | 190 |
| B6SI39 | 60S ribosomal protein L9-1 | 21460.8 | 190 |
| B6T7Z4 | 60S ribosomal protein L9 | 21460.8 | 190 |
| B6TGJ2 | Translocon-associated protein beta containing protein | 20591.5 | 190 |
| B6TH42 | 60S ribosomal protein L9-1 | 21373.8 | 190 |
| C4JBF5 | 60S ribosomal protein L9-1 | 21505 | 190 |
| Q5K097 | NADH dehydrogenase, subunit 9 | 22930.8 | 190 |
| A0A1D6DW25 | Ripening-related protein 3 | 20219.2 | 191 |
| B4FHX9 | Uncharacterized protein | 20950.6 | 191 |
| B6SN99 | Translocon-associated protein beta containing protein | 20703.7 | 191 |
| B6UHB1 | Uncharacterized protein | 21121.7 | 191 |
| C0HIB6 | Histone H2A | 20265.6 | 191 |
| Q3S3T0 | Hemoglobin 2 | 20646.1 | 191 |
| A0A1D6JMQ2 | Uncharacterized protein | 21037.1 | 192 |
| A0A317Y9G6 | Phospholipase A2 3 | 20842 | 192 |
| B4FTE1 | Signal peptidase complex subunit 2 | 21178.4 | 192 |
| B6SHM7 | 40S ribosomal protein S7 | 22211.7 | 192 |
| B6T3U2 | CUE domain containing protein | 20876.5 | 192 |
| B6TAA9 | Uncharacterized protein | 20917.5 | 192 |
| B6TDH2 | Uncharacterized protein | 21303.1 | 192 |
| B6TE31 | 40S ribosomal protein S7 | 22120.5 | 192 |
| B6TLK6 | HVA22-like protein | 22300.3 | 192 |
| B6TMC3 | PRA1 family protein | 20837.1 | 192 |
| B6U9B7 | Lipid transfer protein | 19164.7 | 192 |
| C0HGK7 | Uncharacterized protein | 21313.4 | 192 |
| C0P6I6 | Uncharacterized protein | 20389.4 | 192 |
| C4J122 | HVA22-like protein | 22511.4 | 192 |
| A0A1R3M7Z7 | HMG-Y-related protein A | 19824.4 | 193 |
| B4G1F2 | 39S ribosomal protein L12 | 20361.1 | 193 |
| B6SZS2 | 40S ribosomal protein S9 | 22546.9 | 193 |
| B6T3J9 | PRA1 family protein | 20686.1 | 193 |
| B6TEC7 | Grx_S15.2-glutaredoxin subgroup II | 21256.1 | 193 |
| B6TH64 | 40S ribosomal protein S9-2 | 22516.9 | 193 |
| B6THT0 | Peroxiredoxin-5 | 20678.3 | 193 |
| K7U7F7 | Ripening-related protein 3 | 20413.5 | 193 |
| Q6PLR8 | Putative ras-like small GTP binding ptotein | 22012.3 | 193 |
| A0A1D6KWK2 | 60S ribosomal protein L24-2 | 21920.7 | 194 |
| A0A1D6NM06 | Uncharacterized protein | 20536.2 | 194 |
| B4FBG7 | ADP-ribosylation factor B1B | 21839.9 | 194 |
| B4FGI9 | Trafficking protein particle complex subunit | 21702.8 | 194 |
| B6SHZ5 | Thioredoxin-like protein 1 | 21418.1 | 194 |
| B6T7B2 | 40S ribosomal protein S9-2 | 22431.8 | 194 |
| B6TGW6 | Uncharacterized protein | 21656.2 | 194 |
| B6UHT3 | Uncharacterized protein | 19032.8 | 194 |
| A0A1D6MLW6 | Putative calcium-binding protein CML27 | 20493.2 | 195 |
| A0A1D6KNK5 | DUF761 domain protein | 20717.7 | 196 |
| A0A1D6P5R1 | CDP-diacylglycerol--inositol 3-phosphatidyltransferase | 22030.9 | 196 |
| A0A1D6PYB8 | Nuclear transport factor1 | 21136 | 196 |
| B4FX41 | COP9 signalosome complex subunit 8 | 22109.8 | 196 |
| C0HGH7 | Universal stress family protein | 21162.1 | 196 |
| Q8LK07 | Histone H1-like protein HON101 | 19884.5 | 196 |
| A0A1D6GBA8 | Bifunctional riboflavin kinase/FMN phosphatase | 21977 | 197 |
| A0A317Y7E9 | DNA repair RAD52-like protein 2, chloroplastic | 21287.8 | 197 |
| B6TNG3 | Ubiquitin carrier protein | 21388 | 197 |
| B6U897 | Structural constituent of ribosome | 21481.4 | 197 |
| C0P5L7 | Gluconokinase | 20926.5 | 197 |
| A0A1D6EEE8 | Putative carbohydrate esterase | 21724.8 | 198 |
| A0A1D6EGC6 | 60 kDa jasmonate-induced protein | 21290.8 | 198 |
| A0A1D6GPX9 | Ubiquitin-conjugating enzyme E2 4 | 22747 | 198 |
| A0A1D6H288 | Putative small nuclear ribonucleoprotein F | 22158.4 | 198 |
| A0A1D6K136 | Membrane steroid-binding protein 1 | 21507.8 | 198 |
| A0A1D6LW26 | Alpha-L RNA-binding motif/Ribosomal protein S4 family protein | 23611.6 | 198 |
| A0A1D6LXV3 | ZCN11 | 21245.4 | 198 |
| B4FMF3 | PLASMODESMATA CALLOSE-BINDING PROTEIN 2 | 18918 | 198 |
| B6TF12 | Glycine-rich RNA-binding protein 8 | 21110.6 | 198 |
| B6THS4 | Reticulon-like protein | 21862.7 | 198 |
| C0PA44 | Uncharacterized protein | 21005.1 | 198 |
| A0A1D6FXN9 | Uncharacterized protein | 21416.2 | 199 |
| A0A1D6G1D6 | Putative ubiquitin-conjugating enzyme family | 22318.5 | 199 |
| A0A1D6GTC2 | NADH dehydrogenase [ubiquinone] 1 alpha subcomplex subunit 12 | 22827.7 | 199 |
| A0A1D6HQP2 | La-related protein 6C | 22440.5 | 199 |
| A0A1D6MK43 | Alba DNA/RNA-binding protein | 21798.8 | 199 |
| B4FP34 | Frataxin | 22003.7 | 199 |
| B6SHZ1 | 40S ribosomal protein S5 | 22366.5 | 199 |
| B6SI83 | 40S ribosomal protein S5 | 22226.4 | 199 |
| B6T1M3 | 3-5 exoribonuclease CSL4 | 21145 | 199 |
| C0P6N0 | Calcium load-activated calcium channel | 21027.1 | 199 |
| A0A1D6E5U8 | Protein RETICULATA chloroplastic | 20691.2 | 200 |
| A0A1D6H719 | Serine/threonine-protein phosphatase | 23041.1 | 200 |
| A0A1D6MA73 | Pistil-specific extensin-like protein | 21086.2 | 200 |
| A0A1D6NEY5 | Transcription initiation factor IIB-2 | 22071.5 | 200 |
| B4FGH8 | Uncharacterized protein | 21033.2 | 200 |
| B4G1P2 | NADH dehydrogenase2 | 21941.3 | 200 |
| B6SK58 | Harpin inducing protein | 21383.5 | 200 |
| B6TCI5 | Ras-related protein RABF1 | 21630.1 | 200 |
| B6TDQ5 | NADH-ubiquinone oxidoreductase 20 kDa subunit | 21991.4 | 200 |
| B6UA50 | Nuclear transcription factor Y subunit C-2 | 21549.4 | 200 |
| A0A096QVF7 | Frataxin 2 | 22028.6 | 201 |
| A0A1D6KU38 | Inosine triphosphate pyrophosphatase | 21863 | 201 |
| A0A1D6NIB5 | Protein C2-DOMAIN ABA-RELATED 4 | 22723.3 | 201 |
| A0A1P8NLV1 | Auxin binding protein 1 | 21976.8 | 201 |
| A0A1P8NLV5 | Auxin binding protein 1 | 22106 | 201 |
| A0A1R3MC15 | Inosine triphosphate pyrophosphatase | 21834.9 | 201 |
| B4FU92 | Uncharacterized protein | 22851.2 | 201 |
| B4FWT5 | Soluble inorganic pyrophosphatase | 22765.8 | 201 |
| B6TBT3 | Peptidyl-prolyl cis-trans isomerase | 21569.5 | 201 |
| B6TDH5 | Ubiquitin-conjugating enzyme E2-17 kDa | 21834.9 | 201 |
| B6TRU3 | Protein RER1 | 22179.6 | 201 |
| B6TSR5 | Frataxin | 22070.7 | 201 |
| B6UI95 | Calmodulin-related protein 2, touch-induced | 20881.8 | 201 |
| G1FMQ3 | Auxin-binding protein | 22002.8 | 201 |
| A0A096QUD1 | Putative glycolipid transfer protein (GLTP) family protein | 22552.7 | 202 |
| A0A1D6L996 | 40S ribosomal protein S26 | 23203.5 | 202 |
| A0A1D6N0U0 | NADPH:quinone oxidoreductase | 21880.8 | 202 |
| B4FAQ5 | VIP1 protein | 21200 | 202 |
| B4FL61 | Uncharacterized protein | 19959 | 202 |
| B4FX42 | Glycolipid transfer protein 1 | 22543.8 | 202 |
| B6SK40 | Mitochondrial import receptor subunit TOM20 | 22205.6 | 202 |
| B6T6M9 | Pleckstrin homology domain containing, family A | 22571.8 | 202 |
| B6T7P5 | Cleavage and polyadenylation specificity factor 5 | 23200 | 202 |
| B6TPU9 | RAN guanine nucleotide release factor | 21591.2 | 202 |
| B6TVY3 | Peptidyl-prolyl cis-trans isomerase | 21670.6 | 202 |
| C0HF45 | Mitochondrial import receptor subunit TOM20-4 | 22238.7 | 202 |
| K7V395 | CCAAT-HAP5 transcription factor | 21530.2 | 202 |
| A0A1D6J2Q2 | Pectin methylesterase inhibitor 1 | 21739.6 | 203 |
| B4F7V6 | Ras-related protein RABF2a | 22077.8 | 203 |
| B4FAE1 | Ras-related protein RABD2c | 22506.2 | 203 |
| B4FB35 | Translation machinery-associated protein 22 | 21620.5 | 203 |
| B4FQY4 | Ras-related protein RABD2c | 22488.2 | 203 |
| B4FWD0 | NAD(P)H dehydrogenase (Quinone) FQR1 | 21559.6 | 203 |
| B6SU67 | Ras-related protein RHN1 | 22248.1 | 203 |
| B6SZD1 | Mitochondrial import receptor subunit TOM20-4 | 22416 | 203 |
| B6T3L4 | Ribosomal protein L9, N-terminal domain containing protein | 23299.1 | 203 |
| B6TDT8 | Glycine-rich RNA-binding protein 2 | 21586.9 | 203 |
| B6TFN1 | Minor allergen Alt a 7 | 21669.9 | 203 |
| B6UFM9 | Peroxisomal membrane protein PMP22 | 23213.3 | 203 |
| C0HI97 | ATP synthase3 | 21298.3 | 203 |
| K7VIA7 | Nascent polypeptide-associated complex alpha subunit-like protein | 22229.2 | 203 |
| A0A1D6G9W0 | Bifunctional inhibitor/lipid-transfer protein/seed storage 2S albumin superfamily protein | 22000.5 | 204 |
| A0A1D6JDE4 | Protein AE7 | 23170.3 | 204 |
| B4F944 | Ras-related protein RABF2b | 22085.8 | 204 |
| B4FXW0 | Uncharacterized protein | 22925.8 | 204 |
| B6T267 | Ribosomal protein L15 | 24436.3 | 204 |
| B6T8X4 | Thioredoxin family Trp26 | 22590 | 204 |
| B6T903 | Nascent polypeptide-associated complex alpha subunit-like protein | 22429.5 | 204 |
| B6T9W6 | Charged multivesicular body protein 1b | 22799.9 | 204 |
| B6TMF3 | Wound/stress protein | 21586.2 | 204 |
| B6TP42 | Ribosomal protein L15 | 24360.3 | 204 |
| B6U898 | Dirigent protein | 20814.8 | 204 |
| C0PA88 | Uncharacterized protein | 22326.2 | 204 |
| C4J557 | Uncharacterized protein | 19973.7 | 204 |
| K7VH40 | NAD(P)H dehydrogenase (Quinone) FQR1 | 21668.7 | 204 |
| Q84LS4 | Superal1 | 22815.9 | 204 |
| A0A0B4J3B1 | Ras-related protein RABD1 | 23138.1 | 205 |
| A0A1D6GB06 | Uncharacterized protein | 23139.4 | 205 |
| A0A1D6HXQ3 | 40S ribosomal protein S4-3 | 22179.8 | 205 |
| A0A1D6KJN3 | 60S ribosomal protein L32 | 23564.3 | 205 |
| B4G1D2 | CBS domain protein | 22497.8 | 205 |
| B6SJ28 | Late embryogenesis abundant protein, group 3 | 21190.3 | 205 |
| B6SST2 | Arabinogalactan protein | 23031.2 | 205 |
| B6STP1 | Stress responsive protein | 22943.6 | 205 |
| B6T9A1 | Co-chaperone protein SBA1 | 22104.4 | 205 |
| B6TNK9 | Uncharacterized protein | 21596 | 205 |
| B6UHH3 | Eukaryotic translation initiation factor 3 subunit K | 23491.6 | 205 |
| C4JAE0 | Glycine-rich RNA-binding protein RZ1A | 21860.3 | 205 |
| C4JBB4 | Co-chaperone protein SBA1 | 22044.4 | 205 |
| K7WBY4 | Abscisic acid receptor PYR1 | 21990.4 | 205 |
| A0A1D6HNP0 | ATP-dependent Clp protease proteolytic subunit | 22672 | 206 |
| B4FGM1 | DNA-directed RNA polymerases II and IV subunit 5A | 24125.6 | 206 |
| B6SJA0 | 60S ribosomal protein L13a | 23630 | 206 |
| B6U154 | Harpin inducing protein | 21993.4 | 206 |
| B6U4J7 | Dirigent protein | 22127 | 206 |
| C0HFM4 | 60S ribosomal protein L13a-1 | 23628.9 | 206 |
| C0PA34 | Uncharacterized protein | 23135.9 | 206 |
| C4IYS0 | Uncharacterized protein | 22920.8 | 206 |
| D1MPZ8 | Superoxide dismutase [Cu-Zn] | 20867.2 | 206 |
| D1MPZ9 | Superoxide dismutase [Cu-Zn] | 20861.2 | 206 |
| K7W435 | Jacalin-related lectin 3 | 23600.8 | 206 |
| Q946V3 | Alpha globulin | 22298.3 | 206 |
| A0A1D6MBN3 | Programmed cell death protein 5 | 23713.8 | 207 |
| B4FL64 | Ribosomal protein L19 | 24114.4 | 207 |
| B6SSF8 | Transmembrane emp24 domain-containing protein 10 | 22922.2 | 207 |
| B6T1F1 | Ribosomal protein L19 | 24119.3 | 207 |
| B6T4I0 | Uncharacterized protein | 22978.4 | 207 |
| B6TQ68 | 60S ribosomal protein L19-2 | 24079.2 | 207 |
| B6TTB2 | Zinc knuckle (CCHC-type) family protein | 22105.4 | 207 |
| A0A1D6KC46 | Hsp20/alpha crystallin family protein | 22619.6 | 208 |
| A0A1D6PTR2 | HSP20-like chaperones superfamily protein | 22496.9 | 208 |
| B4FJZ7 | Ribosomal protein L19 | 24207.5 | 208 |
| B4FRS1 | Ras-related protein RABH1b | 22943 | 208 |
| B4FTM9 | Uncharacterized protein | 21922 | 208 |
| B4FWR7 | 60S ribosomal protein L13 | 23937 | 208 |
| B6T5F2 | 60S ribosomal protein L13 | 23893.9 | 208 |
| B6TP60 | CSD transcription factor | 20246.4 | 208 |
| B6TQQ8 | Uncharacterized protein | 22676.2 | 208 |
| B6TXB5 | 22.0 kDa class IV heat shock protein | 22831.6 | 208 |
| B6U684 | Ferredoxin | 22224.5 | 208 |
| B6UDX0 | Tankyrase 1 | 22084.2 | 208 |
| C0P447 | Secretory carrier-associated membrane protein | 23511.6 | 208 |
| C0P8J4 | 22.0 kDa heat shock protein | 22831.6 | 208 |
| C0P995 | Uncharacterized protein | 23511.7 | 208 |
| K7VW99 | ER membrane protein complex subunit 7-like protein | 22706.2 | 208 |
| K7WE09 | Betaine aldehyde dehydrogenase 2 mitochondrial | 23218.9 | 208 |
| A0A1D6G555 | Ubiquitin carrier protein | 22808.6 | 209 |
| A0A1D6MXK3 | Jasmonate-induced protein | 23027.5 | 209 |
| B4FFF1 | Ran-binding protein 1 homolog a | 23119.5 | 209 |
| B6SJJ4 | Jasmonate-induced protein | 23000.4 | 209 |
| B6T2N3 | Peptide-methionine (R)-S-oxide reductase | 22829.6 | 209 |
| B6TEF3 | SelT/selW/selH selenoprotein domain containing protein | 22390.8 | 209 |
| B6THS7 | Splicing factor, arginine/serine-rich 4 | 24616 | 209 |
| B6TNZ3 | Maf-like protein CV_0124 | 22792.9 | 209 |
| B6U7F9 | Splicing factor, arginine/serine-rich 4 | 24596.9 | 209 |
| B8QXQ9 | Ribosome-inactivating protein (Fragment) | 23349.7 | 209 |
| C0P6G4 | PRA1 family protein | 24264.2 | 209 |
| C0PNK2 | Uncharacterized protein | 22475.9 | 209 |
| K7TN39 | Selenium binding | 22504 | 209 |
| K7W0N3 | RNA polymerase II transcriptional coactivator KELP | 23333.8 | 209 |
| A0A1D6E0E7 | Peptidyl-tRNA hydrolase II (PTH2) family protein | 22366.6 | 210 |
| A0A1D6NGV9 | Vacuolar protein sorting protein 25 | 23948.4 | 210 |
| B4FDH9 | Uncharacterized protein | 23435.5 | 210 |
| B4FL36 | Ras-related protein RABB1a | 23055.9 | 210 |
| B6SIM7 | Proteasome subunit beta | 23083.3 | 210 |
| B6SIX9 | Uncharacterized protein | 22779.7 | 210 |
| B6SYI4 | Uncharacterized protein | 23083.3 | 210 |
| B6T5Q6 | Uncharacterized protein | 23046 | 210 |
| B6T7N7 | Uncharacterized protein | 22983.2 | 210 |
| B6TL26 | NADPH quinone oxidoreductase 2 | 22287.1 | 210 |
| B6TQB3 | Ran-binding protein 1 | 23169.7 | 210 |
| B6TQZ7 | Eukaryotic translation initiation factor 4E-2 | 23509.3 | 210 |
| B6TVP5 | VAMP-like protein YKT62 | 23205 | 210 |
| B6TW56 | SAM domain family protein | 23694.6 | 210 |
| B6U232 | Ras-related protein Rab-2-A | 23060.9 | 210 |
| B6U467 | ER membrane protein complex subunit 7-like protein | 22707.2 | 210 |
| B6UC77 | Dirigent protein | 21986.8 | 210 |
| C0PMP6 | Uncharacterized protein | 23326.1 | 210 |
| C4J094 | Superoxide dismutase [Cu-Zn] | 21917.4 | 210 |
| O50019 | Ypt homolog3 | 23041.9 | 210 |
| A0A1D6EN58 | Uncharacterized protein | 23383.9 | 211 |
| B4FAQ9 | DREPP4 protein | 22643.4 | 211 |
| B4FVH9 | Transmembrane emp24 domain-containing protein 10 | 23204.6 | 211 |
| B4FY69 | S-adenosylmethionine-dependent methyltransferase | 23396.6 | 211 |
| B6T4A2 | Proteasome subunit beta | 23241.5 | 211 |
| B6TMR8 | Chloroplast small heat shock protein | 22818.4 | 211 |
| B6TNI6 | DREPP4 protein | 22609.4 | 211 |
| B6UG58 | DREPP4 protein | 22601.4 | 211 |
| C0PFF5 | Proteasome subunit beta | 23213.5 | 211 |
| A0A075T3E7 | Pyrabactin resistance-like protein | 22768.2 | 212 |
| A0A1D6Q0H2 | Thioredoxin domain-containing protein 9-like protein | 23876.7 | 212 |
| B4FM96 | UMP-CMP kinase | 23420.7 | 212 |
| B6T904 | UMP-CMP kinase | 23324.6 | 212 |
| B6TMA1 | SAM domain family protein | 23845.9 | 212 |
| B6TSE5 | Ras-related protein Rab-18 | 23496.6 | 212 |
| B6TZZ4 | Mitochondrial glycoprotein | 23125.9 | 212 |
| C4J0W6 | Abscisic acid receptor PYR1 | 22784.3 | 212 |
| A0A1D6FTA4 | Ras-related protein RABG3f | 23757.7 | 213 |
| A0A1R3NCT7 | CASP-like protein | 22311.4 | 213 |
| B4FE73 | Transmembrane emp24 domain-containing protein p24beta2 | 24981.2 | 213 |
| B4FIW7 | Transmembrane emp24 domain-containing protein p24beta3 | 23956.2 | 213 |
| B4FPX0 | 26S proteasome non-ATPase regulatory subunit 9 | 23151.9 | 213 |
| B6SN63 | Late embryogenesis abundant protein 3 | 21306.5 | 213 |
| B6SZG7 | Peptidylprolyl isomerase | 21877.7 | 213 |
| B6T3F5 | 22.0 kDa class IV heat shock protein | 22637.6 | 213 |
| B6TE63 | Transmembrane emp24 domain-containing protein 3 | 24737.8 | 213 |
| B6TVZ1 | Vacuolar protein sorting-associated protein 28 homolog | 23046.1 | 213 |
| B6TZ58 | VAMP protein SEC22 | 23251.6 | 213 |
| B6UGM9 | Uncharacterized protein | 21773.2 | 213 |
| C0HDT7 | Abscisic acid receptor PYL5 | 23160.8 | 213 |
| A0A1D6F9M3 | Ubiquitin-conjugating enzyme E2 3 | 24604.9 | 214 |
| A0A1D6LYS1 | NAD(P)H dehydrogenase (Quinone) FQR1 | 22001.1 | 214 |
| B4F817 | Dehydroascorbate reductase like3 | 23419.6 | 214 |
| B4FJX6 | Soluble inorganic pyrophosphatase 4 | 24189.6 | 214 |
| B4FT31 | Dehydroascorbate reductase like1 | 23355.5 | 214 |
| B4FUW7 | Uncharacterized conserved protein UCP022280 | 23855.8 | 214 |
| B4FWX1 | Uncharacterized protein | 23978.3 | 214 |
| B4FXF9 | Soluble inorganic pyrophosphatase | 24369.5 | 214 |
| B6SUS6 | Uncharacterized protein | 22825 | 214 |
| B6TFE0 | VAMP protein SEC22 | 23228.5 | 214 |
| B6TQK5 | Adenylate cyclase | 23428.4 | 214 |
| B6TXD4 | Uncharacterized protein | 23675.9 | 214 |
| B6U1A7 | Glutathione S-transferase GSTF2 | 23793.3 | 214 |
| B6UCX2 | Chloride intracellular channel 6 | 23498.7 | 214 |
| C0PA19 | Acyl-CoA N-acyltransferase (NAT) superfamily protein | 23908.1 | 214 |
| C0PCV2 | 40S ribosomal protein S8 | 24309.8 | 214 |
| F1DJR5 | PLATZ transcription factor family protein (Fragment) | 24190.2 | 214 |
| P12653 | Glutathione S-transferase 1 | 23821.3 | 214 |
| Q6RYT6 | Adenine phosphoribosyltransferase | 22705.1 | 214 |
| B4F7V4 | Ras-related protein Rab11A | 23593.4 | 215 |
| B4FB55 | Ras-related protein ARA-3 | 23881.8 | 215 |
| B4FJM7 | CDP-diacylglycerol--inositol 3-phosphatidyltransferase | 24334.6 | 215 |
| B4FSG4 | Jasmonate-induced protein | 24121.1 | 215 |
| B6TCF9 | ABC transporter G family member 5 | 23795.9 | 215 |
| B6TEQ2 | DAG protein | 24274.2 | 215 |
| B6TIP7 | Peptidyl-prolyl cis-trans isomerase | 23078.5 | 215 |
| C4J9U1 | Ras-related protein18A1 | 23739.8 | 215 |
| Q6UG58 | CDP-diacylglycerol--inositol 3-phosphatidyltransferase | 24279.4 | 215 |
| A0A172FJH0 | ATP-dependent Clp protease proteolytic subunit | 24742.3 | 216 |
| A0A1D6E8G8 | Bowman-Birk type bran trypsin inhibitor | 22959.3 | 216 |
| A0A317YD33 | Stromal cell-derived factor 2-like protein | 23407.1 | 216 |
| B4FDT6 | Eucaryotic initiation factor7 | 24103.9 | 216 |
| B4FQ28 | Ras-related protein RABA1f | 23978.8 | 216 |
| B6TBL6 | Mps one binder kinase activator-like 1A | 24675.4 | 216 |
| B6TL27 | ABA-induced protein | 24024 | 216 |
| B6TMU7 | Uncharacterized protein | 23628.2 | 216 |
| B6TVP8 | Stromal cell-derived factor 2 | 23420 | 216 |
| C0PAG2 | Uncharacterized protein | 24129.3 | 216 |
| C4J929 | Ribosomal protein | 24579.9 | 216 |
| A0A1D6GN55 | Uncharacterized protein | 24407.8 | 217 |
| A0A1D6NIA1 | Putative desiccation-related protein LEA14 | 23524.8 | 217 |
| A0A1D6PZJ5 | Calcineurin subunit B | 24512.6 | 217 |
| B4FCM4 | PRA1 family protein | 22656.7 | 217 |
| B4FGY0 | Calcyclin-binding protein | 24363.7 | 217 |
| B4FIB2 | Ras-related protein RABA1d | 23964.7 | 217 |
| B4FLV9 | Ras-related protein RGP2 | 23897 | 217 |
| B4FTQ0 | PRA1 family protein | 22563.6 | 217 |
| B4G1P8 | Peptidase C15 pyroglutamyl peptidase I-like | 23914.1 | 217 |
| B4G242 | Ras-related protein RABA4a | 24016.2 | 217 |
| B6SMW1 | PRA1 family protein | 22583.6 | 217 |
| B6SNG5 | 6,7-dimethyl-8-ribityllumazine synthase | 22659.9 | 217 |
| B6TEC3 | 6,7-dimethyl-8-ribityllumazine synthase | 22650.9 | 217 |
| B6TG03 | Ras-related protein RABA4a | 23911.1 | 217 |
| B6TMN1 | C/VIF2 | 22844.6 | 217 |
| B6UBY9 | Uncharacterized protein | 24500.8 | 217 |
| B6UED6 | Early nodulin-like protein 3 | 22894.4 | 217 |
| C4J0F3 | Uncharacterized protein | 23885 | 217 |
| C4J1S1 | Uncharacterized protein | 20475.8 | 217 |
| K7TTJ0 | Class IV heat shock protein | 23437.2 | 217 |
| A0A317Y853 | Vesicle transport protein | 25557.4 | 218 |
| A8HNN2 | Z1D alpha zein protein (Fragment) | 24436 | 218 |
| B6T649 | Heat shock 22 kDa protein | 23863.8 | 218 |
| B6TBD1 | Eukaryotic translation initiation factor 4E-1 | 24485.2 | 218 |
| B6TU44 | Rhicadhesin receptor | 22837 | 218 |
| D5KXY1 | Eukaryotic translation initiation factor small subunit | 24382.1 | 218 |
| A0A1D6GGB1 | Late embryogenesis abundant protein 31 | 22349.9 | 219 |
| A0A1D6LC68 | Outer envelope pore protein 24A chloroplastic | 23481.6 | 219 |
| A0A1X7YGA6 | Uncharacterized protein | 24058.8 | 219 |
| A0A1X7YI34 | Uncharacterized protein | 23094.7 | 219 |
| A0A317YFH3 | Proteasome subunit beta | 23935.9 | 219 |
| B4F9G6 | 60S ribosomal protein L6 | 24242.1 | 219 |
| B4FU22 | Chloride conductance regulatory protein ICln | 24173 | 219 |
| B4G1K3 | Calcyclin-binding protein | 24644.2 | 219 |
| B6SHW0 | 60S ribosomal protein L6 | 24288.2 | 219 |
| B6SLK4 | Elongation factor 1-beta 2 | 23407 | 219 |
| B6T0C0 | Heme-binding protein 2 | 23703.3 | 219 |
| B6U670 | PHD transcription factor | 24984.1 | 219 |
| B7ZYH1 | PLASMODESMATA CALLOSE-BINDING PROTEIN 2 | 20301.5 | 219 |
| C0HGB3 | Uncharacterized protein | 24867.8 | 219 |
| C0P624 | Thioesterase superfamily protein | 24068.1 | 219 |
| C0P6F7 | Uncharacterized protein | 24421.2 | 219 |
| C0PH34 | 60S ribosomal protein L6 | 24238.1 | 219 |
| C4J2G4 | Late embryogenesis abundant (LEA) hydroxyproline-rich glycoprotein family | 23006.3 | 219 |
| C4J8S0 | Succinate dehydrogenase subunit 4 mitochondrial | 23875.5 | 219 |
| D2JSQ1 | ATP synthase subunit 4 | 24414.4 | 219 |
| Q6R9J5 | ATPase subunit 4 | 24500.6 | 219 |
| A0A1D6GKZ3 | Osmotin-like protein M34 | 22450.2 | 220 |
| A0A1D6GVX5 | Peptidyl-prolyl cis-trans isomerase | 23617.2 | 220 |
| A0A1D6LN79 | Peptidyl-prolyl cis-trans isomerase | 23731.2 | 220 |
| A0A1D6N932 | Osmotin-like protein M34 | 23068.8 | 220 |
| A0A1D6NGC3 | Ras-related protein RABA1f | 24170.7 | 220 |
| A0A317Y5W7 | NADH dehydrogenase [ubiquinone] 1 alpha subcomplex assembly factor 3 | 24151.4 | 220 |
| A5HEH5 | GIF2 | 23397.2 | 220 |
| B6T8F4 | Ran-binding protein 1 homolog a | 23958.3 | 220 |
| B6TG43 | B-cell receptor-associated protein 31-like containing protein | 24802.6 | 220 |
| B6U632 | Ras-related protein Rab11B | 24154.7 | 220 |
| C0PAV8 | Eukaryotic translation initiation factor 4E-1 | 24593.3 | 220 |
| Q6R979 | NADH-ubiquinone oxidoreductase chain 6 | 25240.8 | 220 |
| A0A1D6FF14 | Alba DNA/RNA-binding protein | 24129.5 | 221 |
| A0A1D6GG99 | NADH-ubiquinone oxidoreductase 23 kDa subunit | 25482.6 | 221 |
| A0A1D6M104 | B-cell receptor-associated 31-like | 24692.4 | 221 |
| A0A1D6NHH6 | GTP-binding nuclear protein | 25113.5 | 221 |
| B4FC18 | Vesicle transport v-SNARE 12 | 24874.3 | 221 |
| B4FES7 | Uncharacterized protein | 23927.1 | 221 |
| B4FQ23 | Lactoylglutathione lyase | 23442.5 | 221 |
| B4FRQ7 | Proteasome subunit beta | 24320.4 | 221 |
| B4G1P4 | GTP-binding nuclear protein | 25060.5 | 221 |
| B6SMF2 | Vesicle transport v-SNARE 12 | 24844.3 | 221 |
| B6T1R8 | Hsp20/alpha crystallin family protein | 23975.2 | 221 |
| B6T5R1 | Ran-binding protein 1 homolog a | 23775.3 | 221 |
| B6TGM2 | Adenine phosphoribosyltransferase 2 | 24078.7 | 221 |
| B6TUE7 | Vesicle transport v-SNARE 12 | 24899.4 | 221 |
| B6TUW3 | VAMP protein SEC22 | 24264.8 | 221 |
| B6U6C5 | Auxin-binding protein ABP20 | 23120.5 | 221 |
| K7V044 | GTP-binding nuclear protein | 25173.7 | 221 |
| Q9ZP62 | Glutathione transferase III(A) | 23924.3 | 221 |
| A0A1D6KYW8 | Deoxyuridine 5'-triphosphate nucleotidohydrolase | 22934.1 | 222 |
| A0A1D6M1N0 | Succinate dehydrogenase subunit 4 mitochondrial | 24367 | 222 |
| A0A1D6P122 | Polyadenylate-binding protein-interacting protein 9 | 23826.6 | 222 |
| A0A1R3QP52 | Glutathione transferase23 | 24878.6 | 222 |
| A0A317YHK1 | Superoxide dismutase [Cu-Zn] 2 | 23892.8 | 222 |
| B4FV54 | Uncharacterized protein | 23483.1 | 222 |
| B4FW42 | Uncharacterized protein | 25177.9 | 222 |
| B4G0R1 | Nucleoside diphosphate kinase | 23998.4 | 222 |
| B6SGV9 | Lactoylglutathione lyase | 24987.2 | 222 |
| B6SZF0 | Metal-dependent phosphohydrolase, HD subdomain | 25014.2 | 222 |
| B6TLW2 | Nucleoside diphosphate kinase | 23968.4 | 222 |
| Q9ZP61 | GST6 protein | 25121.7 | 222 |
| A0A1D6JPD5 | 1,2-dihydroxy-3-keto-5-methylthiopentene dioxygenase | 26238.4 | 223 |
| A0A1D6QGS9 | Vacuolar protein sorting-associated protein 32 homolog 1 | 24984.8 | 223 |
| B4FUH1 | NADH dehydrogenase [ubiquinone] iron-sulfur protein 8, mitochondrial | 25724.9 | 223 |
| B6T084 | Pyrrolidone-carboxylate peptidase | 24438.9 | 223 |
| B6T4F7 | Charged multivesicular body protein 2a | 25074.5 | 223 |
| B6TJP8 | Charged multivesicular body protein 2a | 25036.5 | 223 |
| C0HJ53 | Thioesterase superfamily protein | 24250.2 | 223 |
| C0P5U8 | Glutathione S-transferase2 | 24570.2 | 223 |
| Q548E9 | 27 kDa gamma-zein | 23688.6 | 223 |
| Q5IBC6 | DANA2 | 23865.7 | 223 |
| A0A1D6GFH5 | Histone H1 | 23016.4 | 224 |
| A0A1D6MWZ7 | Uncharacterized protein | 24020.7 | 224 |
| A0A1D6NZE6 | Uncharacterized protein | 24925.4 | 224 |
| B4FRF4 | Nucleic acid-binding OB-fold-like protein | 24710.9 | 224 |
| B4FTF8 | Glutathione S-transferase | 24826.1 | 224 |
| B6T8R6 | Ubiquitin carboxyl-terminal hydrolase | 24331.2 | 224 |
| B6TYS6 | Glutathione S-transferase zeta class | 24755.1 | 224 |
| O24595 | Glutathione transferase | 25118.8 | 224 |
| Q94FF4 | Globulin 1 (Fragment) | 25685.7 | 224 |
| A0A1D6F4L4 | SNARE-like superfamily protein | 25336.9 | 225 |
| A0A1D6GLJ6 | Trigger factor-like protein TIG Chloroplastic | 24244.2 | 225 |
| A0A1D6HEK8 | Vesicle-associated protein 1-1 | 25051.8 | 225 |
| B6SK22 | Chalcone-flavonone isomerase family protein | 23729.5 | 225 |
| B6SPI2 | Uncharacterized protein | 23078.6 | 225 |
| B6STC4 | Uncharacterized protein | 24173.9 | 225 |
| B6SUK9 | L-ornithine N5-acetyltransferase NATA1 | 24133.3 | 225 |
| B6TA56 | Histone H1 | 23887.6 | 225 |
| B6TIR4 | Ribulose-phosphate 3-epimerase | 24140.6 | 225 |
| C4JB68 | Vesicle-associated protein 1-1 | 25212 | 225 |
| Q75T24 | MinE | 24903.9 | 225 |
| Q9FQC0 | Glutathione S-transferase GST 19 | 25240.8 | 225 |
| Q9FQC7 | Glutathione S-transferase | 25775.9 | 225 |
| A0A1D6ENH9 | Ras-related protein RABA4c | 25044.3 | 226 |
| A0A1D6LBB5 | Mitochondrial transcription termination factor family protein | 24977.4 | 226 |
| A0A1X7YII1 | Uncharacterized protein | 24808.7 | 226 |
| B4FGG7 | Calcium ion binding protein | 26032.2 | 226 |
| B4FL35 | Eukaryotic translation initiation factor 3 subunit K | 25789.4 | 226 |
| B4FUT3 | Germin-like protein subfamily 1 member 17 | 24603 | 226 |
| B6T2E1 | DANA2 | 24091 | 226 |
| B6T7H0 | Glutathione S-transferase 6 | 25786.1 | 226 |
| B6TG29 | Histone H1 | 23859.4 | 226 |
| B6TVW2 | Germin-like protein subfamily 1 member 17 | 24589 | 226 |
| B8A1U6 | Ribosomal protein L23/L15e family protein | 25135.2 | 226 |
| C4JAF3 | NifU-like protein 2 chloroplastic | 23831.4 | 226 |
| Q4A1J7 | Cysteine proteinase inhibitor (Fragment) | 24562.9 | 226 |
| Q9FQD0 | Glutathione S-transferase 6 | 25877.3 | 226 |
| A0A1D6MHC8 | Early nodulin 20 | 23131.7 | 227 |
| A0A1D6PMN8 | Glutathione transferase25 | 24764.2 | 227 |
| B4FRJ7 | Fumarylacetoacetate (FAA) hydrolase family | 24394.8 | 227 |
| B4FSU4 | SOUL heme-binding family protein | 24878.2 | 227 |
| B6SIY7 | Uncharacterized protein | 25521.3 | 227 |
| B6TV71 | Acylpyruvase FAHD1, mitochondrial | 24380.8 | 227 |
| C0PKL0 | Chalcone-flavonone isomerase family protein | 24141.1 | 227 |
| K7UN56 | Ubiquinone biosynthesis protein COQ4 homolog, mitochondrial | 25617.2 | 227 |
| Q2XXK5 | Zeamatin-like protein | 24031 | 227 |
| Q94FF1 | Globulin 1 (Fragment) | 26097.1 | 227 |
| A0A1D6JEV8 | Starch synthase IIIb-1 | 25703.3 | 228 |
| B6TDW7 | Secretory protein | 24482.1 | 228 |
| B6U5U2 | Uracil phosphoribosyltransferase | 24700.5 | 228 |
| C0P9T1 | Uncharacterized protein | 25964.1 | 228 |
| K7TT73 | 40S ribosomal protein S3-1 | 25448.6 | 228 |
| B4F881 | Transmembrane emp24 domain-containing protein p24delta9 | 25865.5 | 229 |
| B4FBD6 | Ribonuclease 1 | 25032 | 229 |
| B4FSA2 | Thioredoxin domain-containing protein 9 | 25821 | 229 |
| B4FSU1 | Transmembrane emp24 domain-containing protein 10 | 25713.3 | 229 |
| B4FVJ9 | Uncharacterized protein | 25671.2 | 229 |
| B6SIJ5 | Elongation factor 1-delta 1 | 24878.7 | 229 |
| B6SJB3 | Peroxiredoxin | 24905.2 | 229 |
| B6T450 | Eukaryotic translation initiation factor 4E type 3 | 26474.3 | 229 |
| B6TB97 | 40S ribosomal protein S3 | 25557.7 | 229 |
| B6THU5 | Elongation factor 1-delta 1 | 24782.6 | 229 |
| B6TLS0 | Ubiquitin carboxyl-terminal hydrolase | 24987.8 | 229 |
| B6TP74 | Structural molecule | 25439.1 | 229 |
| C0HF65 | Thioredoxin domain-containing protein PLP3B | 25798 | 229 |
| C0PMS4 | Uncharacterized protein | 25226.7 | 229 |
| C0PNJ6 | Vacuolar protein sorting-associated protein 20 homolog 2 | 25778.2 | 229 |
| Q9FQ97 | Glutathione S-transferase GST 42 (Fragment) | 24951.4 | 229 |
| Q9FQB7 | Glutathione S-transferase GST 22 (Fragment) | 25129.8 | 229 |
| A0A1D6LZA7 | 60S ribosomal protein L18-3 | 25959.3 | 230 |
| A0A1D6P3Y5 | Proteasome subunit beta | 25714.3 | 230 |
| A0A317Y9B1 | Germin-like protein 3-7 | 23952 | 230 |
| B4F9U6 | Reticulon-like protein | 25229.5 | 230 |
| B4FVD5 | V-type proton ATPase subunit E3 | 26629.3 | 230 |
| B6SSJ6 | Protein P21 | 23756.2 | 230 |
| B6SZN2 | ATP-dependent Clp protease ATP-binding subunit CLPT1 chloroplastic | 24651.9 | 230 |
| B6T2T0 | DNA binding activity2 | 26613.3 | 230 |
| B8A1M0 | Dephospho-CoA kinase | 25687.8 | 230 |
| C0P3R8 | Glutathione peroxidase | 24993.4 | 230 |
| A0A317Y1Z9 | Putative glutathione S-transferase GSTU6 | 25393.8 | 231 |
| B4FLD1 | Syntaxin-51 | 25920.8 | 231 |
| B4FSU2 | Rho guanine nucleotide exchange factor | 25639.1 | 231 |
| B6SS87 | Glutathione S-transferase GSTU6 | 25560.4 | 231 |
| B6TI09 | Eukaryotic translation initiation factor 3 subunit J | 25801.5 | 231 |
| B6TJA9 | Chalcone-flavonone isomerase family protein | 23952.2 | 231 |
| B6UI06 | Late embryogenesis abundant protein, group 3 | 23542.7 | 231 |
| B8A221 | Arginine/serine-rich splicing factor RSZ28V | 25949.5 | 231 |
| C0P3S5 | Acylpyruvase FAHD1, mitochondrial | 24731.3 | 231 |
| Q08704 | Chalcone--flavonone isomerase | 24249.5 | 231 |
| Q9FQA9 | Glutathione S-transferase GST 30 | 25223.7 | 231 |
| A0A1D6HMW8 | Serine/arginine-rich splicing factor RSZ22 | 25930.1 | 232 |
| A0A317YH81 | Eukaryotic translation initiation factor isoform 4E-2 | 25772 | 232 |
| B4FN58 | Protein-L-isoaspartate O-methyltransferase | 24782.1 | 232 |
| B6TBD5 | IMP dehydrogenase | 24603.1 | 232 |
| B6TD62 | Membrane steroid-binding protein 1 | 24567.4 | 232 |
| B6TF80 | Germin-like protein subfamily 3 member 2 | 24350.5 | 232 |
| B6TLM5 | Glutathione S-transferase GSTU6 | 25652.4 | 232 |
| B6TRA3 | Uncharacterized protein | 26508 | 232 |
| B6TWB5 | Vacuolar protein sorting 37C | 26473.4 | 232 |
| B6TWC3 | Rhicadhesin receptor | 23741.1 | 232 |
| B6U9F3 | Tonneau 1b | 25858.4 | 232 |
| C0P8K8 | Protein ABA DEFICIENT 4 chloroplastic | 25030.1 | 232 |
| C0PN11 | Uncharacterized protein | 24864.3 | 232 |
| C4J0B8 | ABA-induced protein | 25808.3 | 232 |
| K7W229 | Syntaxin-61 | 26580.8 | 232 |
| A0A1D6JM99 | Heavy metal transport/detoxification superfamily protein | 24892.1 | 233 |
| A0A317Y494 | Succinate dehydrogenase subunit 5, mitochondrial | 25272.8 | 233 |
| B4F9H6 | Superoxide dismutase | 25226.5 | 233 |
| B4FBT0 | Uncharacterized protein | 25486.6 | 233 |
| B4FK63 | Spliceosomal protein | 26138.8 | 233 |
| B6SII2 | Uncharacterized protein | 25438.4 | 233 |
| B6TBW2 | ATP synthase delta chain | 25565.4 | 233 |
| B6TIH5 | Uncharacterized protein | 25590.9 | 233 |
| K7VFF7 | Peroxiredoxin-5 | 23759.9 | 233 |
| Q9FQA6 | Glutathione S-transferase GST 33 | 25586.2 | 233 |
| A0A1D6H9X6 | Germin-like protein subfamily 3 member 2 | 24577.8 | 234 |
| B6TL18 | Uncharacterized protein | 25944.3 | 234 |
| B6UC94 | Elongation factor P | 25733 | 234 |
| C0PED2 | Ribosomal protein S24e family protein | 25877.3 | 234 |
| C0PL41 | Uncharacterized protein | 25348.4 | 234 |
| C4IZ87 | Uncharacterized protein | 26482.6 | 234 |
| Q9FQC4 | Glutathione S-transferase F13 | 25563.3 | 234 |
| B4F925 | Superoxide dismutase | 25587 | 235 |
| B4FFX7 | Proteasome subunit alpha type | 25864.2 | 235 |
| B6T969 | Proteasome subunit alpha type | 25880.2 | 235 |
| B6U0X2 | EMB514 | 25464.1 | 235 |
| B6UCC1 | Uncharacterized protein | 25677.1 | 235 |
| C0P5W8 | Putative peroxygenase 4 | 26385.7 | 235 |
| E7DDW6 | Clathrin light chain | 26096.8 | 235 |
| Q43273 | Superoxide dismutase | 25579 | 235 |
| Q9FQB8 | Glutathione S-transferase GST 21 | 26477 | 235 |
| A0A096RL35 | Uncharacterized protein | 26641.6 | 236 |
| B6TBI3 | Calcineurin B-like protein 3 | 26949.6 | 236 |
| B6UGS5 | Dehydrin Xero 1 | 24810 | 236 |
| C0P5X3 | Cytokinin riboside 5'-monophosphate phosphoribohydrolase | 24824.1 | 236 |
| A0A1D6F931 | 40S ribosomal protein S15-4 | 26616.9 | 237 |
| A0A1D6FRP9 | Ubiquitin carrier protein 7 | 26495.1 | 237 |
| A0A1D6IWR7 | Auxin-binding protein 4 | 26220.7 | 237 |
| B4FZP8 | Uncharacterized protein | 25723 | 237 |
| B6TJB6 | Proteasome subunit alpha type | 25977.1 | 237 |
| B6TMG4 | Nucleoside diphosphate kinase | 25724.2 | 237 |
| C0PBZ6 | Non-specific lipid-transfer protein | 24277 | 237 |
| Q71RX2 | Isopentenyl pyrophosphate isomerase1 | 27227.8 | 237 |
| A0A1D6EGD3 | Putative isoprenylcysteine alpha-carbonyl methylesterase ICMEL2 | 26167 | 238 |
| A0A1D6L548 | 60S ribosomal protein L13a-1 | 27134.2 | 238 |
| A0A1D6MM79 | Bowman-Birk type trypsin inhibitor | 25764.6 | 238 |
| A0A1Q0YQ12 | Oil body-associated protein 1B | 26223.8 | 238 |
| B4FRQ0 | MLP3.9 protein | 25563.1 | 238 |
| B6TM09 | Uncharacterized protein | 25592.1 | 238 |
| B6TUE5 | FIP1 | 25949.2 | 238 |
| B6TYR9 | Molybdopterin synthase catalytic subunit | 26179.4 | 238 |
| C0PBY7 | Nucleoside diphosphate kinase | 25972.5 | 238 |
| C4J9Y2 | Aluminum-induced protein homolog1 | 25339.7 | 238 |
| C4JBA7 | Uncharacterized protein | 24661.2 | 238 |
| Q6RW09 | Allene oxide cyclase | 25777 | 238 |
| Q8L8F3 | NADPH HC toxin reductase (Fragment) | 25733.2 | 238 |
| A0A1D6GLR4 | 5S rRNA binding protein | 25884.9 | 239 |
| A0A1D6N0I3 | AWPM-19-like family protein | 24896 | 239 |
| B6SKT6 | Protein LRP16 | 26085.8 | 239 |
| B6T7H7 | Protein LRP16 | 26038.5 | 239 |
| B6TDY7 | Exosome complex exonuclease RRP40 | 26142.1 | 239 |
| B6U9Z8 | Glutathione S-transferase GSTU6 | 26081.3 | 239 |
| A0A1D6G6R0 | Tetratricopeptide repeat (TPR)-like superfamily protein | 26059.7 | 240 |
| B4FPA0 | Alpha/beta-Hydrolases superfamily protein | 25728.3 | 240 |
| B4FRS6 | VIP1 protein | 25590.2 | 240 |
| Q41815 | Heat shock protein 26 | 26377.7 | 240 |
| A0A1D6MEX7 | 25.3 kDa vesicle transport protein | 28099.5 | 241 |
| B4FI76 | Adenylate kinase 4 | 26458.4 | 241 |
| B4FK20 | Vesicle-associated membrane protein 727 | 27473.3 | 241 |
| B6TPF0 | Uncharacterized protein | 26029 | 241 |
| B6UFW7 | Putative carbohydrate esterase | 26149.5 | 241 |
| C0PM74 | Adenylate kinase 4 | 26398.3 | 241 |
| C4IZH7 | Thioredoxin superfamily protein | 26870.6 | 241 |
| C4J093 | Adenylate kinase 4 | 26404.3 | 241 |
| A0A1D6JDF8 | Secretory carrier-associated membrane protein | 27520.4 | 242 |
| A0A1D6JLQ5 | THO complex subunit 7B | 27587.3 | 242 |
| B4FGZ7 | Uncharacterized protein | 27077.3 | 242 |
| B4FS25 | Nucleotide-diphospho-sugar transferase superfamily protein | 27116.3 | 242 |
| B4FT58 | Uncharacterized protein | 24715.9 | 242 |
| B4FUN3 | Uncharacterized protein | 27323.8 | 242 |
| B6SJ51 | FIP1 | 26395.7 | 242 |
| B6T7X5 | Uncharacterized protein | 26823.4 | 242 |
| B6THH7 | Isoamyl acetate-hydrolyzing esterase | 26586.8 | 242 |
| B6TPA4 | Carnitine racemase/ catalytic | 25657.4 | 242 |
| B6U144 | SelT-like protein | 26477.1 | 242 |
| B7ZY29 | GLABRA2 expression modulator | 26370.7 | 242 |
| C0PFY7 | Uncharacterized protein | 27769.9 | 242 |
| A0A1D6FRF8 | Succinate dehydrogenase subunit 4 mitochondrial | 26524.6 | 243 |
| A0A1D6H4W4 | Isochorismatase family protein rutB | 26777 | 243 |
| B4FAQ2 | Pyridoxal phosphate homeostasis protein | 26219.8 | 243 |
| B6TCR9 | Mitochondrial ATP synthase | 27682.8 | 243 |
| B6TLY8 | Mitochondrial ATP synthase | 27532.7 | 243 |
| B6U6A9 | Caleosin | 27285.3 | 243 |
| C0P4U8 | Putative ATP synthase 24 kDa subunit mitochondrial | 27572.7 | 243 |
| C0PCF2 | Golgi SNAP receptor complex member 1 | 26724 | 243 |
| C4JBH5 | Small basic membrane intrinsic protein1b | 25703.2 | 243 |
| A0A1D6HCU1 | Serine/arginine-rich splicing factor RSZ22 | 27481.1 | 244 |
| A0A1D6L558 | PLASMODESMATA CALLOSE-BINDING PROTEIN 2 | 25114.1 | 244 |
| A0A1D6MXM8 | Uveal autoantigen with coiled-coil domains and ankyrin repeats isoform 4 | 26918.2 | 244 |
| A0A317Y2J9 | Accelerated cell death 11 | 26916 | 244 |
| A0A317YAF1 | Aldose 1-epimerase | 26771.8 | 244 |
| B4F9V2 | VAP27 | 26761.4 | 244 |
| B4FQ03 | 60S ribosomal protein L7-2 | 28416.2 | 244 |
| B4FRJ3 | Proteasome subunit beta | 26124.4 | 244 |
| B6UD77 | DAG protein | 26999.3 | 244 |
| C0P9I4 | Uncharacterized protein | 25297.9 | 244 |
| C0PD15 | Uncharacterized protein | 26606.9 | 244 |
| Q8W120 | Histone H1-like protein | 24908.5 | 244 |
| A0A1D6FUU3 | RNA-binding (RRM/RBD/RNP motifs) family protein | 27024.8 | 245 |
| A0A1D6M5H4 | Protein LIKE COV 2 | 27543.9 | 245 |
| B4FKD5 | Eukaryotic translation initiation factor 6 | 26513.8 | 245 |
| B4FSH1 | LisH and RanBPM domains containing protein | 27946.6 | 245 |
| B4FTW5 | 60S ribosomal protein L7-1 | 28334 | 245 |
| B4FV48 | Cysteine proteinase inhibitor | 26696.1 | 245 |
| B6T8U7 | Chaperonin | 25574.2 | 245 |
| B6T915 | Eukaryotic translation initiation factor 6 | 26571.8 | 245 |
| B6TFP6 | Tubulin-specific chaperone B | 27377.5 | 245 |
| B6TH05 | DeSI-like protein | 26999.9 | 245 |
| C0PHH8 | Phosphoglycerate mutase family protein | 26644.9 | 245 |
| C4J938 | Uncharacterized protein | 27947.6 | 245 |
| A0A096S3I6 | Tubulin-folding cofactor B | 27461.6 | 246 |
| A0A1D6FWL7 | Uncharacterized protein | 26054 | 246 |
| A0A1D6HYA4 | Maternally expressed gene4 | 19839.7 | 246 |
| B4F7U0 | Proteasome subunit alpha type | 27487.2 | 246 |
| B4FTV3 | Chaperone DnaJ-domain superfamily protein | 29189.1 | 246 |
| B6T568 | Cupin, RmlC-type | 27570.2 | 246 |
| B6TLT8 | Chaperonin | 25753.4 | 246 |
| B6TXU4 | RWD domain-containing protein 1 | 27744.1 | 246 |
| B6U7Y2 | Glutathione peroxidase | 26824.4 | 246 |
| C0PFK3 | Proteasome subunit alpha type | 27417 | 246 |
| C0PHZ4 | Serine/arginine-rich splicing factor SC35 | 29204 | 246 |
| K7TSG0 | DAG protein | 27270.6 | 246 |
| K7V2N6 | Putative USP family protein | 27075.6 | 246 |
| P23444 | Histone H1 | 25347.9 | 246 |
| A0A1D6F5N5 | BRASSINOSTEROID INSENSITIVE 1-associated receptor kinase 1 | 26465.2 | 247 |
| A0A317YGN4 | Stem-specific protein TSJT1 | 26162.3 | 247 |
| A7U021 | Globulin-1 (Fragment) | 28242.4 | 247 |
| C0P8L5 | Uncharacterized protein | 27093.4 | 247 |
| Q4FZ52 | Cysteine proteinase inhibitor | 26843.3 | 247 |
| A0A1D6ES06 | Caffeoyl-CoA O-methyltransferase 1 | 26486 | 248 |
| A0A317Y3H6 | Tetratricopeptide repeat protein 1 | 27307.3 | 248 |
| A7TZX4 | Globulin-1 (Fragment) | 28328.5 | 248 |
| B4G080 | Caffeoyl-CoA O-methyltransferase 1 | 27318.8 | 248 |
| B6SKP5 | Osmotin-like protein | 26245.7 | 248 |
| B6SPB2 | Flavoprotein wrbA | 25728.2 | 248 |
| B6TCE9 | Peptidyl-prolyl cis-trans isomerase | 26217.6 | 248 |
| B6TJH8 | Mitochondrial glycoprotein family protein | 27167.7 | 248 |
| B8A212 | Uncharacterized protein | 25838.5 | 248 |
| C0HHW6 | Tetratricopeptide repeat (TPR)-like superfamily protein | 27351.3 | 248 |
| C0P330 | Vesicle-associated protein 1-1 | 27855.6 | 248 |
| A0A1D6G9Q0 | Proteasome subunit alpha type | 27276.9 | 249 |
| A0A1D6KKS3 | Putative transcription factor PosF21 | 26205.9 | 249 |
| A0A1D6PX25 | Ribosome-recycling factor chloroplastic | 27836.9 | 249 |
| A0A317Y0X5 | Derlin | 28472.2 | 249 |
| B4F7T6 | SelT-like protein | 27861.8 | 249 |
| B4F822 | Proteasome subunit alpha type | 27256.7 | 249 |
| B4FDK8 | Uncharacterized protein | 26948.6 | 249 |
| B4FF68 | THO complex subunit 4B | 26708.7 | 249 |
| B4FGB5 | Uncharacterized protein | 25418.9 | 249 |
| B4FR89 | Phosphomannomutase | 28129.8 | 249 |
| B6STT0 | Thaumatin-like protein | 25300.6 | 249 |
| B6SZA5 | 3-isopropylmalate dehydratase small subunit 2 | 25618.9 | 249 |
| B6U6N3 | Oligoribonuclease | 28111.8 | 249 |
| B6U6R6 | Histone H1 | 25221.9 | 249 |
| B6U9D5 | Glutathione S-transferase T1 | 28135.5 | 249 |
| C0HJ49 | Allene oxide cyclase 3 chloroplastic | 27237.8 | 249 |
| C0PI81 | Proteasome subunit alpha type | 27334 | 249 |
| C0PNN5 | Derlin | 28506.3 | 249 |
| K7V4Q5 | Proteasome subunit alpha type | 27197.6 | 249 |
| A0A1D6KTI5 | Vacuolar protein sorting-associated protein 2 homolog 3 | 27765.6 | 250 |
| A0A1Q1C9T5 | Secreted protein | 27349.6 | 250 |
| B4FAR8 | Proteasome subunit alpha type | 27067.5 | 250 |
| B4FCW6 | Uncharacterized protein | 27533.2 | 250 |
| B4FDY6 | Proteasome subunit alpha type | 27009.5 | 250 |
| B4FF90 | Phosducin-like protein 3 | 28341.5 | 250 |
| B4G031 | Ascorbate peroxidase2 | 27227.5 | 250 |
| B6SKR6 | Aquaporin TIP1.1 | 25919.9 | 250 |
| B6T675 | Peroxisomal coenzyme A diphosphatase NUDT7 | 27478.2 | 250 |
| B6T923 | Transcription factor APFI | 26489.8 | 250 |
| B6TGD3 | Caffeoyl-CoA O-methyltransferase 1 | 26738.3 | 250 |
| B6TJJ8 | 50S ribosomal protein L21 | 27725.1 | 250 |
| B6TM55 | APx1-Cytosolic Ascorbate Peroxidase | 27307.7 | 250 |
| B6TVL8 | APx2-Cytosolic Ascorbate Peroxidase | 27201.5 | 250 |
| B6UB73 | APx1-Cytosolic Ascorbate Peroxidase | 27384.7 | 250 |
| K7UUB0 | Triose phosphate isomerase3 | 26730.4 | 250 |
| A0A1D6LAH5 | Soluble epoxide hydrolase | 28046.9 | 251 |
| A0A1D6LIK1 | Peptidyl-prolyl cis-trans isomerase | 26443.8 | 251 |
| A0A1D6M5T1 | Peptidylprolyl isomerase | 27044.8 | 251 |
| A0A1X7YH95 | Uncharacterized protein | 28765.3 | 251 |
| B4FTS4 | Dirigent protein | 25110 | 251 |
| B6T1H0 | 40S ribosomal protein S6 | 28645.1 | 251 |
| B6TGL4 | ER membrane protein complex subunit 3 | 28368.9 | 251 |
| B6TR61 | MTA/SAH nucleosidase | 26564.5 | 251 |
| B6TYF4 | FIP1 | 27119.4 | 251 |
| C0HHI9 | Nucleotidyltransferase family protein | 28431.8 | 251 |
| C4J6J5 | 40S ribosomal protein S6 | 28604.1 | 251 |
| K0DCL1 | CA5P8 CCAAT-HAP5 type transcription factor (Fragment) | 27507.3 | 251 |
| K7U5U7 | Nicotinamidase 1 | 27492.5 | 251 |
| O04014 | 40S ribosomal protein S6 | 28617 | 251 |
| Q94F79 | NAP1-related protein 2 | 28440.9 | 251 |
| Q9FYS0 | 40S ribosomal protein S6 | 28544 | 251 |
| A0A1D6FMB4 | NifU-like protein 4 mitochondrial | 27711.2 | 252 |
| A0A1R3PYI9 | Oil body-associated protein 2A | 27726.2 | 252 |
| B4FT23 | 14-3-3-like protein GF14 nu | 28590.9 | 252 |
| B6T3S1 | Catalytic/ hydrolase | 27328 | 252 |
| B6T442 | Putative ribosomal protein S11 mitochondrial | 27915.6 | 252 |
| B6U1C7 | Vacuolar protein sorting-associated protein | 28102 | 252 |
| B8A1Y5 | Uncharacterized protein | 27370.5 | 252 |
| C0PPG1 | 25.3 kDa heat shock protein chloroplastic | 27435.4 | 252 |
| C4J3B1 | Pectinesterase | 27408.6 | 252 |
| Q84LL1 | Opaque-2 protein | 27061.9 | 252 |
| A0A1D6E511 | Soluble inorganic pyrophosphatase | 28641.4 | 253 |
| B4FNW1 | Triose phosphate isomerase4 | 27024.8 | 253 |
| B6SIG5 | Triosephosphate isomerase, cytosolic | 27066.9 | 253 |
| B6T346 | THO complex subunit 4B | 26553.4 | 253 |
| B6TC68 | Peroxisomal biogenesis factor 19 | 27523.7 | 253 |
| B8A0Z7 | Uncharacterized protein | 27452.3 | 253 |
| C0PN27 | Uncharacterized protein | 26681.6 | 253 |
| K7TZS3 | NEDD8-specific protease 1 | 27135.4 | 253 |
| A0A1D6E9G7 | Uncharacterized protein | 27396.1 | 254 |
| A0A1D6IK73 | Serine/arginine-rich splicing factor SC35 | 29943.9 | 254 |
| A0A1D6NAC7 | Polyketide cyclase / dehydrase and lipid transport protein | 28314.1 | 254 |
| B4F8J5 | Reticulon-like protein | 28343.7 | 254 |
| B4FUT7 | Uncharacterized protein | 26786.6 | 254 |
| B6TIK3 | Stress protein | 27345.8 | 254 |
| B6TVN1 | BCAS2 protein | 29010.9 | 254 |
| B6U543 | Versicolorin reductase | 26212.7 | 254 |
| C0PJK8 | Pre-mRNA-splicing factor SPF27-like protein | 29012.9 | 254 |
| C0PKD9 | Chaperonin10 | 26407 | 254 |
| C0PM53 | U1 small nuclear ribonucleoprotein A | 28302.6 | 254 |
| K7TNL9 | Uncharacterized protein | 27514.9 | 254 |
| P29036 | Ferritin-1, chloroplastic | 28024.5 | 254 |
| Q8W0W6 | CCAAT-HAP5-transcription factor 59 | 27890.8 | 254 |
| A0A317Y3E5 | Receptor-interacting serine/threonine-protein kinase 4 | 27201.1 | 255 |
| B4FL68 | Tetratricopeptide repeat (TPR)-like superfamily protein | 27729.6 | 255 |
| B6SNW4 | Glutamine amidotransferase subunit pdxT | 27603.2 | 255 |
| B6TPR6 | Electron transfer flavoprotein beta-subunit | 27565 | 255 |
| B6UE05 | 60S ribosomal protein L7a | 28840.2 | 255 |
| B7ZZT5 | Thymidylate kinase | 27983.7 | 255 |
| C0HGC9 | Uncharacterized protein | 28688.6 | 255 |
| A0A1D6FHA9 | Nucleic acid-binding OB-fold-like protein | 26932.6 | 256 |
| A0A1D6IN02 | Uncharacterized protein | 26895.2 | 256 |
| A0A317Y6I2 | Eukaryotic translation initiation factor 3 subunit K | 29338.5 | 256 |
| B4F7X5 | Reticulon-like protein | 27646 | 256 |
| B4FCK0 | USP family protein | 27140.9 | 256 |
| B4FDR1 | Signal recognition particle binding | 28292.2 | 256 |
| B4FRX0 | Peptide deformylase | 27271.3 | 256 |
| B6SHK4 | 14-3-3-like protein | 28961.4 | 256 |
| B6SMQ5 | Triose phosphate isomerase5 | 27339.2 | 256 |
| B6SRI4 | 14-3-3-like protein | 28947.3 | 256 |
| B6TG01 | Reticulon-like protein | 27627 | 256 |
| B6TK34 | PHD finger protein ALFIN-LIKE 6 | 29109.4 | 256 |
| B6TLL9 | Signal recognition particle receptor beta subunit | 28287.2 | 256 |
| B6TQ71 | Dual specificity protein phosphatase Diacylglycerol kinase catalytic region | 28273.6 | 256 |
| B6U284 | 14-3-3-like protein | 28864.1 | 256 |
| A0A1D6EPL1 | Annexin | 29251.1 | 257 |
| A0A1D6KKP0 | Putative 14-3-3 protein | 29007.2 | 257 |
| A0A1D6QSL7 | OTU-like cysteine protease family protein | 28912.7 | 257 |
| A0A317YFK7 | Uncharacterized protein | 27783.3 | 257 |
| B4F7Y1 | 60S ribosomal protein L7a-1 | 29188.6 | 257 |
| B4F893 | NAD(P)-binding Rossmann-fold superfamily protein | 26842 | 257 |
| B6T8S7 | RNA binding (RRM/RBD/RNP motifs) family protein | 28780.8 | 257 |
| B6T962 | NAD-dependent epimerase/dehydratase | 27769.3 | 257 |
| B6TGL5 | Reticulon-like protein | 28656.1 | 257 |
| B6TPG9 | FIP1 | 27561.9 | 257 |
| C0PCY4 | Alfin-like transcription factor | 28822 | 257 |
| C4JAG0 | Uncharacterized protein | 28527.1 | 257 |
| Q64HC2 | ASF/SF2-like pre-mRNA splicing factor SRP32 | 28909.8 | 257 |
| A0A317Y6E5 | Eukaryotic translation initiation factor 6 | 28065.7 | 258 |
| B4FEH6 | Expansin | 27993 | 258 |
| B4FGF5 | Hydroxyacylglutathione hydrolase cytoplasmic | 28755.7 | 258 |
| B4FYM2 | Ribosomal protein L25/Gln-tRNA synthetase anti-codon-binding domain | 28731.9 | 258 |
| B6SN55 | AIR12 | 25092.4 | 258 |
| B6T769 | 60S ribosomal protein L7a-1 | 29402.8 | 258 |
| Q6VWJ0 | Caffeoyl-CoA 3-O-methyltransferase 1 | 28876.7 | 258 |
| Q94F78 | Nucleosome/chromatin assembly factor A104 | 29331.6 | 258 |
| A0A0D5BXR6 | Methionine sulfoxide reductase | 28360.5 | 259 |
| A0A1D6QU12 | Putative pyridoxal 5'-phosphate synthase subunit PDX2 | 28015.7 | 259 |
| A0A317YBQ6 | Peptide methionine sulfoxide reductase A4, chloroplastic | 28432.6 | 259 |
| A0A317YJX1 | Vesicle-associated protein 1-1 | 28553.8 | 259 |
| B6T7F3 | Ribonuclease P | 28485.4 | 259 |
| B6TAV8 | Ribosome recycling factor | 27772.8 | 259 |
| B6TFS9 | 14-3-3-like protein A | 28621.9 | 259 |
| B6U8S9 | Peptide methionine sulfoxide reductase | 28389.6 | 259 |
| B7ZZK8 | Uncharacterized protein | 28793.8 | 259 |
| M1HIW8 | Arginine/serine-rich splicing factor SCL30 transcript II | 29653.4 | 259 |
| A0A1D6LF73 | Mitochondrial import inner membrane translocase subunit TIM22-2 | 26704.4 | 260 |
| B4F9R4 | 60S ribosomal protein L2 | 28102.2 | 260 |
| B4FM07 | 2-Cys peroxiredoxin BAS1 chloroplastic | 28163.9 | 260 |
| B4FQY8 | ATP-dependent Clp protease proteolytic subunit | 28022.7 | 260 |
| B6T8U5 | ER6 protein | 27737.9 | 260 |
| B6TAQ5 | 40S ribosomal protein S3a | 29741.5 | 260 |
| B6TDA9 | 2-cys peroxiredoxin BAS1 | 28147.9 | 260 |
| B6UG10 | 40S ribosomal protein S3a | 29723.4 | 260 |
| B6UHB6 | Histone H1 | 26689.6 | 260 |
| C4J9M7 | 2-Cys peroxiredoxin BAS1 chloroplastic | 28272 | 260 |
| Q6R985 | Cytochrome c oxidase subunit 2 | 29679.2 | 260 |
| A0A1D6IKI0 | Exosome complex component RRP41-like | 28306.8 | 261 |
| A0A1D6MXZ7 | Glycine-rich RNA-binding protein 3 mitochondrial | 25975.4 | 261 |
| A0A1D6PYX5 | 40S ribosomal protein S8 | 29905.2 | 261 |
| A0A1D6QMQ3 | Nuclear speckle RNA-binding protein A | 27342.1 | 261 |
| A0A317Y573 | Zinc finger protein GIS2 | 28649.3 | 261 |
| B4F8L1 | COP9 signalosome complex subunit 7 | 29560.4 | 261 |
| B4FKN3 | DNA-binding protein HEXBP | 28394 | 261 |
| B6SQE8 | 40S ribosomal protein S4 | 29324.9 | 261 |
| B6TBM4 | USP family protein | 27633.4 | 261 |
| B6TGT0 | 60S ribosomal protein L2 | 28219.3 | 261 |
| B6U3Y1 | Versicolorin reductase | 26942.3 | 261 |
| B7ZYI0 | 14-3-3-like protein GF14-6 | 29662.1 | 261 |
| C4J159 | Glycine-rich protein2 | 25682.2 | 261 |
| K7TL05 | General regulatory factor2 | 29629 | 261 |
| A0A1D6ELS0 | Histone H2B | 28161.7 | 262 |
| A0A1D6HTI7 | Bax inhibitor-1 family protein | 28990.2 | 262 |
| A0A1D6M1S3 | Late embryogenesis abundant (LEA) hydroxyproline-rich glycoprotein family | 26879.7 | 262 |
| A0A1D6N2A6 | Thiamine pyrophosphokinase | 28707.5 | 262 |
| A0A1Q0ZK72 | Tonoplast intrinsic protein3 | 27202.4 | 262 |
| B4FEW6 | Uncharacterized protein | 28768.6 | 262 |
| B6T8V1 | Uncharacterized protein | 29760.7 | 262 |
| B6TDS2 | Transcription factor APFI | 28105.7 | 262 |
| B6TIF9 | Nana plant1 | 29013.8 | 262 |
| B6TMG9 | Mitochondrial NADH ubiquinone oxidoreductase 29 kDa subunit | 27889.8 | 262 |
| B6UHM7 | Phosphate import ATP-binding protein pstB 1 | 27872 | 262 |
| K7UR36 | Alba DNA/RNA-binding protein | 29579.5 | 262 |
| K7V7L8 | Nuclear speckle RNA-binding protein A | 28232.3 | 262 |
| A0A1D6FPM5 | Protein LIKE COV 2 | 29107.6 | 263 |
| A0A1D6GIJ3 | Maf-like protein | 29238.5 | 263 |
| A0A317Y5M8 | 3-oxoacyl-[acyl-carrier-protein] reductase FabG | 27494.8 | 263 |
| B4FFM6 | Tropinone reductase-like protein | 27787.7 | 263 |
| B4FP70 | Small nuclear ribonucleoprotein-associated protein | 27655.3 | 263 |
| B4FUV9 | Uncharacterized protein | 29268 | 263 |
| B4G0M2 | S-adenosyl-L-methionine-dependent methyltransferase superfamily protein | 28326.2 | 263 |
| B6SMB8 | COV1-like protein | 29183.6 | 263 |
| B6SUU1 | Tropinone reductase 2 | 27734.8 | 263 |
| B6T9F7 | Proteasome subunit beta | 28796.5 | 263 |
| B6TB21 | Anamorsin homolog | 27487.1 | 263 |
| B6TFV4 | Mitochondrial glycoprotein family protein | 29578.7 | 263 |
| B6TQ65 | Proteasome subunit beta | 28882.5 | 263 |
| B8A1C0 | Proliferating cell nuclear antigen | 29342.3 | 263 |
| C0PBH7 | Uncharacterized protein | 27424.3 | 263 |
| K7USN8 | Gamma carbonic anhydrase 1 mitochondrial | 28419.1 | 263 |
| Q9SAQ3 | Proliferating cell nuclear antigen | 29313.3 | 263 |
| A0A1D6FTN0 | Peptidyl-tRNA hydrolase II (PTH2) family protein | 28254.4 | 264 |
| A0A1D6INR0 | Stress responsive protein | 29273.7 | 264 |
| B4F8F2 | Uncharacterized protein | 27923.3 | 264 |
| B4FY74 | Alpha/beta-Hydrolases superfamily protein | 28656.4 | 264 |
| B6U5K2 | Uncharacterized protein | 29056.9 | 264 |
| B6U7L3 | Acyl-protein thioesterase 1 | 28394.3 | 264 |
| A0A1D6E5P3 | Putative aquaporin TIP3-2 | 27463.7 | 265 |
| B4F7S5 | Copper ion binding | 29119.6 | 265 |
| B4FMI5 | 40S ribosomal protein S4 | 30016.8 | 265 |
| B4FYF7 | Expansin | 28450.1 | 265 |
| B6T2A6 | Stem-specific protein TSJT1 | 27961.4 | 265 |
| K7VEA3 | Embryonic cell protein 63 | 28374.3 | 265 |
| K7VTU7 | Expansin-B4 | 27549.8 | 265 |
| O22453 | 40S ribosomal protein S4 | 30171.2 | 265 |
| Q41844 | Zein | 28702.3 | 265 |
| A0A1D6E7Z0 | Plant UBX domain-containing protein 1 | 29955.7 | 266 |
| A0A1D6IPS9 | Acyl carrier protein | 28413.8 | 266 |
| A0A1D6KSA0 | Tonoplast intrinsic protein3 | 27361.4 | 266 |
| A0A317Y816 | Protein CutA 1, chloroplastic | 29369.2 | 266 |
| B4FRG1 | 14-3-3-like protein | 29314.3 | 266 |
| B6TDB2 | Ser/Thr-rich protein T10 in DGCR region | 29645.3 | 266 |
| B6TEL8 | Uncharacterized protein | 29338.7 | 266 |
| B6UH67 | Late embryogenesis abundant protein D-34 | 27177.1 | 266 |
| B6UHU4 | Acidic leucine-rich nuclear phosphoprotein 32 family member A | 28315.6 | 266 |
| C0P9Y6 | Chromophore lyase CRL chloroplastic | 30047.6 | 266 |
| C0PC41 | Uncharacterized protein | 29212.2 | 266 |
| C0PLU9 | Hypoxanthine phosphoribosyltransferase | 29085.1 | 266 |
| D1ME29 | Uncharacterized protein | 29644.4 | 266 |
| K4JBV1 | WHIRLY-type transcription factor (Fragment) | 29438.4 | 266 |
| A0A1D6HF43 | Expansin-B4 | 27714.9 | 267 |
| A0A1D6J1U1 | RNA-binding protein 8A | 29853.4 | 267 |
| A0A1D6KLF6 | Ribosomal protein S5 domain 2-like superfamily protein | 28945 | 267 |
| B4FQ73 | Uncharacterized protein | 30490.2 | 267 |
| B4FR70 | 26S proteasome non-ATPase regulatory subunit 8 homolog A | 30781 | 267 |
| B6SI09 | Aquaporin TIP3.1 | 27283.6 | 267 |
| B6TEX0 | Inositol-1-monophosphatase | 28863.7 | 267 |
| B6TI18 | Eukaryotic translation initiation factor 2 beta subunit | 30372.4 | 267 |
| B6TPW4 | Eukaryotic translation initiation factor 2 beta subunit | 30241.2 | 267 |
| B6UAQ1 | Protein YIF1A | 30387.3 | 267 |
| B8A235 | Uncharacterized protein | 29635.4 | 267 |
| C0P2Y2 | Thiamine pyrophosphokinase | 30340.4 | 267 |
| C0P3M0 | Uncharacterized protein | 26839.2 | 267 |
| C4J9T7 | Uncharacterized protein | 29224.7 | 267 |
| K7UH50 | Multisynthetase complex auxiliary component p43 | 28687.5 | 267 |
| Q9FQ98 | Glutathione S-transferase GST 41 | 28996.5 | 267 |
| A0A1D6H5M1 | Chalcone-flavonone isomerase family protein | 29039.9 | 268 |
| A0A1D6I6L8 | Fasciclin-like arabinogalactan protein 8 | 26696.1 | 268 |
| A0A1D6J4J9 | Serine/arginine-rich splicing factor RS31A | 31062.5 | 268 |
| A0A317YDV2 | Uncharacterized protein | 27932.6 | 268 |
| B4FHC1 | Uncharacterized protein | 29385.9 | 268 |
| B4FHI0 | Protein YIPF | 29719 | 268 |
| B4FT47 | Uncharacterized protein | 29523.6 | 268 |
| B6SUI9 | Thiamine pyrophosphokinase | 29038.7 | 268 |
| B6TWE1 | USP family protein | 28058.1 | 268 |
| K7U5A5 | 14-3-3-like protein | 29542.6 | 268 |
| A0A1D6FJV7 | RNase PH-like protein | 29600 | 269 |
| A0A1D6GD81 | NAD(P)-binding Rossmann-fold superfamily protein | 29053.2 | 269 |
| A0A1D6LCF1 | ABC transporter G family member 3 | 29462.4 | 269 |
| B4FDN9 | V-type proton ATPase subunit D | 29129.4 | 269 |
| B4FL38 | Haloacid dehalogenase-like hydrolase (HAD) superfamily protein | 29048.4 | 269 |
| B4FTK0 | Early nodulin-like protein 9 | 26415.1 | 269 |
| B6SUF4 | Fasciclin-like arabinogalactan protein 7 | 28212.3 | 269 |
| B6TH59 | Vacuolar ATP synthase subunit D 1 | 29087.4 | 269 |
| B8A280 | THO complex subunit 4C | 29523.8 | 269 |
| C0P8K0 | Uncharacterized protein | 28872.1 | 269 |
| C4JA36 | Binding partner of ACD11 1 | 28069.6 | 269 |
| P29023 | Endochitinase B (Fragment) | 28165.5 | 269 |
| A0A1D6G8G2 | Integral membrane HRF1 family protein | 30845.8 | 270 |
| A0A1D6HZ73 | Putative carbohydrate esterase | 28744.9 | 270 |
| A0A1D6MBX4 | Syntaxin-72 | 30176.9 | 270 |
| A0A1D6NZ51 | Expansin-B4 | 28053.4 | 270 |
| B4FCJ6 | Secretory carrier-associated membrane protein | 30286.8 | 270 |
| B6SGQ2 | SAM domain family protein | 28317.4 | 270 |
| B6TK42 | Syntaxin 72 | 30204.9 | 270 |
| B6U036 | Alcohol dehydrogenases | 29018.1 | 270 |
| K7VJL9 | Senescence-associated protein DH | 29666.2 | 270 |
| K7VXI7 | Haloacid dehalogenase-like hydrolase (HAD) superfamily protein | 30523.6 | 270 |
| A0A075T7D0 | Pyrabactin resistance-like protein | 30064.2 | 271 |
| A0A317YJZ5 | Proteasome assembly chaperone 2 | 29472 | 271 |
| B4FRX2 | ACT domain-containing protein ACR12 | 30035 | 271 |
| B4FXA2 | Haloacid dehalogenase-like hydrolase domain-containing protein | 29719.8 | 271 |
| B4FZE8 | Fasciclin-like arabinogalactan protein 2 | 27371.8 | 271 |
| B6T3E6 | Uncharacterized protein | 28386.5 | 271 |
| K7URL2 | Cyclase | 29181.1 | 271 |
| Q94FZ7 | Granule-bound starch synthase (Fragment) | 29118.7 | 271 |
| A0A1D6K750 | Glutathione transferase37 | 30151.6 | 272 |
| A0A1D6KJL2 | Oligopeptide transporter-like protein | 29163.9 | 272 |
| A0A1D6ML28 | Uncharacterized protein | 29288.7 | 272 |
| A0A317YA71 | Putative glutathione S-transferase GSTU6 | 30111.5 | 272 |
| B4F892 | Cytochrome b-c1 complex subunit Rieske, mitochondrial | 29683.6 | 272 |
| B4FK71 | ER lumen protein retaining receptor C28H8.4 | 30450 | 272 |
| B6SGC3 | Histone H2B | 29087.7 | 272 |
| B6T3J2 | Eukaryotic translation initiation factor 2 beta subunit | 30695.7 | 272 |
| B6T731 | Myosin-like protein | 30638.4 | 272 |
| B6TFF7 | Replication factor A | 28990.3 | 272 |
| B6TG63 | Proteasome subunit beta | 29247.2 | 272 |
| K7TFB6 | ABA-responsive protein | 29174.8 | 272 |
| K7W0L3 | Uncharacterized protein | 28521.4 | 272 |
| A0A1D6NRY6 | Polyadenylate-binding protein 3 | 30701.1 | 273 |
| B4F8L2 | Uncharacterized protein | 30583 | 273 |
| B4FBQ8 | Cytochrome b-c1 complex subunit Rieske, mitochondrial | 29667.5 | 273 |
| B4FC14 | Cytochrome b-c1 complex subunit Rieske, mitochondrial | 29843.7 | 273 |
| B4FJY5 | Proteasome subunit beta | 29305.2 | 273 |
| B6SSV3 | Fasciclin-like arabinogalactan protein 7 | 28107.7 | 273 |
| B6TJM6 | KID-containing protein | 29091.8 | 273 |
| B6TNP4 | Histone H1 | 27901.1 | 273 |
| B6TUI0 | Estradiol 17-beta-dehydrogenase 8 | 27953.4 | 273 |
| B6TWB2 | ACT domain containing protein | 30258.3 | 273 |
| C0PB60 | Gamma carbonic anhydrase 2 mitochondrial | 29643.7 | 273 |
| C0PIA2 | Nuclear cap-binding protein subunit 2 | 31624.5 | 273 |
| A0A1D6DZK3 | Uncharacterized protein | 31607.9 | 274 |
| A0A1D6GF42 | 40S ribosomal protein S2-1 | 29817.6 | 274 |
| A0A1D6K4Q9 | Uncharacterized protein | 28669.6 | 274 |
| A0A1D6L7U4 | Tryptophan synthase A homolog1 | 28996.4 | 274 |
| A0A1D6MS44 | Serine/arginine-rich-splicing factor SR34 | 31442.5 | 274 |
| A0A1D6NW49 | Histone H1 | 29105.5 | 274 |
| A0A1D6NWE4 | Nuclear speckle RNA-binding protein A | 29501.6 | 274 |
| B4FJ38 | Mitochondrial glycoprotein family protein | 30028.3 | 274 |
| B4FRQ1 | Ribulose-phosphate 3-epimerase | 28933.2 | 274 |
| B6T2X9 | Low molecular weight protein-tyrosine-phosphatase slr0328 | 29096.7 | 274 |
| B6TAJ3 | Proteasome subunit alpha type | 30015.5 | 274 |
| B6TFR7 | Chalcone isomerase | 29236.2 | 274 |
| B6TS31 | Indole-3-glycerol phosphate lyase | 29010.4 | 274 |
| C0PCQ6 | 40S ribosomal protein S2-1 | 29935.7 | 274 |
| C0PPC8 | Ribulose-phosphate 3-epimerase | 29003.2 | 274 |
| G4XT43 | Prolyl 4-hydroxylase 4 | 30502.7 | 274 |
| K7UBM6 | DNA binding protein isoform 1 | 29619.4 | 274 |
| A0A1D6EAQ0 | GrpE protein homolog | 30088.1 | 275 |
| A0A1D6ED43 | Xanthoxin dehydrogenase | 27916.4 | 275 |
| A0A1D6I966 | Uncharacterized protein | 29141.6 | 275 |
| A0A1D6IL23 | Proteasome subunit alpha type | 29955 | 275 |
| B4F7Y5 | Acid phosphatase 1 | 29578.7 | 275 |
| B6SI10 | Uncharacterized protein | 29630.1 | 275 |
| B6SLB7 | Uncharacterized protein | 29151.4 | 275 |
| B6SLD1 | Outer plastidial membrane protein porin | 29779.3 | 275 |
| B6SS48 | Outer mitochondrial membrane protein porin | 29151.9 | 275 |
| B6U9C1 | Outer plastidial membrane protein porin | 29776.3 | 275 |
| C0PIL8 | Tobamovirus multiplication protein 2A | 30217.7 | 275 |
| C0PMJ3 | Gamma-glutamyl peptidase 1 | 29980.9 | 275 |
| C4IYM7 | Mitochondrial outer membrane protein porin 2 | 29183 | 275 |
| C4JAI3 | Cyclase family protein | 29437.4 | 275 |
| K7VJ77 | Porin1 | 29776.3 | 275 |
| A0A1D6I2X6 | Voltage-dependent anion channel protein1b | 29662.3 | 276 |
| A0A1D6Q191 | Uncharacterized protein | 29719.1 | 276 |
| A0A1D6QEM7 | Expansin | 28707 | 276 |
| B4F886 | Cupin, RmlC-type | 30060.8 | 276 |
| B4FT85 | Isochorismate synthase 1 | 29470.1 | 276 |
| B6SUS7 | Outer mitochondrial membrane protein porin | 29699.3 | 276 |
| B6TAR9 | Outer mitochondrial membrane protein porin | 29549 | 276 |
| B8A2G7 | Alba DNA/RNA-binding protein | 30981 | 276 |
| B8QUW4 | Chitinase | 29079.4 | 276 |
| Q9SPD7 | Outer mitochondrial membrane protein porin | 29177.6 | 276 |
| Q9SPD8 | Voltage-dependent anion channel protein 1b | 29648.3 | 276 |
| A0A1D6LN76 | Peptidyl-prolyl cis-trans isomerase | 30501.9 | 277 |
| A0A1D6QHJ2 | Uncharacterized protein | 32000.2 | 277 |
| A0A1X7YE61 | Uncharacterized protein | 30256.4 | 277 |
| A0A317YJ37 | Stem-specific protein TSJT1 | 29453.1 | 277 |
| B4FTM3 | (DL)-glycerol-3-phosphatase 2 | 30218.6 | 277 |
| B6TJ91 | Splicing factor, arginine/serine-rich 7 | 31316.3 | 277 |
| B6TNX7 | Proteasome subunit alpha type | 30315.7 | 277 |
| B6TRZ9 | FIP1 | 29628.2 | 277 |
| B7ZZK0 | Uncharacterized protein | 30600.3 | 277 |
| B8QUX1 | Chitinase | 29031.3 | 277 |
| A0A1D6HAW8 | Glutathione S-transferase Z1 | 30195.1 | 278 |
| A0A1D6JLC2 | Cell division control protein2-like protein | 31710.2 | 278 |
| A0A1D6JTL2 | Caleosin | 31192.9 | 278 |
| A0A317Y9K9 | GDT1-like protein 5 | 30174.9 | 278 |
| B6STQ5 | Urease accessory protein G | 29934.3 | 278 |
| B6TGK1 | Ribonuclease 2 | 30868 | 278 |
| B6TML1 | HemK methyltransferase family member 2 | 29892.7 | 278 |
| B6U0D3 | Uncharacterized protein | 29666.3 | 278 |
| B8QUZ0 | Chitinase | 29162.4 | 278 |
| D0EM57 | Chitinase | 29254.6 | 278 |
| K7UGQ0 | Uncharacterized conserved protein UCP022280 | 29735.4 | 278 |
| B4F808 | KH domain-containing protein SPIN1 | 31128.1 | 279 |
| B4FG48 | CCR4-NOT transcription complex subunit 7 | 30784.7 | 279 |
| B6SU77 | Arginine/serine-rich splicing factor RSP41 | 31815.8 | 279 |
| B6T6T8 | Cysteine sulfinate desulfinase/cysteine desulfurase and related enzymes | 30547.2 | 279 |
| B6TJS9 | Sec20 family protein | 30832.7 | 279 |
| B6TNR8 | 40S ribosomal protein S2 | 30357.1 | 279 |
| B6U7J5 | Cysteine sulfinate desulfinase/cysteine desulfurase and related enzymes | 30490.2 | 279 |
| K7V2C1 | Aluminum induced protein with YGL and LRDR motifs | 29299.5 | 279 |
| Q6JBL0 | Chitinase | 29165.4 | 279 |
| Q6JBP8 | Chitinase | 28925.3 | 279 |
| Q8W2K4 | NADH-cytochrome b5 reductase | 30991.7 | 279 |
| Q9ZPN0 | NADH-cytochrome b5 reductase | 31171.8 | 279 |
| A0A1D6F2I0 | Vesicle transport protein | 29616.7 | 280 |
| A0A1D6I1V0 | Polyadenylate-binding protein-interacting protein 9 | 30834.7 | 280 |
| A0A1D6KQT9 | Trihelix transcription factor GT-2 | 31057.3 | 280 |
| A0A1D6MFY7 | Isoamyl acetate-hydrolyzing esterase | 30648.5 | 280 |
| B4FI86 | Proteasome subunit beta | 30153.8 | 280 |
| B4FSY2 | Transformer-2 protein | 32128.9 | 280 |
| B4FTL2 | Protein TIC 22 chloroplastic | 30080.4 | 280 |
| B4FTS6 | Endochitinase A | 29211.5 | 280 |
| B6U3B8 | Protein-L-isoaspartate O-methyltransferase | 30211.5 | 280 |
| B8QUS8 | Chitinase | 29289.5 | 280 |
| Q6JBQ2 | Chitinase | 28963.4 | 280 |
| A0A1D6F918 | 40S ribosomal protein S15-4 | 31603.9 | 281 |
| A0A1D6FKZ4 | 60S acidic ribosomal protein P2-5 | 30124.8 | 281 |
| A0A1D6GI39 | E3 ubiquitin-protein ligase HOS1 | 30547.8 | 281 |
| A0A1D6PD99 | Glutathione S-transferase L2 chloroplastic | 31696.7 | 281 |
| B4FN92 | KH domain-containing protein SPIN1 | 31522.7 | 281 |
| B4FNZ8 | KH domain-containing protein SPIN1 | 31505.7 | 281 |
| B6TAB7 | SCO1 protein | 31586.7 | 281 |
| B6TKC3 | Protein CHAPERONE-LIKE PROTEIN OF POR1 chloroplastic | 31492.3 | 281 |
| B6TKL0 | Proteasome assembly chaperone 2 | 30494.1 | 281 |
| B6TS08 | ATP-dependent Clp protease proteolytic subunit | 30430.6 | 281 |
| B6U0H2 | 6-phosphogluconolactonase | 31324.8 | 281 |
| C0PEC6 | Uncharacterized protein | 30584.6 | 281 |
| A0A1D6H2K9 | Chorismate mutase2 | 31557.9 | 282 |
| A0A1D6IEQ6 | USP family protein | 29615 | 282 |
| A0A1D6N0R7 | Putative inactive leucine-rich repeat receptor-like protein kinase | 29732.3 | 282 |
| A0A317YGC0 | Expansin-B4 | 31165.9 | 282 |
| B4FVB4 | Phytanoyl-CoA dioxygenase | 31683.4 | 282 |
| B6SGJ3 | Membrane protein | 30135.8 | 282 |
| B6T2R9 | Anthranilate synthase component II | 30253.2 | 282 |
| B6T7A4 | Glyceraldehyde-3-phosphate dehydrogenase | 30354.4 | 282 |
| B6TA72 | Mitochondrial prohibitin complex protein 1 | 30586.6 | 282 |
| B6TWI3 | HD domain containing protein | 30821 | 282 |
| C0PKN5 | Endochitinase EP3 | 29422.7 | 282 |
| Q9M587 | Prohibitin | 30598.6 | 282 |
| A0A1D6DUL4 | Transport protein particle (TRAPP) component | 30848.9 | 283 |
| A0A1D6MF83 | Reticulon-like protein | 31045.6 | 283 |
| A0A1D6MFY8 | Isoamyl acetate-hydrolyzing esterase | 31064.8 | 283 |
| A0A1D6P348 | F-box protein SKIP19 | 32288.5 | 283 |
| A0A1D6Q078 | Uncharacterized protein | 29821.1 | 283 |
| A0A317Y935 | Uncharacterized protein | 32299.5 | 283 |
| B6TKU7 | Sex determination protein tasselseed-2 | 29349.1 | 283 |
| Q6JBQ4 | Chitinase | 29404.9 | 283 |
| A0A1D6GI55 | GLABRA2 expression modulator | 30833.7 | 284 |
| A0A1D6LBZ9 | Inactive protein FRIGIDA | 31528.5 | 284 |
| B4FAP3 | NAD(P)-binding Rossmann-fold superfamily protein | 30821.1 | 284 |
| B4FAZ6 | Adaptin ear-binding coat-associated protein 2 | 30433.9 | 284 |
| B4FIZ5 | Clathrin light chain | 30323 | 284 |
| B4FMZ0 | Uncharacterized protein | 30294.8 | 284 |
| B6STL7 | Adenylate kinase | 31138.3 | 284 |
| B6T3N1 | Plant UBX domain-containing protein 1 | 31938.7 | 284 |
| B6TDI2 | F-box domain containing protein | 31216.3 | 284 |
| C0PAK6 | Nuclear pore complex protein NUP62 | 31348.6 | 284 |
| Q9M584 | Hypersensitive-induced response protein | 31366.5 | 284 |
| Q9M588 | Prohibitin2 | 30720.8 | 284 |
| A0A1D6HKT9 | Polyadenylate-binding protein 3 | 31949.7 | 285 |
| A0A1D6PXQ1 | RanBP2-type zinc finger protein | 30622.9 | 285 |
| B4FJF2 | Protein TIC 22-like chloroplastic | 31028.8 | 285 |
| B4FL94 | Protein TIC 21 chloroplastic | 29653.3 | 285 |
| B6TA10 | Uncharacterized protein | 29706.4 | 285 |
| B6TDC9 | Heme oxygenase 1 | 31491.4 | 285 |
| K7V687 | Histone deacetylase HDT1 | 30434.9 | 285 |
| Q64HC3 | ASF/SF2-like pre-mRNA splicing factor SRP32 | 31637.5 | 285 |
| V5JTN5 | Heme oxygenase 1 | 31566.5 | 285 |
| A0A1D6HE36 | Replication protein A 32 kDa subunit A | 30644.3 | 286 |
| A0A1D6KJW9 | 5'-methylthioadenosine/S-adenosylhomocysteine nucleosidase 1 | 30117.6 | 286 |
| A0A1D6NVY0 | Glycine-rich protein | 31518.1 | 286 |
| B4FBN8 | Chitinase 1 | 31127.8 | 286 |
| B4FTH5 | Xyloglucan endotransglucosylase/hydrolase | 31975.5 | 286 |
| B6T8Z1 | Nucleolar essential protein-related | 31801.3 | 286 |
| B6TBK4 | Ribonucleoprotein | 31231.8 | 286 |
| Q94KS6 | Beta expansin8 | 31626.4 | 286 |
| A0A1D6LT87 | Ras-related protein RABA1d | 31567 | 287 |
| A0A1D6NE71 | RNA-binding KH domain-containing protein | 33099.2 | 287 |
| B4FBV8 | Eukaryotic translation initiation factor 3 subunit G | 31662.3 | 287 |
| B4FRD9 | Eukaryotic translation initiation factor 3 subunit F | 31463.3 | 287 |
| B6T4R9 | Exosome complex exonuclease RRP42 | 30157.8 | 287 |
| B6T4T1 | Peroxisome biogenesis protein 22 | 31747.9 | 287 |
| B6TKH5 | 26S proteasome non-ATPase regulatory subunit 14 | 32268.1 | 287 |
| C0P4M1 | Putative rRNA-processing protein EBP2-like protein | 32939.7 | 287 |
| K7V686 | Eukaryotic translation initiation factor 3 subunit F | 31360.2 | 287 |
| Q4TZJ2 | Pyrroline-5-carboxylate reductase | 29732 | 287 |
| Q9M582 | Hypersensitive induced reaction3 | 31553.8 | 287 |
| Q9M639 | Coatomer subunit epsilon | 31408.6 | 287 |
| A0A1D6L7E0 | 25.3 kDa vesicle transport protein | 32659.5 | 288 |
| A0A1D6PEU1 | ATP-dependent Clp protease ATP-binding subunit CLPT1 chloroplastic | 31237.7 | 288 |
| B4FD65 | Delta3,5-delta2,4-dienoyl-CoA isomerase | 30168.4 | 288 |
| B4FNP2 | Phosphoribosylglycinamide formyltransferase chloroplastic | 30705.9 | 288 |
| B6SK72 | Alpha-soluble NSF attachment protein | 32142.6 | 288 |
| B6SQQ0 | Inorganic pyrophosphatase | 31736.7 | 288 |
| B6TTQ8 | Hypersensitive-induced reaction protein 4 | 32075.5 | 288 |
| Q9SE92 | Adenylyl-sulfate kinase (Fragment) | 31611.9 | 288 |
| A0A1D6FDV7 | Protein ABCI7 chloroplastic | 31090.8 | 289 |
| A0A1D6NV82 | Proteasome subunit beta | 30985.7 | 289 |
| B4FNF5 | ATP-dependent Clp protease proteolytic subunit | 31864.8 | 289 |
| B4FRD1 | Adaptin ear-binding coat-associated protein 1 NECAP-1 | 32077.8 | 289 |
| B4FRX8 | Uncharacterized protein | 31659.7 | 289 |
| B4FZQ7 | Casein kinase II subunit beta | 32432 | 289 |
| B6SIS9 | Inositol polyphosphate multikinase | 30784.8 | 289 |
| B6TBM1 | Alpha-soluble NSF attachment protein 2 | 32396.9 | 289 |
| B7ZXI0 | Uncharacterized protein | 30839.5 | 289 |
| Q9M586 | Prohibitin | 31887.5 | 289 |
| Q9M589 | Prohibitin1 | 32078.6 | 289 |
| A0A1D6EGG2 | 60 kDa jasmonate-induced protein | 31137.6 | 290 |
| A0A1D6ELB0 | G-box-binding factor 1 | 30613.2 | 290 |
| A0A1D6JYM6 | Safener induced1 | 32548.7 | 290 |
| B4F7V3 | Protein phosphatase 2C isoform epsilon | 31734.8 | 290 |
| B4FR28 | Uracil phosphoribosyltransferase chloroplastic | 31249.2 | 290 |
| B6T2Y5 | S-formylglutathione hydrolase | 31885.7 | 290 |
| B6T634 | Aquaporin PIP2.1 | 30213.6 | 290 |
| B6TQS8 | Uncharacterized protein | 30230 | 290 |
| B6TR18 | Aquaporin PIP2.1 | 30273.6 | 290 |
| B6TWT7 | Uncharacterized protein | 32500.2 | 290 |
| C0PN65 | TIP41-like family protein | 32115.5 | 290 |
| Q6IV73 | Protein phosphatase 2C | 31193.2 | 290 |
| A0A1D6GN34 | Putative plastid-lipid-associated protein 10 chloroplastic | 31541.9 | 291 |
| A0A1D6JP57 | Rab28 protein | 28407.7 | 291 |
| A0A1D6L911 | Protein CDI | 32756.6 | 291 |
| A0A1D6LGF1 | Gamma-glutamylcysteine synthetase1 | 34006.8 | 291 |
| B4F7V1 | 2-dehydro-3-deoxyphosphooctonate aldolase 2 | 31746.7 | 291 |
| B4FNY2 | Translin family protein | 32261.1 | 291 |
| B4FY31 | Chloroplast J-like domain 1 | 32454.4 | 291 |
| B6T9M5 | Translin | 32291.1 | 291 |
| B6TN77 | Dehydrin COR410 | 31497.7 | 291 |
| C0P3I5 | Late embryogenesis abundant (LEA) hydroxyproline-rich glycoprotein family | 30207.1 | 291 |
| A0A1D6HGR9 | Secretory carrier-associated membrane protein | 32936.8 | 292 |
| B4FH10 | Bifunctional monothiol glutaredoxin-S16 chloroplastic | 31341.5 | 292 |
| B4FH88 | Bifunctional protein FolD 2 | 30580.4 | 292 |
| B6TS83 | Aquaporin PIP2-2 | 30259.7 | 292 |
| B6TX73 | ATP-dependent Clp protease proteolytic subunit | 31803.6 | 292 |
| C0HIP0 | Uncharacterized protein | 31505.5 | 292 |
| K7VKR9 | Strigolactone esterase D14 | 31563.9 | 292 |
| A0A1D6NJT2 | Uncharacterized protein | 31982.1 | 293 |
| A0A1D6PJW1 | Proteasome subunit alpha type | 31493.1 | 293 |
| B4F8E9 | Uncharacterized protein | 31790.3 | 293 |
| B4FKR4 | Ankyrin repeat family protein | 31413.1 | 293 |
| B6TNA7 | Shikimate kinase | 31843.4 | 293 |
| B6U3A0 | Glycine-rich RNA-binding protein 7 | 31476.4 | 293 |
| A0A1D6IJ81 | Uncharacterized protein | 31735 | 294 |
| A0A317YA84 | Anthranilate synthase beta subunit 2, chloroplastic | 33102.8 | 294 |
| B4FMY8 | Tobamovirus multiplication 3 | 33764 | 294 |
| B4FRC8 | FAD/NAD(P)-binding oxidoreductase | 31605.7 | 294 |
| B4FX07 | Uncharacterized protein | 31130.7 | 294 |
| B6TA31 | Fruit protein PKIWI502 | 31459.6 | 294 |
| B6TWY3 | Cell division control protein 2 | 33891.9 | 294 |
| B6U5X4 | F-box domain containing protein | 32703.4 | 294 |
| C0HEL7 | Protein -9-like protein | 33646.7 | 294 |
| C0PDV0 | Tetraspanin-3 | 32780.2 | 294 |
| K7UY34 | Tetraspanin-3 | 32854.2 | 294 |
| P93518 | PRm 3 | 30118.1 | 294 |
| A0A1D6I1U8 | Polyadenylate-binding protein-interacting protein 9 | 32192.2 | 295 |
| B4FJ98 | Uncharacterized protein | 31528.1 | 295 |
| B4FUG2 | Gamma-soluble NSF attachment protein | 33033.5 | 295 |
| B6TZU6 | Pirin-like protein | 32344.2 | 295 |
| C0HF37 | Uncharacterized protein | 33060.2 | 295 |
| C0P648 | Uncharacterized protein | 32417.2 | 295 |
| C4JA00 | RNA-binding (RRM/RBD/RNP motifs) family protein | 32692.8 | 295 |
| K7UFU7 | 2-hydroxy-3-oxopropionate reductase | 30628.3 | 295 |
| A0A1D6GF60 | Methyltransferase | 32447.1 | 296 |
| A0A1D6H2K7 | Nucleoid-associated protein chloroplastic | 32269.1 | 296 |
| A0A1D6L1H9 | Oxysterol-binding protein-related protein 1C | 31870.9 | 296 |
| A0A1D6MY31 | Glutathione transferase18 | 33545.5 | 296 |
| A0A317YCN0 | Glutathione S-transferase F10 | 33384.9 | 296 |
| B4FEA2 | Mitochondrial carnitine/acylcarnitine carrier-like protein | 30155.7 | 296 |
| B6TF90 | SNF1 protein kinase regulatory subunit beta-1 | 31959.4 | 296 |
| B6U0C2 | Phenazine biosynthesis PhzC/PhzF protein | 31491.7 | 296 |
| K7U915 | Phosphoserine phosphatase chloroplastic | 31461.7 | 296 |
| K7V294 | Putative calcium-binding protein CML50 | 31119.6 | 296 |
| K7VFM9 | Isopentenyl pyrophosphate isomerase2 | 33133.7 | 296 |
| A0A1D6L7R5 | Tryptophan synthase alpha chain chloroplastic | 31615.5 | 297 |
| A0A1D6MEN7 | Glycosyltransferases | 33405.9 | 297 |
| A0A1D6PNQ5 | WD repeat-containing protein VIP3 | 31196 | 297 |
| B4F9A5 | Uncharacterized protein | 30709.1 | 297 |
| B4FC49 | Uncharacterized protein | 32211.3 | 297 |
| B4FND2 | Binding | 32577 | 297 |
| B6SU38 | Phosphoserine phosphatase | 31516.7 | 297 |
| B6SZV0 | Nucleic acid binding protein | 31638.2 | 297 |
| B6T9D2 | Repressor protein | 33551.7 | 297 |
| B6TAE6 | Uncharacterized protein | 32149.3 | 297 |
| B6THZ2 | Delay of germination 1 | 31758.4 | 297 |
| A0A1D6DTQ0 | Uncharacterized protein | 30551.6 | 298 |
| A0A1D6LFH1 | Syntaxin-132 | 31028.9 | 298 |
| A0A1Q1BT41 | CMP-KDO synthetase1 | 32578.2 | 298 |
| B4FDF4 | Uncharacterized protein | 31698.9 | 298 |
| B4FH62 | NAD(P)-binding Rossmann-fold superfamily protein | 31822.1 | 298 |
| B4FP01 | Diphosphocytidyl methyl erythritol synthase1 | 32905.9 | 298 |
| B6TDF2 | Riboflavin synthase alpha chain | 30807 | 298 |
| B6TED5 | Prolyl 4-hydroxylase | 32897.9 | 298 |
| A0A1D6DT47 | Clathrin light chain | 32385.6 | 299 |
| A0A1D6KPB1 | Gibberellin 20 oxidase 2 | 32358.3 | 299 |
| B4F890 | 3-hydroxybutyryl-CoA dehydrogenase | 31670.4 | 299 |
| B4FQL2 | SEC13-related protein | 32194.2 | 299 |
| B6TCN5 | Nudix type motif 22 | 33286.9 | 299 |
| B6THJ5 | Phosphosulfolactate synthase-related protein | 33704.9 | 299 |
| B6TQ27 | Uncharacterized protein | 31524.9 | 299 |
| B6TV54 | Acid phosphatase 1 | 32726.3 | 299 |
| B6U310 | 3-hydroxybutyryl-CoA dehydrogenase | 31685.4 | 299 |
| C0HEG7 | Putative plastid-lipid-associated protein 13 chloroplastic | 32776.2 | 299 |
| K0DCQ3 | C3H4 C3H transcription factor (Fragment) | 34540.8 | 299 |
| K7V902 | Histidine biosynthesis protein hisIE | 32663.5 | 299 |
| Q84JX6 | Phosphoglycerate kinase (Fragment) | 31625.3 | 299 |
| A0A1D6F2Q2 | Transmembrane protein 115 | 33637.2 | 300 |
| B4FFN2 | Flavonol synthase/flavanone 3-hydroxylase | 32503.4 | 300 |
| B4FN73 | Putative 2-oxoglutarate-dependent dioxygenase AOP1 | 32327.3 | 300 |
| B4FWY5 | BSD domain-containing protein | 32996.4 | 300 |
| B6SK06 | Histone deacetylase 2b | 32501.2 | 300 |
| B6TKZ3 | Hydrolase, carbon-nitrogen family | 33475.8 | 300 |
| B8A2C6 | Uncharacterized protein | 33461.4 | 300 |
| A0A1D6I1S7 | Protein SAWADEE HOMEODOMAIN HOMOLOG 2 | 33581.4 | 301 |
| A0A1D6IQ00 | THAUMATIN-LIKE PROTEIN 1 | 30739 | 301 |
| B4FNN7 | Protein KTI12-like protein | 33632 | 301 |
| B4FR99 | Acidic endochitinase | 32535.4 | 301 |
| B6SL69 | Uncharacterized protein | 31999.4 | 301 |
| B6TE60 | Putative proteasome inhibitor | 31554 | 301 |
| B6TIB0 | Uncharacterized protein | 31565 | 301 |
| B6UFK5 | Uncharacterized protein | 33690.9 | 301 |
| K4JRV1 | C3H-type transcription factor (Fragment) | 31253.4 | 301 |
| K7W7E9 | Transferase | 31181.1 | 301 |
| P28522 | Ribosome-inactivating protein | 33329.9 | 301 |
| Q2XXG4 | Ribosome inactivating protein 1 (Fragment) | 33329.8 | 301 |
| Q41848 | 36.4 kDa proline-rich protein | 31646.8 | 301 |
| A0A1D6EWI5 | Casein kinase II subunit beta | 33945.8 | 302 |
| A0A1D6HNJ8 | Plastid transcriptionally active 17 | 34508.8 | 302 |
| A0A1D6I8E8 | Ankyrin repeat domain-containing protein 2A | 32987.8 | 302 |
| A0A1D6IY08 | Zinc ion binding | 34093.9 | 302 |
| A0A1D6KRT5 | Putative prolyl 4-hydroxylase 3 | 33453.7 | 302 |
| A0A1R3N7W5 | Floury endosperm1 | 32585.5 | 302 |
| A0A317YF55 | Phosphoglycerate mutase-like protein 4 | 31937.9 | 302 |
| B4FQA8 | Methylglutaconyl-CoA hydratase | 32385.2 | 302 |
| B4G0G4 | Protein BOBBER 1 | 33665.1 | 302 |
| B6T9B6 | Peroxisomal 2,4-dienoyl-CoA reductase | 32211.8 | 302 |
| B6THG9 | 60S ribosomal protein L5-1 | 34425.6 | 302 |
| B6TKI4 | MAPK activating protein | 34553.9 | 302 |
| B6TVP3 | Phloem-specific lectin | 33480.7 | 302 |
| C4JA45 | 60S ribosomal protein L5-1 homolog b | 34377.5 | 302 |
| K7W104 | 2-methoxy-6-polyprenyl-1,4-benzoquinol methylase, mitochondrial | 33793.3 | 302 |
| A0A1D6JNW2 | L-ornithine N5-acetyltransferase NATA1 | 32904.1 | 303 |
| B4FHA8 | Histone deacetylase102 | 32587.1 | 303 |
| B4FLG1 | RNA-binding (RRM/RBD/RNP motifs) family protein | 33039.8 | 303 |
| B4FN51 | GrpE protein homolog | 33147 | 303 |
| B4FT76 | ABC transporter I family member 6 chloroplastic | 32360.7 | 303 |
| B4FXR6 | Uncharacterized protein | 29033.3 | 303 |
| B6SNP7 | Eyes absent-like protein | 34187.8 | 303 |
| B6TES2 | Uncharacterized protein | 33975.9 | 303 |
| B7ZXE0 | Uncharacterized protein | 34615 | 303 |
| C0PAS3 | Serine/threonine-protein phosphatase | 34754.2 | 303 |
| C4J9C7 | EYES ABSENT-like protein | 34153.8 | 303 |
| A0A1D6LJ95 | Phytoene synthase | 33647.4 | 304 |
| A0A317Y3J1 | Methyltransferase-like protein 13 (Fragment) | 33851.1 | 304 |
| B4F820 | Uncharacterized protein | 32480.7 | 304 |
| B4FT16 | Vacuolar protein sorting 26 | 35333.2 | 304 |
| B4FTJ0 | Triosephosphate isomerase | 32420.6 | 304 |
| B4FUS5 | ATP-dependent Clp protease proteolytic subunit | 32172.3 | 304 |
| B6SML6 | NHL25 | 32379.7 | 304 |
| B6TB83 | Vesicle-associated membrane protein 725 | 33928.2 | 304 |
| B6TDM3 | Syntaxin 132 | 34566.3 | 304 |
| B6TVF0 | GPN-loop GTPase 2 | 34523.2 | 304 |
| B6UEZ8 | Vacuolar protein sorting 26 | 35456.5 | 304 |
| B6UG30 | Triosephosphate isomerase | 32392.6 | 304 |
| K7V1E2 | C3H transcription factor | 35276.6 | 304 |
| A0A097PRW4 | Ankyrin repeat family protein (Fragment) | 33746.1 | 305 |
| A0A317YGT7 | Protein SRG1 | 33187.4 | 305 |
| B4FH50 | Lipid phosphate phosphatase 3 | 34383.5 | 305 |
| B4FI49 | 5'-AMP-activated protein kinase-related | 34155.4 | 305 |
| B4FJF0 | Clathrin light chain | 32847.3 | 305 |
| B4FV03 | Glutathione transferase11 | 34117 | 305 |
| B6SGN7 | Embryonic protein DC-8 | 32277.5 | 305 |
| B6TMN3 | SEC13 protein isoform 1 | 33228.1 | 305 |
| B6TXQ4 | Glutathione S-transferase, C-terminal domain containing protein | 34175 | 305 |
| B6UAJ5 | Methylglutaconyl-CoA hydratase | 32427 | 305 |
| B6UBG2 | Protein transport protein SEC13 homolog B | 33140 | 305 |
| B6UI48 | 2-methoxy-6-polyprenyl-1,4-benzoquinol methylase, mitochondrial | 33818.4 | 305 |
| A0A1D6EX05 | Uncharacterized protein | 33342 | 306 |
| A0A1D6EZJ7 | 5-formyltetrahydrofolate cyclo-ligase mitochondrial | 33888.8 | 306 |
| A0A1D6FC20 | Syntaxin-22 | 33470.3 | 306 |
| A0A1D6LSM6 | Serine/threonine-protein phosphatase | 35164.6 | 306 |
| A0A1D6N8X4 | Cytochrome b5 reductase | 34471.7 | 306 |
| A0A1D6Q9G4 | BSD domain-containing protein | 33170.3 | 306 |
| A0A1D6QBR6 | Oxidoreductase zinc-binding dehydrogenase family protein | 31731.3 | 306 |
| B4FDY3 | 3'-5'-exoribonuclease family protein | 32836.4 | 306 |
| B4FLN7 | PUR ALPHA-1 | 33405.5 | 306 |
| B4FQJ0 | Serine/threonine-protein phosphatase | 35157.5 | 306 |
| B6SS56 | Isomerase | 33464.1 | 306 |
| B6TV09 | 3-beta hydroxysteroid dehydrogenase/isomerase family protein | 32621.1 | 306 |
| B6U122 | F-actin capping protein alpha subunit | 34173.8 | 306 |
| B6UB37 | Zinc finger C-x8-C-x5-C-x3-H type family protein | 31820.8 | 306 |
| C0HED5 | Putative transcriptional regulatory protein | 33571.7 | 306 |
| C0HIG1 | Uncharacterized protein | 32982.7 | 306 |
| A0A1D6EPD2 | Succinate dehydrogenase [ubiquinone] iron-sulfur subunit 3 mitochondrial | 33835.4 | 307 |
| A0A1D6HTN8 | Xylanase inhibitor protein 1 | 32992.2 | 307 |
| A0A1D6LK73 | Peroxisomal membrane protein 11C | 34101 | 307 |
| A0A1D6NN07 | DNA-damage-repair/toleration protein DRT102 | 32989.8 | 307 |
| A0A1D6P7V2 | 5-hydroxyisourate hydrolase | 33569.4 | 307 |
| A0A1R3R097 | Ninja-family protein 5 | 32223.8 | 307 |
| B4F7T4 | Protein SCO1 homolog 1 mitochondrial | 33280.8 | 307 |
| B4FEP9 | Prolyl 4-hydroxylase 1 | 33232.5 | 307 |
| B4FP86 | DDRGK domain-containing protein 1 | 34780.3 | 307 |
| B4FTA4 | Uricase | 34437.1 | 307 |
| B4FZ84 | 40S ribosomal protein SA | 33028.1 | 307 |
| B4G019 | Hydroxyproline-rich glycoprotein family protein | 33358.2 | 307 |
| B6SSF3 | 40S ribosomal protein SA | 32984 | 307 |
| B6T4R7 | Serine/threonine-protein phosphatase | 35189.5 | 307 |
| B6TRW7 | Sigma factor sigB regulation protein rsbQ | 32676 | 307 |
| B6TS48 | HAD-superfamily hydrolase, subfamily IIA | 32771.2 | 307 |
| B6U607 | Carbonyl reductase 1 | 32682 | 307 |
| G4XT60 | Prolyl 4-hydroxylase 8-5 | 33773.3 | 307 |
| K4JCC8 | C3H-type transcription factor (Fragment) | 31890.7 | 307 |
| A0A1D6EQY4 | Putative ethanolamine kinase | 35827.3 | 308 |
| A0A1D6IX22 | PHD finger protein ALFIN-LIKE 6 | 34672.3 | 308 |
| A0A1R3QMY2 | 50kD gamma zein | 36005.6 | 308 |
| B4FBF6 | Mitochondrial dicarboxylate/tricarboxylate transporter DTC | 33147.3 | 308 |
| B4FTP9 | Protein BOBBER 1 | 34601.9 | 308 |
| B4FYX0 | Transcription factor Pur-alpha 1 | 33488.6 | 308 |
| B6T4G7 | Fibrillarin-2 | 32353.7 | 308 |
| B6TCG2 | Lipid binding protein | 34101.3 | 308 |
| B6TLT7 | Copper chaperone for superoxide dismutase | 32209.1 | 308 |
| C4IZK1 | Uncharacterized protein | 33370.5 | 308 |
| K7VGZ6 | 40S ribosomal protein SA | 33499.6 | 308 |
| A0A1D6FJN7 | Uncharacterized protein | 35942.4 | 309 |
| A0A1D6FMY6 | Uncharacterized protein | 34401.9 | 309 |
| A0A1D6PC74 | Glycerol-3-phosphate acyltransferase 9 | 35869.8 | 309 |
| A0A317Y3I5 | Gamma-interferon-inducible lysosomal thiol reductase | 34330.9 | 309 |
| B4FIE0 | Uncharacterized protein | 33033 | 309 |
| B4G1K7 | 26S proteasome regulatory subunit RPN13 | 33766.2 | 309 |
| B6STR1 | dTDP-4-dehydrorhamnose reductase | 34024.6 | 309 |
| B6TVC6 | Isoflavone reductase IRL | 32893.3 | 309 |
| C0P484 | Ras-related protein RABC2a | 33579.1 | 309 |
| C0PB92 | Uncharacterized protein | 33960.8 | 309 |
| K7U9T4 | YbaK/aminoacyl-tRNA synthetase-associated domain | 33500.3 | 309 |
| P52580 | Isoflavone reductase homolog IRL | 32851.3 | 309 |
| A0A1D6MJK0 | Ribosome biogenesis regulatory protein | 35372.5 | 310 |
| A0A1D6MTG0 | 60S ribosomal protein L11-1 | 34569.1 | 310 |
| B4FE53 | Uncharacterized protein | 33851.6 | 310 |
| B4FS63 | 26S proteasome non-ATPase regulatory subunit 7 homolog A | 34916.9 | 310 |
| B4G0P2 | Uncharacterized protein | 34652.8 | 310 |
| B6T8M6 | Mitochondrial 2-oxoglutarate/malate carrier protein | 33403.6 | 310 |
| B6TBJ6 | Ubiquitin fusion degradation protein 1 | 34205.5 | 310 |
| B6TRN5 | Aldose reductase | 34547 | 310 |
| B6TYJ1 | Pantoate--beta-alanine ligase | 32901.2 | 310 |
| B7ZYT3 | ER membrane protein complex subunit 2-A | 34323.1 | 310 |
| C0PJ20 | Uncharacterized protein | 32878.8 | 310 |
| K7W7S3 | Putative RING zinc finger domain superfamily protein isoform 1 | 32442.6 | 310 |
| Q8GZP8 | Satase isoform I | 32579.8 | 310 |
| Q8S4C4 | Thioesterase family protein, mRNA | 32807.8 | 310 |
| A0A317Y256 | Putative NAD(P)H-dependent oxidoreductase 1 | 35065 | 311 |
| B4F9T3 | Uncharacterized protein | 33266.9 | 311 |
| B4FKZ3 | Uncharacterized protein | 34620.4 | 311 |
| B6SKS2 | Uncharacterized protein | 33939.2 | 311 |
| B6T8I4 | Pre-mRNA-splicing factor ISY1 | 36492.9 | 311 |
| B6TAU0 | NADP-dependent D-sorbitol-6-phosphate dehydrogenase | 34653.3 | 311 |
| B6TCK3 | NADH-cytochrome b5 reductase | 33844.7 | 311 |
| B6U6K9 | Autophagy-related protein 3 | 34943.9 | 311 |
| B6UG16 | Deoxyribonuclease ycfH | 34076.4 | 311 |
| B7ZXZ2 | Aldo-keto reductase family 4 member C9 | 33858.3 | 311 |
| B8XVR6 | Autophagy-related protein 3 | 34944.8 | 311 |
| A0A1D6E917 | Plant UBX domain-containing protein 4 | 33194.7 | 312 |
| A0A1D6J5V1 | Farnesylated protein 3 | 33749.5 | 312 |
| A0A1D6LHB1 | Dirigent protein | 33409.2 | 312 |
| B4FNK8 | Chorismate mutase | 34919.6 | 312 |
| B4G1C2 | Uncharacterized protein | 34236.5 | 312 |
| B6TF54 | Chorismate mutase | 35004.6 | 312 |
| B6THK8 | UBA and UBX domain-containing protein | 33150.6 | 312 |
| B6TU00 | Chorismate mutase | 35074.7 | 312 |
| B6U9X1 | CDA1 | 31979 | 312 |
| B8A1N6 | Uncharacterized protein | 34409.8 | 312 |
| K7VNS9 | AT-rich element binding factor 3 | 34179.5 | 312 |
| A0A1D6FUJ6 | Bifunctional protein FolD 2 | 32808.9 | 313 |
| A0A1D6P927 | Putative jumonji-like transcription factor family protein | 34019.1 | 313 |
| B4FA28 | Glyoxylate reductase | 32544.4 | 313 |
| B4FKP4 | Peroxygenase 2 | 34230.2 | 313 |
| B6TBU7 | Serine/threonine-protein phosphatase | 35548.9 | 313 |
| B6TKQ4 | NAD(P)-linked oxidoreductase superfamily protein | 33871.5 | 313 |
| C0HEV8 | Secretory carrier-associated membrane protein | 35102.2 | 313 |
| C0P4K4 | L-ascorbate peroxidase S chloroplastic/mitochondrial | 34133.4 | 313 |
| C0PKZ2 | Uncharacterized protein | 34153.5 | 313 |
| K7TTX0 | Plant UBX domain-containing protein 4 | 33174.6 | 313 |
| A0A1D6JW34 | Chloride conductance regulatory protein ICln | 34560 | 314 |
| A0A1D6M6G1 | Chaperonin | 34243.5 | 314 |
| A0A317YI65 | 60S ribosomal protein L38 | 35524.4 | 314 |
| B4F9A4 | Deoxymugineic acid synthase 1 | 35377.3 | 314 |
| B4FP93 | Enoyl-CoA hydratase 2 peroxisomal | 34395.7 | 314 |
| B4FSX7 | (+)-neomenthol dehydrogenase | 33865.8 | 314 |
| B4FVP4 | Uncharacterized protein | 34885.1 | 314 |
| B6TB94 | Binding partner of ACD11 1 | 33269.2 | 314 |
| B6TPX4 | Plastid-lipid-associated protein 2 | 33626.9 | 314 |
| B6TTZ3 | Decarboxylase family protein | 34292.7 | 314 |
| C4JBV8 | (+)-neomenthol dehydrogenase | 34299.6 | 314 |
| Q43864 | Annexin | 35258.9 | 314 |
| A0A1D6GUW2 | GATA transcription factor 24 | 34420.2 | 315 |
| A0A1D6J4S4 | Amine oxidase | 35264.9 | 315 |
| A0A1D6KJW8 | 5'-methylthioadenosine/S-adenosylhomocysteine nucleosidase 2 | 33681.7 | 315 |
| A0A317YES3 | Uncharacterized protein | 33161.5 | 315 |
| B4FNN8 | Soluble epoxide hydrolase | 35378.4 | 315 |
| B4G107 | Uncharacterized protein | 33816 | 315 |
| B6T460 | Gibberellin receptor GID1L2 | 33999.1 | 315 |
| B6T7U3 | Uncharacterized enzyme involved in pigment biosynthesis | 33343.3 | 315 |
| B6TKR7 | Dirigent protein | 33014.9 | 315 |
| B6TM48 | Serine/threonine-protein phosphatase | 35811.2 | 315 |
| B6TPH0 | Lactoylglutathione lyase | 35161.8 | 315 |
| B7ZZM3 | Uncharacterized protein | 34037.9 | 315 |
| B8A1H0 | Glucose-6-phosphate 1-epimerase | 34240.6 | 315 |
| C0HGL6 | Ubiquinone biosynthesis protein COQ9 mitochondrial | 33661.8 | 315 |
| C0HI22 | Site-determining protein | 33094.9 | 315 |
| C0P503 | Uncharacterized protein | 33975.1 | 315 |
| K7TIR4 | 1-aminocyclopropane-1-carboxylate oxidase 5 | 35020.4 | 315 |
| A0A1D6GYZ2 | Uncharacterized protein | 36051.6 | 316 |
| A0A1D6I1E4 | Embryo defective 1381 | 34449.3 | 316 |
| A0A1D6NE76 | Cytidine deaminase | 32219.2 | 316 |
| B4F9U1 | Transducin/WD40 repeat-like superfamily protein | 35316.5 | 316 |
| B4FKX6 | Retinol dehydrogenase 14 | 34154.7 | 316 |
| B4FVE8 | Membrane-associated protein VIPP1 chloroplastic | 35006.1 | 316 |
| B4FY63 | Annexin | 35869.8 | 316 |
| B6T2X6 | ACI14 | 35088.5 | 316 |
| B6T6V3 | Membrane-associated 30 kDa protein | 35023.2 | 316 |
| B6T720 | DnaJ protein | 34586.2 | 316 |
| B6THT6 | 3-oxoacyl-reductase | 32631.4 | 316 |
| C0HGE9 | Uncharacterized protein | 34013.3 | 316 |
| C0PA53 | HSP40/DnaJ peptide-binding protein | 34645.3 | 316 |
| C0PAT3 | Uncharacterized protein | 33276.8 | 316 |
| C0PCA6 | Uncharacterized protein | 35312.5 | 316 |
| A0A317Y8A9 | Mitochondrial import receptor subunit TOM40-1 | 34897.7 | 317 |
| B4FDV7 | Uncharacterized protein | 35045 | 317 |
| B6SJQ3 | Pyridoxal 5'-phosphate synthase-like subunit PDX1.2 | 33486.2 | 317 |
| B6TGB5 | Phosphopantothenate--cysteine ligase | 35072 | 317 |
| B6TZU3 | Uncharacterized protein | 31449.6 | 317 |
| K7TFA7 | Putative oxidoreductase, aldo/keto reductase family protein | 34384.9 | 317 |
| K7VHN7 | Putative thaumatin domain family protein | 31447.9 | 317 |
| A0A1D6MBB0 | Nucleosome assembly protein 1 | 36358.3 | 318 |
| A0A317YAM5 | Protein FLX-like 1 | 34400 | 318 |
| B4FFY2 | Zinc finger CCCH domain-containing protein 54 | 34996.6 | 318 |
| B4FRP8 | Plastid-lipid-associated protein 2 | 34280.5 | 318 |
| B4FSE0 | Alba DNA/RNA-binding protein | 34398.6 | 318 |
| B6SJL4 | Uncharacterized protein | 34885.4 | 318 |
| B6SMR2 | Peroxidase | 33525.8 | 318 |
| B6T6D6 | 2C-type protein phosphatase protein | 34551.8 | 318 |
| B6T754 | ATP12 ATPase | 34705.8 | 318 |
| B6TE77 | 1-aminocyclopropane-1-carboxylate oxidase 1 | 34603.7 | 318 |
| B6TM99 | Ubiquitin thioesterase | 35104.1 | 318 |
| C0P2Y6 | Spermidine synthase 2 | 34877.6 | 318 |
| K7TN80 | Nitrilase-like protein 2 | 34825.6 | 318 |
| A0A1D6DUW9 | 26S proteasome non-ATPase regulatory subunit 7 homolog A | 35931 | 319 |
| A0A1D6N5L5 | Extradiol ring-cleavage dioxygenase | 35282.3 | 319 |
| A0A317Y7M1 | Haloacid dehalogenase-like hydrolase domain-containing protein Sgpp | 33967.4 | 319 |
| B4F8B2 | Peroxisome biogenesis protein 7 | 35179.3 | 319 |
| B4FNM4 | 60S acidic ribosomal protein P0 | 34488.3 | 319 |
| B4FVR4 | Exosome complex component RRP4-like protein | 35768.7 | 319 |
| B6T887 | Late embryogenesis abundant protein Lea14-A | 35203.6 | 319 |
| B6T9K7 | COMPASS-like H3K4 histone methylase component WDR5A | 34547.6 | 319 |
| B6TI69 | Indole-3-glycerol phosphate lyase | 33121.8 | 319 |
| E9JVD4 | Aldose reductase | 35659.5 | 319 |
| K7VF10 | Coiled-coil domain-containing protein 97 isoform 1 | 36239.2 | 319 |
| A0A1D6JFN1 | OSJNBa0033G05.6-like protein | 35373.6 | 320 |
| A0A1D6K161 | ELMO/CED-12 family protein | 37157.6 | 320 |
| A0A1D6KL36 | BAG family molecular chaperone regulator 1 | 34813.5 | 320 |
| A0A1D6M3Q3 | 1-(5-phosphoribosyl)-5-[(5-phosphoribosylamino)methylideneamino] imidazole-4-carboxamide isomerase chloroplastic | 35400.5 | 320 |
| B4FDB1 | NAD(P)-binding Rossmann-fold superfamily protein | 34930.6 | 320 |
| B6SRY1 | Glyoxylate reductase | 34500.3 | 320 |
| B6T428 | Succinate--CoA ligase [ADP-forming] subunit alpha, mitochondrial | 33196.1 | 320 |
| B6TK66 | Embryonic protein DC-8 | 33571.6 | 320 |
| B6UII7 | Epoxide hydrolase 2 | 35280.5 | 320 |
| C0PF45 | Peroxidase | 33237.9 | 320 |
| K7VB55 | Dihydroflavonol-4-reductase | 35258.2 | 320 |
| A0A1D6FZP6 | Uroporphyrinogen decarboxylase | 35353.2 | 321 |
| A0A1D6GU18 | Protein IRX15-LIKE | 33773.8 | 321 |
| A0A1D6H809 | Pyrimidine reductase riboflavin1 | 35523.1 | 321 |
| A0A1D6N276 | Protein SRC2-like protein | 32859.4 | 321 |
| A0A1D6N867 | Endoplasmic reticulum vesicle transporter protein | 36364.2 | 321 |
| B4FKV2 | Rhomboid-like protein 19 | 35073.7 | 321 |
| B4FR39 | Uncharacterized protein | 33909.7 | 321 |
| B4FYW7 | Nudix hydrolase 14 chloroplastic | 34593.6 | 321 |
| B6SJW3 | Uncharacterized protein | 33913.9 | 321 |
| B6T4E6 | Mo-molybdopterin cofactor sulfurase | 35637.3 | 321 |
| B6TPE1 | Uncharacterized protein | 35946.8 | 321 |
| B6TSF8 | Cell wall integrity protein scw1 | 34058.6 | 321 |
| B8A0M8 | Uncharacterized protein | 33936.2 | 321 |
| A0A1D6PI63 | 40S ribosomal protein S3a | 36296.8 | 322 |
| A0A1D6PTV9 | Cell wall integrity protein scw1 | 34751.7 | 322 |
| B2XWS4 | Isopentenyl transferase IPT2 | 34485.8 | 322 |
| B4FCM3 | Uncharacterized protein | 33814.9 | 322 |
| B4FK62 | Plastid division protein PDV2 | 34241.6 | 322 |
| B4FQC9 | Proline iminopeptidase | 36601.9 | 322 |
| B4FSL4 | Uncharacterized protein | 34520.3 | 322 |
| B4FWT1 | Serine/threonine-protein phosphatase | 36296.4 | 322 |
| B4G0A7 | NAD(P)-binding Rossmann-fold superfamily protein | 35101.7 | 322 |
| B4G1G1 | Desiccation-related protein PCC13-62 | 34030.4 | 322 |
| B6T7H1 | Dihydroflavonol-4-reductase | 35027.6 | 322 |
| B6TNK0 | mRNA decapping enzyme 2 | 36277.8 | 322 |
| B6U705 | 6-phosphogluconolactonase | 34416.8 | 322 |
| B8A2I6 | Uncharacterized protein | 35799.3 | 322 |
| C0PAH7 | ADP-ribosylation factor GTPase-activating protein AGD12 | 35065.4 | 322 |
| K7U066 | NAD(P)-binding Rossmann-fold superfamily protein | 32221.7 | 322 |
| A0A317YBJ7 | Uncharacterized protein | 35844.5 | 323 |
| B4FBJ5 | Uncharacterized protein | 34690.5 | 323 |
| B4FK34 | Cobalamin biosynthesis CobW-like protein | 36126.8 | 323 |
| B4FP72 | Protein FLC EXPRESSOR | 34899.6 | 323 |
| B4FS28 | Serine/threonine-protein phosphatase | 36349.2 | 323 |
| B6SK78 | Transcription factor PosF21 | 34865.4 | 323 |
| B6TA29 | Arp2/3 complex 34 kDa subunit | 36234 | 323 |
| B6TCR8 | Protein binding protein | 34654.6 | 323 |
| B6TI12 | Nuclear pore complex protein NUP35 | 35023 | 323 |
| B6TNC0 | Ankyrin repeat domain-containing protein 2A | 34863.6 | 323 |
| B6U2X8 | Xylanase inhibitor protein 1 | 35351.4 | 323 |
| B6U666 | Fructokinase-1 | 34603.4 | 323 |
| C4J906 | Uncharacterized protein | 34675.5 | 323 |
| A0A1D6FHR1 | Nicastrin | 36049 | 324 |
| A0A1D6FJW2 | Transcription factor GTE3 chloroplastic | 36461.2 | 324 |
| A0A1D6LWS4 | Putative DUF26 domain family protein | 31701.5 | 324 |
| A0A1D6MV47 | Alpha/beta-Hydrolases superfamily protein | 35797.1 | 324 |
| B4FAH3 | Tetratricopeptide repeat (TPR)-like superfamily protein | 36059.7 | 324 |
| B4FNF9 | Putative aldo-keto reductase 4 | 35638.4 | 324 |
| B4FPX5 | NADH dehydrogenase [ubiquinone] flavoprotein 2 mitochondrial | 35199.9 | 324 |
| B4G1H4 | Rhomboid-like protein | 35702.6 | 324 |
| B6TAC1 | Early fruit mRNA | 34639.6 | 324 |
| B6TZE6 | Pectinesterase 31 | 36114.8 | 324 |
| B6TZQ4 | Uncharacterized protein | 33599.3 | 324 |
| B6UAK0 | 6-phosphogluconolactonase | 34781.4 | 324 |
| B7ZY87 | Calcium-dependent lipid-binding (CaLB domain) family protein | 33213.3 | 324 |
| A0A1D6FC22 | Syntaxin-22 | 35140.6 | 325 |
| A0A1D6GL72 | HSP20-like chaperones superfamily protein | 34686.2 | 325 |
| A0A1D6HEY4 | Methylglutaconyl-CoA hydratase | 34751.5 | 325 |
| B4FFT0 | Uncharacterized protein | 34618.4 | 325 |
| B4FNQ0 | Uncharacterized protein | 35505.7 | 325 |
| B4FV87 | Dirigent protein | 34355.5 | 325 |
| B6SI84 | Clavaminate synthase-like protein | 35884.7 | 325 |
| B6TCV6 | ATP binding protein | 36367.1 | 325 |
| B6TXX0 | Ribose-phosphate pyrophosphokinase 4 | 36193.4 | 325 |
| B6TYC6 | OTU domain-containing protein 6B | 35532.4 | 325 |
| B8A0F9 | Uncharacterized protein | 36221.5 | 325 |
| B8A377 | Cysteine synthase | 34133.2 | 325 |
| C0PDX9 | Dirigent protein | 34588.6 | 325 |
| C0PFG4 | Alpha/beta-Hydrolases superfamily protein | 35562.9 | 325 |
| C0PML6 | Glucose-6-phosphate 1-epimerase | 36529.9 | 325 |
| E9NX36 | Nucleoside N-ribohydrolase 1b | 34646.4 | 325 |
| K7UA00 | Protein EXORDIUM | 33305.5 | 325 |
| A0A096Q886 | Eukaryotic translation initiation factor 3 subunit I | 36287.4 | 326 |
| B4F7T8 | Choline-phosphate cytidylyltransferase 2 | 37316 | 326 |
| B4FMT9 | Ubiquinone biosynthesis O-methyltransferase, mitochondrial | 34928.4 | 326 |
| B6SSB6 | Eukaryotic translation initiation factor 3 subunit I | 36259.4 | 326 |
| B6T177 | Non-green plastid inner envelope membrane protein | 35545.1 | 326 |
| B6TEY3 | Protein farnesyltransferase/geranylgeranyltransferase type I alphasubunit | 37594.3 | 326 |
| B6UGH3 | Uncharacterized protein | 31606.3 | 326 |
| C0HFQ1 | Dirigent protein | 34366.5 | 326 |
| C0PBL7 | Uncharacterized protein | 34246.5 | 326 |
| O24478 | B-keto acyl reductase | 35414.2 | 326 |
| Q6A4M2 | 3-ketoacyl reductase GL8B | 35427.2 | 326 |
| Q9M465 | Translocase of chloroplast | 36393.5 | 326 |
| Q9SBX0 | Translocase of chloroplast | 36572.8 | 326 |
| A0A1D6DVE9 | Uncharacterized protein | 35918.9 | 327 |
| A0A1D6ERI4 | Putative carboxylesterase 2 | 34850.8 | 327 |
| A0A1D6FI64 | GTP-binding protein SAR1A | 36820.9 | 327 |
| A0A1D6P4L8 | NAD(P)-binding Rossmann-fold superfamily protein | 35219.1 | 327 |
| A0A317YF11 | 2-hydroxyisoflavanone dehydratase | 35443.7 | 327 |
| B4F8D8 | Ubiquitin carboxyl-terminal hydrolase | 37016.5 | 327 |
| B4F8J6 | Sec14p-like phosphatidylinositol transfer family protein | 37493.6 | 327 |
| B4FGP1 | Calcium-dependent lipid-binding (CaLB domain) family protein | 35193.1 | 327 |
| B4FTP3 | RNA-binding (RRM/RBD/RNP motifs) family protein | 38152.6 | 327 |
| B4FU25 | Rhomboid-like protein | 36080.9 | 327 |
| B6STQ1 | Phi-1-like phosphate-induced protein | 33558.7 | 327 |
| B6TC25 | Gibberellin receptor GID1L2 | 35487.8 | 327 |
| B6TSC4 | Ubiquitin-protein ligase | 36368.2 | 327 |
| A0A1D6E561 | Inositol polyphosphate multikinase beta | 35518.8 | 328 |
| A0A1D6FUV3 | Phosphoglycerate mutase-like protein AT74H | 37999 | 328 |
| A0A1D6FVH2 | Serine/arginine-rich splicing factor RS2Z32 | 36907.4 | 328 |
| A0A1D6HSR3 | Putative carboxylesterase 15 | 35426.6 | 328 |
| A0A1D6K9H8 | Uncharacterized protein | 37041.3 | 328 |
| A0A1D6MS65 | Alpha/beta-Hydrolases superfamily protein | 35895.6 | 328 |
| B4F8J4 | Uncharacterized protein | 37311 | 328 |
| B4FD80 | Uncharacterized protein | 35676.4 | 328 |
| B4FEY9 | Acyl-CoA-binding domain-containing protein 1 | 35120.7 | 328 |
| B4FLR0 | GrpE protein homolog | 36363.2 | 328 |
| B6SRJ0 | SWIB/MDM2 domain containing protein | 36935.1 | 328 |
| B6TCC3 | DNA-directed RNA polymerase II 36 kDa polypeptide A | 36281.5 | 328 |
| B6TIV3 | Uncharacterized protein | 37300 | 328 |
| B6TLM3 | GrpE protein homolog | 36394.1 | 328 |
| B6TMJ8 | GDP-L-fucose synthase 1 | 35710.8 | 328 |
| B6TY66 | Tetratricopeptide repeat protein 15 | 35479.2 | 328 |
| C0HI50 | Ubiquitin carboxyl-terminal hydrolase | 37564.6 | 328 |
| C4J2K0 | Putative voltage-gated potassium channel subunit beta | 36488.1 | 328 |
| K7UPP4 | Putative AP2/EREBP transcription factor superfamily protein | 34228.4 | 328 |
| K7UWX4 | GrpE protein homolog | 36090 | 328 |
| K7V969 | Protein EXORDIUM | 32926.9 | 328 |
| A0A1D6H8U6 | 3-oxoacyl-[acyl-carrier-protein] reductase chloroplastic | 34150.3 | 329 |
| A0A1D6HWM8 | Protein transport protein SEC13 homolog B | 35447.9 | 329 |
| A0A1D6Q5P0 | Alcohol dehydrogenase-like 6 | 35052 | 329 |
| A0A317YGH3 | Peroxidase | 33447.3 | 329 |
| B4FFA1 | Oxidoreductase zinc-binding dehydrogenase family protein | 34488.6 | 329 |
| B4FQR3 | Aldose reductase | 36106.1 | 329 |
| B4FWS6 | Putative carboxylesterase 8 | 34564.2 | 329 |
| B4G1E6 | Uncharacterized protein | 35898.5 | 329 |
| B6SGM5 | Putative voltage-gated potassium channel subunit beta | 36513.1 | 329 |
| B6SUK4 | Ubiquinol oxidase | 37035.3 | 329 |
| B6SUW2 | Alpha/beta-Hydrolases superfamily protein | 36297.1 | 329 |
| B6SZA4 | SWIB/MDM2 domain containing protein | 37249.3 | 329 |
| B6T3J6 | RNA-binding protein 25 | 38500 | 329 |
| B6T438 | Protein AIG1 | 37184.9 | 329 |
| B6TLR8 | NAD(P)H-dependent oxidoreductase | 36120.1 | 329 |
| B6TT83 | SRC2 | 33446.4 | 329 |
| K7V725 | Os01g0606100-like protein | 33950.6 | 329 |
| A0A1D6GFC9 | DEAD-box ATP-dependent RNA helicase 51 | 37125.6 | 330 |
| A0A1D6JT18 | ATP synthase subunit gamma | 35650.5 | 330 |
| B4FD87 | Ribosome biogenesis regulatory protein | 36849.2 | 330 |
| B6SHQ7 | Acyl-CoA-binding domain-containing protein 6 | 35244.8 | 330 |
| B6SLR7 | Glyoxylate reductase | 33942.6 | 330 |
| B6TMI7 | Gibberellin receptor GID1L2 | 35061.2 | 330 |
| B6UHM3 | Arginine N-methyltransferase 2 | 36171.7 | 330 |
| C0PIX4 | Uncharacterized protein | 36662.6 | 330 |
| C0PJB6 | Uncharacterized protein | 35781.6 | 330 |
| C4J9Y5 | Bax inhibitor 1 | 36162.5 | 330 |
| K7VEW7 | Mitogen-activated protein kinase | 38588.9 | 330 |
| K7W544 | Putative carboxylesterase 2 | 35121.5 | 330 |
| K7WFV1 | ATP synthase subunit gamma | 35714.6 | 330 |
| A0A1D6EZ64 | Polygalacturonase inhibitor 1 | 35347.9 | 331 |
| A0A1D6PJ23 | Omega-amidase chloroplastic | 36220.7 | 331 |
| B4FIV0 | Succinate--CoA ligase [ADP-forming] subunit alpha, mitochondrial | 34279.3 | 331 |
| B4FYE5 | Replication factor C subunit 2 | 36538.8 | 331 |
| B6TA83 | Replication factor C subunit 4 | 36538.8 | 331 |
| B6TAD3 | Phospholipid scramblase | 37969.8 | 331 |
| B6TDD0 | Polygalacturonase inhibitor 1 | 35390 | 331 |
| B6TME1 | Epoxide hydrolase 2 | 37152.6 | 331 |
| B6TYI9 | Dihydroflavonol-4-reductase | 35440.4 | 331 |
| B6UI33 | Gibberellin receptor GID1L2 | 34730.8 | 331 |
| B6UI45 | Peroxidase | 35823.4 | 331 |
| B7ZY93 | Thioredoxin reductase | 34806 | 331 |
| C0PGW3 | Soluble epoxide hydrolase | 36515.9 | 331 |
| K4JFL0 | C3H-type transcription factor (Fragment) | 36133.7 | 331 |
| K7TPL8 | NAD(P)-linked oxidoreductase superfamily protein | 36556.5 | 331 |
| Q9XGC6 | Adenosine kinase (Fragment) | 36031.9 | 331 |
| A0A1D6P7V3 | 5-hydroxyisourate hydrolase | 36221.5 | 332 |
| A0A1Q0ZFB6 | Alba DNA/RNA-binding protein | 36558.7 | 332 |
| A0A1R3MB28 | Malate dehydrogenase | 35589.6 | 332 |
| B4FED4 | Dihydroflavonol-4-reductase | 36689.2 | 332 |
| B4FFS6 | Casein kinase II subunit alpha | 39229.9 | 332 |
| B4FMV1 | Uncharacterized protein | 36182.6 | 332 |
| B4FPK4 | THO complex subunit 3 | 36892.4 | 332 |
| B4FRM5 | Red chlorophyll catabolite reductase chloroplastic | 36426.3 | 332 |
| B4G0X5 | Peroxidase | 35436.4 | 332 |
| B6SND5 | SRC2 | 33719.6 | 332 |
| B6T6S5 | Glucose-6-phosphate 1-epimerase | 36626.5 | 332 |
| B6T753 | Dehydrogenase/reductase SDR family member 7 | 36127.7 | 332 |
| B6TKC8 | Gibberellin receptor GID1L2 | 35303.2 | 332 |
| B6TPA7 | Dihydroflavonol-4-reductase | 36275.4 | 332 |
| B6TSD4 | ADP-ribosylation factor GTPase-activating protein AGD12 | 37042 | 332 |
| B6TWG6 | COP9 signalosome complex subunit 6a | 36245 | 332 |
| B7ZYJ9 | Protein XAP5 CIRCADIAN TIMEKEEPER | 38999.4 | 332 |
| C0P7C9 | CK2 protein kinase alpha 4 | 39357.9 | 332 |
| C0PFN4 | Glucose-6-phosphate 1-epimerase | 36652.5 | 332 |
| C0PKE3 | Uncharacterized protein | 35412.9 | 332 |
| K7U372 | GrpE protein homolog | 36166.1 | 332 |
| K7UYZ9 | ADP-ribosylation factor GTPase-activating protein AGD12 | 36949.7 | 332 |
| A0A1D6HWP7 | Probable cytosolic iron-sulfur protein assembly protein CIAO1 homolog | 36761.8 | 333 |
| B4FR86 | Spermidine synthase 2 | 36191 | 333 |
| B4FTG1 | SUMO-activating enzyme subunit 1B-2 | 36497.5 | 333 |
| B6TEU8 | COP9 signalosome complex subunit 6a | 36365 | 333 |
| K7U4C2 | Uncharacterized protein | 36635.9 | 333 |
| A0A1D6HB17 | Gibberellin receptor GID1L2 | 35317.2 | 334 |
| A0A1D6HVN8 | Survival protein SurE-like phosphatase/nucleotidase | 35531.8 | 334 |
| A0A1D6N4Q3 | Ribonuclease 2 | 37602.2 | 334 |
| A0A1D6N5J3 | Putative pectinesterase/pectinesterase inhibitor 51 | 35022.2 | 334 |
| B4F7W5 | Alpha/beta-Hydrolases superfamily protein | 36741.6 | 334 |
| B4FBV2 | 2-oxoglutarate (2OG) and Fe(II)-dependent oxygenase superfamily protein | 37670.3 | 334 |
| B4FKM1 | Guanine nucleotide-binding protein beta subunit-like protein | 36236.3 | 334 |
| B4FXQ4 | Ataxin-3-like protein | 36085.4 | 334 |
| B6SJ21 | Guanine nucleotide-binding protein beta subunit-like protein | 36236.3 | 334 |
| B6T7A7 | Uncharacterized protein | 35929.4 | 334 |
| B6T7D7 | Chaperone protein dnaJ 10 | 37542.3 | 334 |
| B6TBV3 | PDE191 | 37338.5 | 334 |
| B6TC50 | ZCW7 | 38290.2 | 334 |
| B6THQ5 | Ataxin-3 | 36069.4 | 334 |
| B6TJD1 | Protein binding protein | 36160.1 | 334 |
| B6TKF5 | Uncharacterized protein | 36139.7 | 334 |
| B6TR38 | Basic endochitinase A | 35301.3 | 334 |
| B6TWW2 | Purple acid phosphatase | 37965.7 | 334 |
| B6U6W0 | Peroxidase | 34801.9 | 334 |
| B6UEG3 | Uncharacterized protein | 35340.7 | 334 |
| C0PEF9 | WAPL (Wings apart-like protein regulation of heterochromatin) protein | 35944.8 | 334 |
| K7TSP8 | Eukaryotic translation initiation factor 3 subunit H | 38101 | 334 |
| K7V8I9 | UvrB/uvrC motif-containing protein | 37756.1 | 334 |
| B4F8I5 | Mitochondrial carrier protein CoAc2 | 36462.1 | 335 |
| B4FBM4 | Peroxisomal adenine nucleotide carrier 1 | 36713.9 | 335 |
| B4FQA9 | Peroxidase | 35758.8 | 335 |
| B4G137 | Ankyrin repeat domain-containing protein 2A | 36249.9 | 335 |
| B6STR9 | Nuclease | 36921.8 | 335 |
| B6T4W8 | Leucine carboxyl methyltransferase 1 homolog | 37650.3 | 335 |
| B6T9S9 | Peroxisomal carrier protein | 36726 | 335 |
| B6TB39 | Fructokinase-2 | 35487.1 | 335 |
| B6TIJ1 | Mu-crystallin | 34544.2 | 335 |
| B6TPJ8 | Fructokinase-2 | 35469 | 335 |
| B6TXG5 | WD-repeat protein 57 | 37421.2 | 335 |
| C0HFR0 | Phosphoglycerate mutase-like protein | 37063.1 | 335 |
| C0P4U5 | Uncharacterized protein | 37321.6 | 335 |
| A0A1D6EZR5 | Putative MO25-like protein | 38946.5 | 336 |
| A0A1D6J379 | Phosphoglycerate mutase-like protein | 37591.7 | 336 |
| A0A1D6LVH7 | Protein EXORDIUM | 34334.7 | 336 |
| A0A317Y6M7 | Diaminopimelate epimerase, chloroplastic | 36453.2 | 336 |
| B4FPM2 | Aldose 1-epimerase | 36501.9 | 336 |
| B6TAM1 | Peroxisomal carrier protein | 36970.3 | 336 |
| B6TFA5 | Retrotransposon protein Ty3-gypsy subclass | 37057.8 | 336 |
| B6TFA8 | Protein Mo25 | 38864.5 | 336 |
| B6TH79 | Lichenase-2 | 34913.2 | 336 |
| C0P797 | Putative MO25-like protein | 38966.5 | 336 |
| K7U2B4 | HEAT repeat family protein | 36588.6 | 336 |
| P93633 | Actin (Fragment) | 36945.1 | 336 |
| A0A1D6DW09 | Putative isoaspartyl peptidase/L-asparaginase 2 | 34910.3 | 337 |
| A0A1D6E530 | Peroxidase | 35475.8 | 337 |
| A0A1D6PC23 | Calcium-dependent phosphotriesterase superfamily protein | 35724.3 | 337 |
| A0A317Y7F0 | Peroxidase | 36289.9 | 337 |
| B4FUK7 | Hydroxyproline-rich glycoprotein family protein | 37881.3 | 337 |
| B7ZXQ8 | Peroxidase | 35485.8 | 337 |
| C4IZW2 | Uncharacterized protein | 38290.2 | 337 |
| C4JBS8 | Glyceraldehyde-3-phosphate dehydrogenase | 36494.4 | 337 |
| Q43359 | Cytosolic glyceroldehyde-3-phosphate dehydrogenase GAPC4 | 36450.4 | 337 |
| A0A1D6FMA8 | Serine/threonine-protein phosphatase | 38117.5 | 338 |
| A0A1D6FVH3 | Serine/arginine-rich splicing factor RS2Z32 | 38085.5 | 338 |
| A0A317YE26 | Sulfotransferase | 37374.5 | 338 |
| B4F8B4 | Aldolase-type TIM barrel family protein | 36556.6 | 338 |
| B4FIW6 | Serine racemase | 35757.5 | 338 |
| B6SMK8 | Inositol-1-monophosphatase | 36574.1 | 338 |
| B6SUB0 | Root border cell-specific protein | 37005.6 | 338 |
| K7VNQ7 | Mitochondrial import receptor subunit TOM40-1 | 37345.1 | 338 |
| A0A1D6J1P7 | PTI1-like tyrosine-protein kinase 3 | 37020.1 | 339 |
| A0A1D6N1E0 | Essential protein Yae1 N-terminal | 36621.7 | 339 |
| A0A1D6QA38 | Zn-dependent hydrolase, including glyoxylase | 38261.8 | 339 |
| B4FHN3 | L-ascorbate peroxidase S chloroplastic/mitochondrial | 37043.4 | 339 |
| B4FQ89 | Putative carboxylesterase 15 | 36140.8 | 339 |
| B4FTR6 | 3'-5' exonuclease domain-containing protein / K homology domain-containing protein / KH domain-containing protein | 38106.5 | 339 |
| B8A187 | Putative quinone-oxidoreductase homolog chloroplastic | 35241.5 | 339 |
| D5FGN7 | SnRK2.2 | 38549 | 339 |
| E1AFV5 | Beta-1,3-glucanase | 35976.4 | 339 |
| K7TID5 | Peroxidase | 36795.2 | 339 |
| A0A096QXB7 | Annexin | 38250 | 340 |
| A0A096RBT2 | D-amino-acid transaminase chloroplastic | 36448.7 | 340 |
| A0A1D6LAP1 | Lysine--tRNA ligase chloroplastic/mitochondrial | 38087.9 | 340 |
| A0A1D6Q972 | ADP-ribosylation factor GTPase-activating protein AGD12 | 38489.8 | 340 |
| A0A1X7YG11 | Autophagy-related protein 3 | 38428 | 340 |
| A0A317YEK4 | Uncharacterized protein | 37812.3 | 340 |
| B4FQ58 | Uncharacterized protein | 37084 | 340 |
| B4FRJ1 | Malate dehydrogenase | 35639.7 | 340 |
| B4FSV8 | Oligosaccharide transporter | 37950.8 | 340 |
| B4FTQ1 | Arginase 1 mitochondrial | 36954.8 | 340 |
| B4FVH1 | Malate dehydrogenase | 35375.5 | 340 |
| B4FZU8 | Malate dehydrogenase | 35285.4 | 340 |
| B4G1G6 | Transducin/WD40 repeat-like superfamily protein | 36936 | 340 |
| B6T3R6 | Serine-threonine kinase receptor-associated protein | 36937 | 340 |
| B6THJ2 | Mitochondrial uncoupling protein 3 | 35809.4 | 340 |
| K0DG49 | C3H18 C3H type transcription factor (Fragment) | 36287.6 | 340 |
| A0A1D6ENZ5 | Lipid phosphate phosphatase 2 | 38284 | 341 |
| A0A1D6HQQ2 | DDRGK domain-containing protein 1 | 38399.6 | 341 |
| A0A1D6IN00 | Peroxidase | 35283.1 | 341 |
| A0A1D6KGV8 | Alpha/beta-Hydrolases superfamily protein | 38066.9 | 341 |
| A0A1D6Q9Z0 | Uncharacterized protein | 37489.9 | 341 |
| A0A1D6QKF2 | COP9 signalosome complex subunit 6a | 37225 | 341 |
| B4FJZ6 | Uncharacterized protein | 36393.9 | 341 |
| B6TIN4 | Methionine aminopeptidase | 37151.3 | 341 |
| B6UHG6 | Uncharacterized protein | 37089.3 | 341 |
| C0PKD2 | Transducin/WD40 repeat-like superfamily protein | 36826.8 | 341 |
| K7VCN5 | Peroxidase | 36619.4 | 341 |
| A0A096PZ87 | 3-hydroxyisobutyrate dehydrogenase | 36441.4 | 342 |
| A0A1D6EP47 | OBP32pep | 38893.1 | 342 |
| A0A1D6FGJ9 | Peroxidase | 35040.2 | 342 |
| A0A1D6FTB8 | Gamma-glutamyl hydrolase 1 | 37914.8 | 342 |
| A0A1D6IKD3 | Uncharacterized protein | 37520.5 | 342 |
| A0A1D6IRU3 | Uncharacterized protein | 34628.4 | 342 |
| A0A1D6JAW2 | (+)-neomenthol dehydrogenase | 37041.3 | 342 |
| A0A1D6LDL7 | Sequence-specific DNA binding transcription factor | 38868.2 | 342 |
| A0A1D6NAB1 | Homocysteine S-methyltransferase4 | 36401.4 | 342 |
| A0A317Y6U1 | Protein-ribulosamine 3-kinase, chloroplastic | 38046.8 | 342 |
| B4FSB1 | Amino acid kinase family protein | 36681.6 | 342 |
| B6T4W5 | Hyoscyamine 6-dioxygenase | 37728.1 | 342 |
| B6T5H6 | Auxin-induced protein PCNT115 | 37525.6 | 342 |
| B6TU39 | Peroxidase | 34962.9 | 342 |
| B6UBJ6 | Gamma-glutamyl hydrolase | 37883.8 | 342 |
| C0P3P8 | Inositol-tetrakisphosphate 1-kinase | 37312.3 | 342 |
| C0PFM8 | Protein RETICULATA-RELATED 3 chloroplastic | 35139.1 | 342 |
| K7U2X9 | Heterodimeric geranylgeranyl pyrophosphate synthase small subunit chloroplastic | 36135 | 342 |
| K7UH13 | Adenosine kinase 2 | 37002.9 | 342 |
| A0A1D6F9X0 | Gibberellin receptor GID1L2 | 35793 | 343 |
| B4FBM0 | Syntaxin-32 | 37769.3 | 343 |
| B4FH75 | 4-hydroxy-tetrahydrodipicolinate reductase 2 chloroplastic | 37308.8 | 343 |
| B6STJ6 | Sulfotransferase | 37807.9 | 343 |
| B6T632 | Uncharacterized protein | 37894.9 | 343 |
| B6TBG3 | Dihydrodipicolinate reductase | 37283.8 | 343 |
| B6TKI9 | Protein RAE1 | 38091.7 | 343 |
| B6UH61 | Mitotic checkpoint protein BUB3 | 37564.8 | 343 |
| D3G6F2 | Geranylgeranyl pyrophosphate synthase4 | 36119 | 343 |
| K7UGV0 | Zn-finger, RanBP-type, containing protein | 36348.3 | 343 |
| K7VP16 | Protein RETICULATA-RELATED 3 chloroplastic | 35003.9 | 343 |
| A0A1D6E5Z5 | Adenosine kinase 2 | 37287.3 | 344 |
| A0A1D6FBS3 | Inositol-1-monophosphatase | 37305 | 344 |
| A0A1D6IM64 | Protein CTR9-like protein | 38557.7 | 344 |
| A0A1D6KHX5 | Serine/arginine-rich splicing factor SC35 | 39479 | 344 |
| B4FS84 | Protein BTR1 | 36935.2 | 344 |
| B4G0K5 | Uncharacterized protein | 38394.7 | 344 |
| B6TVI5 | Uncharacterized protein | 34945.8 | 344 |
| C0P6Q4 | Protein BTR1 | 37030.2 | 344 |
| K7VEI4 | Alpha/beta-Hydrolases superfamily protein | 39025.7 | 344 |
| P93852 | Methyltransferase | 38778.8 | 344 |
| A0A1D6E9W8 | Protein phosphatase methylesterase 1 | 38055.1 | 345 |
| B6TEB2 | Acetylglutamate kinase | 35445.8 | 345 |
| K7UZN5 | Putative oxidoreductase, aldo/keto reductase family protein | 38251.3 | 345 |
| A0A1D6G824 | Dual specificity protein phosphatase Diacylglycerol kinase catalytic region | 37855.8 | 346 |
| A0A1D6G947 | Protein FATTY ACID EXPORT 3 chloroplastic | 37923.8 | 346 |
| A0A1D6LJL5 | Binding partner of ACD11 1 | 36444.2 | 346 |
| A0A1D6M1U3 | ASF/SF2-like pre-mRNA splicing factor SRP31 | 39341.6 | 346 |
| A0A1D6MJZ6 | Multidrug resistance associated protein7 | 36521.1 | 346 |
| B4F810 | DNAJ heat shock family protein | 38191.2 | 346 |
| B4F8G9 | Uncharacterized protein | 38534.9 | 346 |
| B4FK90 | Brain protein 16 | 37443.3 | 346 |
| B4FR53 | Uncharacterized protein | 37101.1 | 346 |
| B4FSD4 | Acetylglutamate kinase chloroplastic | 35421.7 | 346 |
| B4FT62 | Putative aldo-keto reductase 4 | 37781.7 | 346 |
| B6SRH9 | Peroxidase | 36479 | 346 |
| B6TD84 | Auxin-induced protein PCNT115 | 37616.9 | 346 |
| B6TQU4 | Oxidoreductase/ zinc ion binding protein | 36371.9 | 346 |
| C0PLV4 | Uncharacterized protein | 38437.7 | 346 |
| C4J1Q6 | WAT1-related protein | 38108 | 346 |
| B4FX70 | ARM repeat superfamily protein | 37699.5 | 347 |
| B6TLR9 | Cathepsin B-like cysteine proteinase 3 | 38041 | 347 |
| B6UHT1 | Uncharacterized protein | 39602.9 | 347 |
| C0HEE6 | Peroxidase | 38003.6 | 347 |
| Q8GT27 | Ubiquinol oxidase | 38719.2 | 347 |
| A0A1D6E7P5 | ATP/GTP binding protein | 40527.3 | 348 |
| A0A1D6J6W6 | Ubiquitin interaction motif-containing protein | 37895.3 | 348 |
| A0A1D6KD20 | Heterogeneous nuclear ribonucleoprotein 1 | 37250 | 348 |
| A0A1D6MBJ6 | DNAJ heat shock N-terminal domain-containing protein | 39011.8 | 348 |
| A0A1D6MFW4 | TPR repeat-containing thioredoxin TTL1 | 38407.3 | 348 |
| B4FBQ5 | Protein phosphatase 2C isoform epsilon | 37385.6 | 348 |
| B4FLW2 | 4-hydroxy-tetrahydrodipicolinate synthase | 37897.2 | 348 |
| B4G1E5 | Cytosolic Fe-S cluster assembly factor NBP35 | 37184.4 | 348 |
| B6SRX0 | DNA binding protein | 39138.3 | 348 |
| B6T3D4 | Methionyl-tRNA synthetase | 37809.4 | 348 |
| B6T6P3 | Clathrin binding protein | 39218.1 | 348 |
| B6TEA3 | Cytosolic Fe-S cluster assembly factor NBP35 | 37210.4 | 348 |
| B6TIQ8 | ATP/GTP binding protein | 40549 | 348 |
| B6U7Y1 | Activator heat shock protein ATPase | 38600.3 | 348 |
| K7VHV1 | Phosphoglycerate mutase-like protein AT74H | 39908.1 | 348 |
| A0A1D6HAM1 | Tubulin-folding cofactor C | 38099.3 | 349 |
| A0A317Y4U3 | Metacaspase-1 | 38093.1 | 349 |
| B4FJX1 | Peroxidase | 35751.8 | 349 |
| B4FV70 | Gibberellin 20-oxidase4 | 39165.9 | 349 |
| B4FZN1 | Uncharacterized protein | 36843.1 | 349 |
| B6SIJ0 | Corticosteroid 11-beta-dehydrogenase isozyme 1 | 38550.7 | 349 |
| B6SS03 | Arogenate dehydrogenase isoform 2 | 38561.3 | 349 |
| B6T940 | VTC2 | 39712.9 | 349 |
| B6TE04 | Purple acid phosphatase | 38823.2 | 349 |
| B6TF99 | Gibberellin 20 oxidase 2 | 39228 | 349 |
| C0PM67 | Uncharacterized protein | 38211.7 | 349 |
| K7V269 | Putative metacaspase family protein | 36088.3 | 349 |
| K7VEP4 | Pyridoxal-5'-phosphate-dependent enzyme family protein | 37327.7 | 349 |
| A0A1D6E803 | Galactose oxidase/kelch repeat superfamily protein | 39910 | 350 |
| A0A1D6ELK2 | DNA-directed RNA polymerases II IV and V subunit 3 | 38867.6 | 350 |
| A0A1D6I5Q9 | Mitochondrial phosphate carrier protein 2 mitochondrial | 37108.8 | 350 |
| A0A1D6LZ71 | DnaJ protein ERDJ3B | 39305.5 | 350 |
| B4FAG0 | dTDP-glucose dehydratase homolog csu219 | 39309.8 | 350 |
| B4FF24 | UDP-glucuronic acid decarboxylase 5 | 39325.9 | 350 |
| B4FNT8 | Aldolase like | 37425.2 | 350 |
| B4FSA4 | Nucleotide-diphospho-sugar transferase superfamily protein | 38802.1 | 350 |
| B4FU67 | Uncharacterized protein | 38877 | 350 |
| B6TE01 | Myosin-like protein | 39999.7 | 350 |
| B6TQB4 | 3-beta hydroxysteroid dehydrogenase/isomerase | 37745.7 | 350 |
| B6UGU4 | Steroleosin | 38505.2 | 350 |
| C0PBR5 | Mitogen-activated protein kinase kinase 2 | 38768.1 | 350 |
| C4J3L7 | Uncharacterized protein | 37544.7 | 350 |
| K7UU30 | DnaJ protein ERDJ3B | 39216.6 | 350 |
| Q548K3 | Farnesyl diphosphate synthase | 40013.2 | 350 |
| A0A1D6K0A5 | Uncharacterized protein | 38702.7 | 351 |
| A0A1D6KTZ3 | Chaperonin2 | 37406.8 | 351 |
| A0A1D6MA66 | Serine acetyltransferase 5 | 37352.5 | 351 |
| A0A1D6NSG6 | Putative transcription elongation factor SPT5 homolog 1 | 39841.2 | 351 |
| B4F8K5 | NAD(P)-binding Rossmann-fold superfamily protein | 37928.6 | 351 |
| B6SI41 | Leucoanthocyanidin dioxygenase | 40051.4 | 351 |
| B6TAK5 | Receptor-type tyrosine-protein phosphatase S | 39780.5 | 351 |
| C0P6C7 | HSP40/DnaJ peptide-binding protein | 37827.8 | 351 |
| C4J6R6 | V-type proton ATPase subunit | 40743.1 | 351 |
| A0A1D6FL73 | RNA-binding (RRM/RBD/RNP motifs) family protein | 39000.8 | 352 |
| A0A1D6IXC8 | C2H2-like zinc finger protein | 40554.9 | 352 |
| A0A1D6JQ00 | Embryonic protein DC-8 | 37055.3 | 352 |
| A0A1D6L1S7 | Putative serine/threonine protein kinase IREH1 | 40074.4 | 352 |
| A0A1D6NFB2 | Protein-lysine N-methyltransferase ZEAMMB73_Zm00001d043827 | 38180 | 352 |
| B4F833 | Diaminopimelate epimerase chloroplastic | 37798.8 | 352 |
| B4FE46 | LUC7 N_terminus domain-containing protein | 41054.6 | 352 |
| B4FIL5 | Leucine-rich repeat (LRR) family protein | 39507.7 | 352 |
| B4FTG2 | 3-methyl-2-oxobutanoate hydroxymethyltransferase | 36785.9 | 352 |
| B6TRD8 | Import inner membrane translocase subunit TIM50 | 39312.1 | 352 |
| B6TYV2 | Inner envelope membrane protein | 39131.1 | 352 |
| K7VE95 | Mitochondrial outer membrane import complex protein METAXIN | 38962.8 | 352 |
| A0A1D6F548 | Putative carboxylesterase 18 | 37600 | 353 |
| A0A1D6GLD0 | 5'-nucleotidase | 39261.5 | 353 |
| A0A1D6ICB7 | (S)-ureidoglycine aminohydrolase | 38778 | 353 |
| B8A2R2 | Uncharacterized protein | 37817.4 | 353 |
| B8A3M9 | Uncharacterized protein | 38241.4 | 353 |
| C0PIP7 | Protein FLX-like 1 | 38647.6 | 353 |
| C0PP56 | Arginine/serine-rich splicing factor RS2Z39 transcript III | 39005.9 | 353 |
| Q3V810 | Anthocyanin biosynthetic gene regulator PAC1 | 38263.6 | 353 |
| A0A1D6HT77 | Galactose-1-phosphate uridyl transferase-like protein | 39206.2 | 354 |
| A0A1D6HYS5 | Phosphoglucosamine mutase family protein | 38755.4 | 354 |
| A0A1D6KLE6 | PWWP domain protein | 38440.8 | 354 |
| A0A1D6NVD1 | Pyridoxal-5'-phosphate-dependent enzyme family protein | 37965.5 | 354 |
| A0A1D6Q0H9 | Uncharacterized protein | 38626.9 | 354 |
| B4F7V0 | Putative inactive dual specificity protein phosphatase-like | 39044.2 | 354 |
| B4F8E3 | Electron transfer flavoprotein subunit alpha mitochondrial | 37057.2 | 354 |
| B4FBW5 | Mannitol dehydrogenase | 38378.7 | 354 |
| B4FWA8 | Thiamine thiazole synthase, chloroplastic | 37104.5 | 354 |
| B6T631 | Uncharacterized protein | 40220.9 | 354 |
| B6TDW8 | D-erythro-sphingosine kinase/ diacylglycerol kinase | 38504.5 | 354 |
| B6TS22 | O-methyltransferase ZRP4 | 39183.1 | 354 |
| B6TX55 | Uncharacterized protein | 40007.4 | 354 |
| K7V9Q6 | Src2-like protein | 36149 | 354 |
| A0A1D6E673 | Putative low-specificity L-threonine aldolase 1 | 37813 | 355 |
| A0A1D6FWR5 | Nucleosome assembly protein 1 | 40099.9 | 355 |
| A0A1D6KM35 | Ubiquitin carboxyl-terminal hydrolase | 40368.3 | 355 |
| A0A1D6MWA5 | Homocysteine S-methyltransferase 3 | 38600.5 | 355 |
| A0A1D6N083 | ABC-type transport system periplasmic component | 38445 | 355 |
| B4FAL9 | Fructose-bisphosphate aldolase | 38589.8 | 355 |
| B4FVE3 | Farnesyl diphosphate synthase3 | 40805.4 | 355 |
| B4FWP0 | Fructose-bisphosphate aldolase | 38458.7 | 355 |
| B4G015 | Thiamine thiazole synthase, chloroplastic | 37307.5 | 355 |
| B4G198 | Shwachman-Bodian-Diamond syndrome protein | 40327.8 | 355 |
| B4G1N4 | CsAtPR5 | 39898.8 | 355 |
| B6T856 | L-lactate dehydrogenase | 38396.4 | 355 |
| B6TK96 | Homocysteine S-methyltransferase 3 | 38438.3 | 355 |
| C0HI30 | UDP-glucose 4-epimerase 1 | 38914.2 | 355 |
| K0DCS9 | GBP17 GeBP type transcription factor (Fragment) | 39338.5 | 355 |
| K7VMX2 | 4,5-DOPA dioxygenase extradiol | 39450.1 | 355 |
| O49975 | MEK homolog1 | 39874.6 | 355 |
| Q7XZQ2 | UDP-glucose-4-epimerase | 38888.2 | 355 |
| Q94G04 | Diphosphonucleotide phosphatase 1 | 37387.7 | 355 |
| A0A1D6JNB8 | Tetratricopeptide repeat (TPR)-like superfamily protein | 37383 | 356 |
| A0A1D6MPS7 | Epoxide hydrolase 2 | 39369.5 | 356 |
| B6TAU8 | Serine/threonine-protein kinase SAPK7 | 40704 | 356 |
| B6TNK2 | 2-oxoglutarate (2OG) and Fe(II)-dependent oxygenase superfamily protein | 39243.7 | 356 |
| B8A2V8 | Omega-amidase chloroplastic | 38404.4 | 356 |
| B9TSW1 | Glutamine synthetase | 39309.9 | 356 |
| B9TSW5 | Glutamine synthetase | 39250.9 | 356 |
| C0P813 | Peroxidase | 37055.4 | 356 |
| C0PEJ4 | Serine/threonine-protein kinase SRK2A | 40689.9 | 356 |
| C0PL67 | Uncharacterized protein | 36666.6 | 356 |
| K7U1L9 | 1-aminocyclopropane-1-carboxylate oxidase15 | 39417.2 | 356 |
| A0A1D6FL50 | Putative DUF1296 domain containing family protein | 37640.1 | 357 |
| A0A1D6FPM7 | Ethylene-responsive transcription factor ABR1 | 36208.9 | 357 |
| A0A1D6GR74 | NagB/RpiA/CoA transferase-like superfamily protein | 38590.1 | 357 |
| B6SZI1 | Uncharacterized protein | 39544.4 | 357 |
| B6TD27 | Plastidic phosphate translocator-like protein1 | 39094.7 | 357 |
| B6TKK2 | Gibberellin receptor GID1L2 | 38350.2 | 357 |
| B8A3F5 | Uncharacterized protein | 41021.9 | 357 |
| C4JC57 | 2-oxoglutarate (2OG) and Fe(II)-dependent oxygenase superfamily protein | 40145.2 | 357 |
| Q8L886 | Nucellin-like aspartic protease (Fragment) | 38424.5 | 357 |
| A0A1D6F0W7 | NAD(P)-binding Rossmann-fold superfamily protein | 38622.6 | 358 |
| A0A1D6IAL9 | Uncharacterized protein | 39105.7 | 358 |
| A0A1D6LKR7 | Leucine carboxyl methyltransferase 1 homolog | 40017 | 358 |
| A0A1D6MDW7 | 26S proteasome regulatory subunit RPN13 | 39938.9 | 358 |
| A0A1D6P4T5 | Ribose-phosphate pyrophosphokinase | 39396.1 | 358 |
| B6TDE0 | NADP-dependent oxidoreductase P1 | 38935.2 | 358 |
| B7ZYU7 | Uncharacterized protein | 37570.7 | 358 |
| C4JBG1 | Uncharacterized protein | 40499.7 | 358 |
| A0A1D6N6L0 | Catalytic/ hydrolase | 39645.6 | 359 |
| B4FCZ7 | Uncharacterized protein | 37628 | 359 |
| B4FD46 | Uncharacterized protein | 40728.5 | 359 |
| B4FLM2 | Serine/threonine-protein kinase SRK2A | 41510.7 | 359 |
| B4FNP5 | CXC domain containing TSO1-like protein 1 | 39939.7 | 359 |
| B6SLE7 | Legumin-like protein | 37833.8 | 359 |
| B6TBU8 | Zinc finger protein-like 1 | 39730.6 | 359 |
| B6TU38 | Uncharacterized protein | 40953.8 | 359 |
| B6UA53 | Serine/threonine-protein kinase SAPK4 | 41438.6 | 359 |
| D5FGP3 | SnRK2.8 | 40576.7 | 359 |
| A0A1D6EG28 | Phosphoglycerate mutase-like protein | 40019.6 | 360 |
| A0A1D6F2P9 | Nuclear pore complex protein NUP98A | 40406.6 | 360 |
| A0A1D6GY38 | TMPIT-like protein | 40527.5 | 360 |
| A0A1D6LYW3 | Peroxidase | 38671.6 | 360 |
| A0A1D6PGH7 | Protein VAC14-like protein | 40088.5 | 360 |
| B4FA46 | Probable magnesium transporter | 38700.9 | 360 |
| B4FG65 | Alpha/beta-Hydrolases superfamily protein | 40330.4 | 360 |
| B4FGA2 | RING/U-box superfamily protein | 39834.7 | 360 |
| B4FRN6 | Methionyl-tRNA formyltransferase | 38740.3 | 360 |
| B6SHX1 | Malate dehydrogenase | 37866.6 | 360 |
| B6SRK3 | Arogenate dehydrogenase isoform 2 | 40258.5 | 360 |
| B6TAF8 | Thiol protease aleurain | 39070.8 | 360 |
| B6U0A3 | Catalytic/ hydrolase | 40327.5 | 360 |
| C0HEX8 | Uncharacterized protein | 40318.8 | 360 |
| K7URV3 | Inositol-1-monophosphatase | 38816.8 | 360 |
| Q84TL7 | Legumin-like protein | 37841.9 | 360 |
| A0A1D6GN48 | Uncharacterized protein | 40297.7 | 361 |
| A0A1D6HCE3 | 26S proteasome non-ATPase regulatory subunit 6-like protein | 41165 | 361 |
| A0A1D6M6G5 | Putative VHS/GAT domain containing family protein | 40830.9 | 361 |
| A4ULE1 | Nitrilase 2 | 38828 | 361 |
| B4FEP8 | Mannose-1-phosphate guanylyltransferase 1 | 39583.9 | 361 |
| B4FQV3 | SGT1 disease resistance protein homolog1 | 40358 | 361 |
| B6T9G3 | Alpha-1,4-glucan-protein synthase 1 | 40904.8 | 361 |
| B6TCM9 | Spermidine synthase 1 | 39428.6 | 361 |
| B6THG0 | Peroxidase | 38066.8 | 361 |
| B7ZZ23 | Carbohydrate-binding-like fold | 39029.3 | 361 |
| C0P439 | UDP-arabinopyranose mutase 3 | 40942.9 | 361 |
| C0P4P7 | Uncharacterized protein | 40356.7 | 361 |
| C0PD02 | Aldose 1-epimerase | 39131.9 | 361 |
| C0PKS1 | Peroxidase | 38911.3 | 361 |
| C4JAC1 | Mannose-1-phosphate guanylyltransferase 1 | 39601 | 361 |
| K7V1K3 | Calcium-dependent lipid-binding (CaLB domain) family protein | 38115.2 | 361 |
| K7VJS8 | Protein SGT1 homolog A | 40084.6 | 361 |
| Q6YDN1 | Nitrilase 2 | 38753.9 | 361 |
| B4FAU6 | Serine/threonine-protein kinase SRK2E | 40803.9 | 362 |
| B4FG73 | Uncharacterized protein | 39125 | 362 |
| B4FJC2 | Replication factor C subunit 5 | 39743.4 | 362 |
| B4FQG7 | Uncharacterized protein | 38556.3 | 362 |
| B4FUK9 | COP9 signalosome complex subunit 5b | 40129.8 | 362 |
| B6T9Q1 | DnaJ subfamily B member 5 | 38780.8 | 362 |
| B6TFI5 | Replication factor C subunit 3 | 39742.4 | 362 |
| B6TFN9 | Galactose-1-phosphate uridyl transferase-like protein | 40125.2 | 362 |
| B6UIP9 | Methyltransferase | 40005 | 362 |
| B8A0E5 | Omega-amidase chloroplastic | 38622.5 | 362 |
| B8A230 | Uncharacterized protein | 40221.8 | 362 |
| C0P2T6 | Glucuronokinase 1 | 39613.2 | 362 |
| A0A096UDX3 | Cobalamin biosynthesis CobW-like protein | 40654.7 | 363 |
| A0A1D6K1B9 | Hydroxyproline-rich glycoprotein family protein | 40605.2 | 363 |
| B4F875 | ATPase 100191268 | 40709.4 | 363 |
| B4FSB2 | Uncharacterized protein | 38088.1 | 363 |
| B4FU63 | Bifunctional UDP-glucose 4-epimerase and UDP-xylose 4-epimerase 1 | 39812.9 | 363 |
| B6TA76 | UDP-glucose 4-epimerase | 39738.8 | 363 |
| B6TEK2 | GroES-like zinc-binding alcohol dehydrogenase family protein | 38847.4 | 363 |
| B6TKU0 | Growth inhibition and differentiation-related protein 88-like protein | 39374.9 | 363 |
| B6TNJ2 | ATPase | 40700.4 | 363 |
| D5FGP0 | SnRK2.5 | 41823.9 | 363 |
| Q84TL6 | Legumin-like protein | 38078.1 | 363 |
| A0A1D6J2U9 | Fatty acid amide hydrolase | 39729.9 | 364 |
| A0A1D6NEC2 | Casein kinase 1-like protein 2 | 40764.3 | 364 |
| A0A1D6PCL1 | Eukaryotic translation initiation factor 2 subunit alpha | 41154 | 364 |
| A0A1D6QCK6 | Mitogen-activated protein kinase kinase 5 | 39473.7 | 364 |
| B4FQX1 | Alpha-1,4-glucan-protein synthase [UDP-forming] | 41230 | 364 |
| B4G039 | UDP-arabinopyranose mutase 3 | 41254.2 | 364 |
| B6SUB8 | Aldose 1-epimerase | 38586.4 | 364 |
| B6T6Q7 | Aspartate carbamoyltransferase 1 | 40409.7 | 364 |
| B6TCJ3 | Uncharacterized protein | 40392.6 | 364 |
| B6TFE4 | Ribokinase | 37509.8 | 364 |
| B6TYL8 | Uncharacterized protein | 38661.8 | 364 |
| C0PA91 | Uncharacterized protein | 39463.4 | 364 |
| C0PKQ6 | Stress-induced protein kinase1 | 41850.1 | 364 |
| C4J935 | Aspartate carbamoyltransferase chloroplastic | 40394.6 | 364 |
| Q5GLJ6 | O-methyltransferase | 39567.1 | 364 |
| Q5I204 | Brain acid soluble protein 1 | 38306.9 | 364 |
| Q6VWG5 | O-methyltransferase | 39566.1 | 364 |
| A0A1D6DWV4 | Nuclear RNA polymerase D2/E2 | 41743.8 | 365 |
| A0A1D6G4S4 | Vesicle-associated protein 2-2 | 40394.2 | 365 |
| A0A1D6H5J2 | Uncharacterized protein | 37756.1 | 365 |
| A0A1D6IUU6 | 2-oxoisovalerate dehydrogenase subunit alpha 2 mitochondrial | 41270.4 | 365 |
| B4FAD4 | Isocitrate dehydrogenase [NAD] subunit, mitochondrial | 39724.2 | 365 |
| B4FRY4 | Uncharacterized protein | 40075.6 | 365 |
| B4FZG4 | Alpha carbonic anhydrase 1 chloroplastic | 39563.5 | 365 |
| B6T6X0 | PMP | 39579.7 | 365 |
| B6TD33 | Nucleic acid binding protein | 41666.4 | 365 |
| B6TJM1 | Isocitrate dehydrogenase [NAD] subunit, mitochondrial | 39907.5 | 365 |
| B6TNA5 | Pyridoxamine 5-phosphate oxidase-related | 39395.3 | 365 |
| K7UTW6 | Plasminogen activator inhibitor 1 RNA-binding protein | 39288.7 | 365 |
| A0A1D6H1E9 | Biotin--protein ligase 1 chloroplastic | 40085.6 | 366 |
| A0A1D6IJ74 | Pectinesterase | 39048.8 | 366 |
| A0A1D6J5D2 | S-adenosyl-L-methionine-dependent methyltransferase superfamily protein | 40232.4 | 366 |
| A0A1D6JZE3 | Mitochondrial succinate-fumarate transporter 1 | 39142.3 | 366 |
| A0A1D6KL30 | Sorbitol dehydrogenase | 39042 | 366 |
| A0A1Q0XIG6 | Pale yellow9 | 40318.7 | 366 |
| A0A317Y8Z2 | Sorbitol dehydrogenase | 38967.8 | 366 |
| B4FNN6 | Uncharacterized protein | 38749.8 | 366 |
| B4FW29 | Haloacid dehalogenase-like hydrolase (HAD) superfamily protein | 38684.8 | 366 |
| B6TZM6 | Acyl-[acyl-carrier-protein] hydrolase | 41002.1 | 366 |
| C0HIY2 | Uncharacterized protein | 40936.1 | 366 |
| C0PBS1 | Lipase-like | 39067.7 | 366 |
| O80413 | Mitochondrial phosphate transporter1 | 38657.6 | 366 |
| Q0QWI2 | Sorbitol dehydrogenase | 39088 | 366 |
| Q5EUD6 | Protein disulfide isomerase7 | 40056.4 | 366 |
| A0A1D6HZI0 | RNA-binding protein CP31B chloroplastic | 40013.4 | 367 |
| A0A1D6ICV6 | Cathepsin B-like cysteine proteinase 3 | 40328.5 | 367 |
| A0A1D6NP18 | Nuclear pore complex protein NUP98A | 40981.3 | 367 |
| B4FZD8 | Protein STRICTOSIDINE SYNTHASE-LIKE 6 | 39455.5 | 367 |
| B4FZV9 | Glycerol-3-phosphate dehydrogenase [NAD(+)] | 40314.2 | 367 |
| B6TMB2 | PDIL2-2-Zea mays protein disulfide isomerase | 40140.3 | 367 |
| B6TSD7 | Peroxidase | 38328.1 | 367 |
| B6TUD3 | Dihydroflavonol-4-reductase | 39566.8 | 367 |
| B6TUY9 | Chaperone protein dnaJ 49 | 41731.5 | 367 |
| B6TZU2 | Survival motor neuron containing protein | 40707.9 | 367 |
| B6U7D8 | Cinnamyl alcohol dehydrogenase 2 | 38725.3 | 367 |
| C0HDY1 | mRNA-decapping enzyme-like protein | 39876.7 | 367 |
| C0PNW8 | LysM domain-containing GPI-anchored protein 2 | 38176.3 | 367 |
| C5G5X3 | Geranylgeranyl pyrophosphate synthase1 | 38969.2 | 367 |
| K7U9P8 | Methylthioribose-1-phosphate isomerase | 38621.6 | 367 |
| Q5EUD7 | Protein disulfide isomerase6 | 40122.3 | 367 |
| A0A1D6EVL1 | Putative cysteine protease RD19D | 38990.2 | 368 |
| A0A1D6FKV9 | Malate dehydrogenase | 38581.1 | 368 |
| A0A1D6HJJ4 | Glutamine synthetase root isozyme 3 | 40559.2 | 368 |
| A0A1D6N408 | KDEL-tailed cysteine endopeptidase CEP1 | 40114.4 | 368 |
| B4FPM1 | Uncharacterized protein | 39017.3 | 368 |
| B4FW43 | mRNA-decapping enzyme-like protein | 39897.6 | 368 |
| B4FW61 | PTI1-like tyrosine-protein kinase 3 | 40782.1 | 368 |
| B6T790 | DNA repair protein RAD23-1 | 39874.2 | 368 |
| B6TI04 | Developmentally-regulated GTP-binding protein 1 | 41112.3 | 368 |
| B6TNM0 | Malonyl CoA-acyl carrier protein transacylase | 38616.7 | 368 |
| B6TPF5 | TMPIT-like protein | 41120.6 | 368 |
| B8A398 | Ubiquitin carboxyl-terminal hydrolase 3 | 41802.2 | 368 |
| K7UAQ8 | Putative alcohol dehydrogenase superfamily protein | 40361.6 | 368 |
| K7V945 | EMB1374 isoform 1 | 39563.2 | 368 |
| A0A1D6ELX2 | Aldo-keto reductase/ oxidoreductase | 40785.2 | 369 |
| A0A1D6KE50 | Serine/arginine-rich SC35-like splicing factor SCL30 | 41701.5 | 369 |
| A0A1D6LR33 | Nudix hydrolase 10 | 41163.3 | 369 |
| A0A1D6NUF5 | Mitochondrial outer membrane import complex protein METAXIN | 40954.1 | 369 |
| A0A1D6Q2A0 | Trihelix transcription factor ASR3 | 40157.3 | 369 |
| A0A317YEB5 | Glutamine synthetase root isozyme 2 | 40312.2 | 369 |
| A0A317YK56 | Protein TIC110, chloroplastic | 41730.4 | 369 |
| B4F869 | Proline iminopeptidase | 41552.6 | 369 |
| B6STR0 | Oxidoreductase | 39695.1 | 369 |
| B6T7U2 | Aspartate carbamoyltransferase 1 | 40936.2 | 369 |
| B6TY49 | Octicosapeptide/Phox/Bem1p | 38578.6 | 369 |
| B7ZXD5 | Bifunctional protein FolD 4 chloroplastic | 39548.5 | 369 |
| B8A3K1 | Glutathione S-transferase family protein | 41149.2 | 369 |
| B8A3M0 | Glutamine synthetase | 40201.9 | 369 |
| C0P6B2 | Glyceraldehyde-3-phosphate dehydrogenase-like family protein | 39664.1 | 369 |
| A0A1D6E987 | Aldose 1-epimerase | 40167.2 | 370 |
| A0A1D6I8V3 | Putative transcription factor PosF21 | 39579.8 | 370 |
| B4FGU4 | FKBP12-interacting protein of 37 kDa | 40815.5 | 370 |
| B4FMX6 | Thioredoxin reductase | 38522.2 | 370 |
| B4FPY9 | DNAJ heat shock N-terminal domain-containing protein | 41940.7 | 370 |
| B6TD20 | BRASSINOSTEROID INSENSITIVE 1-associated receptor kinase 1 | 40779.8 | 370 |
| B6THA4 | Oxidoreductase | 40093.6 | 370 |
| B6TWC0 | Oxidoreductase | 40068.6 | 370 |
| B7FBH2 | Deoxyhypusine synthase | 40207.4 | 370 |
| B8A036 | Uncharacterized protein | 40219.9 | 370 |
| C4JA79 | Uncharacterized protein | 41692.1 | 370 |
| A0A1D6DVH2 | Galactose mutarotase-like superfamily protein | 41625.7 | 371 |
| A0A1D6FBG5 | La-related protein 1A | 42439.7 | 371 |
| A0A1D6JT16 | ATP synthase subunit gamma mitochondrial | 40008.4 | 371 |
| A0A1D6LH52 | Serine/arginine-rich splicing factor RS2Z32 | 41317.1 | 371 |
| A0A317Y8V3 | Peroxisomal nicotinamide adenine dinucleotide carrier | 40937.6 | 371 |
| A0A317YH91 | Uncharacterized protein | 41083.8 | 371 |
| B4F8Z9 | Uncharacterized protein | 39615.1 | 371 |
| B4FDK7 | Tetratricopeptide repeat (TPR)-like superfamily protein | 41773.2 | 371 |
| B4FPJ1 | Protein transporter | 42149.9 | 371 |
| B4FQZ7 | Methyltransferase | 41295 | 371 |
| B4FX40 | Cysteine proteinase 1 | 40302.8 | 371 |
| B6T8D4 | 1-acyl-sn-glycerol-3-phosphate acyltransferase zeta | 42756.4 | 371 |
| B6TE54 | Uncharacterized protein | 39760.1 | 371 |
| B6TFF6 | Enoyl-[acyl-carrier-protein] reductase [NADH] | 39177.3 | 371 |
| B6TIL4 | GDP-mannose 3,5-epimerase 2 | 41992.6 | 371 |
| C0HHJ4 | Phosphoglycerate kinase | 38388.7 | 371 |
| C0P654 | SPFH/Band 7/PHB domain-containing membrane-associated protein family | 42557.6 | 371 |
| K4JE81 | BZIP-type transcription factor (Fragment) | 39348.7 | 371 |
| K7U9F9 | GDP-mannose 3,5-epimerase 2 isoform 1 | 42042.7 | 371 |
| Q49HE0 | 12-oxo-phytodienoic acid reductase | 40853.8 | 371 |
| Q70L71 | NADPH-protochlorophyllide oxidoreductase (Fragment) | 39777.1 | 371 |
| A0A096SG60 | Putative low-specificity L-threonine aldolase 1 | 39514 | 372 |
| A0A1D6JJP0 | GroES-like zinc-binding alcohol dehydrogenase family protein | 39461 | 372 |
| A0A1D6KVC3 | Putative prolyl 4-hydroxylase 12 | 41207.4 | 372 |
| A0A1D6N9H2 | Phosphoglycerate kinase | 38833.3 | 372 |
| B4FAR4 | RNA polymerase I subunit 43 | 41687.4 | 372 |
| B4FPH9 | Small G protein family protein / RhoGAP family protein | 41155.5 | 372 |
| B6SJW9 | NAD(P)-binding Rossmann-fold superfamily protein | 40768.2 | 372 |
| B6T484 | Mitogen-activated protein kinase | 42332.8 | 372 |
| B6TK50 | Armadillo/beta-catenin-like repeat family protein | 40687.6 | 372 |
| B8A143 | Fes1A | 40657.6 | 372 |
| C0HHC3 | GDSL esterase/lipase | 39665.4 | 372 |
| C0HJ88 | 2-oxoglutarate (2OG) and Fe(II)-dependent oxygenase superfamily protein | 40492.4 | 372 |
| C0PH23 | Uncharacterized protein | 40074.7 | 372 |
| C0PHP8 | Agmatine deiminase | 41637.9 | 372 |
| C4IZ94 | Ornithine carbamoyltransferase chloroplastic | 40286.1 | 372 |
| K7US11 | TMPIT-like protein | 41719.1 | 372 |
| A0A1D6F561 | Protein kinase Kelch repeat:Kelch | 39816.6 | 373 |
| A0A1D6GEP2 | NAD(P)-binding Rossmann-fold superfamily protein | 40000.6 | 373 |
| A0A1D6J5G8 | Eukaryotic translation initiation factor 3 subunit H | 42074.6 | 373 |
| A0A1D6MVV3 | Pyridoxal kinase | 40657.3 | 373 |
| A0A1D6P4E8 | Tryptophan synthase beta type 2 | 39887.4 | 373 |
| A0A317Y671 | Phosphoglucan phosphatase DSP4, amyloplastic | 41909.3 | 373 |
| B4F879 | Integral membrane protein | 43441.6 | 373 |
| B4FJN0 | Phosphoglucan phosphatase DSP4 chloroplastic | 41850.2 | 373 |
| B4FYN6 | Uncharacterized protein | 40250.3 | 373 |
| B6T3Y3 | Tetratricopeptide repeat domain 4 | 40837.7 | 373 |
| C0PEE2 | Small G protein family protein / RhoGAP family protein | 41407.7 | 373 |
| C0PK29 | Protein TRIGALACTOSYLDIACYLGLYCEROL 2 chloroplastic | 40621.5 | 373 |
| K7V5I3 | 12-oxo-phytodienoic acid reductase2 | 41592.7 | 373 |
| Q9ZQY3 | Pyruvate dehydrogenase E1 component subunit beta | 39812.2 | 373 |
| A0A1Q0Y7X5 | DIBOA-glucoside dioxygenase BX6 | 41368.7 | 374 |
| B4FDQ2 | Peptidylprolyl isomerase | 41995.8 | 374 |
| B4FLA1 | Indole-3-glycerol phosphate synthase chloroplastic | 40883.6 | 374 |
| B6T4S8 | Small GTP-binding protein domain | 41139.8 | 374 |
| B6TBL9 | Arsenical pump-driving ATPase | 41178.4 | 374 |
| B6TIU7 | Uncharacterized protein | 41418.8 | 374 |
| B6TJH6 | 1-acyl-sn-glycerol-3-phosphate acyltransferase PLS1 | 42570.9 | 374 |
| B6TMM8 | Mitogen-activated protein kinase | 43275.2 | 374 |
| B6TYG2 | Enoyl-[acyl-carrier-protein] reductase [NADH] chloroplastic | 39365.5 | 374 |
| C0HEH4 | O-methyltransferase family protein | 39589.7 | 374 |
| C0P537 | Phospholipid synthesis2 | 42538.9 | 374 |
| C0PNW7 | Protein kinase superfamily protein | 41436.9 | 374 |
| Q9ZQY1 | Pyruvate dehydrogenase E1 component subunit beta | 39962.4 | 374 |
| A0A0A0Z0N3 | Mitogen-activated protein kinase | 42437 | 375 |
| A0A1D6DX47 | Heterogeneous nuclear ribonucleoprotein U-like protein 1 | 41667.1 | 375 |
| A0A1D6F2Q3 | Transmembrane protein 115 | 41504.9 | 375 |
| A0A1D6GMZ7 | SH3 domain-containing protein 2 | 41925.4 | 375 |
| A0A1D6GZW9 | Transducin family protein / WD-40 repeat family protein | 41346.6 | 375 |
| A0A1D6I8P6 | Inositol 134-trisphosphate 5/6-kinase 4 | 41384.3 | 375 |
| A0A1D6L823 | Actin cross-linking protein | 40972.9 | 375 |
| A0A1D6MTH3 | Uncharacterized protein | 40111.8 | 375 |
| A0A317Y153 | Heavy metal-associated isoprenylated plant protein 6 | 38697.7 | 375 |
| B4FNJ1 | Protein kinase superfamily protein | 41046.1 | 375 |
| B4FRW8 | Uncharacterized protein | 40335.4 | 375 |
| B6T5E9 | BRASSINOSTEROID INSENSITIVE 1-associated receptor kinase 1 | 41030.1 | 375 |
| B6TC55 | L-allo-threonine aldolase | 39815.2 | 375 |
| B6U926 | E3 ubiquitin-protein ligase RING1-like | 39212 | 375 |
| C0PBS0 | Uncharacterized protein | 40515.5 | 375 |
| Q49HE3 | 12-oxo-phytodienoic acid reductase | 41665.7 | 375 |
| A0A060D8V5 | Orphans transcription factor (Fragment) | 41018.1 | 376 |
| A0A1D6FLF3 | Plasminogen activator inhibitor 1 RNA-binding protein | 40588.2 | 376 |
| A0A1D6HSW5 | Syntaxin 132 | 41772.6 | 376 |
| A0A1D6JXF6 | Clathrin binding protein | 42316 | 376 |
| A0A1D6QK03 | Myosin heavy chain-related protein | 42703.6 | 376 |
| A0A317Y326 | Uncharacterized protein | 41147.3 | 376 |
| B4F872 | 12-oxo-phytodienoic acid reductase1 | 41900.9 | 376 |
| B4FHI1 | Uncharacterized protein | 40485.1 | 376 |
| B6SZ83 | Actin-7 | 41633.3 | 376 |
| B6T5K6 | Actin-1 | 41622.2 | 376 |
| B6TQ29 | Uncharacterized protein | 40612.3 | 376 |
| B6TRR5 | Formate dehydrogenase, mitochondrial | 41419 | 376 |
| C0P848 | Formate dehydrogenase, mitochondrial | 41434.1 | 376 |
| K7V367 | Fructose-16-bisphosphatase cytosolic | 41111.9 | 376 |
| A0A1D6GGL9 | Retrovirus-related Pol polyprotein LINE-1 | 40840.4 | 377 |
| A0A1D6GUH3 | Xyloglucan endotransglucosylase/hydrolase | 41785.3 | 377 |
| A0A1D6JV54 | Coatomer subunit gamma | 41550.5 | 377 |
| A0A1D6NZW7 | Uncharacterized protein | 43035.4 | 377 |
| A0A317YDI1 | Uncharacterized protein | 39898 | 377 |
| B4FK44 | 4-hydroxy-tetrahydrodipicolinate synthase 2 chloroplastic | 41148.8 | 377 |
| B4FMY6 | V-type proton ATPase subunit C | 42707.1 | 377 |
| B4FSJ1 | V-type proton ATPase subunit C | 42663.1 | 377 |
| B4FVB1 | Actin-7 | 41669.4 | 377 |
| B4FX81 | Uncharacterized protein | 40681.2 | 377 |
| B4G0U0 | V-type proton ATPase subunit C | 42620.1 | 377 |
| B4G1E1 | Uncharacterized protein | 42008.6 | 377 |
| B6TJD7 | Isocitrate dehydrogenase subunit 1 | 40538.3 | 377 |
| B6TLR1 | Vignain | 40616.4 | 377 |
| B6TN50 | Uncharacterized protein | 41238.4 | 377 |
| B6TQ08 | Actin-1 | 41796.5 | 377 |
| B6TW18 | Lysine and histidine specific transporter | 42746.9 | 377 |
| B6UHC9 | Uncharacterized protein | 41194.8 | 377 |
| C0HHR4 | Actin-2 | 41711.3 | 377 |
| C0PEZ1 | Protein kinase superfamily protein | 41134 | 377 |
| C0PPD7 | Protein arginine N-methyltransferase PRMT10 | 42373.8 | 377 |
| K7UXU1 | HXXXD-type acyl-transferase family protein | 40159.5 | 377 |
| A0A1D6J2I9 | Zn-finger, RanBP-type, containing protein | 40080.8 | 378 |
| A0A1D6LZ74 | Protein disulfide isomerase7 | 41527.1 | 378 |
| B4FBH4 | Import inner membrane translocase subunit TIM50 | 42503.7 | 378 |
| B4FR90 | Hyaluronan / mRNA binding family | 40462.6 | 378 |
| B6T3R0 | Uncharacterized protein | 41786.7 | 378 |
| B6T8U0 | Plasminogen activator inhibitor 1 RNA-binding protein | 40432.7 | 378 |
| K7UYS3 | Uncharacterized protein | 41728.6 | 378 |
| A0A096T0J9 | Protein CDC73-like protein | 42253.6 | 379 |
| A0A1D6HZH7 | Protein TRIGALACTOSYLDIACYLGLYCEROL 3 chloroplastic | 41474.1 | 379 |
| A0A1D6JS55 | SURF1-like protein | 42171.8 | 379 |
| A0A1D6KLB3 | NADPH HC toxin reductase | 40765.2 | 379 |
| A0A1R3MK79 | Inositol 3-kinase | 39897.7 | 379 |
| A0A1R3Q5L9 | Elongation factor Ts, mitochondrial | 41213.9 | 379 |
| B4FFU8 | GDSL esterase/lipase | 40765.1 | 379 |
| B4FKK9 | Uncharacterized protein | 42746.9 | 379 |
| B4FS59 | Zinc ion binding | 41087.1 | 379 |
| B6TBW5 | Tyrosyl-tRNA synthetase | 42724.9 | 379 |
| B6THM0 | Flap endonuclease 1 | 42361.4 | 379 |
| K7UEP7 | Speckle-type POZ protein (Fragment) | 41847.8 | 379 |
| Q41767 | Adh2-N protein | 40537.3 | 379 |
| Q43264 | Alcohol dehydrogenase 1 | 40931 | 379 |
| A0A1D6EM77 | Lecithin-cholesterol acyltransferase-like 1 | 42378.3 | 380 |
| A0A317YJJ5 | SH3 domain-containing protein 1 | 42647.4 | 380 |
| B4F8D4 | Dihydrodipicolinate synthase 2 | 41215.8 | 380 |
| B4FQA2 | Uncharacterized protein | 40696.5 | 380 |
| B4FRC6 | Uncharacterized protein | 38440.4 | 380 |
| B4FVH4 | Uncharacterized protein | 41783.5 | 380 |
| B6T588 | GDP-mannose 3,5-epimerase 1 | 42956.5 | 380 |
| B6UB59 | Homoserine kinase | 38338.3 | 380 |
| B6UI69 | Acyl-desaturase | 42448 | 380 |
| Q6XZP7 | Fertilization-independent type 1 | 42796.2 | 380 |
| Q84UX6 | Nucleosome assembly protein 1 | 43092.5 | 380 |
| A0A1D6J5C4 | Acyl-[acyl-carrier-protein] desaturase | 43781.4 | 381 |
| A0A1D6LSD9 | Transducin/WD40 repeat-like superfamily protein | 41393.3 | 381 |
| A0A1D6P8D3 | Vacuolar protein sorting-associated protein 52 A | 43237.4 | 381 |
| A0A1D6PF22 | 26S proteasome non-ATPase regulatory subunit 4 | 40160.8 | 381 |
| B4FL07 | Protein RETICULATA chloroplastic | 40243.9 | 381 |
| B4FYG1 | Probable tRNA N6-adenosine threonylcarbamoyltransferase | 41134.8 | 381 |
| B6T6Q8 | S-(hydroxymethyl)glutathione dehydrogenase | 40809.9 | 381 |
| B6T9L9 | Uncharacterized protein | 41926.9 | 381 |
| B6TBH3 | Probable tRNA N6-adenosine threonylcarbamoyltransferase | 41121.8 | 381 |
| B6TD64 | Ferredoxin--NADP reductase, chloroplastic | 42156.9 | 381 |
| B6TRH0 | Ferredoxin--NADP reductase, chloroplastic | 41826.1 | 381 |
| B6TT94 | Uncharacterized protein | 40452.1 | 381 |
| A0A1D6EHH9 | Protein TPLATE | 40776.5 | 382 |
| A0A1D6GG86 | Uncharacterized protein | 42248.7 | 382 |
| A0A1D6JS91 | UPF0496 protein | 42487.1 | 382 |
| A0A1D6LGG5 | Jasmonate-regulated gene 21 | 41731.9 | 382 |
| A0A1D6LMU3 | Uncharacterized protein | 42386.1 | 382 |
| A0A1D6MUY8 | Protein kinase superfamily protein | 43929.8 | 382 |
| A0A1D6PY63 | Auxin-responsive family protein | 38278.9 | 382 |
| A0A1D6Q704 | 2-C-methyl-D-erythritol 2,4-cyclodiphosphate synthase | 41175.8 | 382 |
| B4FN28 | Ribosomal protein | 41374.8 | 382 |
| B4FT43 | HB transcription factor | 42407.2 | 382 |
| B6TI71 | Splicing factor 45 | 42013.6 | 382 |
| A0A1D6EYB9 | Putative ADP-ribosylation factor GTPase-activating protein AGD5 | 41516.6 | 383 |
| A0A1D6F2F7 | Proteasome endopeptidase complex | 42222.4 | 383 |
| A0A1D6PKT6 | Blue fluorescent1 | 40028 | 383 |
| B4FI85 | Cytidyltransferase isoform 1 | 42439.3 | 383 |
| B4FMA8 | RNA-binding (RRM/RBD/RNP motifs) family protein | 39776.4 | 383 |
| B4FTP4 | Alcohol dehydrogenase-like 2 | 40907.2 | 383 |
| B6T565 | Pyruvate dehydrogenase E1 component subunit beta-3 chloroplastic | 41354.3 | 383 |
| B6T5X6 | PDIL5-3-Zea mays protein disulfide isomerase | 42449.9 | 383 |
| B6TGC0 | Casein kinase II subunit alpha-2 | 44155.6 | 383 |
| B6TJ48 | Uncharacterized protein | 42258.2 | 383 |
| B6TRX4 | Deaminase | 42753.8 | 383 |
| C0HDV1 | Alpha/beta-Hydrolases superfamily protein | 42217.2 | 383 |
| C0HFW9 | Putative nucleolin-like family protein | 41197.8 | 383 |
| A0A1D6LR91 | Protein N-terminal asparagine amidohydrolase family protein | 42401.8 | 384 |
| A0A1D6QTM3 | Peroxisomal membrane protein PEX16 | 42857.4 | 384 |
| A0A1P8SET2 | BX13 | 42412.9 | 384 |
| B4FK80 | Uncharacterized protein | 42821.6 | 384 |
| B4FTN5 | Metal-dependent protein hydrolase | 42694.9 | 384 |
| B4FU96 | Endoplasmic reticulum vesicle transporter protein | 43339.9 | 384 |
| B4G038 | Homoserine dehydrogenase | 40593.2 | 384 |
| B6T5H2 | Alcohol dehydrogenase 1 | 40782.8 | 384 |
| B6T785 | Dcp1-like decapping family protein | 41526.4 | 384 |
| B6TBU1 | 1-aminocyclopropane-1-carboxylate oxidase | 42453.9 | 384 |
| B6TGB9 | Heterogeneous nuclear ribonucleoprotein A3 | 39294.2 | 384 |
| B6TN48 | 3-hydroxyisobutyryl-CoA hydrolase-like protein 5 | 42876.4 | 384 |
| B6TY37 | Uncharacterized protein | 43272.1 | 384 |
| B7ZX66 | Uncharacterized protein | 43002.1 | 384 |
| C0P8S9 | RNA-binding (RRM/RBD/RNP motifs) family protein | 39135.1 | 384 |
| C0PBM5 | Magnesium transporter MRS2-2 | 42436.4 | 384 |
| K7W6D2 | Splicing factor 45 | 41923.4 | 384 |
| B4FQ29 | 26S proteasome non-ATPase regulatory subunit 13 homolog A | 43842.2 | 385 |
| B6STS8 | Alpha-L-fucosidase 2 | 41436.8 | 385 |
| B6T7M2 | RHC1A | 39732.5 | 385 |
| B6UAD0 | 26S proteasome non-ATPase regulatory subunit 13 | 43790.8 | 385 |
| A0A1D6IHN6 | Tubulin alpha chain | 42288.8 | 386 |
| A0A1D6JW43 | Calcium-binding EF-hand family protein | 41667.7 | 386 |
| A0A1D6QU03 | Uncharacterized protein | 42259.7 | 386 |
| A0A317Y4Q6 | Serine/threonine-protein kinase SAPK3 | 43826.9 | 386 |
| B1P123 | TRIBOA-glucoside O-methyltransferase BX7 | 42130.3 | 386 |
| B4FZ37 | Myelin-associated oligodendrocyte basic protein isoform 1 | 40833.2 | 386 |
| A0A1D6IKN8 | Pectin acetylesterase | 42569.7 | 387 |
| A0A1D6N0H4 | Protein MITOFERRINLIKE 1 chloroplastic | 39478.9 | 387 |
| B4F871 | Protein DJ-1 homolog D | 41208.9 | 387 |
| B4G178 | ADP,ATP carrier protein | 42391.5 | 387 |
| B6TD97 | Uncharacterized protein | 41228.1 | 387 |
| B6TFA0 | D-amino acid oxidase | 40289.9 | 387 |
| B6TIJ4 | Glucose-6-phosphate/phosphate translocator 2 | 42278.5 | 387 |
| B6TQY2 | Serine/threonine-protein phosphatase 2A activator | 41944.6 | 387 |
| B6U900 | Glutathione transferase | 42698.7 | 387 |
| B8A090 | ADP,ATP carrier protein 2, mitochondrial | 42332.3 | 387 |
| C0PM35 | Uncharacterized protein | 41403 | 387 |
| C4JA40 | Phosphoribosylformylglycinamidine cyclo-ligase | 40910.2 | 387 |
| K7U163 | PTI1-like tyrosine-protein kinase 3 | 42200 | 387 |
| K7V5C5 | Spermine synthase | 42146.7 | 387 |
| O64909 | Glucose-6-phosphate/phosphate translocator 2 | 42217.3 | 387 |
| Q9T0N7 | Phytase1 | 41491 | 387 |
| A0A1D6ELW4 | Nucleoside N-ribohydrolase 3 | 42370.2 | 388 |
| A0A1D6HFP3 | Uncharacterized protein | 43623.3 | 388 |
| A0A1D6IK82 | Uncharacterized protein | 42676.1 | 388 |
| A0A1D6IUK2 | Ubiquitin carboxyl-terminal hydrolase 13 | 45796.4 | 388 |
| A0A1D6QSE1 | Porphobilinogen deaminase chloroplastic | 42240.2 | 388 |
| B4FEH1 | Putative fructokinase-6 chloroplastic | 41227.5 | 388 |
| B6T2U2 | Fructokinase-2 | 41177.5 | 388 |
| B6T9G1 | 26S proteasome non-ATPase regulatory subunit 6-like protein | 44067.2 | 388 |
| B6U5D1 | ATP-dependent Clp protease proteolytic subunit | 42824.5 | 388 |
| K7USI8 | Polyneuridine-aldehyde esterase | 42188.8 | 388 |
| Q6R9D5 | Cytochrome b | 43550.7 | 388 |
| A0A1D6IP15 | Actin-related protein 2 | 44006.2 | 389 |
| A0A1D6NUR2 | 2-oxoglutarate (2OG) and Fe(II)-dependent oxygenase superfamily protein | 42472.7 | 389 |
| B4FFZ6 | 60S ribosomal protein L3 | 44577.9 | 389 |
| B4FH48 | Mitochondrial transcription termination factor family protein | 43057.7 | 389 |
| B4FSN4 | Regulator of chromosome condensation3 | 41873.8 | 389 |
| B4FVL1 | 26S proteasome non-ATPase regulatory subunit 6-like protein | 44270.5 | 389 |
| C0PH06 | Peptidyl-prolyl cis-trans isomerase CYP40 | 42752.7 | 389 |
| C4J1C6 | Copine (Calcium-dependent phospholipid-binding protein) family | 42927 | 389 |
| Q304Z5 | Spermidine synthase 2 | 42517.3 | 389 |
| Q9ZRQ5 | Phytase2 | 41662.2 | 389 |
| A0A1D6GHY7 | Deoxyhypusine synthase | 42986.6 | 390 |
| A0A1D6IAU9 | Transcription initiation factor TFIID subunit 15 | 43276.1 | 390 |
| A0A1D6INV7 | Zinc finger BED domain-containing protein DAYSLEEPER | 43639.1 | 390 |
| A0A1D6L5V0 | Serine/threonine-protein kinase SAPK8 | 44099.6 | 390 |
| B6TAW2 | Chaperone protein dnaJ 10 | 43632 | 390 |
| B6TP75 | Pyruvate dehydrogenase E1 component subunit alpha | 42687.2 | 390 |
| C4IZE2 | LisH/CRA/RING-U-box domains-containing protein | 43960.4 | 390 |
| A0A1D6GAU9 | Pentatricopeptide (PPR) repeat protein-like | 42187.8 | 391 |
| A0A1D6KLE3 | PWWP domain protein | 42439.5 | 391 |
| B4FLS8 | 12-oxo-phytodienoic acid reductase5 | 43489 | 391 |
| B4FZK6 | Methionine aminopeptidase | 43668.7 | 391 |
| B6SKY5 | Flavin-containing monooxygenase | 42687.6 | 391 |
| B6T5F3 | Protein transporter | 43417.5 | 391 |
| B6TDI9 | Methionine aminopeptidase | 43654.7 | 391 |
| B6U1X3 | Uncharacterized protein | 43115.2 | 391 |
| B7ZZU4 | Uncharacterized protein | 41803.5 | 391 |
| C0P9G0 | Vesicle-associated protein 2-2 | 43482.7 | 391 |
| C0PIH1 | p-loop containing nucleoside triphosphate hydrolase superfamily protein | 43575.5 | 391 |
| C0PIW6 | Uncharacterized protein | 43981.5 | 391 |
| Q7XJ90 | NAM-related protein 1 | 42071.9 | 391 |
| A0A1D6FRI4 | ERBB-3 BINDING PROTEIN 1 | 43092.1 | 392 |
| A0A1D6NK45 | Elongation factor Ts, mitochondrial | 42581.4 | 392 |
| A0A1D6PW76 | Putative RNA polymerase II transcription factor B subunit 1-1 | 44571 | 392 |
| B6UI91 | Pyruvate dehydrogenase E1 component subunit alpha | 43161 | 392 |
| K7U2K6 | Acyl-[acyl-carrier-protein] desaturase | 44700.6 | 392 |
| Q3HUP7 | Putative stearoyl-acyl-carrier protein desaturase | 44490.3 | 392 |
| Q9ZQY0 | Pyruvate dehydrogenase E1 component subunit alpha | 42915.5 | 392 |
| A0A1D6FQ51 | WD repeat-containing protein 55 | 42803.9 | 393 |
| A0A1D6NJ87 | Uroporphyrinogen decarboxylase | 43461.7 | 393 |
| A0A317YFG5 | Malonyl-CoA-acyl carrier protein transacylase, mitochondrial | 41413 | 393 |
| B4FBA5 | SEC12-like protein 2 | 41937.2 | 393 |
| B4FTI4 | Protein BRE | 43848.8 | 393 |
| B4FUE0 | Obg-like ATPase 1 | 44242.6 | 393 |
| B6SLF6 | Antimicrobial peptide MBP-1 | 45996 | 393 |
| B6STS0 | Serine/threonine-protein kinase SAPK1 | 44476.8 | 393 |
| B6T4P4 | Membrane-associated salt-inducible protein | 43173.3 | 393 |
| B6T4Q5 | Pro-resilin | 42682.9 | 393 |
| B6TCV1 | Sec12-like protein 2 | 41923.3 | 393 |
| C0PG65 | Uncharacterized protein | 44338.6 | 393 |
| K7WHC8 | Blue fluorescent1 | 41213.4 | 393 |
| A0A1D6DX30 | Formation of crista junctions protein 1 | 43558.3 | 394 |
| A0A1D6H6Z3 | Dihydrolipoamide acetyltransferase component of pyruvate dehydrogenase complex | 42461.1 | 394 |
| A0A1D6IMH7 | Hydroxyproline-rich glycoprotein family protein | 44032 | 394 |
| A0A1D6JQQ6 | Leucine-rich transmembrane protein kinase1 | 44287.5 | 394 |
| A0A1D6MNR8 | Zinc ion binding | 43210.8 | 394 |
| A0A1D6PCS6 | Protein PMR5 | 43475.6 | 394 |
| A0A317Y4M5 | Uncharacterized protein | 43711.7 | 394 |
| A0A317YJN8 | Malate dehydrogenase | 41029.7 | 394 |
| B4FD03 | Aldolase superfamily protein | 42904.3 | 394 |
| B4FEH9 | Uncharacterized protein | 43599.9 | 394 |
| B4FIE9 | S-adenosylmethionine synthase | 42772.2 | 394 |
| B4FIR6 | Uncharacterized protein | 43648.6 | 394 |
| B6TBW4 | ERBB-3 BINDING PROTEIN 1 | 43231.3 | 394 |
| B6TCZ3 | Malate dehydrogenase | 41133.9 | 394 |
| B6TMW7 | Transaminase/ transferase, transferring nitrogenous groups | 43530.7 | 394 |
| B6TQB1 | Bifunctional polymyxin resistance arnA protein | 43873.9 | 394 |
| B6TZB7 | Arsenical pump-driving ATPase | 42914.1 | 394 |
| Q5FC14 | Uncharacterized protein ache | 42439.5 | 394 |
| Q6R9G1 | NADH dehydrogenase subunit 7 | 44279.8 | 394 |
| Q9M585 | Stomatin-like protein 2 | 43276.9 | 394 |
| A0A1D6IYK8 | Histone-lysine N-methyltransferase ASHR2 | 42332.8 | 395 |
| A0A1D6NHP1 | MAP kinase kinase kinase55 | 44007.2 | 395 |
| A0A317Y8F8 | GDP-mannose 3,5-epimerase 1 | 44634.4 | 395 |
| B4F8H9 | Uncharacterized protein | 42469.4 | 395 |
| B6UA62 | Cytochrome b561 and DOMON domain-containing protein | 41158 | 395 |
| A0A1D6EKK1 | NAD-dependent dihydrogenase, Gfo/Idh/MocA family | 43381.3 | 396 |
| A0A1D6LKC6 | Aldo/keto reductase AKR1 | 43140.2 | 396 |
| A0A1D6PP96 | Peptide chain release factor APG3 chloroplastic | 44647.6 | 396 |
| A0A317YG24 | Ricin B-like lectin R40C1 | 44267.4 | 396 |
| A0A317YK58 | 3-oxo-Delta(4,5)-steroid 5-beta-reductase | 44170.7 | 396 |
| B4FR08 | Cysteine synthase | 41574.2 | 396 |
| B4FUF3 | Bifunctional polymyxin resistance arnA protein | 43965 | 396 |
| B4FZ87 | Alpha-L-fucosidase 2 | 42679.8 | 396 |
| B6SSF5 | EF-Hand containing protein | 43293.7 | 396 |
| B6TDR4 | NADPH-protochlorophyllide oxidoreductase | 42325.9 | 396 |
| B6TMS8 | Alpha-L-fucosidase 2 | 42612.7 | 396 |
| B6TN88 | Protein brittle-1 | 42844.4 | 396 |
| B6TQ36 | Pyruvate dehydrogenase E1 component subunit beta | 42321.5 | 396 |
| B6UE86 | Fructose-bisphosphate aldolase | 42685.4 | 396 |
| B7ZZ73 | Ribose-phosphate pyrophosphokinase | 42841.9 | 396 |
| C0PIA6 | Farnesylated protein 3 | 41834.6 | 396 |
| P93643 | Phosphate/phosphoenolpyruvate translocator | 42508 | 396 |
| A0A1D6HLQ2 | Bifunctional D-cysteine desulfhydrase/1-aminocyclopropane-1-carboxylate deaminase mitochondrial | 42466.5 | 397 |
| A0A1D6KKD1 | Pyruvate dehydrogenase E1 component subunit beta | 42334 | 397 |
| B4F938 | Necrotic4 | 44112.4 | 397 |
| B4FFV3 | Malate dehydrogenase | 41616.5 | 397 |
| B4FTU3 | Plastid phosphate/phosphoenolpyruvate translocator2 | 42600.1 | 397 |
| B4FVG6 | Pectin acetylesterase | 43112.6 | 397 |
| B4FY50 | Phosphoribosylaminoimidazole-succinocarboxamide synthase | 43549 | 397 |
| B4G1Q6 | Uncharacterized protein | 42173.6 | 397 |
| B6TBI9 | Pyridoxamine 5-phosphate oxidase family protein | 43573.7 | 397 |
| B6TE59 | Hydrolase, NUDIX family protein | 42842.2 | 397 |
| B6TXB0 | Ribosomal protein S24/S35 mitochondrial | 45845.5 | 397 |
| B6TYB9 | Heat shock protein binding protein | 41566 | 397 |
| B8A068 | S-adenosylmethionine synthase | 43072.5 | 397 |
| C0P9T2 | SEC12-like protein 1 | 43310.3 | 397 |
| K4JFG3 | BZIP-type transcription factor (Fragment) | 42317.8 | 397 |
| A0A1D6E5W0 | OSJNBa0089K21.9-like protein | 44937.4 | 398 |
| A0A1D6JPV7 | Ubiquitin carboxyl-terminal hydrolase-related protein | 44597.6 | 398 |
| A0A1D6KNT8 | Putative CCR4-associated factor 1 homolog 11 | 43273.4 | 398 |
| A0A1D6N1K8 | RNA-binding (RRM/RBD/RNP motifs) family protein | 44807.6 | 398 |
| A0A1D6P390 | 26S protease regulatory subunit S10B homolog B | 44514 | 398 |
| A0A317Y930 | SH3 domain-containing protein 2 | 44483.4 | 398 |
| A0A317YH60 | Protein STRICTOSIDINE SYNTHASE-LIKE 3 | 44202.2 | 398 |
| B4FCF0 | Putative nucleoredoxin 1 | 43588.8 | 398 |
| B4FJJ9 | ATP-dependent (S)-NAD(P)H-hydrate dehydratase | 43012.3 | 398 |
| B6SHI6 | Haloacid dehalogenase-like hydrolase domain-containing protein 1A | 43735.7 | 398 |
| B6SRX9 | Fructose-1,6-bisphosphatase | 43270 | 398 |
| B6TRM6 | Pectin acetylesterase | 43434.4 | 398 |
| C0P2Y4 | Uncharacterized protein | 44514.5 | 398 |
| C0PB28 | Uncharacterized protein | 43618.8 | 398 |
| C0PHK1 | Sorting nexin 1 | 46011 | 398 |
| K7TX82 | Transcription factor VIP1 | 42994.2 | 398 |
| K7VAM9 | Mitogen-activated protein kinase | 44932.8 | 398 |
| A0A1D6DYU9 | Peroxisomal (S)-2-hydroxy-acid oxidase GLO1 | 43919.4 | 399 |
| A0A1D6J6I7 | Uncharacterized protein | 44132.2 | 399 |
| A0A1D6L5V1 | SnRK2 serine threonine protein kinase8 | 45192.9 | 399 |
| A0A1D6LDQ9 | GroES-like zinc-binding alcohol dehydrogenase family protein | 42482.5 | 399 |
| A0A1D6N6K8 | Sorting nexin 1 | 46099.1 | 399 |
| B4FCA4 | COP9 signalosome complex subunit 4 | 45173 | 399 |
| B4FQE0 | EC synthetase | 42947.9 | 399 |
| B6TGG5 | AAR2 protein family | 45296 | 399 |
| B6TJC5 | Developmentally-regulated GTP-binding protein 2 | 44578 | 399 |
| B6TL91 | FAM10 family protein | 43386.2 | 399 |
| C0HFH4 | Pyridoxal phosphate (PLP)-dependent transferase superfamily protein | 44142.1 | 399 |
| K7TFF5 | Defective18 | 43631.7 | 399 |
| Q49HD7 | 12-oxo-phytodienoic acid reductase8 | 44220.5 | 399 |
| A0A1D6E5U7 | Protein RETICULATA chloroplastic | 42444.3 | 400 |
| A0A1D6FJF0 | Farnesyl pyrophosphate synthase1 | 45136.2 | 400 |
| A0A317Y8G1 | SURF1-like protein | 45042.4 | 400 |
| B4FTV9 | 26S protease regulatory subunit S10B | 44570.8 | 400 |
| C0PGM6 | 26S protease regulatory subunit S10B homolog B | 44515.7 | 400 |
| C0PI49 | Prefoldin chaperone subunit family protein | 42837.6 | 400 |
| Q8LK06 | Methyl binding domain protein MBD109 | 42122 | 400 |
| A0A1D6G2H2 | Alpha/beta-Hydrolases superfamily protein | 44394 | 401 |
| A0A1D6H2J0 | Protein phosphatase 2C isoform gamma | 43791.2 | 401 |
| A0A1D6KFP0 | Tetratricopeptide repeat (TPR)-like superfamily protein | 44547.5 | 401 |
| A0A1D6M1Z9 | Uncharacterized protein | 43478.5 | 401 |
| A0A1D6MFW6 | Uncharacterized protein | 40755.7 | 401 |
| B4F9B2 | Acetyl-CoA acetyltransferase, cytosolic 1 | 41187.9 | 401 |
| B4FQ91 | Branched-chain-amino-acid aminotransferase | 43464.7 | 401 |
| B4FSN1 | Thiolase1 | 41121.9 | 401 |
| B6SM26 | 3-oxoacyl-synthase III, mRNA | 41573.2 | 401 |
| B6TCV9 | LanC-like protein 2 | 44456.4 | 401 |
| B6U6Z4 | Dihydroorotase | 44515.8 | 401 |
| K7VDH6 | RNA-binding (RRM/RBD/RNP motifs) family protein | 41594.6 | 401 |
| K7VIW2 | GPN-loop GTPase | 44535.5 | 401 |
| A0A1D6K8W1 | Dynamin-related protein 1E | 45628.4 | 402 |
| A0A1D6KI18 | Elongator complex protein 2 | 44558.2 | 402 |
| A0A1D6QLT9 | DNA polymerase delta catalytic subunit | 46010.7 | 402 |
| B4FAI5 | Peptidase C13 family | 45135.8 | 402 |
| B4FBY6 | Uncharacterized protein | 45000.3 | 402 |
| B4G0K4 | Phosphoglycerate kinase | 42438.8 | 402 |
| B6T0Y0 | Ser/Thr protein phosphatase family | 45479.9 | 402 |
| B6T711 | DNA repair protein RAD23 | 42052.7 | 402 |
| B6TJV6 | AT-hook motif nuclear-localized protein 13 | 40478.1 | 402 |
| B6TRD5 | Uncharacterized protein | 43808.6 | 402 |
| C0HHL1 | Uncharacterized protein | 42756.8 | 402 |
| O04288 | Endosperm specific protein | 43134.9 | 402 |
| A0A1D6FV23 | Tetratricopeptide repeat (TPR)-like superfamily protein | 44967.9 | 403 |
| A0A317Y206 | Serine--glyoxylate aminotransferase | 44119.3 | 403 |
| B4F9T4 | Uncharacterized protein | 43941.7 | 403 |
| B6SJK3 | mTERF family protein | 45201.6 | 403 |
| C0HES6 | Arogenate dehydrogenase 1 chloroplastic | 43911.1 | 403 |
| C0P455 | 60S ribosomal protein L4-1 | 44292.2 | 403 |
| C0PFK1 | GDSL esterase/lipase EXL3 | 42791.7 | 403 |
| A0A1D6DTS1 | Uncharacterized protein | 43328 | 404 |
| A0A1D6J6K7 | Calcineurin-like metallo-phosphoesterase superfamily protein | 45156.9 | 404 |
| A0A317Y6Y3 | Serine/threonine-protein kinase HT1 | 45094 | 404 |
| A0A317YEG7 | 60S ribosomal protein L4-1 | 44347.2 | 404 |
| B0ZBJ2 | MAP kinase kinase | 44508.2 | 404 |
| B4FVJ0 | 60S ribosomal protein L4-1 | 44411.3 | 404 |
| B6T3L6 | 60S ribosomal protein L4 | 44325 | 404 |
| B6T9S3 | Protein-lysine N-methyltransferase | 44306.4 | 404 |
| B6TB55 | 4-diphosphocytidyl-2-C-methyl-D-erythritol kinase | 43890 | 404 |
| B6TF70 | LisH/CRA/RING-U-box domains-containing protein | 45425.7 | 404 |
| B6THM1 | Uncharacterized protein | 43302.8 | 404 |
| C0PLS6 | Uncharacterized protein | 42638.4 | 404 |
| K7UBA1 | Protein AUXIN RESPONSE 4 | 42240.8 | 404 |
| A0A1D6EPW8 | ATP-dependent 6-phosphofructokinase 5 chloroplastic | 44079.1 | 405 |
| A0A1D6HTG4 | Putative ribose-5-phosphate isomerase 3 chloroplastic | 43502 | 405 |
| A0A1D6N7Q6 | Nucleic acid binding protein | 46234.5 | 405 |
| A0A1D6PP97 | Peptide chain release factor APG3 chloroplastic | 45424.4 | 405 |
| A0A1D6Q7L5 | Survival motor neuron protein | 45298.6 | 405 |
| B4FIJ9 | Protein kinase superfamily protein | 44685.4 | 405 |
| B4FSJ2 | Elongation factor Tu | 43869.1 | 405 |
| B6TCP0 | Protein transporter | 45370.3 | 405 |
| B6TKL2 | DNA repair protein RAD23 | 42575.3 | 405 |
| C0HE93 | Protein ELC-like | 43866.2 | 405 |
| A0A1D6EE47 | Glutathione S-transferase family protein | 45458.7 | 406 |
| A0A1D6HGQ8 | Target of Myb protein 1 | 44987.2 | 406 |
| A0A1D6LEN4 | Succinate dehydrogenase [ubiquinone] iron-sulfur subunit, mitochondrial | 46097 | 406 |
| A0A1D6MQX6 | Magnesium-protoporphyrin IX monomethyl ester [oxidative] cyclase chloroplastic | 47075.8 | 406 |
| A0A1D6NJW0 | 3'-5' exonuclease domain-containing protein / K homology domain-containing protein / KH domain-containing protein | 44907.3 | 406 |
| A0A1D6Q251 | Isoamylase 1 chloroplastic | 45692.5 | 406 |
| B4FTL0 | BAG family molecular chaperone regulator 7 | 46539.5 | 406 |
| B6TPZ9 | Arogenate dehydrogenase isoform 2 | 44199.4 | 406 |
| B6TVG6 | Protein brittle-1 | 43724.4 | 406 |
| C0P488 | Polyadenylate-binding protein RBP45C | 44475.1 | 406 |
| A0A1D6E501 | 3-isopropylmalate dehydrogenase | 42987.7 | 407 |
| A0A1D6KWT9 | Glycerophosphoryl diester phosphodiesterase | 46419.7 | 407 |
| A0A1D6NLI9 | Uncharacterized protein | 45648.1 | 407 |
| A0A1D6P550 | Uncharacterized protein | 44855.2 | 407 |
| A0A317YBF0 | 3-hydroxyisobutyryl-CoA hydrolase-like protein 2, mitochondrial | 44775.6 | 407 |
| B4FJS3 | Protein NETWORKED 4A | 46933.6 | 407 |
| B4FPA5 | NAD(P)-binding Rossmann-fold superfamily protein | 44184.9 | 407 |
| B4FQ64 | Isovaleryl-CoA dehydrogenase | 44309.5 | 407 |
| B4FR07 | Chaperone protein dnaJ 15 | 45546.9 | 407 |
| B6SNX1 | Aminomethyltransferase | 44380.6 | 407 |
| B6TAE1 | NADH-ubiquinone oxidoreductase 39 kDa subunit | 44582 | 407 |
| B6TII3 | ADP-ribosylation factor GTPase-activating protein 3 | 44043.7 | 407 |
| B6TVD6 | 3-isopropylmalate dehydrogenase | 42997.7 | 407 |
| B6TXA2 | RNA-binding protein Luc7-like 2 | 47548.6 | 407 |
| B6U1V2 | Uncharacterized protein | 46876.6 | 407 |
| B6U6X4 | Lariat debranching enzyme | 46773.4 | 407 |
| K7U1I2 | Succinate dehydrogenase [ubiquinone] iron-sulfur subunit, mitochondrial | 46052 | 407 |
| Q2MJJ9 | Putative RH2 protein | 45945.5 | 407 |
| A0A1D6E1K1 | UDP-Glycosyltransferase superfamily protein | 45405.1 | 408 |
| A0A1D6P9D4 | Peptidylprolyl isomerase | 45499 | 408 |
| B4FDF0 | Uncharacterized protein | 44333 | 408 |
| B6TBQ7 | RNA-binding protein Luc7-like 2 | 47875 | 408 |
| B6U746 | Metacaspase type II | 44225.6 | 408 |
| C0PBR6 | Uncharacterized protein | 45240.1 | 408 |
| C0PCX3 | Tryptophan--tRNA ligase cytoplasmic | 46146.4 | 408 |
| K7UR93 | Ubiquitin family protein | 44970.7 | 408 |
| Q0GH73 | CMO protein | 45586 | 408 |
| A0A1D6E503 | SKP1-interacting partner 15 | 44103.5 | 409 |
| A0A1D6E8Y9 | Peroxidase | 43982.1 | 409 |
| A0A1D6ECT6 | Purple acid phosphatase 3 | 45869.7 | 409 |
| A0A1D6KRE0 | Membrane steroid-binding protein 1 | 44011.4 | 409 |
| A0A1D6NGE6 | Transducin/WD40 repeat-like superfamily protein | 44831.3 | 409 |
| B4G1Z5 | Indole-3-glycerol phosphate synthase chloroplastic | 44252.2 | 409 |
| B6TB66 | 50S ribosomal protein L1 | 44340.7 | 409 |
| B6THR9 | 3-isopropylmalate dehydrogenase | 43491.3 | 409 |
| B6TQ06 | Aminomethyltransferase | 44034.1 | 409 |
| B6U0I9 | Oxysterol-binding protein OBPa | 44579.9 | 409 |
| B6U2Y9 | Aldo-keto reductase/ oxidoreductase | 45242.5 | 409 |
| K7VUP6 | 50S ribosomal protein L1 | 44366.8 | 409 |
| Q6B7Q9 | RuBisCo subunit binding-protein beta subunit (Fragment) | 44073.8 | 409 |
| A0A1D6GI01 | Vps51/Vps67 family (Components of vesicular transport) protein | 46103.1 | 410 |
| A0A1D6JFM4 | 3-oxoacyl-synthase III, mRNA | 42706.5 | 410 |
| B4FBK3 | Eukaryotic initiation factor 4A-2 | 46582 | 410 |
| B6U5D5 | Uncharacterized protein | 45511.9 | 410 |
| C0P4P0 | Putative choline kinase 2 | 45970.7 | 410 |
| K7WGQ3 | Multiple organellar RNA editing factor 8 chloroplastic/mitochondrial | 43956.2 | 410 |
| Q8W4W2 | Glutathione synthetase | 45955.2 | 410 |
| A0A1D6HKP7 | Annexin | 45426 | 411 |
| A0A1D6M341 | Carbon catabolite repressor protein 4 homolog 4 | 45813.4 | 411 |
| A0A1D6N1A5 | Villin-2 | 45448.3 | 411 |
| A0A1D6NZT3 | Uncharacterized conserved protein (UCP030210) | 45251.5 | 411 |
| B4F9J5 | Glutamate dehydrogenase | 44068.9 | 411 |
| B4FFP2 | Glutamate dehydrogenase | 44427.4 | 411 |
| B4FI21 | ARM repeat superfamily protein | 47089.7 | 411 |
| B6TRV8 | ARM repeat superfamily protein | 47256.1 | 411 |
| B8A293 | Glyceraldehyde-3-phosphate dehydrogenase | 43425 | 411 |
| C0PDB0 | Phosphoglycerate kinase | 43196.4 | 411 |
| Q43260 | Glutamate dehydrogenase | 44021.9 | 411 |
| A0A1D6EFH2 | AAR2 protein family | 47004.5 | 412 |
| A0A1D6M5G7 | Polypyrimidine tract-binding protein homolog 3 | 45718 | 412 |
| A0A1D6QR46 | Peptidase C13 family | 46344.2 | 412 |
| B4FAK9 | Glyceraldehyde-3-phosphate dehydrogenase | 43320 | 412 |
| B4G0Z1 | PHD finger protein-related | 46023 | 412 |
| B6SJX7 | DAG protein | 44582.6 | 412 |
| B6STR2 | Branched-chain-amino-acid aminotransferase | 44691.4 | 412 |
| B6TA28 | Calreticulin-3 | 47682.3 | 412 |
| B6TKK9 | IQ calmodulin-binding motif family protein | 46116.4 | 412 |
| K7V067 | Isocitrate dehydrogenase [NADP] | 46196.4 | 412 |
| A0A1D6G7E6 | CLP protease regulatory subunit CLPX3 mitochondrial | 43940.2 | 413 |
| A0A1D6HR41 | Pyridoxal 5'-phosphate synthase-like subunit PDX1.2 | 44018.3 | 413 |
| A0A317YEN3 | ATP-dependent DNA helicase | 46478.9 | 413 |
| B4F9X6 | Pectin acetylesterase | 45396.5 | 413 |
| A0A0B4J303 | Eukaryotic initiation factor4a | 46982.4 | 414 |
| A0A1D6H6Y2 | Putative elongation factor 1-gamma 2 | 47044.9 | 414 |
| A0A1D6HNH5 | Neurochondrin family protein | 45343 | 414 |
| A0A1D6K5S9 | Erlin-2 | 47603.6 | 414 |
| B4FSZ8 | Beta alanine synthase1 | 45767.5 | 414 |
| B4FU39 | Uncharacterized protein | 43460.5 | 414 |
| C0PES7 | Diphosphonucleotide phosphatase1 | 43607.8 | 414 |
| C0PHE3 | Eucaryotic initiation factor4 | 46952.4 | 414 |
| K7UQT7 | Putative elongation factor 1-gamma 2 | 46950.9 | 414 |
| A0A1D6I9U6 | Phosphatase DCR2 | 45092.5 | 415 |
| A0A1D6M4H7 | Guanosine nucleotide diphosphate dissociation inhibitor | 46806.1 | 415 |
| B4F987 | Putative sarcosine oxidase | 43946.4 | 415 |
| B4FN97 | Isocitrate dehydrogenase [NADP] | 46251.4 | 415 |
| B4FUA8 | Calreticulin-3 | 48398.8 | 415 |
| B4FZU9 | Dihydropyrimidine dehydrogenase (NADP(+)) chloroplastic | 45673 | 415 |
| B6TIK6 | Sarcosine oxidase | 43815.3 | 415 |
| B6TLZ9 | Glycerophosphoryl diester phosphodiesterase | 47243.4 | 415 |
| B7ZZY5 | Aspartyl protease APCB1 | 44630.6 | 415 |
| C4IYK3 | Polyadenylate-binding protein RBP47B | 46026 | 415 |
| K7TM75 | Carrier protein | 44487.4 | 415 |
| K7VAS4 | Eukaryotic translation initiation factor 3 subunit M | 46358.9 | 415 |
| A0A1D6FD79 | Fructose-bisphosphate aldolase | 45146.4 | 416 |
| A0A1D6FT87 | BAG family molecular chaperone regulator 8 chloroplastic | 45565 | 416 |
| A0A1D6GLS4 | Serpin-Z1 | 44386.2 | 416 |
| A0A1D6KWT7 | Glycerophosphodiester phosphodiesterase GDPD6 | 47340.7 | 416 |
| B4F9F9 | ADP-ribosylation factor GTPase-activating protein AGD10 | 44895.8 | 416 |
| B4FA42 | NagB/RpiA/CoA transferase-like superfamily protein | 44771.5 | 416 |
| B4FAI1 | N-acetyl-gamma-glutamyl-phosphate reductase | 45291.8 | 416 |
| B4FQU2 | NagB/RpiA/CoA transferase-like superfamily protein | 44899.7 | 416 |
| B4FR84 | PP2A regulatory subunit TAP46 | 47052.4 | 416 |
| B4FTJ7 | Putative sarcosine oxidase | 43110.7 | 416 |
| B6TVR5 | PP2A regulatory subunit TAP46 | 47020.4 | 416 |
| C0PMW1 | Glycosyltransferase | 44928 | 416 |
| A0A1D6E1K2 | UDP-Glycosyltransferase superfamily protein | 46371.2 | 417 |
| A0A1D6FEB3 | Synaptic glycoprotein SC2 | 47019.6 | 417 |
| A0A1D6FTE2 | Trehalose-6-phosphate synthase1 | 46639.7 | 417 |
| A0A1D6G7Z3 | Sorting nexin 1 | 48160.6 | 417 |
| A0A1D6GG02 | DnaJ protein | 46561.2 | 417 |
| A0A1D6HCA0 | Actin-related protein 3 | 46496.5 | 417 |
| A0A1D6IPX0 | 3-dehydroquinate synthase chloroplastic | 45211.6 | 417 |
| A0A1D6K425 | Peptidylprolyl isomerase | 46364.2 | 417 |
| B4F9X3 | 5-pentadecatrienyl resorcinol O-methyltransferase | 45060 | 417 |
| B4FHK5 | NPL4 family protein | 45588.7 | 417 |
| B4G1G0 | GroES-like zinc-binding alcohol dehydrogenase family protein | 44200.4 | 417 |
| B6T775 | Glycosyl transferase, group 1 | 46682.8 | 417 |
| B6T7G7 | Elongation factor 1-gamma 3 | 47267.1 | 417 |
| B6TGB2 | Elongation factor 1-gamma 3 | 47158.9 | 417 |
| B6TRS1 | Histidinol-phosphate aminotransferase | 46656.4 | 417 |
| B7ZZX8 | Uncharacterized protein | 45768.7 | 417 |
| C0PDB6 | HXXXD-type acyl-transferase family protein | 46132.6 | 417 |
| C0PGV5 | Indole-3-glycerol phosphate synthase chloroplastic | 44825.8 | 417 |
| A0A1D6F7L1 | SEC12-like protein 2 | 44869.8 | 418 |
| A0A1D6HNF6 | Aspartate aminotransferase chloroplastic | 46349.8 | 418 |
| A0A1D6P149 | RNA-binding (RRM/RBD/RNP motifs) family protein | 43565.9 | 418 |
| B4G027 | Acid phosphatase | 43322 | 418 |
| B4G1X9 | Uncharacterized protein | 46810.9 | 418 |
| B6TWZ8 | PDIL5-2-Zea mays protein disulfide isomerase | 46976.3 | 418 |
| C0P7S8 | Chaperone DNA J homolog1 | 46642.3 | 418 |
| Q5EUD3 | Protein disulfide isomerase10 | 46962.3 | 418 |
| A0A1D6H6Z2 | Acetyltransferase component of pyruvate dehydrogenase complex | 45180.1 | 419 |
| A0A1D6JB12 | Serine/threonine-protein kinase GRIK1 | 47087.6 | 419 |
| A0A1D6NMB1 | Vacuolar protein sorting-associated protein 32 homolog 1 | 44686.9 | 419 |
| B6SZN0 | MtN19-like protein | 45006.6 | 419 |
| B6TKL4 | CHY1 | 46189.4 | 419 |
| B6TPB9 | Pentatricopeptide repeat-containing protein mitochondrial | 45555.6 | 419 |
| A0A1D6DW26 | Uncharacterized protein | 45951.6 | 420 |
| A0A1D6FZF4 | PsbP domain-containing protein 5 chloroplastic | 47180.7 | 420 |
| A0A1D6LYW8 | LisH/CRA/RING-U-box domains-containing protein | 47343.1 | 420 |
| B6TRH7 | 26S protease regulatory subunit 6B-like protein | 46722.9 | 420 |
| B6TVN4 | Phospholipase C | 46042.3 | 420 |
| B6UA69 | Polyprotein | 45879.9 | 420 |
| A0A1D6H1H5 | Porphobilinogen deaminase chloroplastic | 45615.7 | 421 |
| A0A1D6N9H5 | Metacaspase-6 | 45930.1 | 421 |
| B4FK00 | ADP-ribosylation factor GTPase-activating protein AGD10 | 45303.3 | 421 |
| B4FYM0 | Acetyl-CoA acetyltransferase, cytosolic 2 | 43077.9 | 421 |
| B6TV06 | CP5 | 46193.5 | 421 |
| C0P928 | Uncharacterized protein | 44756.4 | 421 |
| K7V238 | Secondary cell wall glycosyltransferase family 47 | 47145.9 | 421 |
| K7V5L1 | Uncharacterized protein | 47391.8 | 421 |
| Q43712 | Calcium-binding protein | 48012.3 | 421 |
| A0A1D6ELY8 | Arogenate dehydratase | 45543.1 | 422 |
| A0A1D6ELZ0 | Nuclear pore complex protein GP210 | 47066.2 | 422 |
| A0A1D6H3F7 | Peptidyl-prolyl cis-trans isomerase CYP95 | 47469.6 | 422 |
| A0A1D6K2B5 | Uroporphyrinogen decarboxylase | 46468.1 | 422 |
| A0A1D6M7I9 | TraB protein | 45747.6 | 422 |
| A0A1D6QHN1 | Putative 2-carboxy-D-arabinitol-1-phosphatase | 44985.4 | 422 |
| B4FJC3 | Chaperone protein dnaJ 3 | 47075 | 422 |
| B4FRH5 | Succinate--CoA ligase [ADP-forming] subunit beta, mitochondrial | 45200.9 | 422 |
| B4G124 | 26S protease regulatory subunit 8 homolog B | 47172.3 | 422 |
| B6STX9 | Serine/threonine-protein kinase NAK | 45476.9 | 422 |
| B6TD54 | ATP-citrate synthase | 46635.3 | 422 |
| B6TF42 | CBS domain containing protein | 46751.8 | 422 |
| B6TS21 | Succinate--CoA ligase [ADP-forming] subunit beta, mitochondrial | 45192.8 | 422 |
| B6U1P3 | Protein CLP1 homolog | 45911.5 | 422 |
| B7ZXS8 | Uncharacterized protein | 46982.8 | 422 |
| B8A063 | Uncharacterized protein | 47186.3 | 422 |
| C0P6U7 | Uncharacterized protein | 46923.2 | 422 |
| C0PDY7 | Temperature sensing protein-related | 46906.6 | 422 |
| A0A1D6DVD2 | Putative UDP-arabinopyranose mutase 5 | 46959 | 423 |
| A0A1D6E9I2 | Pentatricopeptide repeat-containing protein mitochondrial | 46759.3 | 423 |
| A0A1D6FWR6 | Nucleosome assembly protein 1 | 48302.1 | 423 |
| A0A1D6GAV1 | Pentatricopeptide (PPR) repeat protein-like | 46513.6 | 423 |
| A0A1D6HJ21 | Aquaporin SIP1-1 | 46625 | 423 |
| A0A1D6PS72 | F-box protein SKIP16 | 47561.7 | 423 |
| B4FTF4 | CD2-binding protein-related | 46763.2 | 423 |
| B4FWV0 | Mov34/MPN/PAD-1 family protein | 46760.2 | 423 |
| B4G1X1 | Alpha-galactosidase | 45962 | 423 |
| B6TJS7 | Mov34/MPN/PAD-1 family protein | 46732.2 | 423 |
| C0HGC8 | Uncharacterized protein | 46584.4 | 423 |
| C0P676 | SNF1-related protein kinase regulatory subunit gamma-1 | 46564.6 | 423 |
| A0A1D6HGQ5 | Survival motor neuron protein | 45876.1 | 424 |
| A0A1D6MBH7 | Mov34/MPN/PAD-1 family protein | 46963.5 | 424 |
| B4FVV2 | Uncharacterized protein | 47898.4 | 424 |
| C4J056 | Ethanolamine-phosphate cytidylyltransferase | 47204.6 | 424 |
| K7TXD7 | COP9 signalosome complex subunit 3 | 47071.4 | 424 |
| A0A1D6EMD1 | Protein prenylyltransferase superfamily protein | 47725.9 | 425 |
| A0A1D6H167 | Cystathionine beta-synthase (CBS) family protein | 44914.6 | 425 |
| A0A1D6H8W6 | Ubiquitin-activating enzyme E1 domain-containing protein 1 | 46427.1 | 425 |
| A0A317YB81 | Alpha-taxilin | 47405.5 | 425 |
| B4FA27 | Alpha-galactosidase | 46899.9 | 425 |
| B4FBL0 | CAAX prenyl protease 1 | 48749.8 | 425 |
| B4FRI1 | Phosphoserine aminotransferase | 44867.9 | 425 |
| B6TDT6 | Methylthioribose kinase | 48111.4 | 425 |
| B6TS69 | Polygalacturonase inhibitor 2 | 43951.8 | 425 |
| B8A2J4 | TLD-domain containing nucleolar protein | 47238.8 | 425 |
| C0P5X2 | Uncharacterized protein | 47410.5 | 425 |
| A0A1D6DWZ1 | FGGY family of carbohydrate kinase | 45438.4 | 426 |
| A0A1D6GVM3 | Delta-aminolevulinic acid dehydratase | 46121.1 | 426 |
| A0A1D6JMA9 | DNA binding protein | 45327.4 | 426 |
| B4FF65 | 26S proteasome non-ATPase regulatory subunit 11-like protein | 47703.5 | 426 |
| B6T080 | MtN19-like protein | 45586.3 | 426 |
| B6U5I1 | Peptidyl-prolyl cis-trans isomerase | 46639.6 | 426 |
| B7ZWT2 | Uncharacterized protein | 47671.3 | 426 |
| C0PHK8 | Uncharacterized protein | 47527.4 | 426 |
| A0A1D6FHW2 | S-adenosylmethionine synthase | 46233.1 | 427 |
| A0A1D6HQ86 | DEAD-box ATP-dependent RNA helicase 34 | 48529.6 | 427 |
| A0A1D6HXF1 | Zn-dependent exopeptidase superfamily protein | 47020.7 | 427 |
| A0A1D6KJV9 | Sulfite oxidase1 | 47516.6 | 427 |
| A0A1D6NAN1 | Branched-chain-amino-acid aminotransferase 5 chloroplastic | 50118.9 | 427 |
| A0A345VNI9 | Glycerol-3-phosphate dehydrogenase [NAD(+)] | 45717.1 | 427 |
| B4FBI6 | Fumarylacetoacetase | 46688.9 | 427 |
| B4FXE1 | Uncharacterized protein | 46531.3 | 427 |
| B4G181 | Uncharacterized protein | 44881.2 | 427 |
| B6TZ03 | Trypsin family protein with PDZ domain | 45028.3 | 427 |
| B7ZWU3 | Uncharacterized protein | 45837.6 | 427 |
| C0HHJ2 | Uncharacterized protein | 46237.8 | 427 |
| C0PB33 | BSD domain-containing protein | 46382 | 427 |
| A0A1D6EFN9 | Pyruvate dehydrogenase E1 component subunit alpha | 46403.5 | 428 |
| A0A1D6EID3 | PP2A regulatory subunit TAP46 | 48516.3 | 428 |
| A0A1D6JYZ2 | Arogenate dehydratase | 47202.5 | 428 |
| A0A1D6N7Q5 | Nucleic acid binding protein | 48816.4 | 428 |
| A0A317Y6C8 | 12-oxophytodienoate reductase 7 | 47729.5 | 428 |
| A0A317YGT5 | Sulfofructose kinase | 45896.1 | 428 |
| B4FYM6 | Alpha-amylase | 47352.7 | 428 |
| B4G0C7 | General transcription factor IIH subunit | 47316.2 | 428 |
| B4G1P9 | 3-N-debenzoyl-2-deoxytaxol N-benzoyltransferase | 45948 | 428 |
| B6TJM5 | 26S protease regulatory subunit 6A | 47687.8 | 428 |
| B6TNN5 | Alpha-galactosidase | 46449.5 | 428 |
| B6TQC0 | 3-N-debenzoyl-2-deoxytaxol N-benzoyltransferase | 46099.2 | 428 |
| B6TVM7 | 4-methyl-5-thiazole monophosphate biosynthesis protein | 45141.9 | 428 |
| B6TXM6 | 10-deacetylbaccatin III 10-O-acetyltransferase | 45483.7 | 428 |
| B7ZYH3 | Uncharacterized protein | 45642.6 | 428 |
| C0P534 | Uncharacterized protein | 46802.4 | 428 |
| K7U3S1 | Sucrose cleavage protein-like protein | 46571.7 | 428 |
| K7U6V4 | Polyadenylate-binding protein RBP45C | 46653.2 | 428 |
| Q94IQ8 | Methyl binding domain106 | 43302.5 | 428 |
| A0A1D6E267 | Fasciclin-like arabinogalactan protein 10 | 44023.5 | 429 |
| A0A1D6LJL1 | Proteasome subunit beta type | 46157.5 | 429 |
| A0A1D6PQP0 | 14-3-3-like protein GF14 epsilon | 47389.5 | 429 |
| A0A1D6Q4P7 | Polyprotein | 47023.3 | 429 |
| B4FRC9 | Aldolase-type TIM barrel family protein | 46245.2 | 429 |
| B4FTR0 | Chaperone protein dnaJ | 46064.4 | 429 |
| B6TZG5 | Fasciclin-like arabinogalactan protein 10 | 44032.5 | 429 |
| C0PFY3 | AP-1 complex subunit mu-2 | 49000.9 | 429 |
| A0A1D6HBV2 | Uncharacterized protein | 49054.7 | 430 |
| A0A1D6QKI8 | HSP protein | 49658.3 | 430 |
| A0A1X7YE53 | Uncharacterized protein | 45736.1 | 430 |
| A0A317YA81 | La-related protein 6B | 46954.6 | 430 |
| B4F9G1 | Aspartate aminotransferase | 47733.2 | 430 |
| B4G1U6 | Phospholipase A1-IIgamma | 46056.2 | 430 |
| B6SYS8 | Alpha/beta-Hydrolases superfamily protein | 47586.5 | 430 |
| B7ZXK1 | Aspartate aminotransferase | 47742.2 | 430 |
| C0PC61 | Aldolase-type TIM barrel family protein | 46220.2 | 430 |
| C0PNW3 | Magnesium transporter MRS2-1 | 47986.6 | 430 |
| A0A1D6IYG6 | Obg-like ATPase 1 | 48707.9 | 431 |
| A0A1D6MES0 | DNAJ heat shock family protein | 47831.1 | 431 |
| A0A317Y808 | Aspartyl protease 25 | 44651.1 | 431 |
| B4FMW6 | Aspartyl protease AED3 | 44623.1 | 431 |
| B4FP94 | Uncharacterized protein | 47446.7 | 431 |
| B5ATU2 | Tryptophan aminotransferase | 47217.2 | 431 |
| K7U924 | Mevalonate kinase | 44941.9 | 431 |
| Q8W514 | Histone-binding protein MSI1 | 48655.9 | 431 |
| A0A1D6ED91 | Mitochondrial ribosomal protein S4 | 50270.4 | 432 |
| A0A1D6EN38 | Calreticulin-2 | 48750.5 | 432 |
| A0A1D6J105 | Calcium/calmodulin-regulated receptor-like kinase 1 | 46959.6 | 432 |
| A0A1D6PK39 | FAD/NAD(P)-binding oxidoreductase family protein | 45770 | 432 |
| C0P539 | Polyadenylate-binding protein RBP45C | 47018.5 | 432 |
| C0PFW3 | Malate dehydrogenase6 | 46786.2 | 432 |
| P15719 | Malate dehydrogenase [NADP], chloroplastic | 46859.1 | 432 |
| A0A1D6EP93 | Putative methyltransferase family protein | 47189.7 | 433 |
| A0A1D6GH81 | ATP binding protein | 48366.4 | 433 |
| A0A1D6JNN0 | Protein SHOOT GRAVITROPISM 6 | 47918.6 | 433 |
| B6T3N8 | Glutaryl-CoA dehydrogenase | 46680.6 | 433 |
| B6T7G0 | Glycylpeptide N-tetradecanoyltransferase | 49199.7 | 433 |
| B6TJM7 | Translocon Tic40 | 47428.5 | 433 |
| B6TXA0 | Anthranilate N-benzoyltransferase protein 1 | 46764.1 | 433 |
| C0P4M0 | Monodehydroascorbate reductase 1 peroxisomal | 46470.4 | 433 |
| C0P5P9 | Glycylpeptide N-tetradecanoyltransferase | 49221.7 | 433 |
| K7VIW5 | Plant UBX domain-containing protein 4 | 46186.2 | 433 |
| A0A1D6FRK7 | Sucrose-phosphatase 1 | 48450.8 | 434 |
| A0A1D6MC07 | 2-oxoglutarate (2OG) and Fe(II)-dependent oxygenase superfamily protein | 49014.2 | 434 |
| A0A1D6Q1A1 | Myrosinase | 48685.4 | 434 |
| B4FMY2 | Flavin-containing monooxygenase | 48187.1 | 434 |
| B4FQN7 | Interferon-related developmental regulator family protein / IFRD protein family | 47548.6 | 434 |
| A0A1D6DX29 | Formation of crista junctions protein 1 | 47850.1 | 435 |
| A0A1D6EPM5 | Plant UBX domain-containing protein 2 | 46683.5 | 435 |
| A0A1D6EPN0 | Ubiquitin receptor RAD23c | 47530.4 | 435 |
| A0A1D6QUI4 | AP-3 complex subunit mu | 48208.6 | 435 |
| A0A317YJU4 | Uncharacterized protein | 48850 | 435 |
| B6STS7 | Actin-like protein 3 | 48811.6 | 435 |
| B6TLN7 | Vacuolar sorting protein 4b | 48247.4 | 435 |
| B8A2I4 | Vacuolar sorting protein 4b | 48249.3 | 435 |
| C0P294 | Uncharacterized protein | 49748.1 | 435 |
| C4J106 | Uncharacterized protein | 47889.9 | 435 |
| C4J4E4 | Monodehydroascorbate reductase homolog1 | 46732 | 435 |
| A0A1D6KFY9 | Sulfurtransferase | 46780.1 | 436 |
| A0A1D6PHT4 | Tetratricopeptide repeat (TPR)-like superfamily protein | 49203.2 | 436 |
| C0P7L2 | Eukaryotic translation initiation factor 3 subunit E | 51030 | 436 |
| C0P8K3 | Uncharacterized protein | 48166.1 | 436 |
| A0A1D6EZQ9 | C2 domain containing protein | 45982.4 | 437 |
| A0A1D6M0K6 | Uncharacterized protein | 49516.1 | 437 |
| B4F832 | Adenine nucleotide transporter BT1 chloroplastic/mitochondrial | 47024.1 | 437 |
| B4FQK5 | Eukaryotic peptide chain release factor subunit 1-1 | 49188.3 | 437 |
| B8A183 | Uncharacterized protein | 49050 | 437 |
| C0P4E8 | Nodulin-related protein 1 | 46613.5 | 437 |
| K7UYS1 | Diacylglycerol O-acyltransferase 1 | 48595.1 | 437 |
| K7VUU0 | Protein DJ-1 homolog B | 46772 | 437 |
| A0A1D6KZB8 | Transcription regulator NOT2/NOT3/NOT5 family protein | 48249 | 438 |
| A0A1D6M3X4 | Uncharacterized protein | 44884.2 | 438 |
| A0A1D6N051 | Uncharacterized protein | 48792.6 | 438 |
| A0A317Y703 | Pentatricopeptide repeat-containing protein | 47067.6 | 438 |
| B6ST15 | Eukaryotic peptide chain release factor subunit 1-1 | 48884.8 | 438 |
| B7ZZR4 | Uncharacterized protein | 49305.2 | 438 |
| C4J9M6 | Pentatricopeptide repeat-containing protein mitochondrial | 47220.7 | 438 |
| K7U8T8 | TBC1 domain family member 22A | 50274.4 | 438 |
| K7USR8 | COP9 signalosome complex subunit 2 | 51374.3 | 438 |
| A0A1D6ERC4 | Protein disulfide-isomerase like 2-2 | 47096.2 | 439 |
| A0A1D6FL04 | Uncharacterized protein | 48775.3 | 439 |
| B4FU03 | Lecithin-cholesterol acyltransferase-like 1 | 49000.7 | 439 |
| B6T4T8 | Protein CLP1 homolog | 47709.5 | 439 |
| B6TRH2 | Sad1/unc-84-like protein 2 | 47531.5 | 439 |
| B6TXK0 | Protein CLP1 homolog | 47624.4 | 439 |
| B6U8E6 | Galactokinase | 47697.8 | 439 |
| B8A1B9 | Uncharacterized protein | 47954.6 | 439 |
| C0P4N7 | Uncharacterized protein | 48854.4 | 439 |
| C0PF66 | Peptidase M20/M25/M40 family protein | 48331.6 | 439 |
| Q5EUD5 | Protein disulfide isomerase8 | 46896 | 439 |
| A0A1D6EHL3 | Calcium dependent protein kinase11 | 49082.3 | 440 |
| A0A1D6IKI4 | VH1-interacting kinase | 49104.5 | 440 |
| A0A1D6NTK0 | Spermidine hydroxycinnamoyl transferase | 46385.6 | 440 |
| A0A317YDE7 | Tryptamine hydroxycinnamoyltransferase 2 | 47899.9 | 440 |
| B4F891 | Tyrosine aminotransferase | 47868.7 | 440 |
| B4FLA2 | Chorismate synthase | 47069.1 | 440 |
| B4FMP7 | Ypt/Rab-GAP domain of gyp1p superfamily protein | 49833.3 | 440 |
| B4FZ10 | Dolichyl-diphosphooligosaccharide--protein glycosyltransferase 48 kDa subunit | 48912 | 440 |
| B4FZ60 | Flavin-containing monooxygenase | 49463.3 | 440 |
| B6SJH0 | 3-dehydroquinate synthase | 47545.1 | 440 |
| B6T554 | Peptidase family M48 containing protein | 48516 | 440 |
| B6TI36 | Transporter, folate-biopterin transporter | 47550.9 | 440 |
| C4J489 | Leucine-rich repeat (LRR) family protein | 45595.9 | 440 |
| K7U7M2 | Inositol-pentakisphosphate 2-kinase | 48951.6 | 440 |
| K7VC90 | TRICHOME BIREFRINGENCE-LIKE 20 | 49201.8 | 440 |
| A0A1D6ERC1 | Protein disulfide-isomerase like 2-2 | 47392.6 | 441 |
| A0A1D6I411 | Plant UBX domain-containing protein 2 | 47087.9 | 441 |
| A0A1D6I6V0 | ATP phosphoribosyltransferase 2 chloroplastic | 48656.5 | 441 |
| A0A1D6LUH7 | 3-oxoacyl-[acyl-carrier-protein] synthase | 46842.1 | 441 |
| A0A1D6M0D9 | Lactoylglutathione lyase | 49106.4 | 441 |
| B6T6U2 | Nucleic acid binding protein | 47022.7 | 441 |
| B6TTU5 | Alliin lyase | 47498.4 | 441 |
| C0PL38 | Uncharacterized protein | 48718 | 441 |
| A0A1D6EAJ9 | Protein disulfide isomerase10 | 49658.4 | 442 |
| A0A1D6FL49 | Putative DUF1296 domain containing family protein | 46739.8 | 442 |
| A0A1D6JL07 | AP-2 complex subunit alpha-2 | 48281.7 | 442 |
| A0A1D6NMY7 | DNA replication licensing factor MCM4 | 49187.7 | 442 |
| A0A1D6QCD9 | Nudix hydrolase 3 | 49746 | 442 |
| A0A1D6QJ05 | RBR-type E3 ubiquitin transferase | 49993 | 442 |
| A0A317Y6H5 | DnaJ | 49024.1 | 442 |
| A0A317YJF6 | Uncharacterized protein | 46394.7 | 442 |
| B4FT77 | Glucan endo-1,3-beta-glucosidase 11 | 46129.9 | 442 |
| B4G0F2 | IAA-amino acid hydrolase ILR1 | 47701.3 | 442 |
| B6TA85 | Protein binding protein | 47570.2 | 442 |
| B6TLH4 | N-acetylglucosaminyltransferase I | 51248 | 442 |
| B6U9G1 | IAA-amino acid hydrolase ILR1-like 4 | 46097 | 442 |
| B6UCS8 | Anthranilate N-benzoyltransferase protein 2 | 48414.7 | 442 |
| B8A019 | Peptidase family M48 family protein | 48702.1 | 442 |
| C0P3P6 | Exocyst subunit Exo70 family protein | 49945.4 | 442 |
| C0PC30 | Spermidine hydroxycinnamoyl transferase | 47242.5 | 442 |
| C4JBW7 | BAHD acyltransferase DCR | 47438.3 | 442 |
| K7UAW0 | Putative regulator of chromosome condensation (RCC1) family protein | 46469.1 | 442 |
| K7V1Z4 | WD repeat-containing protein domain phosphoinositide-interacting protein 3 | 47939.5 | 442 |
| A0A1D6DW08 | D-3-phosphoglycerate dehydrogenase 3 chloroplastic | 47055.1 | 443 |
| A0A1D6FQX3 | Proline-rich receptor-like protein kinase PERK1 | 48398.1 | 443 |
| A0A1D6HPZ2 | 26S proteasome non-ATPase regulatory subunit 12 homolog B | 50525.2 | 443 |
| A0A1D6KGR4 | Haloacid dehalogenase-like hydrolase (HAD) superfamily protein | 46810.1 | 443 |
| A0A1D6L2V2 | Tubulin alpha chain | 48957.9 | 443 |
| A0A1D6N2N0 | ENTH/VHS family protein | 48142.1 | 443 |
| A0A1D6N918 | Vacuolar protein sorting-associated protein 36 | 48536.1 | 443 |
| B4FTT2 | Regulator of chromosome condensation2 | 47158.2 | 443 |
| B6U6Y1 | RNA binding protein | 49597 | 443 |
| C0P4D6 | 26S proteasome non-ATPase regulatory subunit 12 homolog B | 50543.3 | 443 |
| C0PG96 | IAA-amino acid hydrolase ILR1 | 46837.8 | 443 |
| K0D9N0 | C3H11 C3H type transcription factor (Fragment) | 47830.5 | 443 |
| A0A096R0K1 | Polypyrimidine tract-binding protein homolog 3 | 49805 | 444 |
| A0A1D6EQY2 | Putative ethanolamine kinase | 49952 | 444 |
| A0A1D6HWD3 | Protein RETICULATA chloroplastic | 46936.2 | 444 |
| B4F922 | Acyltransferase | 47580.5 | 444 |
| B6TCG6 | Uncharacterized protein | 47573.1 | 444 |
| B6TYM4 | C2 domain containing protein | 46436 | 444 |
| B6TZ19 | Catalytic/ protein phosphatase type 2C | 47243.8 | 444 |
| C4IZS6 | Amidase 1 | 47267.5 | 444 |
| I7HIS1 | 4-hydroxyphenylpyruvate dioxygenase | 47002.2 | 444 |
| P18026 | Tubulin beta-2 chain | 49882.9 | 444 |
| Q5TIM9 | Alpha-6-galactosyltransferase | 48938.7 | 444 |
| A0A1D6FZH5 | Pectin lyase-like superfamily protein | 47311 | 445 |
| A0A1D6GUE1 | Cytoplasmic 60S subunit biogenesis factor REI1 homolog 1 | 50514.6 | 445 |
| A0A317Y4H2 | COP9 signalosome complex subunit 1 | 49918.6 | 445 |
| B4FF32 | Uncharacterized protein | 47041.9 | 445 |
| B4G082 | Guanosine nucleotide diphosphate dissociation inhibitor | 49811.5 | 445 |
| B6SRS7 | Copine-1 | 49546.7 | 445 |
| C0P9U4 | Transducin/WD40 repeat-like superfamily protein | 47444.7 | 445 |
| C0PG55 | Aladin | 48366.4 | 445 |
| C0PGX5 | Nuclear pore complex protein NUP54 | 47264.7 | 445 |
| C4J6K9 | Guanosine nucleotide diphosphate dissociation inhibitor | 49921.4 | 445 |
| Q41784 | Tubulin beta-7 chain | 50094.1 | 445 |
| Q43697 | Tubulin beta-5 chain | 50043.1 | 445 |
| A0A096SSY4 | Tubulin beta chain | 50011 | 446 |
| A0A1D6FBC9 | Hydroxymethylglutaryl-CoA lyase mitochondrial | 46286.6 | 446 |
| A0A1D6GR68 | NagB/RpiA/CoA transferase-like superfamily protein | 48455 | 446 |
| A0A1D6KJ07 | Aminoacylase-1 | 49019.1 | 446 |
| A0A1D6MDT4 | GBF-interacting protein 1 | 47390.6 | 446 |
| B4FWD4 | Uncharacterized protein | 47232.8 | 446 |
| B6T768 | Vacuolar protein sorting protein 36 | 48537 | 446 |
| B6TDS0 | Polygalacturonase | 46975.9 | 446 |
| B6TFG5 | Dihydrolipoyllysine-residue succinyltransferase component of 2-oxoglutarate dehydrogenase complex | 48677.2 | 446 |
| B6TLR4 | Carbamoyl-phosphate synthase small chain | 48626.5 | 446 |
| B7ZXD9 | Uncharacterized protein | 47780.8 | 446 |
| B7ZZ40 | Tubulin beta chain | 50203.2 | 446 |
| B8A0A6 | 26S proteasome regulatory subunit 4 homolog A | 49447.5 | 446 |
| B8A0W7 | Uncharacterized protein | 48135.2 | 446 |
| B8A188 | Uncharacterized protein | 49572.9 | 446 |
| C0HGV5 | Enolase2 | 48114.3 | 446 |
| P26301 | Enolase 1 | 48063.2 | 446 |
| A0A1D6ELV4 | Single-stranded nucleic acid binding R3H protein | 49488.4 | 447 |
| A0A1D6EY66 | Elongation factor 1-alpha | 49267.6 | 447 |
| A0A1D6F4V9 | 26S proteasome regulatory subunit 4 homolog A | 49480.4 | 447 |
| A0A1D6IP54 | 26S proteasome regulatory subunit 4 homolog A | 49434.4 | 447 |
| A0A1D6KA29 | Glycerol-3-phosphate acyltransferase, chloroplastic | 49974.6 | 447 |
| A0A1D6KWP0 | DEK domain-containing chromatin associated protein | 49690 | 447 |
| A0A1D6LPV5 | Peptidyl-prolyl cis-trans isomerase CYP59 | 51396.1 | 447 |
| A0A1D6ND62 | Phenylalanine--tRNA ligase chloroplastic/mitochondrial | 50837.4 | 447 |
| B4F946 | Tubulin beta chain | 50116.1 | 447 |
| B6ST92 | Agmatine coumaroyltransferase | 48025.1 | 447 |
| B6SXY1 | Anthranilate N-benzoyltransferase protein 1 | 48708.1 | 447 |
| B6T4W9 | Uncharacterized protein | 50113.3 | 447 |
| B6TIJ2 | Acetylornithine deacetylase | 49178.7 | 447 |
| B6TK31 | ATP binding protein | 50895.4 | 447 |
| B6TR34 | Methionine aminopeptidase 2 | 49830.2 | 447 |
| B6TV58 | Heterogeneous nuclear ribonucleoprotein A3-like protein 2 | 44566.8 | 447 |
| B6UHJ4 | Elongation factor 1-alpha | 49304.7 | 447 |
| C0HFM5 | IAA-amino acid hydrolase ILR1-like 4 | 46477.5 | 447 |
| K7TEL4 | Purple acid phosphatase | 48607.1 | 447 |
| K7UUB7 | Elongation factor 1-alpha | 49223.7 | 447 |
| O50018 | Elongation factor 1-alpha | 49289.7 | 447 |
| Q41782 | Tubulin beta-4 chain | 50132.1 | 447 |
| Q9M7E1 | Elongation factor 1-alpha | 49147.6 | 447 |
| A0A1D6Q270 | Ubiquitin-associated (UBA)/TS-N domain-containing protein | 50651 | 448 |
| B4F9U9 | Hydroxyproline-rich glycoprotein family protein | 49361 | 448 |
| B4G0V3 | Adenosylhomocysteinase | 48775.7 | 448 |
| B4G179 | SNF4 | 49871.2 | 448 |
| B6T4N2 | Uncharacterized protein | 50557.1 | 448 |
| B8A3G9 | Uncharacterized protein | 47967.6 | 448 |
| C0P558 | UBP1-associated protein 2C | 44752 | 448 |
| C0P664 | Tubulin beta chain | 50293.2 | 448 |
| K7UT28 | Zinc finger CCCH domain-containing protein 58 | 48616.5 | 448 |
| K7VBB3 | Putative lung seven transmembrane receptor family protein | 50285.9 | 448 |
| K7VKH1 | Tubulin alpha chain | 49852.1 | 448 |
| K7W0A9 | Glycosyltransferase 5 | 51654.8 | 448 |
| Q2WBH7 | Sialyltransferase-like protein 2 | 49501.5 | 448 |
| Q8VZZ8 | Ribosomal protein L2 family | 48153.5 | 448 |
| A0A1D6F8R0 | RNA-binding (RRM/RBD/RNP motifs) family protein | 47878.7 | 449 |
| A0A1D6QRD4 | Fumarylacetoacetase | 49019.7 | 449 |
| A1DZD5 | COBRA-like protein | 49840.9 | 449 |
| B6SST3 | COBRA-like protein | 49792.8 | 449 |
| B6T6H4 | Oligouridylate-binding protein 1C | 48813 | 449 |
| B7ZYS9 | La-related protein 1C | 47172 | 449 |
| C0P7R9 | Non-specific serine/threonine protein kinase | 50648.7 | 449 |
| C4JAR3 | Uncharacterized protein | 50727.6 | 449 |
| A0A1D6HN90 | Diphosphomevalonate decarboxylase | 49189.6 | 450 |
| A0A1D6L3J0 | Diphosphomevalonate decarboxylase | 49325.8 | 450 |
| A0A1D6PF20 | 26S proteasome non-ATPase regulatory subunit 4 | 48147.9 | 450 |
| A0A317YC42 | Cupincin | 49870.5 | 450 |
| B4F861 | IAA-amino acid hydrolase ILR1-like 4 | 48291.6 | 450 |
| B4FH57 | Elongation factor Tu | 48403.3 | 450 |
| B4FWK5 | 7-dehydrocholesterol reductase | 51200.6 | 450 |
| B6TDX6 | Aspartic proteinase nepenthesin-1 | 46662.9 | 450 |
| B8A1S6 | Protein TIC 40 chloroplastic | 48983.9 | 450 |
| C4JBJ2 | Tubulin alpha chain | 49560.5 | 450 |
| K7VXL1 | Pentatricopeptide repeat-containing protein mitochondrial | 50524.4 | 450 |
| Q546I4 | Tubulin alpha chain | 49624.6 | 450 |
| Q7M1Z8 | Globulin-2 | 49922.6 | 450 |
| A0A1D6E566 | AP-4 complex subunit mu | 50537.4 | 451 |
| A0A1D6FMJ0 | Elongation factor 1-alpha | 49641.1 | 451 |
| A0A1D6FPH7 | Uncharacterized protein | 51391.5 | 451 |
| A0A1D6M1G2 | Elongation factor 1-alpha | 49644.2 | 451 |
| A0A1D6ML15 | Acyl-coenzyme A oxidase 4 peroxisomal | 48722.9 | 451 |
| A0A1D6PA29 | Beta-adaptin-like protein C | 48979 | 451 |
| B4FX20 | AKIN gamma | 48416.3 | 451 |
| B6SPX4 | Tubulin alpha chain | 49716.6 | 451 |
| B6T3M2 | Cysteine desulfurase | 49658.5 | 451 |
| B6T4S0 | Cysteine desulfurase | 49636.4 | 451 |
| B6T7C2 | Eukaryotic translation initiation factor 5 | 48915.9 | 451 |
| B6T978 | Uncharacterized protein | 48718.1 | 451 |
| B6TEH8 | Glycosyltransferase | 49347.2 | 451 |
| B6UGJ0 | Globulin-1 S allele | 49963.6 | 451 |
| K7V5N4 | Eukaryotic aspartyl protease family protein | 48758.1 | 451 |
| Q41789 | Calcium-dependent protein kinase (Fragment) | 50596.2 | 451 |
| A0A1D6MXA8 | DNA replication licensing factor MCM7 | 50385 | 452 |
| A0A1D6QRE3 | Uncharacterized protein | 49348.8 | 452 |
| A0A317Y540 | 2-haloacrylate reductase | 47726.6 | 452 |
| A0A317Y5B1 | Acyl transferase 1 | 49126.2 | 452 |
| B4FVB2 | Elongation factor Tu | 48483.4 | 452 |
| C0P6H1 | BSD domain-containing protein | 49640.9 | 452 |
| C0P6Z0 | Uncharacterized protein | 48234.4 | 452 |
| C0PHH2 | SNF4 | 50305.6 | 452 |
| A0A1D6J6D5 | Sucrose nonfermenting 4-like protein | 50932.9 | 453 |
| A0A1D6J6Y1 | Monooxygenase 1 | 48085.4 | 453 |
| A0A1D6MDV5 | Carboxypeptidase | 48142.3 | 453 |
| A0A1D6MUC5 | Putative Ufm1-specific protease | 49949.8 | 453 |
| A0A1D6QTX9 | RuvB-like helicase | 49622.5 | 453 |
| B4FUJ6 | Myosin heavy chain-related | 50837.3 | 453 |
| B4FVM1 | Uncharacterized protein | 48987.9 | 453 |
| B6TAM4 | Zinc finger C-x8-C-x5-C-x3-H type family protein | 52252.4 | 453 |
| B8A305 | Heterogeneous nuclear ribonucleoprotein 1 | 44969.7 | 453 |
| K7VPB0 | Pentatricopeptide repeat-containing protein mitochondrial | 50018.3 | 453 |
| Q9LKY2 | WD-repeat protein RBAP1 | 49834.3 | 453 |
| A0A096TSS0 | Phenolic glucoside malonyltransferase 1 | 48250.5 | 454 |
| A0A097PLK9 | Transducin/WD40 repeat-like superfamily protein (Fragment) | 50584.9 | 454 |
| A0A1D6EZH3 | Glucan endo-13-beta-glucosidase 4 | 49325.4 | 454 |
| A0A1D6FQ43 | Guanosine nucleotide diphosphate dissociation inhibitor | 50861.8 | 454 |
| A0A1D6FV54 | Isovaleryl-CoA dehydrogenase mitochondrial | 49721.1 | 454 |
| A0A1D6FW13 | Actin-7 | 50233.2 | 454 |
| A0A1D6GUA2 | Putative 2-oxoglutarate-dependent dioxygenase DIN11 | 50656.7 | 454 |
| A0A1D6IIU2 | Saccharopine dehydrogenase | 48557.2 | 454 |
| A0A1D6JYA9 | DNA binding protein | 48829 | 454 |
| A0A1D6KCC9 | Alpha/beta-Hydrolases superfamily protein | 50503.6 | 454 |
| B4FT02 | Uncharacterized protein | 52513 | 454 |
| B4FYX5 | Pentatricopeptide (PPR) repeat protein-like | 49797.3 | 454 |
| B6T3D2 | Saccharopine dehydrogenase | 48622.2 | 454 |
| B6TU21 | Anthocyanin 5-aromatic acyltransferase | 47985.1 | 454 |
| B6U9U3 | Dihydrolipoamide acetyltransferase component of pyruvate dehydrogenase complex | 47489.8 | 454 |
| K7W7R1 | Homogentisate 12-dioxygenase | 50224.4 | 454 |
| A0A1D6F4Z3 | ARM repeat superfamily protein | 52548.3 | 455 |
| A0A1D6G762 | Sister chromatid cohesion 1 protein 4 | 50333.1 | 455 |
| A0A1D6L7E9 | Hydroxyproline-rich glycoprotein family protein | 49142.4 | 455 |
| A0A1D6LZ40 | E3 ubiquitin-protein ligase UPL6 | 52087.2 | 455 |
| A0A1D6N631 | Glutamate-oxaloacetate transaminase4 | 49327.3 | 455 |
| B4F9I2 | RuvB-like helicase | 49800.8 | 455 |
| B4FRM0 | RuvB-like helicase | 49851 | 455 |
| B6TJJ4 | RNA binding protein | 46751.4 | 455 |
| B6UEC8 | GTP binding protein | 49857.5 | 455 |
| C0PMD9 | Uncharacterized protein | 49321.2 | 455 |
| A0A1D6EWS4 | Adenosine 5'-phosphosulfate reductase-like2 | 49525.8 | 456 |
| A0A1D6F3N4 | Glycosyltransferase | 48365.5 | 456 |
| A0A1D6FCW8 | Putative acetyl-CoA acetyltransferase cytosolic 2 | 46796.9 | 456 |
| A0A1D6HAH8 | Phosphoinositide phosphatase SAC6 | 52277.2 | 456 |
| A0A1D6I8V4 | Basic-leucine zipper (BZIP) transcription factor family protein | 49081.3 | 456 |
| A0A1D6MJP6 | HXXXD-type acyl-transferase family protein | 48425.7 | 456 |
| A0A1D6MKY7 | Transcription initiation factor TFIID subunit 15b | 43573.1 | 456 |
| A0A1D6QUY1 | Putative jumonji-like transcription factor family protein | 50925.1 | 456 |
| A0A317YD98 | Trafficking protein particle complex subunit 13 | 50355.7 | 456 |
| B4FIG6 | Transferase | 49075.7 | 456 |
| B4FPR2 | Uncharacterized protein | 49831 | 456 |
| B4FTF3 | BURP domain protein RD22 | 45761.6 | 456 |
| B6TBS1 | Ankyrin-1 | 48493.8 | 456 |
| K7UB05 | Spermidine hydroxycinnamoyl transferase | 48382.6 | 456 |
| K7UBP6 | Putative xyloglucan 6-xylosyltransferase 5 | 51179.9 | 456 |
| K7UCA4 | Myosin heavy chain-related | 50896.2 | 456 |
| A0A1D6DU36 | Uncharacterized protein | 51024 | 457 |
| A0A1D6Q8T9 | S-adenosyl-L-methionine-dependent methyltransferase superfamily protein | 50360.4 | 457 |
| A0A1D6QCY8 | 26S protease regulatory subunit 7 homolog A | 51277.9 | 457 |
| A0A317Y489 | mRNA cap guanine-N7 methyltransferase 1 | 51731.1 | 457 |
| B4FD17 | Dihydrolipoamide acetyltransferase component of pyruvate dehydrogenase complex | 47878.5 | 457 |
| B4G1C9 | Dihydrolipoamide acetyltransferase component of pyruvate dehydrogenase complex | 47659.2 | 457 |
| B6SSZ5 | Actin-like protein 3 | 51195.7 | 457 |
| B6T4W6 | Aminoacylase-1 | 50304.6 | 457 |
| B6T994 | Polygalacturonase | 50295.7 | 457 |
| B6TZE8 | Pinin/SDK/memA/ protein conserved region containing protein | 52338.6 | 457 |
| B6UEY2 | Vacuolar processing enzyme, beta-isozyme | 50901.2 | 457 |
| C0PDN9 | p-loop containing nucleoside triphosphate hydrolase superfamily protein | 51566.1 | 457 |
| A0A1D6ESJ2 | Myosin heavy chain-related | 51845.5 | 458 |
| A0A1D6HI72 | Ribose-phosphate pyrophosphokinase 4 | 50469.9 | 458 |
| A0A1D6HIZ6 | Retrotransposon protein Ty1-copia subclass | 48992.7 | 458 |
| B4FRC4 | Serine--tRNA ligase cytoplasmic | 51663.3 | 458 |
| B6SMP5 | Alanine aminotransferase 2 | 50770.5 | 458 |
| A0A1D6EZU2 | SUMO-activating enzyme subunit 2 | 51301.2 | 459 |
| A0A1D6L568 | Glycosyltransferase | 49953.1 | 459 |
| A0A1D6M1Z5 | UDP-glucuronic acid decarboxylase 4 | 50418.9 | 459 |
| A0A1D6Q4B5 | Actin-related protein 3 | 51469.3 | 459 |
| A0A317Y9V6 | Iron-sulfur protein NUBPL | 48742.2 | 459 |
| A0A317YCG5 | Pentatricopeptide repeat-containing protein | 51535.3 | 459 |
| B4FI96 | XS domain containing protein expressed | 50752 | 459 |
| B4FUH2 | Aspartate aminotransferase | 50182 | 459 |
| B6T9J4 | Aspartate aminotransferase | 50351.2 | 459 |
| C0HF79 | Phosphotransferase | 49325.7 | 459 |
| C4IZE8 | UBX domain-containing protein | 50548.2 | 459 |
| K7U0Y2 | Anthocyanin 5-aromatic acyltransferase | 47614.8 | 459 |
| K7UP24 | Glycosyltransferase | 49455.9 | 459 |
| A0A096RY80 | ACT domain-containing protein ACR3 | 49892 | 460 |
| A0A1D6FLR1 | Spliceosome RNA helicase BAT1 isoform 1 | 52148.6 | 460 |
| A0A1D6HI50 | Pyrophosphate--fructose 6-phosphate 1-phosphotransferase subunit alpha 2 | 50291.2 | 460 |
| A0A1D6M4G0 | Beta-hexosaminidase 3 | 50834.6 | 460 |
| A0A317YD99 | AAA-ATPase ASD, mitochondrial | 51784.1 | 460 |
| B4F8P3 | Uncharacterized protein | 49166.4 | 460 |
| B4FYA3 | Xylem bark cysteine peptidase 3 | 48739.6 | 460 |
| B6SI86 | TPR domain containing protein | 49019.1 | 460 |
| B6SWJ8 | LEC14B | 51471.7 | 460 |
| B6TAE3 | TPR domain containing protein | 49086.2 | 460 |
| A0A1D6DU45 | Putative cysteine protease RD21B | 49948.6 | 461 |
| A0A317YCJ7 | Mannose-1-phosphate guanyltransferase alpha | 51380.8 | 461 |
| B4G195 | Glutamate-oxaloacetate transaminase5 | 49306.9 | 461 |
| B6T289 | Glucan endo-1,3-beta-glucosidase 7 | 47379.9 | 461 |
| B6TGG7 | 3-oxoacyl-[acyl-carrier-protein] synthase | 48864.4 | 461 |
| B6TIL6 | 3-ketoacyl-CoA thiolase 2, peroxisomal | 47985.5 | 461 |
| B6TK79 | Aspartate aminotransferase | 49367 | 461 |
| C0P820 | 3-ketoacyl-CoA thiolase 2 peroxisomal | 48145.1 | 461 |
| C0PEG4 | Amino acid permease 6 | 49502.5 | 461 |
| C4J5B2 | Oxysterol-binding protein-related protein 3B | 51956.2 | 461 |
| A0A096PXB4 | Vacuolar cation/proton exchanger | 48864.6 | 462 |
| A0A1R3M158 | Benzoxazinone synthesis9 | 50016.7 | 462 |
| A0A317Y255 | Polyadenylate-binding protein-interacting protein 10 | 50356 | 462 |
| B4FZ85 | 3-oxoacyl-[acyl-carrier-protein] synthase | 48290.2 | 462 |
| B6THD2 | Glucan endo-1,3-beta-glucosidase | 48723.7 | 462 |
| B6THU8 | SUN domain protein1 | 50960.3 | 462 |
| C0PBI7 | Uncharacterized protein | 51076.3 | 462 |
| C0PDR3 | 4-hydroxy-3-methylbut-2-enyl diphosphate reductase | 51601.2 | 462 |
| C0PF10 | Uncharacterized protein | 49069.8 | 462 |
| A0A1D6I8Y0 | 3-hydroxy-3-methylglutaryl coenzyme A synthase | 51965.7 | 463 |
| A0A317YGJ9 | Putative polygalacturonase | 49830.2 | 463 |
| B4FCB4 | Carboxypeptidase | 51870.1 | 463 |
| B4FWQ4 | Carboxypeptidase | 51912.1 | 463 |
| B6SV15 | 5-nucleotidase | 53480.9 | 463 |
| A0A1D6F172 | Uncharacterized protein | 51986.2 | 464 |
| A0A1D6F4V7 | 26S proteasome regulatory subunit 4 homolog A | 51488.7 | 464 |
| A0A1D6FB18 | COP9 signalosome complex subunit 2 | 54396.9 | 464 |
| A0A1D6KS17 | Nucleic acid binding protein | 51416.6 | 464 |
| A0A1R3LJZ7 | Arginine biosynthesis bifunctional protein ArgJ, chloroplastic | 47951.2 | 464 |
| A0A317YDG1 | DIMBOA UDP-glucosyltransferase BX9 | 50248 | 464 |
| B4FSL5 | Glycerol-3-phosphate dehydrogenase [NAD(+)] | 51387.6 | 464 |
| B6TNU0 | Eukaryotic translation initiation factor 2 gamma subunit | 50771.9 | 464 |
| C0HFV7 | Apyrase 1 | 50322.5 | 464 |
| K7U2B2 | 2C-type protein phosphatase protein | 48826.3 | 464 |
| A0A1D6II10 | Spotted leaf protein 11 | 47686.4 | 465 |
| A0A1D6M0K4 | Uncharacterized protein | 52899.4 | 465 |
| B4G0Q5 | Polyadenylate-binding protein RBP45C | 51960.3 | 465 |
| B6SSU4 | DNA binding protein | 49714.9 | 465 |
| B6TPG0 | Elongation factor Tu | 50536.6 | 465 |
| B6TYN1 | Transposon protein Mutator sub-class | 49843.6 | 465 |
| C0PAJ1 | Uncharacterized protein | 49877.6 | 465 |
| C4J040 | 3-hydroxy-3-methylglutaryl coenzyme A synthase | 51810.4 | 465 |
| A0A1D6F9F8 | TatD related DNase | 51045.2 | 466 |
| A0A1D6LEM2 | COP9 signalosome complex subunit 3 | 52004 | 466 |
| A0A1D6M6F7 | Putative aminotransferase class III superfamily protein | 50145 | 466 |
| A0A1D6MCX0 | 60S ribosomal protein L18a-2 | 52713.6 | 466 |
| A0A1D6NKT9 | SIT4 phosphatase-associated family protein | 51930.2 | 466 |
| A0A317Y668 | Rhomboid-like protein 15 | 51368.3 | 466 |
| B4FPC4 | Oxysterol-binding protein-related protein 3B | 52228.1 | 466 |
| B6SKL9 | Lipoprotein | 48937.3 | 466 |
| B6TD00 | Fas-associated factor 1-like protein | 50826.6 | 466 |
| C0P699 | Elongation factor Tu | 50776 | 466 |
| K7U3X9 | Putative bZIP transcription factor superfamily protein | 49699 | 466 |
| Q5EUC8 | Adenosine 5'-phosphosulfate reductase 2 | 50403.7 | 466 |
| A0A1D6HCT7 | La protein 1 | 51922.8 | 467 |
| A0A1D6NQR8 | Uncharacterized protein | 53146.8 | 467 |
| A0A1D6P5J7 | Protein kinase superfamily protein | 51646.3 | 467 |
| B4F9N8 | RNA-binding (RRM/RBD/RNP motifs) family protein | 51279.3 | 467 |
| B4FRJ6 | Glycosyltransferase | 51128.7 | 467 |
| B6TX16 | Nematode-resistance protein | 51187.9 | 467 |
| C0P3V1 | G-type lectin S-receptor-like serine/threonine-protein kinase SD2-5 | 48813.7 | 467 |
| A0A1D6GF31 | Uncharacterized protein | 49521.1 | 468 |
| A0A1D6HIY4 | S-adenosyl-L-methionine-dependent methyltransferase superfamily protein | 51524.8 | 468 |
| A0A1D6J0T1 | Chaperone protein dnaJ A6 chloroplastic | 50512.9 | 468 |
| B6SGJ5 | Ectonucleotide pyrophosphatase/phosphodiesterase 1 | 51175.2 | 468 |
| B6SIW6 | V-type proton ATPase subunit H | 53311.3 | 468 |
| B6TJL6 | Amidohydrolase family protein | 51405.6 | 468 |
| B6TNK6 | Adenylyltransferase and sulfurtransferase MOCS3-1 | 50274.8 | 468 |
| C0P2W6 | Nematode resistance protein-like HSPRO2 | 51440.3 | 468 |
| C0P8K5 | Pentatricopeptide repeat (PPR) superfamily protein | 51585.4 | 468 |
| A0A1D6IVE7 | Uncharacterized protein | 51876.4 | 469 |
| A0A317YJZ9 | Plant UBX domain-containing protein 10 | 51236.4 | 469 |
| B4FEM2 | Uncharacterized protein | 50106.2 | 469 |
| B4FMI4 | Plant UBX domain-containing protein 10 | 51176.3 | 469 |
| B6TCC2 | Nuclear pore complex protein NUP58 | 50488.8 | 469 |
| B6TGM9 | Cysteine protease 1 | 50762.1 | 469 |
| B6TRG3 | Fasciclin domain | 50348.1 | 469 |
| B6U892 | Dihydroorotate dehydrogenase (quinone), mitochondrial | 49547.4 | 469 |
| C0HG73 | Tubulin gamma chain | 52840.6 | 469 |
| C0P5R8 | Putative ureidoglycolate hydrolase | 50465 | 469 |
| C0PEW9 | Pleckstrin homology (PH) domain superfamily protein | 50472.6 | 469 |
| C0PN58 | Tubulin gamma chain | 52884.7 | 469 |
| A0A1D6F477 | Glycosyltransferase | 51072.1 | 470 |
| A0A1D6F563 | La-related protein 6B | 51271.8 | 470 |
| A0A1D6HNF9 | Aspartate aminotransferase chloroplastic | 51603.7 | 470 |
| A0A1D6I2J5 | Sulfite exporter TauE/SafE family protein | 50825.9 | 470 |
| A0A1D6N224 | Potassium transporter 3 | 52994.7 | 470 |
| A0A1D6ND81 | Protein farnesyltransferase subunit beta | 51853.3 | 470 |
| B4FS90 | Cysteine protease 1 | 50227.1 | 470 |
| B4FTA9 | Glycosyltransferase | 50923.8 | 470 |
| B4FU01 | Cystathionine beta-lyase chloroplastic | 50063.5 | 470 |
| B6TR02 | Glycosyltransferase | 50851.3 | 470 |
| B6TXC2 | Isopenicillin N epimerase | 51030.4 | 470 |
| C0P855 | Glycosyltransferase | 51332.5 | 470 |
| C0PJB8 | Carboxypeptidase | 52214.5 | 470 |
| A0A1D6KXU0 | NAD(P)-linked oxidoreductase superfamily protein | 53083.6 | 471 |
| A0A317Y7L5 | Glycosyltransferase | 49694.9 | 471 |
| B4FA12 | HXXXD-type acyl-transferase family protein | 50664.2 | 471 |
| B6SXN9 | Transferase | 50551.1 | 471 |
| B6T7J7 | Serine hydroxymethyltransferase | 51647.5 | 471 |
| B6TWL7 | Uncharacterized protein | 50974.8 | 471 |
| C0LNQ9 | UDP-glucosyltransferase | 50710 | 471 |
| C0P972 | Dihydrolipoamide acetyltransferase component of pyruvate dehydrogenase complex | 48946 | 471 |
| K7TVF9 | Carboxypeptidase | 52496 | 471 |
| Q19R34 | Glycosyltransferase | 48746.9 | 471 |
| A0A1D6E684 | Protein root UVB sensitive 2 chloroplastic | 51694 | 472 |
| A0A1D6MNJ0 | Deoxy xylulose reductoisomerase1 | 51256.4 | 472 |
| B4F8U6 | Ornithine aminotransferase | 51411.8 | 472 |
| B4FIC0 | Citrate synthase | 52456.2 | 472 |
| B4FUZ2 | Dihydrolipoamide acetyltransferase component of pyruvate dehydrogenase complex | 49020.1 | 472 |
| B4FW32 | DnaJ subfamily C member 7 | 52577.3 | 472 |
| B4FWH2 | Uncharacterized protein | 51101.5 | 472 |
| B4G000 | Glycosyltransferase | 51290.8 | 472 |
| B6T437 | DnaJ subfamily C member 7 | 52620.4 | 472 |
| B6T784 | UDP-sulfoquinovose synthase | 52510.2 | 472 |
| B7ZWY9 | Citrate synthase | 52430.1 | 472 |
| C4JAX7 | UDP-sulfoquinovose synthase chloroplastic | 52452.1 | 472 |
| C4JBU1 | Glycosyltransferase | 51027.5 | 472 |
| Q9FXZ7 | Putative 1-deoxy-D-xylulose 5-phosphate reductoisomerase | 51284.5 | 472 |
| A0A1D6ECC8 | Dihydrolipoyllysine-residue succinyltransferase component of 2-oxoglutarate dehydrogenase complex | 51741.8 | 473 |
| A0A1D6H8U7 | Leucine aminopeptidase 2 chloroplastic | 49569.8 | 473 |
| A0A1D6M7J3 | Glycosyltransferase | 50156.9 | 473 |
| A0A1D6QBX9 | MAK10-like protein | 54319.6 | 473 |
| A0A1X7YHF6 | Uncharacterized protein | 50191.8 | 473 |
| B4FAD9 | UTP--glucose-1-phosphate uridylyltransferase | 52177.4 | 473 |
| B6T402 | Endoplasmic oxidoreductin-1 | 53268.3 | 473 |
| B6T4R3 | UTP--glucose-1-phosphate uridylyltransferase | 52087.3 | 473 |
| B6TPE4 | Glutamate-1-semialdehyde 2,1-aminomutase | 50025.9 | 473 |
| B6TSL5 | Glycosyltransferase | 51576.1 | 473 |
| B6UAU8 | O-succinylhomoserine sulfhydrylase | 50086.8 | 473 |
| C0HHF3 | Transcription regulator NOT2/NOT3/NOT5 family protein | 51987.1 | 473 |
| C0PDJ5 | Glycosyltransferase | 51317 | 473 |
| A0A1D6GXI4 | Phosphoinositide phospholipase C | 52556.1 | 474 |
| A0A1D6J0T2 | Chaperone protein dnaJ A6 chloroplastic | 51292.8 | 474 |
| A0A1D6JII2 | BR-signaling kinase 3 | 53122.6 | 474 |
| A0A1D6ME26 | Putative casein kinase family protein | 53740.1 | 474 |
| A0A1D6NHS9 | Glycosyltransferase | 52044.8 | 474 |
| A0A1D6PUB2 | Putative aminotransferase class III superfamily protein | 51997 | 474 |
| B6TQW0 | Carboxypeptidase | 52657.9 | 474 |
| B6TWY5 | ATP binding protein | 51459.4 | 474 |
| B7ZYW4 | Glutamate-1-semialdehyde 21-aminomutase 2 chloroplastic | 50164 | 474 |
| C0P3M5 | Uncharacterized protein | 50227.5 | 474 |
| K7TTR0 | Tryptophan synthase | 50698.2 | 474 |
| A0A0P0HX16 | Glucose-1-phosphate adenylyltransferase | 52269.2 | 475 |
| A0A1D6FWB9 | Argonaute1 | 50552.3 | 475 |
| A0A1D6IWV9 | Oxo-glutarate/malate transporter1 | 48815.9 | 475 |
| A0A1D6LUN4 | Choline/ethanolaminephosphotransferase 1 | 52785.1 | 475 |
| A0A1D6Q353 | SPla/RYanodine receptor (SPRY) domain-containing protein | 53402.7 | 475 |
| A0A317YLY6 | ADP,ATP carrier protein, mitochondrial | 51881.4 | 475 |
| B6SRJ5 | Bifunctional 3-phosphoadenosine 5-phosphosulfate synthetase 2 | 52354.6 | 475 |
| C0HI51 | Zn-dependent exopeptidase superfamily protein | 52199 | 475 |
| C0P384 | Digalactosyldiacylglycerol synthase 2 chloroplastic | 54374.9 | 475 |
| K7UTY7 | Putative casein kinase family protein | 53622 | 475 |
| Q7XB40 | Glutathione synthetase | 52537.6 | 475 |
| A0A1D6F581 | Acetolactate synthase small subunit 1 chloroplastic | 51827.1 | 476 |
| A0A1D6HCT8 | La protein 1 | 52837.1 | 476 |
| A0A1D6IF20 | GC-rich sequence DNA-binding factor-like protein | 53145.8 | 476 |
| A0A1D6N6H9 | Anthocyanidin 3-O-glucosyltransferase | 52580.6 | 476 |
| A0A1D6NHR8 | Zn-dependent exopeptidase superfamily protein | 52328.1 | 476 |
| A0A317Y1K9 | Glycosyltransferase | 50852.5 | 476 |
| A0A317Y1V8 | Histidinol dehydrogenase, chloroplastic | 50946.1 | 476 |
| B4G0S0 | Putative ADP-ribosylation factor GTPase-activating protein AGD11 | 51248.9 | 476 |
| B4G191 | Uncharacterized protein | 50524 | 476 |
| B6SWS9 | Purple acid phosphatase | 55064.2 | 476 |
| B6T3B8 | Anthocyanidin 3-O-glucosyltransferase | 51889.3 | 476 |
| B6TGL7 | Enolase | 50260.7 | 476 |
| B7ZZM4 | Uncharacterized protein | 51855.2 | 476 |
| A0A1D6JZR5 | LL-diaminopimelate aminotransferase chloroplastic | 51549.2 | 477 |
| A0A1D6MZX4 | Receptor-like kinase LIP2 | 51342.7 | 477 |
| A0A1D6Q2L8 | Nuclear transport factor 2 (NTF2) family protein with RNA binding (RRM-RBD-RNP motifs) domain | 51000.4 | 477 |
| B4FAW1 | ATP-dependent 6-phosphofructokinase | 51230.7 | 477 |
| B4FG76 | Uncharacterized protein | 53900.9 | 477 |
| B6SMI4 | Uncharacterized protein | 53784.6 | 477 |
| B6U4L5 | Uncharacterized protein | 52322.9 | 477 |
| C4J0A1 | Uncharacterized protein | 51128 | 477 |
| K7V228 | Isovaleryl-CoA dehydrogenase mitochondrial | 51804.7 | 477 |
| A0A1D6JQ63 | Alanine--glyoxylate aminotransferase 2 homolog 1 mitochondrial | 52014.5 | 478 |
| A0A1D6M043 | Succinate--CoA ligase [ADP-forming] subunit beta mitochondrial | 52381.4 | 478 |
| B6STU5 | Proline dehydrogenase | 50645.3 | 478 |
| B6SWD5 | Aldehyde dehydrogenase | 52171.2 | 478 |
| B6T6V5 | Ubiquitin carboxyl-terminal hydrolase 6 | 53612.4 | 478 |
| B6TAN0 | Monodehydroascorbate reductase, cytoplasmic isoform 2 | 51965.1 | 478 |
| B6TCK9 | Glycosyltransferase | 51769.3 | 478 |
| B6TN62 | RuvB-like helicase | 52166.1 | 478 |
| B6TX89 | F-box domain containing protein | 52229.8 | 478 |
| B8A028 | Monodehydroascorbate reductase 4 peroxisomal | 51949.1 | 478 |
| C0P7X2 | Aldehyde dehydrogenase | 52183 | 478 |
| Q9LKI4 | UMP synthase | 51246.9 | 478 |
| A0A096S680 | UDP-glycosyltransferase 71B1 | 50792.3 | 479 |
| A0A1D6HSH6 | Non-specific serine/threonine protein kinase | 53946.7 | 479 |
| A0A1D6J8L6 | Transglutaminase15 | 50824.2 | 479 |
| A0A1D6M7B9 | Putative glucan endo-13-beta-glucosidase BG1 | 49282.4 | 479 |
| A0A1D6Q567 | Succinate--CoA ligase [ADP-forming] subunit beta, mitochondrial | 51552.3 | 479 |
| A0A317YCX7 | LanC-like protein GCL2 | 52452.2 | 479 |
| B4FAN3 | Glycosyltransferase | 51287 | 479 |
| B4FP22 | Glycosyltransferase | 50650.5 | 479 |
| B6TIF7 | Glucan endo-1,3-beta-glucosidase A6 | 51588 | 479 |
| K7TR93 | Tryptophan synthase | 51088.5 | 479 |
| K7UBB6 | Glycosyltransferase 6 | 52287.7 | 479 |
| K7VN61 | Enolase | 51303.9 | 479 |
| A0A1D6IN22 | Glycosyltransferase | 52397.5 | 480 |
| A0A1D6JU82 | F-box protein SKIP22 | 51422.6 | 480 |
| A0A1D6M7C2 | Phosphoglycerate kinase | 49838.9 | 480 |
| A0A1D6N1Z8 | 6-phosphogluconate dehydrogenase, decarboxylating | 52655.7 | 480 |
| A0A1D6QPH2 | Putative heavy metal transport/detoxification superfamily protein | 48690 | 480 |
| A0A317Y7Q6 | F-box protein SKIP22 | 51538.8 | 480 |
| A0A317Y968 | UDP-glucose 6-dehydrogenase | 52911.3 | 480 |
| B4F8X9 | THO complex subunit 5B | 53727.9 | 480 |
| B6SNQ2 | Glycosyltransferase | 52396.5 | 480 |
| B6ST52 | RNA-binding protein-like | 51015.4 | 480 |
| B6STH5 | Phosphoglycerate kinase | 49868.9 | 480 |
| B6TC87 | Uncharacterized protein | 51316.9 | 480 |
| B6U0V5 | Carboxypeptidase | 52580.2 | 480 |
| B7ZYX8 | UDP-glucose 6-dehydrogenase | 52937.4 | 480 |
| B7ZZ56 | Glycosyltransferase | 52067.6 | 480 |
| C0PL33 | 6-phosphogluconate dehydrogenase, decarboxylating | 52674.7 | 480 |
| K7V4T1 | Glycosyltransferase | 51182.7 | 480 |
| A0A1D6GTH0 | Serine carboxypeptidase-like 19 | 53145.7 | 481 |
| B6SXW4 | Aldehyde dehydrogenase | 52442.5 | 481 |
| B6TCC9 | Dolichyl-diphosphooligosaccharide--protein glycosyltransferase subunit 1 | 54064.8 | 481 |
| B6TPK0 | CAK1AT | 52459.7 | 481 |
| B6TZF0 | Glutamate-rich WD repeat-containing protein 1 | 53013.7 | 481 |
| K7TZ51 | Argininosuccinate synthase chloroplastic | 52539.7 | 481 |
| K7TZS8 | Carbamoyl-phosphate synthase small chain | 52341.1 | 481 |
| K7V9P7 | 3-oxoacyl-[acyl-carrier-protein] synthase I chloroplastic | 51240.2 | 481 |
| A0A096PQR7 | Allene oxide synthase 2 | 53052.5 | 482 |
| A0A1D6DZK9 | Adenosylmethionine aminotransferase1 | 53152.7 | 482 |
| A0A1D6E781 | LETM1-like protein | 54836.6 | 482 |
| A0A1D6GPP6 | Heterogeneous nuclear ribonucleoprotein 1 | 48351.6 | 482 |
| A0A1D6GRB4 | Alanine aminotransferase5 | 52854.2 | 482 |
| A0A1D6KCZ2 | Alanine aminotransferase 2 mitochondrial | 52790 | 482 |
| A0A1D6LEU7 | Pre-mRNA-splicing factor SLU7-A | 56138 | 482 |
| B4FIH9 | Xylose isomerase | 53740.7 | 482 |
| B4FSV6 | 6-phosphogluconate dehydrogenase, decarboxylating | 52850.8 | 482 |
| C0P4G2 | Allene oxide synthase1 | 53034.3 | 482 |
| Q6RW10 | Allene oxide synthase | 53062.4 | 482 |
| A0A1D6G4W3 | WD repeat-containing protein domain phosphoinositide-interacting protein 3 | 52458.7 | 483 |
| A0A1D6JSU1 | Beta-glucosidase 40 | 54325 | 483 |
| A0A1D6KU80 | Glutamate-ammonia ligase | 53266.4 | 483 |
| A0A1D6NE33 | Alpha-amylase 3 chloroplastic | 54269.5 | 483 |
| A0A1D6QAU4 | TBC1 domain family member 22A | 55024.7 | 483 |
| A0A317YC74 | Protein NAR1 | 53317.2 | 483 |
| B4FT69 | Vacuolar protein sorting-associated protein 9A | 54213.8 | 483 |
| B6SUP5 | Cytochrome P450 CYP74A19 | 53104.6 | 483 |
| B6T8U9 | Glycosyltransferase | 51956 | 483 |
| B6TKH0 | Serine/threonine-protein phosphatase 5 | 54678.8 | 483 |
| B6TXK4 | Acetolactate synthase/ amino acid binding protein | 52301.6 | 483 |
| C0PGS3 | Acidic leucine-rich nuclear phosphoprotein 32-related protein | 52444.3 | 483 |
| Q5EUD1 | Protein disulfide isomerase12 | 54485.6 | 483 |
| Q946V2 | Legumin 1 | 52980.3 | 483 |
| A0A1D6JSU6 | SecY protein transport family protein | 53248.1 | 484 |
| A0A1D6L8E9 | Acetyltransferase component of pyruvate dehydrogenase complex | 52040.7 | 484 |
| A0A1D6PHU3 | Mannose-1-phosphate guanylyltransferase 1 | 53575.4 | 484 |
| B4FHJ2 | Xylose isomerase | 53986.8 | 484 |
| B6TF15 | Xylose isomerase | 54072.9 | 484 |
| B6TK69 | Peroxin Pex14 | 51894.9 | 484 |
| B6TYF9 | Xylose isomerase | 54033.1 | 484 |
| C0P6L3 | Pentatricopeptide repeat-containing protein PNM1 mitochondrial | 53985.1 | 484 |
| C0PFA1 | Adenylosuccinate synthetase, chloroplastic | 51954.7 | 484 |
| C0PH97 | Metallo-hydrolase/oxidoreductase superfamily protein | 53241.9 | 484 |
| C4J0N7 | Uncharacterized protein | 52361.5 | 484 |
| K7TJC4 | Glycosyltransferase | 53504.4 | 484 |
| K7U5U9 | Glycosyltransferase | 53318.3 | 484 |
| K7VFD2 | DUF1682 family protein | 53419.4 | 484 |
| M1RFF3 | Endoglucanase | 53548.5 | 484 |
| A0A1D6FH31 | DUF4378 domain protein | 52084.9 | 485 |
| A0A1D6G6F0 | Dolichyl-diphosphooligosaccharide--protein glycosyltransferase subunit 2 | 51928.6 | 485 |
| A0A1D6HPI2 | Myosin2 | 55535.7 | 485 |
| A0A1D6M5J2 | DUF1682 family protein | 53243.1 | 485 |
| A0A1D6NLC5 | Aspartyl protease family protein 2 | 49930.8 | 485 |
| A0A317YJE7 | Actin-related protein 4 | 53930.1 | 485 |
| B4FP24 | Long chain base biosynthesis protein 1 | 52456.8 | 485 |
| B6TDJ8 | Peroxin Pex14 | 52099.1 | 485 |
| B7ZZV0 | Uncharacterized protein | 52809.4 | 485 |
| C0PHR4 | Adenosylhomocysteinase | 53233.9 | 485 |
| K7U2S8 | Putative peptidyl-prolyl cis-trans isomerase and WD40 repeat domain family protein | 55074.4 | 485 |
| K7V2F6 | E3 ubiquitin-protein ligase RGLG1 | 52716.3 | 485 |
| A0A1D6HCR9 | Acetolactate synthase small subunit 1 chloroplastic | 52388.9 | 486 |
| A0A1D6KU72 | Glutamate-ammonia ligase | 53728.9 | 486 |
| A0A317YCI4 | DIMBOA UDP-glucosyltransferase BX9 | 52593.1 | 486 |
| B4FG90 | Glycosyltransferase | 52965.4 | 486 |
| B4FJU6 | Uncharacterized protein | 55138.4 | 486 |
| B4G0U5 | Pectin lyase-like superfamily protein | 51744 | 486 |
| B6T9Q7 | Diaminopimelate decarboxylase | 53089.4 | 486 |
| C0P618 | Uncharacterized protein | 52018.8 | 486 |
| A0A1D6HVY9 | Oligosaccharide transferase1 | 52998.3 | 487 |
| A0A1D6I371 | Zinc finger CCCH domain-containing protein 64 | 53583.4 | 487 |
| A0A1D6IBB1 | Uncharacterized protein | 55097.8 | 487 |
| A0A1D6J0T0 | Chaperone protein dnaJ A6 chloroplastic | 52774.6 | 487 |
| A0A1D6NSE2 | Triacylglycerol lipase-like 1 | 53993.4 | 487 |
| A0A1D6PNA3 | U2 snRNP auxiliary factor large subunit | 52019 | 487 |
| B4F8T3 | Senescence/dehydration-associated protein-related | 52424.8 | 487 |
| B4FAU8 | Uncharacterized protein | 53247.6 | 487 |
| B6SZ57 | Lysine-specific histone demethylase 1 | 52967.9 | 487 |
| C0P6J3 | ATP sulfurylase1 | 53614.9 | 487 |
| A0A1D6GIQ5 | Adenylyl cyclase-associated protein | 51934.7 | 488 |
| A0A1D6HB18 | Glycosyltransferase | 52867.3 | 488 |
| A0A1D6KX35 | Ornithine aminotransferase | 53458 | 488 |
| A0A1D6L2Q2 | Adenylyl cyclase-associated protein | 51882.7 | 488 |
| B4F923 | 2-oxoisovalerate dehydrogenase subunit alpha 2 mitochondrial | 54221.4 | 488 |
| B6T3H3 | Diaminopimelate decarboxylase | 53298.6 | 488 |
| B6TKF2 | RanBP1 domain containing protein | 51478.5 | 488 |
| C0P6I1 | Beta-amylase | 55436.4 | 488 |
| Q1KKK5 | NADH dehydrogenase subunit 2 | 53634.8 | 488 |
| A0A1D6E6B3 | Isocitrate dehydrogenase [NADP] | 54563.3 | 489 |
| A0A1D6F7A7 | Centromere/kinetochore protein zw10-like protein | 53587.6 | 489 |
| A0A1D6IW54 | F-box family protein | 53224.6 | 489 |
| A0A1D6L0I4 | Adenylosuccinate synthetase, chloroplastic | 53170.3 | 489 |
| A0A1D6L3D6 | Uncharacterized protein | 53031.5 | 489 |
| A0A1D6L518 | Protein kinase superfamily protein | 52919.9 | 489 |
| A0A317YF18 | Glycosyltransferase | 52993 | 489 |
| B6SGC0 | Uncharacterized protein | 57197.3 | 489 |
| B6TDA2 | Protein binding protein | 50100.3 | 489 |
| C0PB05 | Protein ENHANCED DISEASE RESISTANCE 2 | 55159.2 | 489 |
| C0PFQ7 | ATP sulfurylase 1 chloroplastic | 53780.1 | 489 |
| O82110 | Seryl-tRNA synthetase (Fragment) | 53682.5 | 489 |
| A0A1D6DTZ9 | Uncharacterized protein | 53369.3 | 490 |
| A0A1D6F4V0 | Mitochondrial import inner membrane translocase subunit TIM44-2 | 55470.1 | 490 |
| A0A1D6G3U1 | Transducin family protein / WD-40 repeat family protein | 53262.7 | 490 |
| A0A1D6GFT3 | O-Glycosyl hydrolase family 17 protein | 52710.4 | 490 |
| A0A1D6L4E5 | Exoglucanase1 | 53358.9 | 490 |
| B4FVB5 | AAA-type ATPase family protein | 53951.9 | 490 |
| B6T7J2 | Uridine kinase | 54415.9 | 490 |
| C0P9T6 | Sterol 14-demethylase | 55623.5 | 490 |
| C0PNY5 | tRNA (guanine(37)-N1)-methyltransferase | 55413.6 | 490 |
| K7UNY3 | Glycosyltransferase | 53398.2 | 490 |
| Q84VG9 | Lycopene beta cyclase chloroplastic | 53360.1 | 490 |
| A0A1D6M0I9 | Carboxypeptidase | 54524.5 | 491 |
| A0A1D6QJ03 | RBR-type E3 ubiquitin transferase | 54858.5 | 491 |
| B4FBW3 | Protein kinase protein with tetratricopeptide repeat domain | 54383.7 | 491 |
| B6TJN6 | Pepsin A | 52800.3 | 491 |
| B6U0H4 | SET domain containing protein | 53821.5 | 491 |
| C0P531 | 26S proteasome non-ATPase regulatory subunit 3 homolog A | 55607 | 491 |
| Q6E0J0 | U1 snRNP | 58080 | 491 |
| A0A1D6EKY1 | UDP-glycosyltransferase 76C1 | 53938.4 | 492 |
| A0A1D6ESX2 | Hydroxyproline-rich glycoprotein family protein | 55245 | 492 |
| A0A1D6HI18 | Pentatricopeptide repeat (PPR) superfamily protein | 54628.9 | 492 |
| A0A1D6K864 | Proline dehydrogenase | 52060.9 | 492 |
| A0A1D6KE60 | Uncharacterized protein | 55737.1 | 492 |
| A0A1D6MK95 | DEAD-box ATP-dependent RNA helicase 14 | 53910.7 | 492 |
| A0A1D6PRW9 | mRNA capping enzyme family protein | 58058 | 492 |
| B4FEB1 | Uncharacterized protein | 55875 | 492 |
| B4FSP1 | Uncharacterized protein | 53908.7 | 492 |
| B6TAE2 | 26S proteasome non-ATPase regulatory subunit 3 | 55736.1 | 492 |
| B6TYJ6 | 3-isopropylmalate dehydratase large subunit 2 | 53136.1 | 492 |
| B6UHU1 | Catalase | 56755.9 | 492 |
| B8A2L6 | Uncharacterized protein | 54738 | 492 |
| Q5QPY4 | Glycosyltransferase | 53872.4 | 492 |
| A0A1D6E4M2 | Vacuolar processing enzyme, beta-isozyme | 54484.3 | 493 |
| A0A1D6ESJ0 | Myosin heavy chain-related | 55697.1 | 493 |
| A0A1D6HSY6 | Ankyrin repeat family protein / regulator of chromosome condensation (RCC1) family protein | 53365.5 | 493 |
| A0A1D6HY96 | Serine carboxypeptidase-like 19 | 54783.8 | 493 |
| A0A1D6ICG5 | Glycosyltransferase | 52351.5 | 493 |
| A0A1D6J013 | Putative cellulose synthase-like family protein | 54740.9 | 493 |
| A0A1D6JLB6 | Protein-ribulosamine 3-kinase chloroplastic | 52782.8 | 493 |
| A0A1D6L8E8 | Dihydrolipoamide acetyltransferase component of pyruvate dehydrogenase complex | 53044.9 | 493 |
| A0A317YJ25 | Uncharacterized protein | 55886.2 | 493 |
| A0A317YJT0 | Sulfite oxidase | 54200.5 | 493 |
| B4F972 | Glutamate decarboxylase | 55218.8 | 493 |
| B4FAL8 | Selenium-binding protein 3 | 54179.7 | 493 |
| B4FRH7 | UDP-N-acetylglucosamine diphosphorylase 2 | 54482.7 | 493 |
| B4FZF7 | UDP-N-acetylglucosamine diphosphorylase 2 | 54232.4 | 493 |
| B6SLU3 | Aldehyde dehydrogenase | 52565.9 | 493 |
| B6TW83 | Glucan endo-1,3-beta-glucosidase 6 | 53142.8 | 493 |
| C0P8T4 | Uncharacterized protein | 54239.4 | 493 |
| K7UEK4 | UDP-N-acetylglucosamine diphosphorylase 2 | 54855.1 | 493 |
| K7VE89 | Zinc finger CCCH domain-containing protein 4 | 55154.7 | 493 |
| O22499 | Cysteine proteinase Mir2 | 53019 | 493 |
| A0A1D6KDB4 | Metallopeptidase M24 family protein | 54717.1 | 494 |
| A0A1D6MVE6 | Methyl binding domain105 | 50304.7 | 494 |
| A0A1D6MZK9 | Transducin/WD40 repeat-like superfamily protein | 53990.4 | 494 |
| C0PGF8 | 3-oxoacyl-[acyl-carrier-protein] synthase II chloroplastic | 52955.2 | 494 |
| C4JC13 | Signal recognition particle 54 kDa protein | 54432.2 | 494 |
| K7UGM3 | Catalase | 56577.4 | 494 |
| Q5QL06 | Vacuolar processing enzyme 1 | 54707.3 | 494 |
| A0A1D6IPR4 | La-related protein 1C | 52479.2 | 495 |
| A0A1D6JEN5 | SIT4 phosphatase-associated family protein | 55544.4 | 495 |
| A0A1D6LGE4 | Putative acetylornithine/succinylornithine aminotransferase family protein | 52075.2 | 495 |
| A0A1D6M0N9 | Proline-rich receptor-like protein kinase PERK1 | 53331.1 | 495 |
| B4G0D8 | TPR domain containing protein | 50541.5 | 495 |
| C0P8H5 | Uncharacterized protein | 54386 | 495 |
| C0PEW8 | Alanine aminotransferase 2 mitochondrial | 54789.2 | 495 |
| C0PJK5 | Uncharacterized protein | 54929.9 | 495 |
| K7UIS1 | Peptidylprolyl isomerase | 53887.2 | 495 |
| A0A1D6II60 | SUMO-activating enzyme subunit 2 | 55326.8 | 496 |
| A0A1D6JHM3 | UBX domain-containing protein | 54715.1 | 496 |
| A0A1D6JRW8 | Ubiquitin system component Cue protein | 53183.4 | 496 |
| A0A1D6NK38 | DENN domain and WD repeat-containing protein SCD1 | 54226.4 | 496 |
| B4FTM1 | Tubulin beta chain | 55267.8 | 496 |
| B4FWY6 | Tyrosine--tRNA ligase | 53864.4 | 496 |
| B6SJZ0 | 3-oxoacyl-synthase I | 53096.5 | 496 |
| B6U923 | NADPH:adrenodoxin oxidoreductase, mitochondrial | 54566.2 | 496 |
| B6UFL7 | Periodic tryptophan protein 1 | 53988.7 | 496 |
| C0P4Z9 | Glucan endo-13-beta-glucosidase 13 | 53988.9 | 496 |
| Q9FUY4 | Protein kinase AKINbetagamma-2 | 54798.9 | 496 |
| A0A1D6EMD6 | Protein prenylyltransferase superfamily protein | 55704.6 | 497 |
| A0A1D6FBA3 | NEDD8-activating enzyme E1 catalytic subunit | 54624.3 | 497 |
| A0A1D6GMC8 | Protein DETOXIFICATION | 53425.5 | 497 |
| A0A1D6N1P8 | Phosphotransferase | 53494.6 | 497 |
| A0A1D6NE83 | WD-repeat protein RBAP2 | 55456.8 | 497 |
| A0A1D6PGW7 | Methionine--tRNA ligase cytoplasmic | 54234.6 | 497 |
| B4FFF5 | Aminopeptidase | 57984.2 | 497 |
| B6UED9 | Uncharacterized protein | 54958.4 | 497 |
| C0HGN9 | Uncharacterized protein | 51337.2 | 497 |
| C4J9I2 | Uncharacterized protein | 52529.8 | 497 |
| K7TNM4 | Nuclear transport factor 2 (NTF2) family protein with RNA binding (RRM-RBD-RNP motifs) domain | 52190.6 | 497 |
| K7UD52 | Cofactor-independent phosphoglycerate mutase | 53146.1 | 497 |
| K7W1K3 | Phosphotransferase | 53407.5 | 497 |
| Q9FUY5 | Protein kinase AKINbetagamma-1 | 54938.1 | 497 |
| A0A1D6P248 | Vacuolar proton pump2 | 55368.5 | 498 |
| A0A1R3NS96 | ATP synthase subunit beta | 54040.5 | 498 |
| B4FR32 | Glyceraldehyde-3-phosphate deHaseN1 | 53284.1 | 498 |
| B4FUA0 | ZPR1 zinc-finger domain protein | 54221.7 | 498 |
| B6SS66 | Phenylalanyl-tRNA synthetase alpha chain | 56119.6 | 498 |
| B7ZXF6 | Phenylalanine--tRNA ligase alpha subunit cytoplasmic | 56103.6 | 498 |
| C0PDA6 | Fumarate hydratase 1 mitochondrial | 53583.8 | 498 |
| C0PEG2 | Uncharacterized protein | 55476.5 | 498 |
| A0A1D6P6P4 | Ras-related protein Rab5 | 54785.9 | 499 |
| A0A317YHH0 | Serine carboxypeptidase-like 18 | 54739.3 | 499 |
| B4FQ54 | Monothiol glutaredoxin-S17 | 53871.9 | 499 |
| B4FQK0 | Monodehydroascorbate reductase 5 mitochondrial | 53764.8 | 499 |
| B4FR21 | ATP-dependent RNA helicase dhh1 | 55843.3 | 499 |
| B4FSW8 | Grx_S17-glutaredoxin subgroup II | 54048.1 | 499 |
| B6TT27 | Glutamate decarboxylase | 55972.5 | 499 |
| B8A2W6 | Uncharacterized protein | 53693.4 | 499 |
| K7US39 | Dihydrolipoyl dehydrogenase | 52653.6 | 499 |
| A0A1D6EU55 | La-related protein 1C | 51972.5 | 500 |
| A0A1D6IHG9 | Protein FREE1 | 53605.8 | 500 |
| B4FTB0 | Uncharacterized protein | 53496.1 | 500 |
| B6SLS9 | Protein arginine methyltransferase NDUFAF7 | 54584.3 | 500 |
| B6SVJ3 | BSD domain containing protein | 56420.1 | 500 |
| B6UAG9 | Glycosyltransferase | 53096.4 | 500 |
| C0P7P6 | Pentatricopeptide repeat-containing protein mitochondrial | 56610.3 | 500 |
| K7U5X9 | Uncharacterized protein | 53198.6 | 500 |
| K7URK6 | Glycosyltransferase | 52572.4 | 500 |
| K7VI04 | Glutamate--cysteine ligase chloroplastic | 56940 | 500 |
| A0A1D6L4K3 | Inosine-5'-monophosphate dehydrogenase | 52570.1 | 501 |
| A0A317YEK2 | Inosine-5'-monophosphate dehydrogenase | 52604.1 | 501 |
| A0A317YJZ8 | Uncharacterized protein | 56524 | 501 |
| B4FJT1 | Uncharacterized protein | 54729.1 | 501 |
| B4FQS9 | Trans-cinnamate 4-monooxygenase | 56984.8 | 501 |
| B6SKW5 | Inosine-5'-monophosphate dehydrogenase | 52678.3 | 501 |
| C0P905 | Glucan endo-13-beta-glucosidase 3 | 53685.4 | 501 |
| C0P9U8 | Uncharacterized protein | 55130.8 | 501 |
| K7TE79 | Sterol 14-demethylase | 56659.7 | 501 |
| U5Y1J4 | Sucrose transporter 4 | 53449.7 | 501 |
| A0A1D6FH77 | tRNAse Z4 | 55336.5 | 502 |
| A0A1D6L2Q1 | Adenylyl cyclase-associated protein | 53322.2 | 502 |
| A0A1D6LJP0 | 6-phosphogluconate dehydrogenase, decarboxylating | 54940.2 | 502 |
| A0A1D6N2N7 | DEK domain-containing chromatin associated protein | 54878.5 | 502 |
| A0A1D6QH29 | Putative alpha-L-arabinofuranosidase family protein | 55542.7 | 502 |
| A0A317YK66 | Tetratricopeptide repeat protein 38 | 57035.8 | 502 |
| B4FBE5 | Inositol 134-trisphosphate 5/6-kinase 4 | 55564.5 | 502 |
| B4FDQ6 | Uncharacterized protein | 56401 | 502 |
| B4G004 | Beta-glucosidase 44 | 56415.3 | 502 |
| B6TES9 | Protein lap1 | 55296.1 | 502 |
| B6TJP9 | Cytochrome P450 CYP76H18 | 55586.5 | 502 |
| B7ZZN9 | Serine/threonine protein phosphatase 2A regulatory subunit | 57372.7 | 502 |
| C0P4J8 | Cytochrome P450 family 76 subfamily C polypeptide 7 | 55572.4 | 502 |
| K7TZ17 | Glycosyltransferase | 52796.7 | 502 |
| Q6QHT5 | Hydroperoxide lyase | 55364.9 | 502 |
| A0A1D6EK73 | Non-specific serine/threonine protein kinase | 57625.4 | 503 |
| A0A1D6H6F1 | Citrate synthase | 55251.7 | 503 |
| A0A1D6HG26 | Uncharacterized protein | 54728.8 | 503 |
| A0A1D6JRK3 | Mannose-1-phosphate guanylyltransferase 1 | 55503.8 | 503 |
| A0A1D6MA77 | Non-specific serine/threonine protein kinase | 57356.8 | 503 |
| A0A1D6MSE3 | Dihydrolipoyl dehydrogenase | 52695.6 | 503 |
| A0A1D6N785 | Regulator of chromosome condensation1 | 51936.5 | 503 |
| A0A317YDF7 | Long chain base biosynthesis protein 1a | 54427.2 | 503 |
| B4FBD2 | Non-specific serine/threonine protein kinase | 57621.2 | 503 |
| B4FFR9 | Uncharacterized protein | 56309.3 | 503 |
| B4FJ99 | Uncharacterized protein | 56508.5 | 503 |
| B6TLY3 | ATP-dependent RNA helicase dhh1 | 56263.7 | 503 |
| B6U2X9 | Phosphotransferase | 54373.2 | 503 |
| B6UER0 | Phosphotransferase | 54334.1 | 503 |
| K7TP80 | Zinc finger (C3HC4-type RING finger) family protein | 54779.7 | 503 |
| A0A1D6JCL0 | Argonaute1b | 57228.2 | 504 |
| A0A1D6MUV3 | Protein kinase superfamily protein | 58723.4 | 504 |
| A0A317YL33 | DEAD-box ATP-dependent RNA helicase 38 | 55774.1 | 504 |
| B4FL42 | Splicing factor SF3a60-like protein | 59325.2 | 504 |
| B4FTE2 | NADH dehydrogenase [ubiquinone] flavoprotein 1, mitochondrial | 55296.9 | 504 |
| B4FYI4 | Uncharacterized protein | 55014.3 | 504 |
| B6SLS0 | ATP-dependent RNA helicase DBP5 | 55793.2 | 504 |
| B6SPK3 | Zinc ion binding protein | 57135.8 | 504 |
| B6SVV3 | LIM transcription factor | 57172.9 | 504 |
| B6TRN2 | Nucleotide binding protein | 55456.3 | 504 |
| K7UC48 | 3-isopropylmalate dehydratase large subunit | 54474.6 | 504 |
| A0A1D6N7M5 | UDP-glucuronic acid decarboxylase 1 | 56512.8 | 505 |
| A5GZ73 | Glucose-1-phosphate adenylyltransferase | 55291.6 | 505 |
| B4FA57 | Allantoate deiminase | 53861.1 | 505 |
| B4FVP3 | Ent-cassadiene C2-hydroxylase | 56020.2 | 505 |
| B4FYE0 | Uncharacterized protein | 56302.7 | 505 |
| B6TRM9 | Mitochondrial-processing peptidase alpha subunit | 54026.7 | 505 |
| B7ZX32 | Uncharacterized protein | 54985.5 | 505 |
| C0P9J6 | Aminoaldehyde dehydrogenase 1 | 54912.5 | 505 |
| C4JBG7 | 3-isopropylmalate dehydratase large subunit | 54654.8 | 505 |
| Q53CF4 | Betaine aldehyde dehydrogenase | 54975.6 | 505 |
| A0A1D6FLZ8 | Tubulin gamma chain | 57130.6 | 506 |
| A0A1D6HJY5 | Rho-related protein from plants 1 | 55538.4 | 506 |
| A0A1D6LEP6 | Serine/threonine protein phosphatase 2A regulatory subunit | 57399.6 | 506 |
| A0A1D6MLR2 | Protein ASPARTIC PROTEASE IN GUARD CELL 1 | 51822.3 | 506 |
| A0A1D6NCX2 | Phosphotransferase | 55041.6 | 506 |
| A0A1D6NT88 | Glycosyltransferase | 52414.1 | 506 |
| A0A1D6NVZ6 | 3-phosphoshikimate 1-carboxyvinyltransferase | 53452.2 | 506 |
| A0A317YBC2 | Putative ADP-ribosylation factor GTPase-activating protein AGD8 | 54753.8 | 506 |
| B4FSK2 | AMSH-like ubiquitin thioesterase 3 | 56439.4 | 506 |
| B6SWI7 | Betaine-aldehyde dehydrogenase | 54805.3 | 506 |
| B8A1P7 | Uncharacterized protein | 55264.5 | 506 |
| C0P753 | Sugar transporter ERD6-like 6 | 54084.8 | 506 |
| A0A1D6FAT9 | Putative WRKY transcription factor 47 | 53193.5 | 507 |
| A0A1D6K2X7 | Nuclear cap-binding protein subunit 1 | 58173.9 | 507 |
| A0A1D6MAK9 | Phosphotransferase | 54691.1 | 507 |
| A0A317YGW9 | Uncharacterized transposon-derived protein F52C9.6 | 58428 | 507 |
| B6TSB3 | Glucose-6-phosphate 1-dehydrogenase | 57628.2 | 507 |
| B6UA70 | Uncharacterized protein | 57187.6 | 507 |
| B7ZY06 | Uncharacterized protein | 54897.3 | 507 |
| C0HHF0 | Carboxypeptidase | 55802.6 | 507 |
| C0PD06 | Uncharacterized protein | 56244.7 | 507 |
| G2XK55 | AMSH-like ubiquitin thioesterase 3 | 56663.7 | 507 |
| K7TVZ1 | 6-phosphogluconate dehydrogenase, decarboxylating | 54167 | 507 |
| A0A1D6E225 | Proline--tRNA ligase cytoplasmic | 58097 | 508 |
| A0A1D6GSC8 | U1 snRNP | 60013.3 | 508 |
| A0A1D6M4H0 | Guanosine nucleotide diphosphate dissociation inhibitor 2 | 56796.2 | 508 |
| A0A1D6NI59 | Cytochrome P450 11 | 58332.8 | 508 |
| A0A1D6NM08 | Maternal effect embryo arrest 18 | 55405 | 508 |
| B6SPD1 | Purple acid phosphatase | 57195.6 | 508 |
| B7ZXD1 | Uncharacterized protein | 56109 | 508 |
| C0P3W2 | Flowering locus K homology domain | 54203 | 508 |
| C0PDP9 | DEAD-box ATP-dependent RNA helicase 5 | 55301.3 | 508 |
| K7UDG5 | Proline--tRNA ligase cytoplasmic | 58133.2 | 508 |
| K7V3X5 | Putative calcium-dependent protein kinase family protein | 56478.8 | 508 |
| Q6R987 | ATP synthase subunit alpha | 55179.8 | 508 |
| A0A1D6M6F5 | Putative aminotransferase class III superfamily protein | 55109.7 | 509 |
| A0A1D6PNQ4 | Carboxypeptidase | 53089.2 | 509 |
| A0A317YHB1 | Putative serine protease EDA2 | 56100.5 | 509 |
| B4FWG0 | Glutathione synthetase | 56103.6 | 509 |
| B4G0F5 | Ent-cassadiene C2-hydroxylase | 56661.5 | 509 |
| B6T574 | Pyruvate kinase | 54918.4 | 509 |
| B6TB11 | Aldehyde dehydrogenase family 7 member A1 | 54417.4 | 509 |
| B6THH6 | Prolyl carboxypeptidase like protein | 56056.5 | 509 |
| B6TL64 | Xaa-Pro dipeptidase | 56698.9 | 509 |
| B6UAK5 | Phospho-2-dehydro-3-deoxyheptonate aldolase | 56543 | 509 |
| B6UCV5 | Shikimate biosynthesis protein aroDE | 54827 | 509 |
| K7URA3 | Putative aminotransferase class III superfamily protein | 55753.2 | 509 |
| K7VX77 | Phosphotransferase | 54863.5 | 509 |
| O04981 | Cystathionine gamma-synthase | 54801.8 | 509 |
| Q6RXY1 | Non-specific serine/threonine protein kinase | 58322.6 | 509 |
| A0A1D6GCF3 | Phenylalanine--tRNA ligase chloroplastic/mitochondrial | 58587.5 | 510 |
| A0A1D6HCF4 | Glyceraldehyde-3-phosphate dehydrogenase4 | 55731.1 | 510 |
| A0A1D6IJN4 | 3-oxoacyl-[acyl-carrier-protein] synthase II chloroplastic | 54727.3 | 510 |
| A0A1D6JIK2 | Cell division protein FtsZ homolog 2-2 chloroplastic | 54162.3 | 510 |
| A0A1D6KSD9 | DNA ligase | 57665.4 | 510 |
| A0A1D6QBT7 | AP-1 complex subunit gamma-1 | 56856.3 | 510 |
| A0A317YD32 | U3 snoRNP-associated protein-like YAOH | 56505.2 | 510 |
| B6T9P4 | Inositol-3-phosphate synthase | 56258.9 | 510 |
| B6THW0 | XS domain containing protein expressed | 55035.3 | 510 |
| C0P3K6 | Aspartic proteinase A1 | 54552.9 | 510 |
| C4J4T0 | Serine/threonine protein phosphatase 2A regulatory subunit | 58196.5 | 510 |
| Q941P2 | Glucose-1-phosphate adenylyltransferase | 55936.4 | 510 |
| A0A1D6G8Y5 | Internal alternative NAD(P)H-ubiquinone oxidoreductase A1 mitochondrial | 56427.3 | 511 |
| A0A1D6GGD7 | Allene oxide synthase chloroplastic | 56583.5 | 511 |
| A0A1D6Q8W2 | Rubisco methyltransferase family protein | 57158.5 | 511 |
| B4FMU0 | Phospho-2-dehydro-3-deoxyheptonate aldolase | 56585.1 | 511 |
| B4FQQ6 | Beta-glucosidase 47 | 57615.6 | 511 |
| B7ZWZ2 | Serine/threonine-protein phosphatase 2A 55 kDa regulatory subunit B | 57102.3 | 511 |
| C0PG71 | Uncharacterized protein | 56925.2 | 511 |
| A0A1D6FPF7 | Uncharacterized protein | 57540.6 | 512 |
| A0A1D6HCZ6 | Uncharacterized protein | 55532.7 | 512 |
| A0A1D6I5Q5 | Aspartate aminotransferase | 55660.2 | 512 |
| A0A1D6M0A2 | O-acyltransferase | 57547 | 512 |
| A0A1D6M3E7 | Decapping nuclease DXO homolog chloroplastic | 56385.7 | 512 |
| B4FDS5 | Cytochrome P450 98A3 | 58274.1 | 512 |
| B4FU23 | Uncharacterized protein | 57539.4 | 512 |
| B4G0F6 | Uncharacterized protein | 57410.7 | 512 |
| B6SLF3 | Cytochrome P450 CYP98A29 | 58259.2 | 512 |
| B6SWH5 | ARM repeat superfamily protein | 57443 | 512 |
| B6TBG7 | Seryl-tRNA synthetase | 56175.3 | 512 |
| B7ZXH6 | Beta-glucosidase 44 | 58436.7 | 512 |
| C0PBZ4 | Phosphomevalonate kinase | 54967.1 | 512 |
| Q5EUE0 | Protein disulfide-isomerase | 56682.6 | 512 |
| A0A1D6DSQ4 | CASP-like protein 1 | 57594.8 | 513 |
| A0A1D6ET42 | Thioesterase/thiol ester dehydrase-isomerase superfamily protein | 56536.5 | 513 |
| A0A1D6F9T8 | Ubiquitin carboxyl-terminal hydrolase 13 | 60742.8 | 513 |
| A0A1D6HSU0 | Ubiquitin carboxyl-terminal hydrolase 13 | 60694.7 | 513 |
| A0A1D6MRM2 | ATP-citrate synthase beta chain protein 2 | 55563.5 | 513 |
| B4FJ63 | Uncharacterized protein | 58303.8 | 513 |
| B6T7Q7 | Serine hydroxymethyltransferase | 56534.3 | 513 |
| C4J2M5 | Pyruvate kinase | 55330.2 | 513 |
| P56521 | Probable histone deacetylase 19 | 57545.9 | 513 |
| A0A1D6EKR3 | Glutamine-dependent NAD(+) synthetase | 57177.8 | 514 |
| A0A1D6HBY3 | Calcineurin-like metallo-phosphoesterase superfamily protein | 58151.4 | 514 |
| A0A1D6M360 | Putative transmembrane GTPase FZO-like chloroplastic | 55858.5 | 514 |
| A0A1D6QQE1 | Acetyltransferase component of pyruvate dehydrogenase complex | 55654 | 514 |
| B4F8P5 | Uncharacterized protein | 57191.2 | 514 |
| B7ZZ12 | Diphthamide synthesis DPH2 family protein | 56686.4 | 514 |
| C0P8G0 | CTC-interacting domain 7 | 56357.1 | 514 |
| C0PD86 | Serine/threonine-protein phosphatase 2A 55 kDa regulatory subunit B | 57315.6 | 514 |
| Q5EUE1 | Protein disulfide-isomerase | 56873 | 514 |
| A0A1D6EPK6 | Malonyl-CoA decarboxylase family protein | 56877.7 | 515 |
| A0A1D6FT70 | AGC (cAMP-dependent cGMP-dependent and protein kinase C) kinase family protein | 60006.2 | 515 |
| A0A1D6HC36 | Eukaryotic translation initiation factor 4B | 55570.5 | 515 |
| A0A1D6M065 | Serine/threonine-protein phosphatase 5 | 58920.2 | 515 |
| B6SPX1 | Outer membrane protein, OMP85 family protein | 55318.6 | 515 |
| C0HH11 | Uncharacterized protein | 56842.2 | 515 |
| K7TLK6 | Outer membrane OMP85 family protein | 55046.5 | 515 |
| A0A1D6GZG2 | Pectin lyase-like superfamily protein | 54603 | 516 |
| A0A1D6KNN0 | D-3-phosphoglycerate dehydrogenase | 54410.2 | 516 |
| A0A1D6ND26 | NADPH reductase TAH18 | 57612.2 | 516 |
| A0A1D6NHZ7 | Glucose-1-phosphate adenylyltransferase | 57034.4 | 516 |
| A0A1D6QEI5 | p-loop containing nucleoside triphosphate hydrolase superfamily protein | 57588.9 | 516 |
| A0A317YDU3 | RNA-binding protein 39 | 57253.2 | 516 |
| B4FA61 | Uncharacterized protein | 56540.2 | 516 |
| B4FAV4 | Argininosuccinate lyase chloroplastic | 57542.3 | 516 |
| B4G0V4 | Coatomer subunit delta | 56891.4 | 516 |
| B6U7A9 | Glycosyltransferase | 55440.1 | 516 |
| B8A0Q3 | Carboxypeptidase | 56727.2 | 516 |
| J7H390 | Glucose-1-phosphate adenylyltransferase | 57099.5 | 516 |
| P55241 | Glucose-1-phosphate adenylyltransferase large subunit 1, chloroplastic/amyloplastic | 57070.5 | 516 |
| A0A1D6K7T1 | Putative serine/threonine-protein kinase | 57699.3 | 517 |
| A0A1D6KES1 | Nucleobase-ascorbate transporter 3 | 55628.1 | 517 |
| A0A1Q0YC85 | Probable bifunctional methylthioribulose-1-phosphate dehydratase/enolase-phosphatase E1 | 56969.7 | 517 |
| B4FMT5 | Cytochrome P450 709B2 | 57781.1 | 517 |
| B6T965 | ATP-dependent RNA helicase dhh1 | 59005 | 517 |
| B6TD89 | WD-40 repeat protein MSI4 | 56584.7 | 517 |
| C0P4C1 | Uncharacterized protein | 58048.3 | 517 |
| C0P8L3 | Carboxypeptidase | 56090.6 | 517 |
| C0PMR3 | Glucose-6-phosphate 1-dehydrogenase | 58651.3 | 517 |
| K7V4K2 | Putative serine peptidase S28 family protein | 57361.3 | 517 |
| K7VEB4 | Alpha/beta-Hydrolases superfamily protein | 57219 | 517 |
| Q3SAE3 | Glucose-1-phosphate adenylyltransferase | 56717.3 | 517 |
| Q947B9 | Glucose-1-phosphate adenylyltransferase | 56481.8 | 517 |
| Q94F82 | Histone deacetylase | 57931.2 | 517 |
| A0A1D6DZK7 | Adenosylmethionine aminotransferase1 | 56667.4 | 518 |
| A0A1D6FKF7 | Aspartic proteinase A1 | 54788 | 518 |
| A0A1D6J230 | DNA repair helicase UVH6 | 58446.6 | 518 |
| A0A1D6KUM0 | Splicing factor CC1-like | 57468.5 | 518 |
| A0A1D6PZG7 | Glucose-1-phosphate adenylyltransferase | 57070.7 | 518 |
| A0A317Y7K2 | Copper transport protein ATX1 | 55439.2 | 518 |
| B4FQ24 | Cytochrome P450 family 706 subfamily A polypeptide 5 | 56552.1 | 518 |
| B6SRT2 | TIP | 57319.3 | 518 |
| B6SZ21 | Cytochrome P450 CYP81N5 | 55667.7 | 518 |
| B6TCZ8 | Glucose-1-phosphate adenylyltransferase | 57450.1 | 518 |
| B7ZXG2 | Uncharacterized protein | 56538 | 518 |
| B8A356 | Poly(ADP-ribose) glycohydrolase 1 | 57534.2 | 518 |
| A0A1D6FSY4 | Pseudouridine synthase family protein | 57140.2 | 519 |
| A0A1D6GIP1 | Adenylyl cyclase-associated protein | 55763.5 | 519 |
| A0A1D6HPF3 | Myosin2 | 59351.2 | 519 |
| A0A1D6M4F9 | Beta-hexosaminidase | 57493.8 | 519 |
| A0A1D6NNP0 | Pre-mRNA-processing protein 40A | 62780.9 | 519 |
| B6TX13 | Growth regulator like protein | 59173.2 | 519 |
| A0A1D6EM50 | Chaperonin 60 subunit alpha 2 chloroplastic | 55747 | 520 |
| A0A1D6JMR9 | Ubiquitin domain-containing protein DSK2b | 54638.9 | 520 |
| A0A1D6JNJ8 | Lethal leaf-spot 1 | 58145.8 | 520 |
| A0A1D6JSL6 | Low phytic acid1 | 57468.3 | 520 |
| A0A1D6QT91 | ILITYHIA | 57979.2 | 520 |
| A0A317Y6J2 | Pheophorbide a oxygenase, chloroplastic | 58141.9 | 520 |
| B6TB19 | Patellin-5 | 57768.3 | 520 |
| B7ZYD3 | Phosphoribosylamine--glycine ligase chloroplastic | 54901.4 | 520 |
| A0A1D6GN58 | Uncharacterized protein | 58215.9 | 521 |
| A0A1D6K7S4 | Ubiquitin domain-containing protein DSK2b | 54374.6 | 521 |
| A0A317YFU9 | Ent-cassadiene C2-hydroxylase | 57818.1 | 521 |
| B4F816 | Uncharacterized protein | 56658.3 | 521 |
| B6SXV2 | RNA-binding protein AKIP1 | 53286.4 | 521 |
| B6SZ78 | Cytochrome P450 CYP71W7 | 57855.1 | 521 |
| C0P576 | UBP1-associated protein 2A | 53415.6 | 521 |
| K7UZS2 | Galactose oxidase/kelch repeat superfamily protein | 56179.4 | 521 |
| A0A1D6E9T6 | Putative translation initiation factor IF-2 | 55991.1 | 522 |
| A0A1D6FJ37 | Ubiquitin fusion degradation UFD1 family protein | 58436.6 | 522 |
| A0A1D6HGI4 | AP-2 complex subunit mu | 58204.9 | 522 |
| A0A1D6MLM6 | Cytochrome P450 family 87 subfamily A polypeptide 6 | 57674.9 | 522 |
| A0A1D6P1C1 | Serine/threonine-protein phosphatase 2A 55 kDa regulatory subunit B | 58251.6 | 522 |
| A0A1D6Q1G1 | Dihydrolipoamide acetyltransferase component of pyruvate dehydrogenase complex | 54422.2 | 522 |
| B6TPP7 | Threonine synthase | 57130.6 | 522 |
| C0P3K4 | Uncharacterized protein | 52816.2 | 522 |
| C0P9Q9 | Putative DUF21 domain-containing protein | 55793.8 | 522 |
| A0A1D6E098 | Ubiquitin-specific protease family C19-related protein | 56489 | 523 |
| A0A1D6E223 | Proline--tRNA ligase cytoplasmic | 59774.3 | 523 |
| A0A1D6HD80 | RAN GTPase-activating protein 2 | 56830.6 | 523 |
| A0A1D6JST8 | Beta-glucosidase 40 | 58769 | 523 |
| A0A317YFR0 | Phosphomevalonate kinase, peroxisomal | 56236.5 | 523 |
| A0A317YIV1 | Beta-glucosidase 6 | 58782 | 523 |
| A0A317YJ29 | Cytochrome c1-1, heme protein, mitochondrial | 57250.4 | 523 |
| B4FTD3 | Uncharacterized protein | 57415.8 | 523 |
| B6TJY4 | Dihydrolipoamide acetyltransferase component of pyruvate dehydrogenase complex | 56789.6 | 523 |
| B6TZ71 | Glycerol kinase | 56475.2 | 523 |
| C0HE85 | Eukaryotic translation initiation factor 2A | 57078.6 | 523 |
| Q9M640 | Coatomer subunit delta | 57498 | 523 |
| A0A1D6FI02 | Sequence-specific DNA binding transcription factor | 56374 | 524 |
| A0A1D6GW74 | NF-E2 inducible protein-like | 57473.6 | 524 |
| A0A1D6HG82 | GPI transamidase subunit PIG-U | 58034.4 | 524 |
| A0A1D6JI11 | Pyruvate kinase | 56565.5 | 524 |
| A0A1D6K2X5 | Nuclear cap-binding protein subunit 1 | 59943.2 | 524 |
| A0A1D6L376 | Glucose-1-phosphate adenylyltransferase | 57458.1 | 524 |
| A0A1D6MC44 | ARM repeat superfamily protein | 57169 | 524 |
| A0A1D6MXQ4 | Cofactor-independent phosphoglycerate mutase | 56182 | 524 |
| A0A1D6PIW6 | Beta-1,4-mannosyltransferase | 58498.3 | 524 |
| A0A317Y642 | Plasma membrane ATPase 3 | 58632.7 | 524 |
| B4FI84 | Protein kinase superfamily protein | 57610.8 | 524 |
| A0A1D6ELB5 | Dolichyl-diphosphooligosaccharide--protein glycosyltransferase 48 kDa subunit | 59026.5 | 525 |
| A0A1D6EP71 | Proton pump-interactor 1 | 59267.2 | 525 |
| B4FBF2 | Carboxypeptidase | 57638.6 | 525 |
| B4FLP9 | Uncharacterized protein | 58842.5 | 525 |
| B6SSZ8 | 60S ribosomal export protein NMD3 | 58732.4 | 525 |
| B6T8X5 | T-complex protein 1 subunit beta | 57403.6 | 525 |
| B8A0D2 | Uncharacterized protein | 58409.3 | 525 |
| B8A2Z7 | DEAD-box ATP-dependent RNA helicase 8 | 59857.8 | 525 |
| C0P6C5 | Threonine synthase 1 chloroplastic | 57441.9 | 525 |
| C0PC62 | Uncharacterized protein | 57229.6 | 525 |
| C4J1M8 | ATP-dependent 6-phosphofructokinase | 57475 | 525 |
| C4J3S4 | MAP kinase kinase kinase63 | 57638.2 | 525 |
| G2XK63 | T-complex protein 1 subunit beta | 57394.6 | 525 |
| K7UPD0 | Putative TCP-1/cpn60 chaperonin family protein | 57372.5 | 525 |
| K7VGK6 | 60S ribosomal export protein NMD3 | 58964 | 525 |
| A0A1D6KRS1 | Mediator of RNA polymerase II transcription subunit 16 | 57040.5 | 526 |
| A0A1D6MHG9 | DEAD-box ATP-dependent RNA helicase 20 | 58451.9 | 526 |
| A0A1D6MLZ0 | Metallo-hydrolase/oxidoreductase superfamily protein | 57704.1 | 526 |
| A0A1D6MPN8 | Importin subunit alpha | 57659.2 | 526 |
| A0A317Y3Y6 | KH domain-containing protein | 56116.1 | 526 |
| A0A317YDD6 | 1,2-dihydroxy-3-keto-5-methylthiopentene dioxygenase | 60114.8 | 526 |
| B6SUZ9 | Importin subunit alpha | 56503.1 | 526 |
| B6TRQ7 | Pre-mRNA-processing factor 19 homolog 2 | 57595.7 | 526 |
| B8A1T6 | Folate synthesis bifunctional protein | 57912.9 | 526 |
| C0PCK9 | Putative receptor-like protein kinase | 58226.8 | 526 |
| C0PCX4 | Cytochrome P450 family 704 subfamily A polypeptide 2 | 58814.2 | 526 |
| A0A1D6FC43 | Importin subunit alpha | 57852.4 | 527 |
| A0A1D6H840 | Importin beta-like SAD2 | 59670.7 | 527 |
| A0A1D6ITT8 | Transducin/WD40 repeat-like superfamily protein | 58328.8 | 527 |
| A0A1D6K0V8 | Putative pre-mRNA-splicing factor ATP-dependent RNA helicase DEAH3 | 58665.1 | 527 |
| B4F8J8 | Pyruvate kinase | 57292.3 | 527 |
| B4F9G8 | Pyruvate kinase | 57342.4 | 527 |
| B4FYG3 | Aldehyde dehydrogenase | 56112.3 | 527 |
| B6TBN6 | Pyruvate kinase | 57281.3 | 527 |
| B6THZ8 | Threonine synthase1 | 57613.8 | 527 |
| B6TK43 | Pyruvate kinase | 57194.2 | 527 |
| B6TPI6 | Aldehyde dehydrogenase | 56176.3 | 527 |
| B6TZD9 | Eukaryotic translation initiation factor 4B | 57095.3 | 527 |
| B6UBI2 | Acyl activating enzyme | 55218.3 | 527 |
| C0HIJ7 | Cytochrome P450 709B2 | 59707.9 | 527 |
| H2BJB6 | Acyl-CoA-like protein | 55192.2 | 527 |
| K7VWF2 | Outer membrane OMP85 family protein | 57242.6 | 527 |
| A0A1D6FA15 | U3 small nucleolar RNA-associated protein 6 | 59899.7 | 528 |
| A0A1D6GLY5 | Cytochrome P450 71D7 | 58631.3 | 528 |
| A0A1D6JJ37 | Farnesylcysteine lyase | 58113.7 | 528 |
| A0A1D6K5C1 | Sterol-4-alpha-carboxylate 3-dehydrogenase, decarboxylating | 58965.5 | 528 |
| A0A1D6QK51 | Copper-transporting ATPase PAA1 chloroplastic | 56027.8 | 528 |
| C0HFI5 | ATP-dependent 6-phosphofructokinase | 57292.9 | 528 |
| C0P5C0 | Importin subunit alpha | 58131.6 | 528 |
| C0PHF7 | Uncharacterized protein | 58700.6 | 528 |
| Q8LL74 | Cytochrome P450 monooxygenase CYP72A16 | 60641.6 | 528 |
| A0A1D6EAS8 | Transcription factor bHLH95 | 54016.4 | 529 |
| A0A1D6FIA2 | Kinesin-like protein | 59696.8 | 529 |
| A0A1D6FKF4 | Aspartic proteinase A1 | 56151.6 | 529 |
| A0A1D6HP35 | Glutathione reductase | 56882.6 | 529 |
| A0A1D6L398 | Low phytic acid3 | 59515.1 | 529 |
| A0A1D6MQM1 | DNA topoisomerase 2 | 59720.7 | 529 |
| A0A317YL29 | NEDD8-activating enzyme E1 regulatory subunit | 58905.5 | 529 |
| B4FR25 | Uncharacterized protein | 56869.5 | 529 |
| B6T5L0 | KH domain containing protein | 57341 | 529 |
| C0PA67 | Tubulin beta chain | 59140.6 | 529 |
| K7VEB9 | Importin subunit alpha | 58200.7 | 529 |
| K7VV23 | Polypyrimidine tract-binding protein homolog 3 | 59172.6 | 529 |
| Q5EUD0 | Protein disulfide isomerase | 58973.7 | 529 |
| A0A1D6QIC9 | Outer envelope protein 64 chloroplastic | 58657.8 | 530 |
| A0A317YIK7 | Putative mitochondrial-processing peptidase subunit beta, mitochondrial | 58392.8 | 530 |
| B4F932 | Putative mitochondrial-processing peptidase subunit beta mitochondrial | 58378.8 | 530 |
| B6TG70 | Mitochondrial-processing peptidase beta subunit | 58442.6 | 530 |
| B6UF43 | Calcium-dependent protein kinase, isoform 2 | 59937.6 | 530 |
| K7VKM2 | Peptidase beta subunit | 58378.6 | 530 |
| A0A1D6H287 | 4-coumarate--CoA ligase 1 | 56375.6 | 531 |
| A0A1D6HSP4 | Cytochrome P-450 19 | 57703.6 | 531 |
| A0A1D6HXL9 | Calcium-dependent lipid-binding (CaLB domain) family protein | 58263.3 | 531 |
| A0A1D6ICZ3 | Calcium dependent protein kinase8 | 59365.1 | 531 |
| A0A1D6QQ83 | Serine/threonine-protein phosphatase 2A 55 kDa regulatory subunit B | 59261.7 | 531 |
| B4FE51 | Mechanosensitive ion channel protein 1 mitochondrial | 57699.8 | 531 |
| B6SY30 | Cytochrome P450 CYP84A34 | 58361.5 | 531 |
| B6T7E5 | Transcription initiation factor IIF, alpha subunit | 58367.1 | 531 |
| B6TE72 | ATP-dependent 6-phosphofructokinase | 57644.4 | 531 |
| B6TID7 | Uncharacterized protein | 58430 | 531 |
| C0HH08 | Uncharacterized protein | 58906.3 | 531 |
| C0PHM8 | Glutathione synthetase | 58334.2 | 531 |
| C4J038 | Uncharacterized protein | 59464.3 | 531 |
| Q41790 | Calcium dependent protein kinase3 | 59352.2 | 531 |
| A0A1D6F2G1 | SWI/SNF complex component SNF12-like protein | 58327.3 | 532 |
| A0A1D6GJK8 | Protein CASP | 60824.7 | 532 |
| A0A1D6HJU0 | p-loop containing nucleoside triphosphate hydrolase superfamily protein | 57901.4 | 532 |
| A0A1D6J3T2 | Putative glycosyl transferase family 28 protein | 57364.9 | 532 |
| A0A1D6LB96 | Nicotinate phosphoribosyltransferase 2 | 59007.1 | 532 |
| A0A1D6N5B0 | Protein root UVB sensitive 6 | 58318.7 | 532 |
| A0A1D6NTB9 | Uncharacterized protein | 57903.6 | 532 |
| A0A1D6P6K6 | BR-signaling kinase 2 | 59002.7 | 532 |
| A0A1D6P9Y5 | Alpha-N-acetylglucosaminidase | 61819.6 | 532 |
| A0A317Y4W4 | Alanine--glyoxylate aminotransferase 2 1, mitochondrial | 58000.6 | 532 |
| B4FAK8 | Calnexin homolog2 | 60245.5 | 532 |
| B6SWA3 | Cytochrome P450 CYP84A33v2 | 58474.7 | 532 |
| B7ZXQ4 | Uncharacterized protein | 59889.3 | 532 |
| B7ZY34 | Uncharacterized protein | 58120.4 | 532 |
| C0HFZ5 | Outer membrane OMP85 family protein | 57812.4 | 532 |
| K7VN82 | BTB/POZ domain-containing protein | 57011.9 | 532 |
| A0A1D6H0M2 | SUPPRESSOR OF AUXIN RESISTANCE1 | 60779.6 | 533 |
| A0A1D6KWM5 | DUF21 domain-containing protein chloroplastic | 59302.3 | 533 |
| A0A1D6M3I3 | Putative DUF21 domain-containing protein | 57273.8 | 533 |
| A0A1D6MHW3 | Cytokinin oxidase1 | 57156.1 | 533 |
| A0A1D6MS74 | Suppressor of mec-8 and unc-52 protein homolog 1 | 60124.5 | 533 |
| A0A1D6NJJ3 | ATP-dependent zinc metalloprotease FTSH 11 chloroplastic/mitochondrial | 58229.5 | 533 |
| A0A1D6PQL3 | Seryl-tRNA synthetase | 58419.8 | 533 |
| B4F8S1 | Alpha/beta-Hydrolases superfamily protein | 58706.9 | 533 |
| B6TNT9 | Ubiquilin-1 | 56371.7 | 533 |
| C4JAJ7 | Beta-glucosidase 11 | 59439.4 | 533 |
| A0A1D6DVI1 | Lipoxygenase6 | 58037.9 | 534 |
| A0A1D6G6A6 | AP-4 complex subunit epsilon | 56419.8 | 534 |
| A0A1D6GPU5 | Nucleotide binding protein | 58838.3 | 534 |
| A0A1D6HAP1 | Ligatin | 59391.3 | 534 |
| A0A1D6N0K9 | Uncharacterized protein | 58516 | 534 |
| A0A1D6P6J2 | Plant intracellular Ras-group-related LRR protein 4 | 58309.9 | 534 |
| A0A1D6Q167 | Methylcrotonoyl-CoA carboxylase beta chain mitochondrial | 56902.2 | 534 |
| A0A317Y155 | 3beta-hydroxysteroid-dehydrogenase/decarboxylase isoform 2 | 58849.1 | 534 |
| B6SKG0 | Non-cyanogenic beta-glucosidase | 60049.6 | 534 |
| B6T3L3 | Permease I | 58140.2 | 534 |
| B6TNF1 | Calnexin homolog1 | 60400.7 | 534 |
| K7UVH6 | Putative cytochrome P450 superfamily protein | 57984.3 | 534 |
| K7VXF0 | Triacylglycerol lipase-like 1 | 58229.5 | 534 |
| Q6KF70 | Transglutaminase | 60969.3 | 534 |
| A0A1D6FPA9 | Arginyl-tRNA--protein transferase | 59616.8 | 535 |
| A0A1D6H5J1 | Uncharacterized protein | 55068.7 | 535 |
| A0A1D6HXM2 | Calcium-dependent lipid-binding (CaLB domain) family protein | 58666.7 | 535 |
| A0A1D6J993 | RBR-type E3 ubiquitin transferase | 60578.3 | 535 |
| A0A1D6LEU6 | Pre-mRNA-splicing factor SLU7-A | 62045.7 | 535 |
| B4F8V9 | T-complex protein 1 subunit delta | 57645 | 535 |
| B4FS49 | T-complex protein 1 subunit epsilon | 58998.5 | 535 |
| B6U118 | T-complex protein 1 subunit zeta | 59086.5 | 535 |
| B6UIP3 | Tubulin-specific chaperone E | 58965.7 | 535 |
| C0HGT5 | T-complex protein 1 subunit delta | 57732.2 | 535 |
| A0A1D6DW78 | Zinc finger (Ubiquitin-hydrolase) domain-containing protein | 59258.8 | 537 |
| A0A1D6F1X1 | Phospho-2-dehydro-3-deoxyheptonate aldolase | 58998.8 | 537 |
| A0A1D6GNV7 | Flowering locus K homology domain | 59001.9 | 537 |
| A0A1D6ILV0 | Glycosyltransferase family 61 protein | 59804.7 | 537 |
| A0A1D6J4Q8 | Translation elongation factor EF1A/initiation factor IF2gamma family protein | 58929 | 537 |
| A0A1D6PEC5 | ERAD-associated E3 ubiquitin-protein ligase component HRD3A | 60945 | 537 |
| B4G066 | Methylmalonate-semialdehyde dehydrogenase [acylating] mitochondrial | 57517.3 | 537 |
| B6TJE8 | Prolyl-tRNA synthetase | 59668.7 | 537 |
| A0A1D6KEP0 | Serine hydroxymethyltransferase | 58546.3 | 538 |
| A0A1D6P1B7 | Beta-glucosidase 11 | 60351.2 | 538 |
| A0A1D6Q8C1 | Protein CROWDED NUCLEI 1 | 63531.8 | 538 |
| B4FQJ2 | Dihydropyrimidinase | 57729.7 | 538 |
| B6TUB8 | Phospho-2-dehydro-3-deoxyheptonate aldolase | 59003.8 | 538 |
| C0P472 | Protein TIC 55 chloroplastic | 59406.2 | 538 |
| C0P7J2 | FHA domain-containing protein DDL | 61974.7 | 538 |
| A0A1D6IGS1 | Elongation initiation factor2 | 58152.3 | 539 |
| A0A1D6LE84 | EMBRYO DEFECTIVE 140 | 60948.3 | 539 |
| B6TBV7 | Calcium lipid binding protein-like | 61012.4 | 539 |
| B6TUA2 | Acetyltransferase component of pyruvate dehydrogenase complex | 58381.9 | 539 |
| B6UDD6 | Alpha-L-fucosidase 1 | 59549.1 | 539 |
| C0P5F2 | Uncharacterized protein | 60085 | 539 |
| C4J2I7 | Uncharacterized protein | 60630.6 | 539 |
| K7VTH8 | Putative calcium-dependent protein kinase family protein | 60731.4 | 539 |
| A0A1D6G0C7 | Rubisco methyltransferase family protein | 60437.7 | 540 |
| A0A1D6GPL1 | Serine/threonine protein phosphatase 2A regulatory subunit B''alpha | 61779.4 | 540 |
| A0A1D6GW71 | NF-E2 inducible protein-like | 59068.4 | 540 |
| A0A1D6JCC2 | Putative polyphenol oxidase family protein | 59115.3 | 540 |
| A0A317YES4 | Serine/threonine protein phosphatase 2A regulatory subunit B''alpha | 61829.5 | 540 |
| B6SVH9 | Uncharacterized protein | 59307.2 | 540 |
| B6T7S1 | Chaperone protein dnaJ 13 | 60354.5 | 540 |
| K7VV41 | Amidophosphoribosyltransferase | 58093.4 | 540 |
| A0A1D6EEC1 | Putative translation elongation/initiation factor family protein | 59297.6 | 541 |
| A0A1D6FY14 | ATP-dependent zinc metalloprotease FTSH 4 mitochondrial | 58145.1 | 541 |
| A0A1D6IJP9 | Alanine aminotransferase9 | 58738.3 | 541 |
| A0A1D6LLU5 | Prolyl oligopeptidase family protein | 60748.7 | 541 |
| B6T5J4 | Ran GTPase activating protein | 59089.9 | 541 |
| C0PGJ3 | Cytochrome P450 71A26 | 60341.3 | 541 |
| C4J9J4 | RNA-binding KH domain-containing protein | 57812.9 | 541 |
| A0A1D6HD59 | Polyribonucleotide nucleotidyltransferase 2 mitochondrial | 58279.7 | 542 |
| A0A1D6NY88 | Asparagine synthetase1 | 60690.5 | 542 |
| B4FRW5 | Pyruvate kinase | 59223.4 | 542 |
| C0PG82 | Protein FREE1 | 57640.1 | 542 |
| C0PKZ3 | Non-specific phospholipase C1 | 60587.2 | 542 |
| Q9LLD9 | Hexose transporter (Fragment) | 56941.8 | 542 |
| Q9SWR9 | Acetyltransferase component of pyruvate dehydrogenase complex | 58331.6 | 542 |
| A0A1D6GPF1 | Putative eukaryotic translation initiation factor 5-2 | 59229.5 | 543 |
| A0A1D6M5J5 | Amidophosphoribosyltransferase | 58381.6 | 543 |
| A0A1D6MK45 | Retrovirus-related Pol polyprotein LINE-1 | 61880.1 | 543 |
| A0A1D6QUM7 | Signal peptide peptidase | 59528.9 | 543 |
| A0A317Y6L8 | Adenylosuccinate lyase | 60339.6 | 543 |
| B4F8Z3 | Adenylosuccinate lyase | 60579.8 | 543 |
| B4FGA1 | Heparanase-like protein 3 | 58269 | 543 |
| B4FLB2 | Adenylosuccinate lyase | 60283.6 | 543 |
| B6SXU7 | Heparanase-like protein 3 | 58299.1 | 543 |
| A0A1D6EEY0 | Protein phosphatase homolog10 | 57447.8 | 544 |
| A0A1D6GU11 | EH domain-containing protein 1 | 61377 | 544 |
| A0A1D6MBD6 | Phosphoribosylamine--glycine ligase chloroplastic | 57532.3 | 544 |
| B4F9N9 | Protoporphyrinogen oxidase | 59046.4 | 544 |
| B4FL26 | TLD family protein | 58595.9 | 544 |
| B4G0Y2 | Acyl-CoA-binding domain-containing protein 3 | 57930.7 | 544 |
| B6UBU1 | Glucan endo-1,3-beta-glucosidase 3 | 57038.9 | 544 |
| C0PEX3 | Calcium-dependent protein kinase 6 | 61650.8 | 544 |
| A0A1D6FGX9 | Aspartate--tRNA ligase 2 cytoplasmic | 60639.7 | 545 |
| A0A1D6GDN3 | Ubiquinone biosynthesis monooxygenase COQ6, mitochondrial | 59319.3 | 545 |
| A0A1D6H1K6 | Tubulin-folding cofactor E | 60087.1 | 545 |
| A0A1D6HAU2 | Chloride channel | 59126.6 | 545 |
| A0A1D6HN01 | T-complex protein 1 subunit alpha | 59265.6 | 545 |
| A0A1D6J9Q4 | Polyadenylate-binding protein | 60225.4 | 545 |
| A0A1D6KJS7 | FHA domain-containing protein FHA2 | 59560.2 | 545 |
| A0A317Y696 | FRIGIDA-like protein | 59950.9 | 545 |
| A0A317YEX4 | Multiple inositol polyphosphate phosphatase 1 | 60957.4 | 545 |
| B6ST04 | Beta-hexosaminidase | 60566.3 | 545 |
| B6SVD7 | Aspartic-type endopeptidase/ pepsin A | 56789 | 545 |
| B6TKY3 | T-complex protein 1 subunit alpha | 59194.6 | 545 |
| B7ZX71 | Aspartate--tRNA ligase 2 cytoplasmic | 60639.8 | 545 |
| B8A2G2 | Transcription initiation factor TFIID subunit 6 | 60291.1 | 545 |
| C0PH60 | GMP synthase [glutamine-hydrolyzing] | 59393.6 | 545 |
| Q41728 | Carbonic anhydrase | 59190.5 | 545 |
| A0A1D6HSP6 | Outer envelope protein 61 | 59775.4 | 546 |
| A0A1D6JPH5 | Glutathione reductase | 58809.6 | 546 |
| A0A1D6JWS7 | ERAD-associated E3 ubiquitin-protein ligase component HRD3A | 61638.1 | 546 |
| A0A1D6PVM6 | Regulator of Vps4 activity in the MVB pathway protein | 60301.1 | 546 |
| C0P4T5 | Aspartate--tRNA ligase 2 cytoplasmic | 60795.8 | 546 |
| K7U772 | Glutamyl-tRNA(Gln) amidotransferase subunit B, chloroplastic/mitochondrial | 60310.6 | 546 |
| A0A1D6F303 | Glycosyltransferase family 61 protein | 60434.4 | 547 |
| A0A1D6FZV8 | Beta-adaptin-like protein | 61241 | 547 |
| A0A1D6G6E6 | Dolichyl-diphosphooligosaccharide--protein glycosyltransferase subunit 2 | 59098 | 547 |
| A0A1D6GY41 | Ferrochelatase | 60378.6 | 547 |
| A0A1D6JFB6 | Formation of crista junctions protein 1 | 60320.9 | 547 |
| A0A1D6NK05 | ATP-dependent zinc metalloprotease FTSH 4 mitochondrial | 58583.7 | 547 |
| C0P867 | Calcium dependent protein kinase5 | 60359.9 | 547 |
| A0A1D6HP12 | Octicosapeptide/Phox/Bem1p family protein | 57340.8 | 548 |
| A0A1D6HRM1 | T-complex protein 1 subunit epsilon | 60463.2 | 548 |
| A0A1D6IZ65 | SET domain-containing protein | 60286.2 | 548 |
| A0A1D6JYM2 | Glutathione S-transferase L2 chloroplastic | 61325.7 | 548 |
| A0A1D6K378 | Nucleolar protein NOP56-like protein | 60750.8 | 548 |
| A0A1D6L2B0 | Dihydroxyacetone kinase | 56559.5 | 548 |
| A0A1D6NKT6 | SIT4 phosphatase-associated family protein | 61622.4 | 548 |
| A0A1D6PLH1 | Brown midrib4 | 60098.3 | 548 |
| A0A1D6QGN6 | Cysteine desulfurase 1 chloroplastic | 59535.9 | 548 |
| A0A1D6QVF4 | Uridine kinase | 61217.9 | 548 |
| A0A1Q0XLC1 | Terpene synthase6 | 63937.7 | 548 |
| K7UCH1 | Putative TCP-1/cpn60 chaperonin family protein | 58906 | 548 |
| K7VEQ6 | Non-specific serine/threonine protein kinase | 62465.4 | 548 |
| A0A1D6IBP5 | Asparagine--tRNA ligase chloroplastic/mitochondrial | 60908.5 | 549 |
| A0A1D6IV37 | Alpha-galactosidase | 62281.9 | 549 |
| A0A1D6LQY2 | Phox (PX) domain-containing protein | 60899.7 | 549 |
| B6TA37 | Protein RCC2 | 58115.4 | 549 |
| C0HIL2 | Uncharacterized protein | 60445.5 | 549 |
| K7TUK0 | U2 snRNP auxiliary factor large subunit | 61009 | 549 |
| Q43274 | Aldehyde dehydrogenase | 59445.6 | 549 |
| Q8W0Q2 | Putative aldehyde dehydrogenase MIS1 | 60567.4 | 549 |
| A0A1D6ELZ2 | Nuclear pore complex protein GP210 | 60267.2 | 550 |
| A0A1D6MF47 | Eukaryotic translation initiation factor 3 subunit L | 64172.1 | 550 |
| B8A2U9 | Alkaline/neutral invertase CINV2 | 62840.7 | 550 |
| Q3T7E5 | X1 (Fragment) | 63294.5 | 550 |
| A0A1D6KCV3 | NAD(P)H-hydrate epimerase | 60793 | 551 |
| B4G058 | 4-coumarate--CoA ligase-like 5 | 59442.2 | 551 |
| C0PKN3 | Uncharacterized protein | 61165.8 | 551 |
| Q2UVH9 | Phosphomethylpyrimidine kinase/thiamin-phosphate pyrophosphorylase | 57755.9 | 551 |
| Q768R2 | Plastidic general dicarboxylate transporter | 58658.2 | 551 |
| Q768R3 | Dicarboxylate transporter 2.1, chloroplastic | 58600.1 | 551 |
| A0A1D6F7X9 | Sister-chromatid cohesion protein 3 | 62594.1 | 552 |
| A0A1D6II11 | Calcium-dependent lipid-binding (CaLB domain) family protein | 57913.3 | 552 |
| A0A1D6MB68 | Putative aldehyde dehydrogenase MIS1 | 60637.6 | 552 |
| A0A1D6Q3U9 | Glycerophosphodiester phosphodiesterase GDPDL3 | 58944.8 | 552 |
| A0A317YCN1 | Vicilin-like seed storage protein | 59736.7 | 552 |
| B4FN41 | Uncharacterized protein | 61505.5 | 552 |
| B4FZF8 | Nicalin | 60293.2 | 552 |
| C4IZK3 | Uncharacterized protein | 60324 | 552 |
| A0A1D6FJP5 | ATP synthase subunit beta | 59006.8 | 553 |
| A0A1D6I6C9 | S-adenosyl-L-methionine-dependent methyltransferase superfamily protein | 61194.9 | 553 |
| A0A1D6J4Q6 | Translation elongation factor EF1A/initiation factor IF2gamma family protein | 61021.6 | 553 |
| A0A1D6K268 | Vicilin-like seed storage protein | 60008.8 | 553 |
| A0A1D6M2Q6 | Sorting nexin 2B | 60600.2 | 553 |
| A9P711 | Phosphoinositide phospholipase C | 62913.2 | 553 |
| B4FZG8 | GeBP transcription factor | 59883.7 | 553 |
| B6SVV9 | ATP synthase subunit beta | 58978.7 | 553 |
| B6TI78 | Peptidylprolyl isomerase | 61691 | 553 |
| C0HF32 | ATP synthase subunit beta | 59102.9 | 553 |
| A0A1D6GMN0 | Ypt/Rab-GAP domain of gyp1p superfamily protein | 62256 | 554 |
| A0A1D6J8J1 | RNA binding (RRM/RBD/RNP motifs) family protein | 61958.8 | 554 |
| A0A1D6LAT0 | Cellulose synthase-6 | 63407.7 | 554 |
| A0A1D6LWI0 | Putative AMP-dependent synthetase and ligase superfamily protein | 60225.8 | 554 |
| A0A1D6NLC1 | Pentatricopeptide repeat-containing protein | 61130.9 | 554 |
| B6SVG7 | Chitin-inducible gibberellin-responsive protein 2 | 60893.1 | 554 |
| C0P6K9 | GRAS transcription factor | 60835 | 554 |
| Q768R4 | Plastidic general dicarboxylate transporter | 59562.3 | 554 |
| A0A096S078 | 4-coumarate--CoA ligase-like 7 | 59455.9 | 555 |
| A0A1D6E141 | Peroxisome biogenesis protein 6 | 60230.4 | 555 |
| A0A1D6F8N4 | Coatomer subunit gamma | 61359.1 | 555 |
| A0A1D6FT76 | Non-specific serine/threonine protein kinase | 64269 | 555 |
| A0A1D6IC30 | Malic enzyme | 61962.5 | 555 |
| A0A1D6LK99 | SET domain-containing protein | 61496.4 | 555 |
| A0A1D6MQK0 | Rab proteins geranylgeranyltransferase component | 59656.9 | 555 |
| A0A1D6NHX9 | Putative transcription factor | 63246.1 | 555 |
| B4FFD2 | Cellulase (Glycosyl hydrolase family 5) protein | 61160.5 | 555 |
| B4G146 | D-lactate dehydrogenase [cytochrome] mitochondrial | 60133.9 | 555 |
| K4JFK6 | GeBP-type transcription factor (Fragment) | 60297.8 | 555 |
| Q6Q297 | 4-coumarate coenzyme A ligase | 59870.5 | 555 |
| A0A096QFX2 | Nucleobase-ascorbate transporter 3 | 60500.3 | 556 |
| A0A1D6E8D9 | Eukaryotic translation initiation factor 4B2 | 60111.1 | 556 |
| A0A1D6GJ42 | Exportin-2 | 61247.5 | 556 |
| A0A1D6L222 | Exportin-2 | 61252.3 | 556 |
| A0A1D6M5V5 | Cysteine--tRNA ligase 1 cytoplasmic | 63921.1 | 556 |
| B6SH65 | Cysteine--tRNA ligase 1 cytoplasmic | 63998.3 | 556 |
| B6U076 | WD-repeat protein 50 | 61835.3 | 556 |
| C0HHU2 | 23-bisphosphoglycerate-independent phosphoglycerate mutase 1 | 60289 | 556 |
| C0PAX7 | Non-specific serine/threonine protein kinase | 64309.9 | 556 |
| C3UZ61 | CDPK protein | 61238.9 | 556 |
| K7V6Z1 | Putative patellin family protein | 61327.9 | 556 |
| A0A1D6NXN4 | Acyl-coenzyme A oxidase | 61990.4 | 557 |
| A0A317Y154 | 4-coumarate--CoA ligase-like 1 | 58918.8 | 557 |
| B8A3C8 | Transducin/WD40 repeat-like superfamily protein | 58997.3 | 557 |
| A0A1D6F6Y8 | T-complex protein 1 subunit theta | 60200.6 | 558 |
| A0A1D6H1C9 | FAD/NAD(P)-binding oxidoreductase family protein | 60579.7 | 558 |
| A0A1D6H1X6 | Protein STRUBBELIG-RECEPTOR FAMILY 1 | 61383.1 | 558 |
| A0A1D6JSN1 | T-complex protein 1 subunit gamma | 60732.7 | 558 |
| A0A1D6JXW0 | Protein TOC75-3 chloroplastic | 62016.7 | 558 |
| A0A1D6K2M1 | U3 ribonucleoprotein (Utp) family protein | 64023.4 | 558 |
| A0A1D6L8W5 | Uncharacterized protein | 62401.1 | 558 |
| A0A1D6LNA5 | Mitogen-activated protein kinase | 63713.7 | 558 |
| A0A1D6MDR4 | ADP glucose pyrophosphorylase2 | 61950.2 | 558 |
| A0A1D6N2E5 | Retrovirus-related Pol polyprotein LINE-1 | 60690.1 | 558 |
| A0A317Y8G7 | Protein tesmin/TSO1-like CXC 2 | 60952.2 | 558 |
| B6T7E3 | Long-chain-fatty-acid-CoA ligase-like protein | 61126.6 | 558 |
| B6TJS6 | MAC/Perforin domain containing protein | 61711.9 | 558 |
| C0PEL1 | MACPF domain-containing protein CAD1 | 61725.9 | 558 |
| C0PL01 | T-complex protein 1 subunit gamma | 60685.6 | 558 |
| C4J3M2 | Malic enzyme | 61381.1 | 558 |
| A0A1D6E7V9 | Malate synthase | 61843.6 | 559 |
| A0A1D6FAT4 | Retrotransposon protein SINE subclass | 60928.9 | 559 |
| A0A1D6FH92 | KH domain-containing protein HEN4 | 60228.7 | 559 |
| A0A1D6H7H1 | NSP (Nuclear shuttle protein)-interacting GTPase | 60596.2 | 559 |
| A0A1D6HSP5 | Outer envelope protein 61 | 61299.1 | 559 |
| A0A1D6IHR1 | Uncharacterized protein | 59041.6 | 559 |
| A0A1D6L914 | Mitochondrial Rho GTPase 1 | 62125.8 | 559 |
| A0A317YDJ6 | Uncharacterized protein | 64873.3 | 559 |
| B6TME5 | Aminotransferase, class IV family protein | 62308.3 | 559 |
| B6U6U9 | Uncharacterized protein | 59139.8 | 559 |
| B8A306 | 2,3-bisphosphoglycerate-independent phosphoglycerate mutase | 60629.2 | 559 |
| C0P5I5 | Alkaline/neutral invertase CINV2 | 63133 | 559 |
| P49081 | Malate synthase, glyoxysomal | 61636.3 | 559 |
| A0A1D6FXM0 | Vacuolar protein-sorting protein 33 | 62860.1 | 560 |
| A0A1D6KKI7 | Glycine--tRNA ligase mitochondrial 1 | 62755.5 | 560 |
| A0A1D6MAI6 | U3 small nucleolar ribonucleoprotein protein MPP10 | 62713.7 | 560 |
| A0A1D6NUU8 | Starch synthase, chloroplastic/amyloplastic | 62107.1 | 560 |
| A0A1D6QS28 | Putative STRUBBELIG family receptor protein kinase | 61418.1 | 560 |
| B4FBP0 | T-complex protein 1 subunit eta | 60299.4 | 560 |
| B6SVB4 | Uncharacterized protein | 61293.2 | 560 |
| A0A1D6GPK1 | Serine/threonine protein phosphatase 2A regulatory subunit B''alpha | 64308.2 | 561 |
| A0A1D6JM96 | C-terminal binding protein AN | 60861.6 | 561 |
| A0A1D6L917 | Mitochondrial Rho GTPase 1 | 62826.8 | 561 |
| A0A1D6QT57 | L-arabinokinase | 60848.4 | 561 |
| C0P5B0 | Asparaginyl-tRNA synthetase, cytoplasmic 3 | 62663.1 | 561 |
| C0PHQ1 | Cysteine--tRNA ligase 1 cytoplasmic | 64528.7 | 561 |
| Q5EUD8 | Protein disulfide-isomerase | 61888 | 561 |
| A0A096QRL3 | Nana plant2 | 65110.8 | 562 |
| A0A1D6M4Z8 | Glycosyl hydrolase family protein | 60774.7 | 562 |
| A0A1D6PJ93 | Pentatricopeptide repeat-containing protein mitochondrial | 61708.3 | 562 |
| B4F9F0 | CDK5RAP3-like protein | 62739.9 | 562 |
| B4F9J2 | Lipid binding protein | 63598.9 | 562 |
| B4FWJ7 | D-2-hydroxyglutarate dehydrogenase mitochondrial | 61278.4 | 562 |
| B6T2L5 | CDK5RAP3-like protein | 62725.8 | 562 |
| B6T9R4 | Xylulose kinase | 61668.4 | 562 |
| K7UW74 | Dihydrolipoyl dehydrogenase | 59712 | 562 |
| A0A1D6ELR2 | p-loop NTPase domain-containing protein LPA1 homolog 1 | 62473.9 | 563 |
| A0A1D6F298 | DNAJ heat shock N-terminal domain-containing protein | 63237.2 | 563 |
| A0A1D6G875 | Putative ubiquitin-like-specific protease 2B | 60867 | 563 |
| A0A1D6H898 | Polyadenylate-binding protein-interacting protein 3 | 61752.9 | 563 |
| A0A1D6IBB0 | Uncharacterized protein | 62882.4 | 563 |
| A0A1D6K7M5 | Hexose transporter | 60523.5 | 563 |
| A0A1D6MLT0 | Cytochrome P450 family 77 protein | 59466.6 | 563 |
| A0A1D6PJL1 | Aconitase3 | 61543.3 | 563 |
| B6TAX4 | MON1 | 62721.7 | 563 |
| B8A2X5 | Pectinesterase | 60093.6 | 563 |
| C0HG18 | Glutamate--tRNA ligase chloroplastic/mitochondrial | 62468.5 | 563 |
| K7TGE1 | Beta-glucosidase2 | 64120 | 563 |
| K7VAN4 | Putative DUF1421 domain family protein | 59267.3 | 563 |
| A0A1D6GH80 | ATP binding protein | 62903.6 | 564 |
| A0A1D6PW03 | Protein PAF1-like protein | 63687.3 | 564 |
| A0A1D6QNS8 | Uncharacterized protein | 61656.6 | 564 |
| B4FQM2 | Pyrophosphate--fructose 6-phosphate 1-phosphotransferase subunit beta | 61018.1 | 564 |
| C0P567 | Pyrophosphate--fructose 6-phosphate 1-phosphotransferase subunit beta | 61051.2 | 564 |
| C4J601 | ATP-dependent 6-phosphofructokinase | 61941.5 | 564 |
| A0A1D6IYV6 | Evolutionarily conserved C-terminal region 10 | 61868 | 565 |
| A0A1D6PSY9 | Bifunctional dihydrofolate reductase-thymidylate synthase | 63853.8 | 565 |
| B6UCT9 | Nucleotide binding protein | 60378.8 | 565 |
| B8A027 | Uncharacterized protein | 62332.5 | 565 |
| C0PHH5 | Dihydrolipoyl dehydrogenase | 59353.5 | 565 |
| C0PHV7 | alpha-1,2-Mannosidase | 64268.9 | 565 |
| A0A1D6E496 | RNA recognition water-stress protein1 | 63314.3 | 566 |
| A0A1D6F2D9 | Synaptotagmin-5 | 64040.5 | 566 |
| A0A1D6FB00 | DNA-binding protein | 63895.2 | 566 |
| A0A1D6LDX4 | KH domain-containing protein | 61652.6 | 566 |
| A0A1D6M009 | Heat shock 70 kDa protein 14 | 62339.7 | 566 |
| A0A1D6MU35 | Mitogen-activated protein kinase kinase 3 | 63516.6 | 566 |
| B4FB16 | Uncharacterized protein | 62431.1 | 566 |
| A0A1D6EPV9 | ATP-dependent 6-phosphofructokinase 5 chloroplastic | 62602.2 | 567 |
| A0A1D6FJX1 | Golgin candidate 5 | 65519.9 | 567 |
| A0A1D6IZ80 | Regulator of Vps4 activity in the MVB pathway protein | 62925.5 | 567 |
| A0A1D6PTZ4 | Evolutionarily conserved C-terminal region 10 | 62211.1 | 567 |
| A0A1D6QAB1 | Putative integral membrane protein conserved region (DUF2404) | 62449.5 | 567 |
| B4G0N0 | Glucose-6-phosphate isomerase | 62236.8 | 567 |
| B6SVZ0 | Beta-amylase | 63071.1 | 567 |
| B6TVE3 | Patellin-1 | 61748.3 | 567 |
| B6UDC0 | Vacuolar protein-sorting protein 45 | 64535.6 | 567 |
| A0A1D6DWJ6 | Ubiquitin-associated (UBA)/TS-N domain-containing protein | 62046.5 | 568 |
| A0A1D6JNW8 | Transducin family protein / WD-40 repeat family protein | 61942.7 | 568 |
| A0A1D6M465 | Uncharacterized protein | 61990.2 | 568 |
| A0A1D6MRB8 | ABC transporter B family member 21 | 62450.4 | 568 |
| A0A1D6N2F8 | Aspartokinase 1 chloroplastic | 61411.4 | 568 |
| A0A317Y7P8 | Monocopper oxidase-like protein SKU5 | 62426.8 | 568 |
| A0A317YGB1 | Galactokinase | 61029 | 568 |
| B4FEC3 | Pyruvate kinase | 62538.9 | 568 |
| B4FQ71 | Uncharacterized protein | 59731.8 | 568 |
| B4FRW6 | Uncharacterized protein | 61246.1 | 568 |
| B4FYH2 | Pyruvate kinase | 61478.5 | 568 |
| Q5EUD9 | Protein disulfide-isomerase | 62416.5 | 568 |
| A0A096SC75 | Dentin sialophosphoprotein-related | 61932 | 569 |
| A0A1D6KLM9 | Structural maintenance of chromosomes (SMC) family protein | 66153.3 | 569 |
| A0A1D6L8X0 | Uncharacterized protein | 63525.5 | 569 |
| A0A1D6N0A4 | Pentatricopeptide repeat-containing protein chloroplastic | 63291.1 | 569 |
| B4FLB0 | Uncharacterized protein | 63387.3 | 569 |
| B6SKY3 | Cytochrome P450 CYP727A4 | 62571.1 | 569 |
| B6U0E2 | Uncharacterized protein | 61881.6 | 569 |
| K7VIA0 | Nucleolar protein NOP56-like protein | 63156.4 | 569 |
| A0A1D6FCY4 | Beta-glucosidase 44 | 63351.9 | 570 |
| A0A1D6FJ36 | Ubiquitin fusion degradation UFD1 family protein | 63583.4 | 570 |
| A0A1D6FQN8 | Malic enzyme | 62703 | 570 |
| A0A1D6FYS0 | Transcriptional corepressor LEUNIG | 62249.3 | 570 |
| A0A1D6JS43 | Peptidyl-prolyl cis-trans isomerase CYP65 | 62862.7 | 570 |
| A0A1D6MAN7 | Dolichyl-diphosphooligosaccharide--protein glycosyltransferase subunit STT3A | 63534.7 | 570 |
| A0A317YBT1 | CDPK-related kinase 8 | 63037.5 | 570 |
| A5PJ46 | Zeta-carotene desaturase | 63126.7 | 570 |
| B4FJY4 | Elongator complex protein 3 | 63765.7 | 570 |
| B6THD0 | Uncharacterized protein | 62102.4 | 570 |
| B6TVG1 | Malic enzyme | 62893.4 | 570 |
| B6TW66 | Cytosolic purine 5-nucleotidase | 64698 | 570 |
| A0A1D6GNF8 | Uncharacterized protein | 58756.7 | 571 |
| A0A1D6I8D5 | Beta-glucosidase 11 | 63769.4 | 571 |
| A0A1D6K146 | Uncharacterized protein | 63514.3 | 571 |
| A0A1D6L0W8 | ARM repeat superfamily protein | 63771.5 | 571 |
| A0A1D6LLP8 | AP-1 complex subunit gamma-1 | 64094.7 | 571 |
| A0A1D6MG93 | Mannosyltransferase | 64515.5 | 571 |
| A0A1D6MXP0 | Golgin candidate 6 | 63248.4 | 571 |
| A0A1D6NG11 | ATP binding protein | 64000.9 | 571 |
| A0A1D6NUE9 | Ribonucleoside-diphosphate reductase large subunit | 64116.2 | 571 |
| A0A1D6P5D3 | Protein STRUBBELIG-RECEPTOR FAMILY 1 | 62951.5 | 571 |
| A0A1D6PPK0 | Nuclear pore complex protein | 64139.7 | 571 |
| A0A317YFE1 | Protein misato 1 | 62529.4 | 571 |
| C0PGU3 | Elongator complex protein 3 | 63729.7 | 571 |
| C4J008 | 15-cis-phytoene desaturase chloroplastic/chromoplastic | 64145.2 | 571 |
| P49086 | 15-cis-phytoene desaturase, chloroplastic/chromoplastic | 64115.2 | 571 |
| A0A1D6DXS9 | Uncharacterized protein | 62054.6 | 572 |
| A0A1D6EDJ5 | DnaJ protein ERDJ2A | 63950.5 | 572 |
| A0A1D6ET31 | Reticulon-like protein | 61985.8 | 572 |
| A0A1D6K7R4 | Ubiquitin domain-containing protein DSK2b | 59200.7 | 572 |
| A0A1D6PF93 | Transmembrane 9 superfamily member | 65413.2 | 572 |
| A0A1X7YIM9 | Uncharacterized protein | 60519.5 | 572 |
| B4FTF9 | Isocitrate lyase | 62682.5 | 572 |
| B6SRT0 | Isocitrate lyase | 62680.5 | 572 |
| B6SXN4 | Beta-amylase | 61532.9 | 572 |
| B6UBK2 | Zeta-carotene desaturase | 63089.8 | 572 |
| A0A1D6F6Y9 | T-complex protein 1 subunit theta | 61804.4 | 573 |
| A0A1D6FCR1 | Deoxy xylulose reductoisomerase2 | 63090.4 | 573 |
| A0A1D6HSV7 | Hua enhancer1 | 63502.9 | 573 |
| A0A1D6L185 | Mitogen-activated protein kinase kinase kinase 1 | 61901.3 | 573 |
| A0A1D6NK00 | ATP-dependent zinc metalloprotease FTSH 4 mitochondrial | 61687.8 | 573 |
| A0A317Y0I7 | Plant intracellular Ras-group-related LRR protein 4 | 61852.9 | 573 |
| B6SYP0 | Beta-amylase | 61692.1 | 573 |
| B6U6X1 | Uncharacterized protein | 62749 | 573 |
| C0P9F2 | Pleckstrin homology (PH) domain-containing protein | 62540.9 | 573 |
| A0A1D6GPK2 | Serine/threonine protein phosphatase 2A regulatory subunit B''alpha | 65667.7 | 574 |
| A0A1D6M805 | Topoisomerase II-associated protein PAT1 | 63336.9 | 574 |
| A0A1D6MTI4 | tRNA (guanine(37)-N1)-methyltransferase | 65000.4 | 574 |
| A0A1D6QRF4 | Tubulin binding cofactor C domain-containing protein | 62757.6 | 574 |
| A0A317YEM9 | Pyruvate kinase | 62585.7 | 574 |
| B6T9G4 | Mannosyltransferase | 64837.9 | 574 |
| B7ZWU7 | Uncharacterized protein | 65978.9 | 574 |
| C0P428 | Tubulin binding cofactor C domain-containing protein | 62518.1 | 574 |
| A0A1D6ECA1 | Protein AUXIN SIGNALING F-BOX 3 | 64260.8 | 575 |
| A0A1D6MAI0 | ATP-dependent 6-phosphofructokinase | 63220.7 | 575 |
| A0A1D6NYI1 | ARM repeat superfamily protein | 63994.3 | 575 |
| A0A317Y4H8 | Uncharacterized protein | 61992.6 | 575 |
| B6U1T9 | 2-hydroxyphytanoyl-CoA lyase | 60234.3 | 575 |
| B6U4K3 | Vacuolar sorting receptor 3 | 64330.6 | 575 |
| C0PGB5 | Pyruvate kinase | 62638.2 | 575 |
| C4J0G6 | Uncharacterized protein | 63309.1 | 575 |
| A0A1D6GNW8 | ALG2-interacting protein X | 63951.7 | 576 |
| A0A1D6QTB5 | Glutamate--tRNA ligase chloroplastic/mitochondrial | 63722.9 | 576 |
| A0A1R3MBV7 | Chaperonin CPN60-2, mitochondrial | 60934.6 | 576 |
| A0A221I3S0 | Ubiquilin1-like protein | 59701.2 | 576 |
| B4FSU9 | Hydrolase, hydrolyzing O-glycosyl compounds | 62447.6 | 576 |
| B7ZZV1 | Mitogen-activated protein kinase | 65474.5 | 576 |
| C0PFA7 | Ubiquitin domain-containing protein DSK2b | 59690.3 | 576 |
| C0PGN6 | Uncharacterized protein | 62820.1 | 576 |
| A0A1D6FAV4 | Protoporphyrinogen oxidase | 61502.7 | 577 |
| A0A1D6GDN4 | Ubiquinone biosynthesis monooxygenase COQ6, mitochondrial | 63259.7 | 577 |
| A0A1D6J3T3 | Putative glycosyl transferase family 28 protein | 62298.5 | 577 |
| A0A1D6KJ04 | Plant intracellular Ras-group-related LRR protein 4 | 62347.4 | 577 |
| A0A1D6LA97 | Anthranilate synthase alpha subunit 2 chloroplastic | 64487 | 577 |
| A0A1D6LDP5 | Phosphoinositide phosphatase SAC1 | 65360 | 577 |
| A0A1D6N2N9 | DEK domain-containing chromatin associated protein | 63263.3 | 577 |
| A0A1D6NGT8 | Pyruvate kinase | 62808.4 | 577 |
| A0A1Q0ZL30 | Chaperonin 1 | 61211 | 577 |
| B6SVE6 | Uncharacterized protein | 64816.5 | 577 |
| B7ZWU2 | Acetyl-coenzyme A synthetase | 64087.7 | 577 |
| A0A1D6G6A7 | AP-4 complex subunit epsilon | 64802.1 | 578 |
| A0A1D6HAJ0 | Phosphoinositide phosphatase SAC6 | 65617.5 | 578 |
| A0A1D6NC66 | MACPF domain-containing protein CAD1 | 63762.4 | 578 |
| A0A317YA69 | Ubiquitin domain-containing protein DSK2b | 60015.6 | 578 |
| B4G0Z0 | Protein KINESIN LIGHT CHAIN-RELATED 1 | 63205.7 | 578 |
| B6SWZ4 | Methylcrotonoyl-CoA carboxylase beta chain mitochondrial | 61532.6 | 578 |
| B7ZXR4 | Heat shock 70 kDa protein 8 | 62467.5 | 578 |
| K7VM84 | Folylpolyglutamate synthase | 63599.1 | 578 |
| Q768R5 | Oxo-glutarate/malate transporter1 | 59887.9 | 578 |
| A0A1D6IWN4 | Shikimate dehydrogenase1 | 62425.1 | 579 |
| A0A1D6LJS9 | Chaperonin 60 subunit beta 2 chloroplastic | 61850.3 | 579 |
| A0A1D6Q9M2 | Aldehyde dehydrogenase2 | 62261.5 | 579 |
| A0A1D6QIC2 | Outer envelope protein 64 chloroplastic | 64485.6 | 579 |
| B4FFZ2 | Ketol-acid reductoisomerase | 63002.2 | 579 |
| B6TF69 | Ketol-acid reductoisomerase | 63046.2 | 579 |
| K7UIK0 | Late embryogenesis abundant domain-containing protein / LEA domain-containing protein | 60402.9 | 579 |
| A0A1D6JWA9 | Glyoxal oxidase | 62469.2 | 580 |
| A0A1D6KZS7 | ATPase 4 plasma membrane-type | 64928.7 | 580 |
| A0A1D6MM59 | Uncharacterized protein | 62965.8 | 580 |
| A0A1D6QGL4 | Vacuolar-sorting receptor 1 | 64133.3 | 580 |
| B4FQT3 | Uncharacterized protein | 65411.3 | 580 |
| B6SSX2 | Gamma-glutamyltranspeptidase 1 | 61254.8 | 580 |
| B6U4Q2 | Eukaryotic translation initiation factor 3 subunit D | 64865.9 | 580 |
| C0HFM6 | Chaperonin CPN60-like 2 mitochondrial | 61264 | 580 |
| C0P732 | Hsp70-Hsp90 organizing protein 3 | 65124.7 | 580 |
| C0PI30 | Putative ribose-5-phosphate isomerase 2 | 63271.4 | 580 |
| A0A1D6FB39 | Glucosidase 2 subunit beta | 65881.9 | 581 |
| A0A1D6GJ75 | Phosphoglucomutase2 | 62952.7 | 581 |
| A0A1D6J8M9 | Eukaryotic translation initiation factor 4B | 63371.7 | 581 |
| A0A1D6NDH6 | Regulator of chromosome condensation (RCC1) family protein | 62859.4 | 581 |
| A0A1D6Q8I9 | Pyrophosphate--fructose 6-phosphate 1-phosphotransferase subunit alpha | 63099.9 | 581 |
| A0A1D6QHI2 | AMP-binding protein | 64181.3 | 581 |
| A0A317YGW6 | Heat shock protein 10, mitochondrial | 62352.3 | 581 |
| B6UAA5 | Protein Kinase-associated protein phosphatase | 63664.1 | 581 |
| C0HGS2 | FHA transcription factor | 63708.1 | 581 |
| C0P9Y0 | Tubulin binding cofactor C domain-containing protein | 63244.1 | 581 |
| C4J4W3 | Hsp70-Hsp90 organizing protein 3 | 65318.1 | 581 |
| A0A1D6F2M3 | Nuclear pore complex protein NUP98A | 62986.6 | 582 |
| A0A1D6FDT2 | Uncharacterized protein | 63331.3 | 582 |
| A0A1D6MCY7 | Eukaryotic translation initiation factor 3 subunit D | 65119.1 | 582 |
| A0A1D6N2Y0 | Mannosyl-oligosaccharide glucosidase GCS1 | 66989.3 | 582 |
| A0A1D6PNQ3 | AAA-type ATPase family protein | 65046.7 | 582 |
| A0A1D6PSZ3 | Bifunctional dihydrofolate reductase-thymidylate synthase | 65790.1 | 582 |
| B6SS27 | AMP-binding protein | 63238.2 | 582 |
| Q03865 | Vicilin-like embryo storage protein | 66161.6 | 582 |
| A0A1D6FKV6 | Ketol-acid reductoisomerase | 62910 | 583 |
| A0A1D6M6Y5 | Putative NOT transcription complex subunit VIP2 | 62263.7 | 583 |
| A0A1D6NL27 | Aldehyde dehydrogenase3 | 62870.4 | 583 |
| A0A317Y7T8 | Aldehyde oxidase GLOX | 62685.3 | 583 |
| B4FAE5 | Uncharacterized protein | 63096.8 | 583 |
| C0PCB7 | Uncharacterized protein | 63523.6 | 583 |
| C4J411 | Imidazole glycerol phosphate synthase hisHF | 62598.5 | 583 |
| C4JC17 | H/ACA ribonucleoprotein complex subunit 4 | 64211.3 | 583 |
| K7VFD7 | Serine/threonine-protein phosphatase 2A 65 kDa regulatory subunit A beta isoform | 65291.2 | 583 |
| Q9SPJ8 | Cell wall invertase (Fragment) | 64717.1 | 583 |
| A0A1D6LUW9 | Putative transcription elongation factor SPT5 homolog 1 | 66366.5 | 584 |
| A0A1D6MH98 | Phosphoribosylaminoimidazole carboxylase family protein / AIR carboxylase family protein | 63444.3 | 584 |
| A0A317YEU7 | Uncharacterized protein | 59704.1 | 584 |
| B6SXW8 | RuBisCO large subunit-binding protein subunit alpha | 61398.5 | 584 |
| B8A0P5 | CTP synthase | 63825.3 | 584 |
| C0P4G8 | Electron transfer flavoprotein-ubiquinone oxidoreductase mitochondrial | 64581 | 584 |
| C0PF76 | Uncharacterized protein | 63668.7 | 584 |
| C4JC43 | Target of Myb protein 1 | 63371.8 | 584 |
| K7VQ06 | Putative DUF1421 domain family protein | 64457.5 | 584 |
| A0A1D6EIS6 | Acyl-CoA synthetase long-chain family member 3 | 64083.1 | 585 |
| A0A1D6FPE5 | Sec23/sec24 transport family protein | 64888.7 | 585 |
| A0A1D6G7R9 | Serine hydroxymethyltransferase 7 | 63653.3 | 585 |
| A0A1D6GBQ3 | Trehalose-6-phosphate synthase15 | 66678 | 585 |
| A0A1D6H1V4 | Protein STRUBBELIG-RECEPTOR FAMILY 1 | 64370.5 | 585 |
| A0A1D6HAD2 | Transmembrane 9 superfamily member | 66227.6 | 585 |
| A0A1D6IUF2 | Laccase | 63624.5 | 585 |
| A0A1D6ML60 | Uncharacterized protein | 66281.9 | 585 |
| A0A1D6QGW9 | Long chain acyl-CoA synthetase 8 | 64271.3 | 585 |
| A0A1D6FGB3 | FACT complex subunit SSRP1 | 65789.4 | 586 |
| A0A1D6H1D4 | FAD/NAD(P)-binding oxidoreductase family protein | 63491 | 586 |
| A0A1D6JY57 | RING-type E3 ubiquitin transferase | 63373.2 | 586 |
| A0A1D6LTC1 | FAD-binding Berberine family protein | 64133.2 | 586 |
| A0A1D6M539 | Sec23/sec24 transport family protein | 64975.7 | 586 |
| A0A1D6NYE7 | Restorer of fertility2 | 63795.6 | 586 |
| B6SY54 | Transmembrane 9 superfamily member | 67012.9 | 586 |
| C0HFL3 | Uncharacterized protein | 65864.8 | 586 |
| A0A1D6F562 | Brassinosteroid LRR receptor kinase | 64786.5 | 587 |
| A0A1D6H286 | 4-coumarate--CoA ligase 1 | 63437.4 | 587 |
| A0A1D6H9Z1 | Beta-fructofuranosidase, cell wall isozyme | 64887.2 | 587 |
| A0A1D6QQW5 | Putative ARF GTPase-activating domain family protein isoform 1 | 64065.3 | 587 |
| A0A1D6QU24 | Anthranilate phosphoribosyltransferase | 63559 | 587 |
| B8A349 | Uncharacterized protein | 65592.5 | 587 |
| H7BRM0 | Pyrophosphate--fructose 6-phosphate 1-phosphotransferase subunit alpha | 63659.8 | 587 |
| A0A1D6EEN1 | Peptidylprolyl isomerase | 66337.2 | 588 |
| A0A1D6FPA5 | Arginyl-tRNA--protein transferase | 65031.9 | 588 |
| A0A1D6GZH4 | Anthranilate phosphoribosyltransferase | 63557.1 | 588 |
| A0A1D6H0A8 | Cycloartenol synthase | 67014.2 | 588 |
| A0A1D6HPY9 | ABC transporter F family member 3 | 65714.4 | 588 |
| A0A1D6JS40 | Peptidyl-prolyl cis-trans isomerase CYP65 | 64854.1 | 588 |
| A0A1D6LHI9 | Beta-hexosaminidase | 64790 | 588 |
| A0A1D6QV35 | Protein transport protein SEC16B-like protein | 63568.8 | 588 |
| A0A317Y9K1 | Outer envelope protein 64, chloroplastic | 63563.1 | 588 |
| C0HGR4 | 4-alpha-glucanotransferase | 65295.9 | 588 |
| C0PHL9 | Exocyst subunit Exo70 family protein | 64839.7 | 588 |
| C4JC33 | DCD (Development and Cell Death) domain protein | 64387.4 | 588 |
| A0A1D6G8I0 | Putative regulator of chromosome condensation (RCC1) family protein | 62050 | 589 |
| A0A1D6JR89 | Conserved oligomeric Golgi complex subunit 8 | 64989.4 | 589 |
| A0A1D6JS44 | Peptidyl-prolyl cis-trans isomerase CYP65 | 64862.9 | 589 |
| A0A1D6MNG6 | Protein EXECUTER 2 chloroplastic | 64092.9 | 589 |
| A0A1X7YIP4 | Pescadillo homolog | 67442.2 | 589 |
| B4FVC1 | RBR-type E3 ubiquitin transferase | 66385.9 | 589 |
| A0A1D6DW81 | Zinc finger (Ubiquitin-hydrolase) domain-containing protein | 65743.9 | 590 |
| A0A1D6EHT1 | Actin-7 | 65891.2 | 590 |
| A0A1D6FHZ5 | Protein ABC transporter 1 mitochondrial | 64319.5 | 590 |
| A0A317Y1E0 | 4-coumarate--CoA ligase-like 1 (Fragment) | 63631.4 | 590 |
| K7TJ36 | Polynucleotide adenylyltransferase family protein | 66941.2 | 590 |
| K7VD58 | Vacuolar protein sorting-associated protein 52 A | 67810.2 | 590 |
| A0A1D6ERL1 | Glucose-6-phosphate isomerase | 64860.6 | 591 |
| A0A1D6IC29 | Malic enzyme | 65462.2 | 591 |
| A0A1D6N8Z5 | Putative ADP-ribosylation factor GTPase-activating protein AGD14 | 65023.8 | 591 |
| A0A1D6NG08 | ATP binding protein | 65124.4 | 591 |
| A0A1D6NRB0 | Dynamin-2A | 63804 | 591 |
| B4FBP3 | Signal recognition particle subunit SRP68 | 67137.8 | 591 |
| B4FFJ0 | Asparagine synthetase1 | 66320.8 | 591 |
| B4FWX5 | Dihydroxy-acid dehydratase chloroplastic | 63267.3 | 591 |
| B6U4F2 | Signal recognition particle subunit SRP68 | 67135.8 | 591 |
| C0P4H6 | ABC transporter F family member 1 | 66335.5 | 591 |
| C0P869 | Dihydroxy-acid dehydratase chloroplastic | 63261.3 | 591 |
| C0PCZ0 | Coronatine-insensitive protein 1 | 66632 | 591 |
| Q9ZTL2 | Cell wall invertase Incw1 | 65192.6 | 591 |
| A0A1D6GP74 | Pre-mRNA-processing protein 40C | 66984 | 592 |
| A0A1D6GZH5 | Anthranilate phosphoribosyltransferase | 64018.7 | 592 |
| A0A1D6L192 | Mitogen-activated protein kinase kinase kinase 1 | 63670.2 | 592 |
| B4FBI4 | Evolutionarily conserved C-terminal region 10 | 64827.2 | 592 |
| B7ZXE2 | Uncharacterized protein | 66837.2 | 592 |
| B7ZZU6 | Uncharacterized protein | 65796.2 | 592 |
| C0PG78 | Monocopper oxidase-like protein SKU5 | 65802.1 | 592 |
| A0A1D6MYM9 | Dihydrolipoamide acetyltransferase component of pyruvate dehydrogenase complex | 63409.7 | 593 |
| A0A1D6N363 | Protein root UVB sensitive 1 chloroplastic | 64488 | 593 |
| B4FQ52 | Protein OBERON 2 | 66373.2 | 593 |
| B6T9C2 | Transmembrane 9 superfamily member | 67496.7 | 593 |
| B6TXL9 | Pyruvate decarboxylase isozyme 1 | 63646.8 | 593 |
| B6TZS3 | Uncharacterized protein | 63702.8 | 593 |
| B8A2V9 | Malic enzyme | 65479.2 | 593 |
| K7UBL0 | Plant UBX domain-containing protein 8 | 65814.2 | 593 |
| A0A1D6IBD1 | ATP binding protein | 65835.6 | 594 |
| A0A1D6LIV5 | Phenylalanine--tRNA ligase beta subunit cytoplasmic | 67043.5 | 594 |
| A0A1D6LYT0 | Uncharacterized protein | 66682.5 | 594 |
| A0A1D6NNM2 | Pre-mRNA-processing protein 40A | 71370.8 | 594 |
| B4FPJ4 | ADP,ATP carrier protein | 64213 | 594 |
| B6U4M6 | Transmembrane 9 superfamily member | 67623.5 | 594 |
| C0P852 | U2 snRNP auxiliary factor large subunit | 65797.2 | 594 |
| C0P9Q6 | Evolutionarily conserved C-terminal region 10 | 65169.3 | 594 |
| C0PDM8 | Protein SUPPRESSOR OF GENE SILENCING 3-like protein | 67752.2 | 594 |
| C0PH33 | Dihydroxyacetone kinase | 61706.4 | 594 |
| A0A1D6DZR9 | AICARFT/IMPCHase bienzyme family protein | 64295.5 | 595 |
| A0A1D6G430 | Phosphatidylserine decarboxylase proenzyme 3 | 65921.2 | 595 |
| A0A1D6HZS3 | Uncharacterized protein | 65399.3 | 595 |
| A0A1D6IGV0 | Exocyst complex component EXO84C | 66740.4 | 595 |
| A0A1D6M791 | Global transcription factor group B1 | 69149.4 | 595 |
| A0A1D6MTX4 | ABC transporter F family member 1 | 66227.3 | 595 |
| A0A1D6ND11 | Haloacid dehalogenase-like hydrolase family protein | 64619.3 | 595 |
| C4J4W6 | Os05g0597150-like protein | 63205.3 | 595 |
| A0A1D6G6Q4 | GPI transamidase component PIG-S-related | 63830.5 | 596 |
| A0A1D6GJ65 | Dynamin-related protein 1C | 66314.3 | 596 |
| A0A1D6HKS2 | Protein EXORDIUM | 61747.1 | 596 |
| A0A1D6HN61 | Leucine aminopeptidase 2 chloroplastic | 61876.9 | 596 |
| A0A1D6IZU5 | Ramosa 1 enhancer locus 2 | 64634.6 | 596 |
| A0A1D6KEA9 | RNA-binding (RRM/RBD/RNP motifs) family protein | 67852 | 596 |
| A0A1D6KGE4 | GTP binding protein | 66265 | 596 |
| A0A1D6KQ33 | Phosphoglucan phosphatase LSF1 chloroplastic | 66119.6 | 596 |
| A0A1D6LKI4 | GPI transamidase component Gpi16 subunit family protein | 67063.2 | 596 |
| A0A1D6P9C6 | Peptidylprolyl isomerase | 66623.2 | 596 |
| A0A317Y170 | Putative nucleoredoxin 1-1 | 66333.7 | 596 |
| A0A317Y1V7 | GTPase LSG1-2 | 66215.9 | 596 |
| B6U832 | GTP binding protein | 66199 | 596 |
| K7VSV1 | Pentatricopeptide repeat-containing protein | 67194.6 | 596 |
| A0A1D6E9Z3 | Transmembrane CLPTM1 family protein | 68306.9 | 597 |
| A0A1D6G6E8 | Dolichyl-diphosphooligosaccharide--protein glycosyltransferase subunit 2 | 64370.7 | 597 |
| A0A1D6IUT1 | ABC transporter F family member 1 | 66375.4 | 597 |
| A0A1D6KC64 | Ubiquitin carboxyl-terminal hydrolase 10 | 67313.3 | 597 |
| A0A1D6KE24 | Heat shock protein 70 | 65066 | 597 |
| B4FEE6 | Uncharacterized protein | 68359.3 | 597 |
| Q9ZTQ4 | Cell wall invertase | 64804 | 597 |
| A0A1D6E202 | Mechanosensitive ion channel protein 1 mitochondrial | 65513.9 | 598 |
| A0A1D6G969 | Protein kinase family protein with ARM repeat domain | 64523.5 | 598 |
| A0A317YIW0 | Trehalase | 66631.9 | 598 |
| C4J625 | Phosphoinositide phosphatase SAC6 | 68142.4 | 598 |
| C4J6N6 | Clathrin interactor EPSIN 1 | 63322.1 | 598 |
| K7U1M0 | Putative L-gulonolactone oxidase 6 | 64766.1 | 598 |
| K7U267 | Glucose-6-phosphate 1-dehydrogenase | 67039.8 | 598 |
| A0A1D6EVW0 | Ribulose bisphosphate carboxylase large chain | 67412.3 | 599 |
| A0A1D6IP11 | BADH-like protein | 66549.5 | 599 |
| A0A1D6IYL7 | AICARFT/IMPCHase bienzyme family protein | 65460.6 | 599 |
| A0A1D6JE12 | Nucleotide binding | 66764.3 | 599 |
| B8A2C4 | Beta-hexosaminidase | 66150.4 | 599 |
| A0A1D6DZ22 | SIT4 phosphatase-associated family protein | 67364.4 | 600 |
| A0A1D6FYP8 | C3HC zinc finger-like | 65179.4 | 600 |
| C0PHP3 | Chaperonin 60 subunit beta 2 chloroplastic | 64029.8 | 600 |
| K7US08 | BSD transcription factor | 67872.5 | 600 |
| A0A1D6IYM1 | AICARFT/IMPCHase bienzyme family protein | 65632.8 | 601 |
| A0A1D6K7E1 | C-terminal binding protein AN | 65886.1 | 601 |
| B4G180 | Uncharacterized protein | 67711.9 | 601 |
| B6U1M3 | Ligatin | 66853 | 601 |
| B8A270 | Protease Do-like 2 chloroplastic | 65669 | 601 |
| K7VJ23 | Vacuolar protein-sorting protein 33 | 67735.8 | 601 |
| A0A1D6FZV2 | Beta-adaptin-like protein | 67076.5 | 602 |
| A0A1D6HJT6 | p-loop containing nucleoside triphosphate hydrolase superfamily protein | 65778.4 | 602 |
| A0A1D6HUM9 | D-2-hydroxyglutarate dehydrogenase mitochondrial | 66238.3 | 602 |
| A0A1D6L144 | Beta-glucosidase | 67142.2 | 602 |
| A0A1D6MJB2 | Leucine-rich repeat extensin-like protein 3 | 64038.4 | 602 |
| B7ZYL8 | Uncharacterized protein | 67844.8 | 602 |
| A0A1D6HN63 | Leucine aminopeptidase 2 chloroplastic | 62728 | 603 |
| A0A1D6NDJ3 | Malic enzyme | 66772 | 603 |
| B7ZXS6 | RING-type E3 ubiquitin transferase | 65158.9 | 603 |
| A0A096RRD4 | ABC transporter E family member 2 | 68262.2 | 604 |
| A0A1D6GSN6 | Heat shock 70 kDa protein 16 | 66307.9 | 604 |
| A0A1D6NJK9 | Phosphoglucomutase chloroplastic | 66075.4 | 604 |
| A0A317YEP0 | Ferredoxin-3, chloroplastic | 66226.2 | 604 |
| B4FMR1 | Arginine--tRNA ligase chloroplastic/mitochondrial | 68015.1 | 604 |
| B4FVK0 | Arginine--tRNA ligase chloroplastic/mitochondrial | 67972.1 | 604 |
| B6SKQ9 | Asparagine synthetase | 67228.6 | 604 |
| B6U7C7 | Brassinosteroid LRR receptor kinase | 66612.6 | 604 |
| C0PD88 | Putative indole-3-acetic acid-amido synthetase GH3.1 | 66982.8 | 604 |
| K7WDQ7 | ABC transporter E family member 2 | 68205.2 | 604 |
| Q45KJ2 | Anthranilate synthase alpha subunit | 66624.2 | 604 |
| A0A096SFU6 | Lysine--tRNA ligase | 68601.8 | 605 |
| A0A1D6E011 | 26S proteasome non-ATPase regulatory subunit 1 homolog B | 66145.4 | 605 |
| A0A1D6HAU6 | Calmodulin binding protein1 | 67064 | 605 |
| B6UC67 | UDP-sugar pyrophospharylase | 66609.1 | 605 |
| B7ZZ67 | Carbon catabolite repressor protein 4 2 | 67027.4 | 605 |
| F5A847 | Starch synthase, chloroplastic/amyloplastic | 65966.1 | 605 |
| K7UGR2 | Putative TCP-1/cpn60 chaperonin family protein isoform 1 | 64364.2 | 605 |
| A0A1D6EW35 | Peptide-N(4)-(N-acetyl-beta-glucosaminyl)asparagine amidase | 68079.3 | 606 |
| A0A1D6F512 | Phosphoinositide phospholipase C | 69033.3 | 606 |
| A0A1D6FM60 | Protein NETWORKED 4A | 68670.1 | 606 |
| A0A1D6IV21 | Melibiase family protein | 68263.4 | 606 |
| A0A1D6L203 | Threonine dehydratase | 65752.7 | 606 |
| A0A1D6MAT1 | Vesicle-fusing ATPase | 66546.4 | 606 |
| B5U8J7 | Asparagine synthetase2 | 67442.7 | 606 |
| F5A846 | Starch synthase, chloroplastic/amyloplastic | 66117.3 | 606 |
| Q8S4W9 | Pyruvate decarboxylase | 65061.6 | 606 |
| A0A1D6JK35 | Signal recognition particle protein subunit 9 | 66507.9 | 607 |
| A0A1D6K6W2 | Trehalase | 67669.1 | 607 |
| A0A1D6KMC9 | Dihydroxy-acid dehydratase chloroplastic | 65263 | 607 |
| A0A1D6PLH2 | Folylpolyglutamate synthase | 67058.3 | 607 |
| A0A1D6PT84 | Protein transport protein Sec24-like CEF | 67189.5 | 607 |
| A0A1D6FHT8 | ATP-citrate synthase beta chain protein 2 | 65975.3 | 608 |
| A0A1D6L143 | Beta-glucosidase | 67750 | 608 |
| A0A1D6LA98 | Anthranilate synthase alpha subunit 2 chloroplastic | 68456.7 | 608 |
| A0A317YD05 | Putative galacturonosyltransferase 11 | 67569 | 608 |
| B6U0W0 | Protein DGS1 mitochondrial | 68769.4 | 608 |
| K7U9C9 | Putative DEAD-box ATP-dependent RNA helicase family protein | 64561.6 | 608 |
| K7UZF0 | Peptide-N4-(N-acetyl-beta-glucosaminyl)asparagine amidase A protein | 66148.9 | 608 |
| A0A1D6EFI8 | Sterol 3-beta-glucosyltransferase UGT80A2 | 66296.9 | 609 |
| A0A1D6GLI2 | Translation initiation factor eIF-2B delta subunit | 66596.6 | 609 |
| A0A1D6NHZ3 | Glucose-1-phosphate adenylyltransferase | 66892.8 | 609 |
| B6U167 | Starch synthase, chloroplastic/amyloplastic | 66858.3 | 609 |
| B6U4J8 | Dynamin-related protein 1A | 68727.2 | 609 |
| B6U6N6 | Translation initiation factor eIF-2B delta subunit | 66645.6 | 609 |
| B6UCY8 | Pre-mRNA-splicing factor prp45 | 67879.6 | 609 |
| B7ZZ13 | Uncharacterized protein | 66688.5 | 609 |
| B8A2L4 | Starch synthase, chloroplastic/amyloplastic | 66810.2 | 609 |
| C0P7E7 | Actin-interacting protein 1-2 | 66206.2 | 609 |
| C4J495 | Uncharacterized protein | 65354.1 | 609 |
| A0A1D6E297 | Protein CTR9-like protein | 69203.4 | 610 |
| A0A1D6GMN6 | Heat shock 70 kDa protein | 65741.8 | 610 |
| A0A1D6IAM2 | Transmembrane 9 superfamily member | 69421.7 | 610 |
| A0A1D6KEB0 | RNA-binding (RRM/RBD/RNP motifs) family protein | 69317.7 | 610 |
| A0A1D6Q3W7 | Protein kinase superfamily protein | 67469.4 | 610 |
| B4FA40 | MACPF domain-containing protein | 68079.3 | 610 |
| B6UDC2 | Actin-interacting protein 1-2 | 66286.2 | 610 |
| A0A096RQJ0 | DEAD-box ATP-dependent RNA helicase 53 | 63910.5 | 611 |
| A0A1D6FFK8 | Uncharacterized protein | 63812.2 | 611 |
| A0A1D6JTC3 | Putative glucan 13-alpha-glucosidase | 68384.7 | 611 |
| A0A1D6N5J5 | Nitroreductase family protein | 67504.8 | 611 |
| A0A1D6NS99 | Acylaminoacyl-peptidase1 | 67749.5 | 611 |
| C0P5E7 | Leukotriene A-4 hydrolase | 67769.2 | 611 |
| C0PHK6 | Dynamin-related protein 1C | 67984.1 | 611 |
| A0A1D6LUR7 | Calcium-binding EF hand family protein | 67517 | 612 |
| A0A1D6MC13 | Dynamin-related protein 1A | 69144.7 | 612 |
| A0A1D6N0E9 | Zn-dependent exopeptidase superfamily protein | 67696.7 | 612 |
| A0A317Y5U3 | Nuclear cap-binding protein subunit 1 | 69879.5 | 612 |
| B4FAB6 | Tetratricopeptide repeat (TPR)-like superfamily protein | 66041.7 | 612 |
| C0HI04 | Uncharacterized protein | 66473.9 | 612 |
| C0PLN9 | D-3-phosphoglycerate dehydrogenase | 63923.1 | 612 |
| A0A1D6FYP9 | C3HC zinc finger-like | 66644 | 613 |
| A0A1D6LPW2 | Twinkle homolog protein chloroplastic/mitochondrial | 69459 | 613 |
| A0A1D6M464 | Uncharacterized protein | 67120.1 | 613 |
| A0A1D6MC91 | Golgin candidate 5 | 70546.8 | 613 |
| A0A1D6ME77 | DNA damage-binding protein 1a | 68824.8 | 613 |
| A0A1D6MR04 | Pentatricopeptide repeat-containing protein mitochondrial | 69039.1 | 613 |
| A0A1D6MZZ0 | SAND family protein | 68650.8 | 613 |
| C0PFJ5 | MACPF domain-containing protein | 68050.5 | 613 |
| C4J3I7 | Thiaminase2 | 67982.8 | 613 |
| A0A1D6F072 | Indole-3-acetic acid amido synthetase | 67208.9 | 614 |
| A0A1D6FI70 | Dihydrolipoyl dehydrogenase | 65950.2 | 614 |
| A0A1D6J9Q7 | Polyadenylate-binding protein | 67419.6 | 614 |
| A0A1D6PR01 | Acyl-coenzyme A oxidase | 67542.7 | 614 |
| A0A1D6Q9E0 | Fimbrin homolog1 | 68757.6 | 614 |
| B6SHH4 | Lysine--tRNA ligase | 69280.5 | 614 |
| C0PG73 | Transducin family protein / WD-40 repeat family protein | 69331.8 | 614 |
| A0A096QP97 | Proton pump-interactor 1 | 68943 | 615 |
| A0A1D6EU89 | Serine/threonine-protein kinase STY46 | 68037 | 615 |
| A0A1D6FCY8 | Protein decapping 5 | 64356 | 615 |
| A0A1D6H2I3 | Protein RRC1 | 69358.1 | 615 |
| A0A1D6LPC7 | External alternative NAD(P)H-ubiquinone oxidoreductase B1 mitochondrial | 68639.1 | 615 |
| A0A1D6LR32 | Putative nucleolar protein 5-1 | 68412.4 | 615 |
| K7W4U7 | RNI-like superfamily protein | 66215.7 | 615 |
| B6SGB6 | Pyrophosphate--fructose 6-phosphate 1-phosphotransferase subunit alpha | 67107.5 | 616 |
| B6SWT4 | Glucose-6-phosphate isomerase | 67508.3 | 616 |
| C0PAU7 | Glucose-6-phosphate isomerase | 67430.5 | 616 |
| C0PGS0 | GPI transamidase component PIG-S-related | 66320.8 | 616 |
| A0A096RIV3 | Proton pump-interactor 1 | 69283.3 | 617 |
| A0A1D6H884 | Polyadenylate-binding protein-interacting protein 3 | 68084 | 617 |
| A0A1D6LTG3 | Haloacid dehalogenase-like hydrolase domain-containing protein 1A isoform 1 | 68354.1 | 617 |
| A0A1D6MZI0 | Uncharacterized protein | 69934 | 617 |
| B6U857 | Purple acid phosphatase | 68810.5 | 617 |
| A0A1D6E6L0 | Polyadenylate-binding protein | 67896.3 | 618 |
| A0A317Y590 | Dynamin-related protein 1E | 69126.1 | 618 |
| B4FWE4 | Phenylalanine ammonia-lyase | 66937 | 618 |
| B6SWB9 | ATP-dependent RNA helicase DDX41 | 68566.7 | 618 |
| B7ZYR6 | Pyrophosphate--fructose 6-phosphate 1-phosphotransferase subunit alpha | 67281.8 | 618 |
| C4IYL3 | Hydroxyproline-rich glycoprotein family protein | 68370.6 | 618 |
| K7U5B0 | Protein CYPRO4 | 67306.5 | 618 |
| A0A1D6I5W1 | ARP protein (REF) | 67332.2 | 619 |
| A0A1D6LR54 | Stromal processing peptidase chloroplastic | 69370.3 | 619 |
| A0A1D6PYJ4 | Thiaminase1 | 68654.4 | 619 |
| A0A317Y9I8 | Translation initiation factor eIF-2B subunit delta | 68283.6 | 619 |
| B7ZXD3 | Glycosyl hydrolase family protein | 67423.3 | 619 |
| B8A0Q6 | Succinate dehydrogenase [ubiquinone] flavoprotein subunit, mitochondrial | 67969.2 | 619 |
| C0HIB9 | Pescadillo homolog | 70892.3 | 619 |
| C0P758 | Malic enzyme | 69133.3 | 619 |
| A0A1D6MUF2 | Heat shock 70 kDa protein 6 chloroplastic | 66285.2 | 620 |
| A0A1D6PAT4 | Putative GTP diphosphokinase RSH1 chloroplastic | 69656.1 | 620 |
| B4FBC9 | Patellin-1 | 68034.6 | 620 |
| B7ZXG5 | Ubiquitin interaction motif-containing protein | 67639 | 620 |
| A0A1D6FP79 | LETM1-like protein | 70678.4 | 621 |
| A0A1D6G8N5 | Heat shock 70 kDa protein | 67981.5 | 621 |
| A0A1D6PSR2 | Protein transport protein SEC23 | 67247.4 | 621 |
| A0A1D6Q1U4 | Transmembrane 9 superfamily member | 70764.4 | 621 |
| B6UH55 | Vacuolar ATP synthase catalytic subunit A | 68418.5 | 621 |
| C0P432 | SEC1 family transport protein SLY1 | 67277.1 | 621 |
| C0PHC0 | Vacuolar proton pump3 | 68446.5 | 621 |
| A0A1D6N836 | ENTH/VHS/GAT family protein | 67075.2 | 622 |
| A0A1D6QAN9 | ATG8-interacting protein 1 | 65627.6 | 622 |
| K7V8U5 | DNA-binding protein | 70089.3 | 622 |
| A0A1Q1A2C4 | Pyrimidine reductase riboflavin1 | 67801.6 | 623 |
| A0A317Y855 | Dolichyl-diphosphooligosaccharide--protein glycosyltransferase subunit 1 | 70066.1 | 623 |
| B6T8I6 | Uncharacterized protein | 67593 | 623 |
| B6U2F8 | Dolichyl-diphosphooligosaccharide--protein glycosyltransferase subunit 1 | 70003.1 | 623 |
| C0P6D0 | FRIGIDA-like protein | 67972.9 | 623 |
| A0A1D6EAS7 | Transcription factor bHLH95 | 63406.6 | 624 |
| A0A1D6EJ13 | Vacuolar-sorting receptor 1 | 69257.1 | 624 |
| A0A1D6K0R9 | Chloroplast protein HCF243 | 68497.4 | 624 |
| A0A1D6LYR2 | Arginine--tRNA ligase chloroplastic/mitochondrial | 69890.1 | 624 |
| A0A1D6MFW1 | Phosphoacetylglucosamine mutase | 67819.9 | 624 |
| B6SH97 | 2-isopropylmalate synthase B | 67178.6 | 624 |
| C0PGB3 | Uncharacterized protein | 66795.3 | 624 |
| A0A1D6ELG8 | Polyadenylate-binding protein | 68000.6 | 625 |
| A0A1D6IU51 | Polynucleotide adenylyltransferase family protein | 71047 | 625 |
| A0A1D6L978 | Methylenetetrahydrofolate reductase | 70153.9 | 625 |
| A0A1D6LHW8 | Putative serine/threonine-protein kinase WNK3 | 69712.9 | 625 |
| A0A1D6DW07 | D-3-phosphoglycerate dehydrogenase | 64795.1 | 626 |
| A0A1D6E7V2 | Inactive LRR receptor-like serine/threonine-protein kinase BIR2 | 67241 | 626 |
| A0A1D6N311 | Dynamin-related protein 3A | 69533.6 | 626 |
| C0P8I4 | 2-isopropylmalate synthase 1 chloroplastic | 67334.8 | 626 |
| C0PG72 | Alkaline/neutral invertase A mitochondrial | 70093.2 | 626 |
| A0A1D6GJK4 | Protein CASP | 72436.8 | 627 |
| A0A1D6HPY8 | ABC transporter F family member 3 | 70550.3 | 627 |
| A0A1D6I0G7 | Polyadenylate-binding protein | 68145.5 | 627 |
| A0A1D6JRB2 | RING/U-box superfamily protein | 68171.7 | 627 |
| A0A1D6M3H9 | Sugary4 | 69424.4 | 627 |
| A0A1D6MRM3 | ATP-citrate synthase beta chain protein 2 | 68240 | 627 |
| A0A1D6ET20 | Uncharacterized protein | 71394.4 | 628 |
| A0A1D6GRP8 | Calmodulin-binding transcription activator 2 | 69580.9 | 628 |
| A0A1D6NMA5 | TPR repeat-containing thioredoxin TTL1 | 66953.8 | 628 |
| B6SSJ8 | SEC1 family transport protein SLY1 | 68339.1 | 628 |
| A0A1D6F7X7 | Sister-chromatid cohesion protein 3 | 71665.8 | 629 |
| B4FAS1 | Glycerol-3-phosphate dehydrogenase | 68153.1 | 629 |
| A0A1D6IU13 | Fatty acid amide hydrolase | 67232.4 | 630 |
| A0A1D6LKL2 | Zinc finger (C3HC4-type RING finger) family protein | 67980.1 | 630 |
| C0PDK8 | Uncharacterized protein | 69889 | 630 |
| K7VIS6 | Protein kinase superfamily protein | 70436.1 | 630 |
| K7W272 | Vicilin-like seed storage protein | 71135.8 | 630 |
| K7W7S8 | RING-type E3 ubiquitin transferase | 68159.3 | 630 |
| C4J6T2 | Exocyst subunit Exo70 family protein | 71142.3 | 631 |
| A0A1D6DQG3 | Acetyl-coenzyme A synthetase | 70168.4 | 632 |
| A0A1D6GN81 | Tubulin-folding cofactor D | 70078.6 | 632 |
| A0A1D6IQY0 | 2-isopropylmalate synthase 1 chloroplastic | 68061.6 | 632 |
| A0A1D6IU03 | Long chain acyl-CoA synthetase 9 chloroplastic | 69800.9 | 632 |
| A0A1D6IVE1 | Prolyl endopeptidase | 70780.3 | 632 |
| K7UEH3 | RNA-binding KH domain-containing protein | 68932.4 | 632 |
| A0A1D6FIN3 | Chromatin-remodeling complex ATPase | 72722.8 | 633 |
| A0A1D6LJU9 | Plant/F18O14-17 protein | 67511.6 | 633 |
| A0A1D6PGW1 | Putative glucan 13-alpha-glucosidase | 71319 | 633 |
| C0HHY2 | Purple acid phosphatase | 70408.5 | 633 |
| A0A1D6LIR4 | Uncharacterized protein | 70115 | 634 |
| A0A1D6P3C3 | Fructose-bisphosphate aldolase 7 cytosolic | 67838.9 | 634 |
| A0A1D6J9J9 | Uncharacterized protein | 68907.7 | 635 |
| A0A1D6M7L9 | Sulfite reductase1 | 69980.3 | 635 |
| A0A1D6E6V0 | Uncharacterized protein | 70280.1 | 637 |
| C0PHP0 | Uncharacterized protein | 67835.6 | 637 |
| K9JA88 | Carotene beta-ring hydroxylase | 69349.8 | 637 |
| A0A1D6E2F8 | Putative methyltransferase PMT15 | 70596.2 | 638 |
| A0A1D6EZK6 | Uncharacterized protein | 71067.9 | 638 |
| A0A1D6N3L6 | DEAD box RNA helicase1 | 69812 | 638 |
| A0A1D6NRE0 | RNI-like superfamily protein | 68461.2 | 638 |
| A0A1Q1A360 | Acetolactate synthase | 68929.7 | 638 |
| B6U2A8 | BAH domain containing protein | 72182.6 | 638 |
| A0A1D6KMU9 | Transmembrane 9 superfamily member | 72490.1 | 639 |
| A0A1D6LTI2 | Haloacid dehalogenase-like hydrolase domain-containing protein 1A isoform 1 | 70742.8 | 639 |
| B4FTE5 | FRIGIDA-like protein | 68384.8 | 639 |
| C4J230 | Uncharacterized protein | 70237.5 | 639 |
| A0A1D6HKJ7 | Exocyst complex component SEC6 | 72955.7 | 640 |
| A0A1D6JYH3 | ABC transporter B family member 6 | 71698.4 | 640 |
| A0A1D6KS20 | Nucleic acid binding protein | 70134.2 | 640 |
| A0A1D6MUJ1 | Exocyst complex component SEC10 | 70094.7 | 640 |
| C0P7J4 | Aminopeptidase P1 | 71419.2 | 640 |
| C4J3S6 | 2-isopropylmalate synthase 1 chloroplastic | 68490 | 640 |
| A0A1D6EZK3 | Uncharacterized protein | 70148 | 641 |
| A0A1D6FZH1 | ATP-dependent zinc metalloprotease FTSH 11 chloroplastic/mitochondrial | 70585.1 | 641 |
| A0A1D6MID0 | Glutamine--tRNA ligase cytoplasmic | 74018.9 | 641 |
| A0A1D6QEH4 | Exocyst complex component SEC6 | 73745.7 | 641 |
| A0A1D6QRE9 | Tubulin binding cofactor C domain-containing protein | 70323.3 | 641 |
| C0P9J3 | SWAP (Suppressor-of-White-APricot)/surp RNA-binding domain-containing protein | 70024.3 | 641 |
| C4J073 | Uncharacterized protein | 68794.9 | 641 |
| K7TVN3 | Putative methyltransferase PMT15 | 70996.5 | 641 |
| A0A1D6NL97 | Leucine-rich repeat extensin-like protein 3 | 69199.7 | 642 |
| B6SVL2 | ADP,ATP carrier protein | 69659.4 | 642 |
| B6SWN1 | 2-isopropylmalate synthase B | 68851.1 | 642 |
| A0A1D6F8K6 | DEAD-box ATP-dependent RNA helicase 52 | 67989.3 | 643 |
| A0A1D6FY81 | SIT4 phosphatase-associated family protein | 71835.7 | 643 |
| C9DQ39 | Cryptochrome 2 | 72809.2 | 643 |
| A0A1D6HGR1 | p-loop containing nucleoside triphosphate hydrolase superfamily protein | 71634.8 | 644 |
| A0A1D6JPX6 | CCR4-NOT transcription complex subunit 6 | 71607.6 | 644 |
| A0A1D6K417 | Peptidylprolyl isomerase | 71975.6 | 644 |
| A0A317Y515 | RNA-binding KH domain-containing protein PEPPER | 69538.5 | 644 |
| B6SVD0 | Phosphatidylserine decarboxylase proenzyme 2 | 71330.3 | 644 |
| C0PAL4 | Alpha-galactosidase | 72563.4 | 644 |
| A0A1D6E1F8 | RBR-type E3 ubiquitin transferase | 72290 | 645 |
| A0A1D6EW29 | Peptide-N(4)-(N-acetyl-beta-glucosaminyl)asparagine amidase | 72163.6 | 645 |
| A0A1D6GMR1 | Trihelix transcription factor GT-2 | 68630.7 | 645 |
| B7ZZX2 | Coatomer subunit gamma | 71587.8 | 645 |
| A0A1D6FGQ2 | TPR repeat-containing thioredoxin TTL1 | 69924.5 | 646 |
| A0A1D6NUF2 | Ribonucleoside-diphosphate reductase | 72609 | 646 |
| C0PD60 | Putative peptidyl-prolyl cis-trans isomerase and WD40 repeat domain family protein | 72507.8 | 646 |
| C0PFQ6 | DNA-binding protein SMUBP-2 | 70891.8 | 646 |
| A0A096RF51 | DEAD-box ATP-dependent RNA helicase 52 | 68369.8 | 647 |
| A0A1D6GEY0 | Mitochondrial Rho GTPase | 71809.5 | 647 |
| A0A1D6N8V1 | Protein MOR1 | 72171.2 | 647 |
| K7V6B0 | Formin-like protein 18 | 71869 | 647 |
| A0A1D6FJS8 | Dynamin protein 1A | 72835.1 | 648 |
| A0A1D6GEZ0 | Mitochondrial Rho GTPase | 71981.7 | 648 |
| A0A1D6JFG4 | Beta-D-xylosidase 4 | 68798.3 | 648 |
| A0A1D6L1Y9 | Phosphoglucomutase1 | 70461.4 | 648 |
| A0A1D6L6W2 | Tetratricopeptide repeat (TPR)-like superfamily protein | 74306.7 | 648 |
| A0A1D6L934 | Mitochondrial Rho GTPase | 72009.6 | 648 |
| A0A1D6N7I4 | Putative mediator of RNA polymerase II transcription subunit 37c | 70843.7 | 648 |
| A0A1D6QEG0 | Aspartic proteinase-like protein 1 | 70725.7 | 648 |
| B8A1R5 | ABC transporter B family member 27 | 69916.7 | 648 |
| C4J410 | Heat shock 70 kDa protein | 70881.8 | 648 |
| A0A097PN36 | Translation initiation factor 2 (Fragment) | 69812.7 | 649 |
| A0A1D6H2B0 | Vacuolar import/degradation Vid27-related protein | 70529.4 | 649 |
| A0A1D6KE29 | Heat shock protein 70 | 71091.9 | 649 |
| B6U1E4 | Heat shock cognate 70 kDa protein 2 | 71165.9 | 649 |
| B7ZZ42 | Heat shock 70 kDa protein 3 | 71162 | 649 |
| C4J1T9 | WEB family protein | 71520.8 | 649 |
| K7VW90 | Putative mediator of RNA polymerase II transcription subunit 37c | 70955.8 | 649 |
| A0A0H4NYD3 | Wall-associated receptor-like kinase | 71646.6 | 650 |
| A0A1D6GJ73 | Phosphoglucomutase2 | 70792.7 | 650 |
| A0A1D6LEZ3 | WPP domain-associated protein | 74294.1 | 650 |
| A0A1D6NH78 | Transducin/WD40 repeat-like superfamily protein | 70920.9 | 650 |
| A0A1D6J424 | Glucose-6-phosphate 1-dehydrogenase | 72812.4 | 651 |
| A0A1D6N2Y4 | Mannosyl-oligosaccharide glucosidase GCS1 | 72901.6 | 651 |
| B4FW80 | Protein arginine N-methyltransferase | 72766 | 651 |
| A0A1D6FA60 | Phosphatidylinositol 345-trisphosphate 3-phosphatase and protein-tyrosine-phosphatase PTEN2A | 70409.8 | 652 |
| A0A1D6FSK8 | Putative histone-lysine N-methyltransferase family protein | 72066.7 | 652 |
| A0A1D6PT07 | DNA replication licensing factor MCM2 | 73403.4 | 652 |
| A0A317Y496 | Uncharacterized protein | 69544.5 | 652 |
| B4F8U2 | Uncharacterized protein | 69135.6 | 652 |
| B6SXK3 | Uncharacterized protein | 72232.5 | 652 |
| K7TJA7 | Putative DUF827 domain containing family protein | 71504.8 | 652 |
| K7VJF3 | Heat shock 70 kDa protein 5 | 71501.5 | 652 |
| O50015 | Malic enzyme | 71869.6 | 652 |
| Q5G1U0 | Malic enzyme | 71636.4 | 652 |
| A0A1D6ED25 | DnaJ domain | 73254.5 | 653 |
| A0A1D6FB29 | Glucosidase 2 subunit beta | 73928.7 | 653 |
| A0A1D6GD84 | Oligomeric Golgi complex subunit 2 | 72368.1 | 653 |
| A0A1D6HHN9 | Phosphoribulokinase / Uridine kinase family | 73826.4 | 653 |
| A0A317Y4E0 | Uncharacterized protein | 69820.3 | 653 |
| B7ZX44 | Hydroxyproline-rich glycoprotein family protein | 70314.7 | 653 |
| C0P4P9 | WPP domain-interacting tail-anchored protein 1 | 72352.6 | 653 |
| A0A1D6FU97 | Arginine--tRNA ligase chloroplastic/mitochondrial | 73185.1 | 654 |
| A0A1D6PYC4 | Nodulin-like protein | 70703.4 | 654 |
| A0A096PQY2 | Long chain acyl-CoA synthetase 5 | 72666 | 655 |
| A0A1D6MUF1 | Heat shock 70 kDa protein 6 chloroplastic | 69768.2 | 655 |
| B6UCN7 | Protein CYPRO4 | 70838.7 | 655 |
| A0A1D6FJS7 | Dynamin protein 1A | 73839.2 | 656 |
| A0A1D6H070 | Leucine-rich repeat/extensin 2 | 67434.7 | 656 |
| A0A1D6HIG4 | Fimbrin-1 | 72957.4 | 656 |
| A0A1D6M236 | Serine/threonine-protein kinase TOR | 73061.4 | 656 |
| C0P4P5 | Alpha-L-arabinofuranosidase 1 | 72199.5 | 656 |
| A0A1D6E7Z9 | Galactose oxidase/kelch repeat superfamily protein | 75457 | 657 |
| A0A1D6FXZ4 | Protein ROOT HAIR DEFECTIVE 3 | 73977.4 | 657 |
| A0A1D6GM13 | Uncharacterized protein | 73706.9 | 657 |
| A0A1D6GTF8 | Protein ENHANCED DISEASE RESISTANCE 2 | 74032.6 | 657 |
| A0A1D6I5H6 | Gamma-tubulin complex component | 72728.3 | 657 |
| A0A1D6KVJ4 | Phospholipid-transporting ATPase | 74399.4 | 657 |
| A0A1D6NK33 | Protein ROOT HAIR DEFECTIVE 3 | 73775.2 | 657 |
| C0P3Y1 | Molybdopterin biosynthesis protein CNX1 | 68825.1 | 657 |
| C0PG00 | Uncharacterized protein | 73346.5 | 657 |
| Q309F9 | Molybdenum cofactor biosynthesis protein | 68867.1 | 657 |
| A0A1D6MK32 | Cellulose synthase | 74310.8 | 658 |
| A0A096SVP6 | Octicosapeptide/Phox/Bem1p family protein | 69623.7 | 659 |
| A0A1D6P7N7 | Putative SAP DNA-binding domain family protein isoform 1 | 72303.4 | 659 |
| B6U8G5 | Acyl-CoA binding protein | 72141.1 | 659 |
| K7U6T4 | Putative alpha-L-arabinofuranosidase family protein | 72398.7 | 659 |
| K7VNE0 | Phosphoenolpyruvate carboxykinase homolog2 | 72569.5 | 659 |
| A0A1D6PVX3 | Putative NAC domain transcription factor superfamily protein | 73575.7 | 660 |
| A0A1D6QAS2 | p-loop NTPase domain-containing protein LPA1 homolog 1 | 72777.9 | 660 |
| A0A1D6H482 | Outer envelope protein 80 chloroplastic | 71376.1 | 661 |
| A0A1D6KU83 | Glutamate-ammonia ligase | 73223.8 | 661 |
| A0A1D6LYU9 | Uncharacterized protein | 74480 | 661 |
| A0A1D6PVX4 | Putative NAC domain transcription factor superfamily protein | 73703.8 | 661 |
| B6SHD7 | SNARE-interacting protein KEULE | 74893.9 | 661 |
| B6ST61 | Transmembrane 9 superfamily member | 73901.9 | 661 |
| A0A1D6H177 | DExH-box ATP-dependent RNA helicase DExH11 | 73723.5 | 662 |
| A0A1D6J4J6 | tRNA-dihydrouridine(47) synthase [NAD(P)(+)] | 73417.7 | 662 |
| K7VWG1 | Exocyst subunit Exo70 family protein | 71927.7 | 662 |
| A0A1D6LVT4 | Starch synthase, chloroplastic/amyloplastic | 73262.5 | 663 |
| A0A1D6P3C4 | Fructose-bisphosphate aldolase 7 cytosolic | 71318.1 | 663 |
| B4FW90 | ER luminal binding protein | 73084.2 | 663 |
| O24581 | Luminal-binding protein 3 | 73156.3 | 663 |
| A0A1D6HP20 | Trimeric LpxA-like enzyme | 73002.3 | 664 |
| A0A1D6IH83 | Retrotransposon-like protein | 75528.1 | 664 |
| A0A1D6MF07 | VHS domain-containing protein | 71908 | 664 |
| B4FW89 | Uncharacterized protein | 70390.8 | 664 |
| C0PGQ9 | Long chain acyl-CoA synthetase 5 | 73414.8 | 664 |
| A0A1D6IVI7 | Uncharacterized protein | 72967.2 | 665 |
| A0A1D6M0Q5 | Phosphoribosylaminoimidazole carboxylase family protein / AIR carboxylase family protein | 72525.9 | 665 |
| B6SW65 | Topoisomerase-like protein | 73601.5 | 665 |
| B7ZY51 | Octicosapeptide/Phox/Bem1p family protein | 70396.5 | 665 |
| B7ZYX0 | Uncharacterized protein | 73701.2 | 665 |
| C0P7J5 | Acyl-coenzyme A oxidase | 74177.2 | 665 |
| A0A1D6J2D5 | Cullin-1 | 77483.5 | 666 |
| A0A317YBS1 | Beta-glucosidase BoGH3B | 72275.2 | 666 |
| C0PBR2 | Signal recognition particle subunit SRP72 | 73641.4 | 666 |
| A0A1D6HEP1 | Hsp70-Hsp90 organizing protein 3 | 75073.5 | 667 |
| A0A1D6IKV5 | TolB protein-related | 72177.8 | 667 |
| A0A1D6LY85 | Pyrophosphate-energized vacuolar membrane proton pump 1 | 69640.4 | 667 |
| A0A1D6PY00 | Pumilio homolog 5 | 73698 | 667 |
| C0PGM1 | Acyl-coenzyme A oxidase | 74263.6 | 667 |
| Q5YLM2 | COBRA-like protein 7 | 71376.7 | 667 |
| A0A317YEF6 | Acyl-CoA-binding domain-containing protein 4 | 73017.1 | 668 |
| B4FBI3 | Putative metal-nicotianamine transporter YSL6 | 72331.8 | 668 |
| A0A1D6G7U7 | Uncharacterized protein | 70899.9 | 669 |
| A0A1D6FB78 | Argonaute4a | 74718.5 | 670 |
| A0A1D6LY84 | Pyrophosphate-energized vacuolar membrane proton pump 1 | 70540.5 | 670 |
| A0A1D6Q881 | RabGAP/TBC domain-containing protein | 76426.3 | 670 |
| A0A1D6MRP6 | FAM91A1-like protein | 73247 | 671 |
| A0A1D6N8Z8 | Putative ADP-ribosylation factor GTPase-activating protein AGD14 | 73766.1 | 671 |
| B4FB26 | Uncharacterized protein | 73527.2 | 671 |
| C0HI10 | Uncharacterized protein | 74226.8 | 671 |
| C0P9I5 | RabGAP/TBC domain-containing protein | 76525.4 | 671 |
| A0A1D6GXV6 | Putative hect E3 ubiquitin ligase | 72641.1 | 672 |
| A0A1D6LCQ5 | Uncharacterized protein | 74135.2 | 672 |
| A0A1D6N230 | Uncharacterized protein | 74615.2 | 672 |
| A0A1D6QJ70 | Uncharacterized protein | 73591.1 | 672 |
| A0A317YIY5 | Uncharacterized protein | 75838 | 672 |
| B6U0P4 | RHM1 | 75891.1 | 672 |
| A0A1D6QKI4 | HSP protein | 77433.3 | 673 |
| B6U1S6 | Cleavage and polyadenylation specificity factor subunit 2 | 75138.7 | 673 |
| B6U4G0 | Uncharacterized protein | 76554.1 | 673 |
| A0A1D6ES21 | Heat shock protein 90-2 | 77391.3 | 674 |
| A0A1D6HQ63 | Uncharacterized protein | 74334.7 | 674 |
| A0A1D6KEA2 | Diacylglycerol kinase | 74024.8 | 674 |
| B6UHC0 | Acyl-peptide hydrolase-like | 74932.7 | 674 |
| A0A1D6H3M3 | Nuclear pore complex protein NUP205 | 76812.1 | 675 |
| A0A1D6HBU4 | Glycerophosphodiester phosphodiesterase GDPDL3 | 73620.1 | 675 |
| A0A1D6HCJ4 | Hydroxymethylbutenyl diphosphate synthase1 | 75046.3 | 675 |
| A0A1D6I6T7 | Heat shock protein 90-2 | 77433.2 | 675 |
| A0A1D6KID1 | Heat shock protein 90-2 | 77383.1 | 675 |
| A0A317YFX6 | Uncharacterized protein | 76124.1 | 675 |
| A0A1D6HSY2 | Ankyrin repeat family protein / regulator of chromosome condensation (RCC1) family protein | 71792.5 | 676 |
| A0A1D6MK62 | Phospholipase D1 | 77287.1 | 677 |
| A0A1D6PX78 | KH domain-containing protein | 72246 | 677 |
| A0A1D6Q4T5 | Uncharacterized protein | 73541.2 | 677 |
| B1P2H3 | Filamentation temperature-sensitive H 2A | 72435.5 | 677 |
| C0P9K2 | Uncharacterized protein | 72547.7 | 677 |
| C0P9N8 | Peptidylprolyl isomerase | 74676.8 | 677 |
| A0A1D6GLH6 | DNA-directed RNA polymerase subunit beta | 76846.6 | 678 |
| B4FW48 | Phospholipid:diacylglycerol acyltransferase 1 | 75153.2 | 678 |
| B6U4A3 | Heat shock 70 kDa protein | 72747.9 | 678 |
| A0A1D6EC22 | Glycine--tRNA ligase mitochondrial 1 | 75956.3 | 679 |
| A0A1D6KI93 | Peroxisome biogenesis protein 5 | 75251 | 679 |
| A0A1X7YII0 | Exocyst subunit Exo70 family protein | 74946.9 | 679 |
| A0A1D6FRZ7 | Ypt/Rab-GAP domain of gyp1p superfamily protein | 76714.8 | 681 |
| A0A1D6GCA5 | Peptidase M28 family protein | 74176.2 | 681 |
| A0A1D6H175 | DExH-box ATP-dependent RNA helicase DExH11 | 77029.4 | 681 |
| A0A1D6KNL5 | Dentin sialophosphoprotein-related | 74686.9 | 681 |
| A0A1D6QLH1 | Long chain acyl-CoA synthetase 9 chloroplastic | 74822 | 681 |
| A0A317YDT7 | Conserved oligomeric Golgi complex subunit 3 | 76288.5 | 681 |
| C4JBB8 | Heat shock 70 kDa protein 9 mitochondrial | 72743.3 | 681 |
| K7VZE6 | Electron transporter | 72949 | 681 |
| A0A1D6JDF0 | Translation elongation factor EF1A/initiation factor IF2gamma family protein | 73937 | 682 |
| A0A1D6N5T8 | Beta-galactosidase | 75711.2 | 682 |
| A0A096R6Z8 | Heat shock 70 kDa protein 6 chloroplastic | 73020.9 | 683 |
| A0A1D6HXF2 | Zn-dependent exopeptidase superfamily protein | 75207.3 | 683 |
| A0A1D6LA76 | Protein DEFECTIVE IN EXINE FORMATION 1 | 76525.5 | 684 |
| A0A1D6MUZ1 | Protein kinase superfamily protein | 79125.4 | 684 |
| A0A317Y709 | DEAD-box ATP-dependent RNA helicase 21 | 78569.4 | 684 |
| B6U7U8 | Acyl-coenzyme A oxidase | 76167.5 | 684 |
| A0A1D6QK44 | Copper-transporting ATPase PAA1 chloroplastic | 71379.6 | 685 |
| A0A1D6QQ59 | KH domain-containing protein | 70037.8 | 685 |
| C0PFY6 | Uncharacterized protein | 70275.2 | 685 |
| A0A1D6FYU4 | Putative leucine-rich repeat receptor-like protein kinase family protein | 70841 | 686 |
| C0P5A5 | Conserved oligomeric Golgi complex subunit 6 | 76505.6 | 686 |
| C0PFM0 | Uncharacterized protein | 76645.4 | 686 |
| C0P3Y3 | Uncharacterized protein | 74326.6 | 687 |
| Q9XFL9 | Lipoxygenase (Fragment) | 77507.9 | 687 |
| A0A1D6ELI8 | RNA binding | 74704.6 | 688 |
| Q6Y2W8 | GCK-like kinase MIK | 75563.1 | 688 |
| A0A1D6GDM6 | DPP6 N-terminal domain-like protein | 73941.3 | 689 |
| A0A1D6HFI8 | Mechanosensitive ion channel protein | 78234.8 | 689 |
| B4F9M6 | KH domain-containing protein | 70155 | 690 |
| B4FJE0 | Uncharacterized protein | 75159.4 | 690 |
| A0A1D6I3Y4 | Trehalose-6-phosphate synthase13 | 76602.2 | 691 |
| B6SWC4 | AMP-binding protein | 75816.3 | 691 |
| B6U114 | GTP-ase activating protein for Arf containing protein | 74824 | 691 |
| A0A1D6J2V0 | Fatty acid amide hydrolase | 75610.9 | 692 |
| A0A1D6KKI9 | Glycine--tRNA ligase mitochondrial 1 | 76722.3 | 692 |
| B4FXY5 | Uncharacterized protein | 74628.2 | 692 |
| C0PGQ1 | Glycine--tRNA ligase mitochondrial 1 | 76765.4 | 692 |
| K7U6W2 | Putative ARF GTPase-activating domain family protein isoform 1 | 74905.2 | 692 |
| A0A1D6HX03 | E3 ubiquitin-protein ligase ORTHRUS 2 | 76106.1 | 694 |
| A0A1D6Q991 | WD40, G-beta domain containing family protein, family | 76185.7 | 694 |
| C0HFL6 | NADPH--cytochrome P450 reductase | 76409.7 | 694 |
| Q6Y2W9 | Atypical receptor-like kinase MARK | 71923.1 | 694 |
| Q84UE5 | Isoamylase-type starch debranching enzyme ISO3 | 78615.6 | 694 |
| A0A1D6JWX5 | ARM repeat superfamily protein | 76773 | 695 |
| A0A1D6NRA2 | Dynamin-2A | 75769.1 | 696 |
| A0A1D6JWX9 | ARM repeat superfamily protein | 76974.2 | 697 |
| A0A317Y1U4 | Cleavage and polyadenylation specificity factor subunit 3-I | 77451 | 697 |
| C0PGI0 | Terpene cyclase/mutase family member | 79322 | 697 |
| A0A1D6DTH3 | Dolichyl-diphosphooligosaccharide--protein glycosyltransferase subunit STT3B | 78187.4 | 698 |
| A0A1D6ES19 | Heat shock protein 90-2 | 80171.4 | 698 |
| A0A1D6H3D0 | Guanylate-binding family protein | 79661.3 | 698 |
| A0A1D6QLG5 | Long chain acyl-CoA synthetase 9 chloroplastic | 76696.1 | 698 |
| B4FW57 | Uncharacterized protein | 78808.7 | 698 |
| C0PFV1 | Plasma membrane ATPase | 77643.6 | 698 |
| A0A1D6HB66 | Cryptochrome-1 | 78976.7 | 699 |
| A0A1D6I6T8 | Heat shock protein 90-2 | 80273.5 | 699 |
| A0A1D6JDV7 | Nuclear pore complex protein NUP88 | 76897.4 | 699 |
| A0A317YI54 | CSC1-like protein HYP1 | 79018.3 | 699 |
| C3UZ63 | HSP protein | 80361.5 | 699 |
| K7URC3 | Putative nucleolin-like family protein | 74477.9 | 700 |
| A0A1D6GRJ8 | RNA-binding (RRM/RBD/RNP motifs) family protein | 76788 | 701 |
| A0A317YHL6 | ABC transporter B family member 25 | 77037.4 | 701 |
| B6SH90 | Histone deacetylase | 76762.4 | 701 |
| A0A1D6LM36 | Putative glycosyl transferase family protein | 81338.5 | 702 |
| B8A0M3 | Uncharacterized protein | 72416.3 | 702 |
| A0A1D6GX90 | Putative integral membrane protein conserved region (DUF2404) | 78061 | 703 |
| A0A1D6HZB6 | ABC transporter B family member 28 | 76133 | 703 |
| B6UFB3 | Stromal 70 kDa heat shock-related protein | 74669.8 | 703 |
| B8A046 | Phenylalanine ammonia-lyase | 75463.4 | 703 |
| C0PDN3 | Uncharacterized protein | 78017.9 | 703 |
| K7UCZ5 | Heat shock 70 kDa protein 6 chloroplastic | 74669.8 | 703 |
| A0A1D6HIG5 | Fimbrin-1 | 78099.3 | 704 |
| A0A1D6KNJ3 | Sec34-like family protein | 78723.4 | 704 |
| B4FYV1 | Gamma-tubulin complex component | 79099.4 | 704 |
| K7V321 | ATP-dependent zinc metalloprotease FTSH 4 mitochondrial | 76043.2 | 704 |
| A0A1D6H9J3 | Acetyl-coenzyme A synthetase | 78020.1 | 706 |
| A0A1D6PZE1 | Putative fucosyltransferase-like protein | 78248 | 706 |
| A0A1D6F2G9 | Tetratricopeptide repeat (TPR)-like superfamily protein | 81703.7 | 707 |
| A0A1D6ELI7 | RNA binding | 76567.6 | 708 |
| A0A1D6GFA7 | Exocyst complex component EXO84B | 77325.6 | 708 |
| A0A1D6KTC2 | Exportin-4 | 78825.8 | 708 |
| A0A1D6NBY9 | Nuclear pore complex protein Nup85 | 79157.5 | 708 |
| A0A1D6F948 | ABC transporter F family member 4 | 77671.1 | 709 |
| A0A1D6L8J5 | Cellulose synthase | 79566.6 | 709 |
| A0A1D6HTM3 | ABC transporter F family member 4 | 77726.2 | 711 |
| A0A1D6JTQ0 | Protein VAC14-like protein | 80275.1 | 711 |
| A0A1D6K6C1 | Xanthine dehydrogenase 1 | 78411.1 | 711 |
| A0A317YG83 | Catalase | 81318.2 | 711 |
| A0A1D6EJ54 | Ubiquitin-activating enzyme E1 2 | 79341.2 | 712 |
| A0A1D6LPJ5 | Heat shock 70 kDa protein 16 | 78801 | 712 |
| A0A1D6NWK3 | Zn-dependent exopeptidase superfamily protein | 77859.5 | 713 |
| A0A1D6L6V3 | Cullin-4 | 82440.6 | 714 |
| C0P4Q3 | Heat shock protein 90 kDa | 81801.8 | 714 |
| A0A1D6FA33 | Suppressor of RPS4-RLD 1 | 80215.4 | 715 |
| A0A1D6KA81 | CSC1-like protein | 83114.4 | 715 |
| A0A317YBF4 | Glutamate--tRNA ligase, cytoplasmic | 80893.1 | 715 |
| B6SRL9 | Phenylalanine ammonia-lyase | 76688.6 | 715 |
| A0A1D6FB81 | Argonaute4a | 80105.6 | 716 |
| A0A1D6FNY1 | Cytosolic endo-beta-N-acetylglucosaminidase 1 | 80054.5 | 716 |
| A0A1D6QT69 | Terpene cyclase/mutase family member | 80762.5 | 716 |
| C0PI10 | Uncharacterized protein | 77423.7 | 716 |
| C4IYE1 | Delta-1-pyrroline-5-carboxylate synthase | 77707.3 | 717 |
| A0A1D6M201 | Uncharacterized protein | 79104.2 | 718 |
| B6U0Z0 | Phenylalanine ammonia-lyase | 77423.7 | 718 |
| A0A1D6K4K9 | SAP domain-containing protein | 77981.3 | 719 |
| B6SRN0 | Transposon protein | 80211 | 719 |
| A0A1D6LEN8 | MA3 domain-containing protein | 78928.1 | 720 |
| A0A1D6PHQ2 | Tetratricopeptide repeat (TPR)-like superfamily protein | 80687.3 | 720 |
| C0P790 | Eukaryotic translation initiation factor 3 subunit B | 82999 | 720 |
| A0A1D6PUT4 | 5'-3' exoribonuclease | 83517.4 | 721 |
| B6UCK4 | Catalytic/ ligase | 78443.1 | 722 |
| C0PL35 | Enoyl-CoA hydratase1 | 79030.5 | 723 |
| K7VFE6 | Uncharacterized protein | 82266.6 | 723 |
| A0A1D6HKJ4 | Exocyst complex component SEC6 | 81772.8 | 724 |
| A0A1D6KKV4 | Uncharacterized protein | 79990 | 725 |
| A0A1D6KSP7 | Threonine--tRNA ligase mitochondrial 1 | 83003.3 | 725 |
| A0A1D6QC13 | DNA helicase | 79755.2 | 725 |
| A0A1D6G4U3 | Potassium transporter 3 | 81274.4 | 726 |
| A0A1D6JY56 | RING-type E3 ubiquitin transferase | 78487.9 | 726 |
| A0A1D6PGX0 | Methionine--tRNA ligase cytoplasmic | 81011 | 726 |
| A0A2Z3N082 | Long chain acyl-CoA synthetase | 80130.5 | 726 |
| B6U6S2 | Acyl-CoA synthetase long-chain family member 3 | 79895.3 | 726 |
| C0P6Q6 | DNA gyrase subunit B | 80215.8 | 726 |
| A0A1D6FY51 | SIT4 phosphatase-associated family protein | 80983.5 | 727 |
| A0A1D6H4X4 | Uncharacterized protein | 78611.4 | 727 |
| A0A1D6LMX0 | Endoplasmin-like protein | 83630.4 | 727 |
| A0A1D6N0A8 | Cellulose synthase-like protein G3 | 78242.1 | 727 |
| A0A1D6QNT3 | Uncharacterized protein | 78475.2 | 727 |
| A0A1D6PK68 | Uncharacterized protein | 79994.6 | 729 |
| A0A317YCJ3 | Putative acyl-activating enzyme 18, peroxisomal | 79062.6 | 729 |
| B8A348 | DNA helicase | 80232.7 | 729 |
| K7UGU4 | Putative WD40-like beta propeller repeat family protein | 80877.4 | 730 |
| A0A1D6F5U8 | Alpha-mannosidase | 81874 | 731 |
| A0A1D6GWU3 | Calcium-binding EF hand family protein | 79405.6 | 731 |
| A0A1D6JHN7 | Trypsin family protein | 77978.4 | 731 |
| B6U134 | Prolyl endopeptidase | 81873.7 | 731 |
| A0A1D6IJH7 | Inter-alpha-trypsin inhibitor heavy chain-related | 80595.5 | 732 |
| A0A1D6LJH7 | Protein transport protein SEC16B-like protein | 78717.7 | 732 |
| A0A1D6GAB2 | Vps51/Vps67 family (Components of vesicular transport) protein | 80570.9 | 733 |
| A0A1D6I5L0 | Formate tetrahyrofolate ligase | 78483.7 | 733 |
| A0A1D6M6I4 | DNA helicase | 80841.3 | 733 |
| B7ZZV8 | Uncharacterized protein | 79732.9 | 733 |
| A0A1D6PJZ1 | Cell division control protein 48 homolog D | 81807.1 | 734 |
| A0A1D6QEK1 | p-loop containing nucleoside triphosphate hydrolase superfamily protein | 82460.5 | 734 |
| A0A1D6EJ50 | Ubiquitin-activating enzyme E1 2 | 81281.6 | 735 |
| A0A1D6HKC3 | Cullin-3B | 85582.7 | 736 |
| A0A1D6HNY0 | Endoribonuclease | 79618.2 | 736 |
| A0A1D6NVZ7 | Transketolase 1 | 79093.7 | 736 |
| A0A1D6HP15 | Trimeric LpxA-like enzyme | 81687.8 | 737 |
| A0A1D6JNX2 | Cell division control protein 48 homolog D | 81995.2 | 737 |
| A0A1D6KSQ7 | Threonine--tRNA ligase mitochondrial 1 | 84467 | 737 |
| A0A1D6PW49 | DNA topoisomerase 1 beta | 83208.9 | 737 |
| C0PFX5 | TIM-barrel signal transduction protein isoform 2 | 78762.8 | 737 |
| A0A1D6MXW5 | Heat shock protein 90-6 mitochondrial | 83128.3 | 738 |
| B6SP44 | Glutamate carboxypeptidase 2 | 80628.6 | 738 |
| A0A1D6G0L5 | Transducin/WD40 repeat-like superfamily protein | 80370.2 | 739 |
| A0A1D6GKX8 | Threonine--tRNA ligase mitochondrial 1 | 84419.9 | 739 |
| A0A1D6MCT3 | Guanylate-binding family protein | 83869.1 | 739 |
| A0A1D6PVQ2 | Cullin-3B | 85735.6 | 739 |
| A0A1D6PW46 | DNA topoisomerase 1 beta | 82177.7 | 740 |
| A0A1D6KFW8 | Methylcrotonoyl-CoA carboxylase subunit alpha | 80446.7 | 741 |
| A0A1D6LE60 | HXXXD-type acyl-transferase family protein | 79781.7 | 741 |
| C0P406 | Vesicle-fusing ATPase | 81317.3 | 741 |
| A0A1D6HNX9 | Endoribonuclease | 80709.3 | 742 |
| A0A1D6HW11 | Golgin subfamily A member 4 | 82084.7 | 742 |
| B7ZXU2 | Serrate RNA effector molecule | 82181.9 | 742 |
| A0A1D6GRR2 | Leucine aminopeptidase 2 chloroplastic | 81896.4 | 743 |
| A0A1D6IDF9 | ABC transporter G family member 34 | 84512.9 | 743 |
| B7ZXG1 | Uncharacterized protein | 80738.9 | 743 |
| A0A1D6L210 | NADH dehydrogenase [ubiquinone] iron-sulfur protein 1 mitochondrial | 80717 | 744 |
| A0A317Y5Q7 | NADH dehydrogenase [ubiquinone] iron-sulfur protein 1, mitochondrial | 80623.8 | 744 |
| K7U2E4 | Amine oxidase | 82989.6 | 744 |
| A0A1D6F7B1 | Centromere/kinetochore protein zw10-like protein | 82865.2 | 746 |
| A0A1D6L0C8 | Zinc finger CCCH domain-containing protein 24 | 79770.9 | 746 |
| A0A1D6GK17 | Zinc finger CCCH domain-containing protein 30 | 79792.8 | 747 |
| A0A1D6LIR7 | Uncharacterized protein | 82951 | 748 |
| A0A1D6G8S2 | V-type proton ATPase subunit a | 85474 | 749 |
| A0A1D6J612 | Double Clp-N motif-containing P-loop nucleoside triphosphate hydrolase superfamily protein | 83305.5 | 749 |
| B6SWP8 | Carbohydrate binding protein | 77271.2 | 749 |
| A0A1D6HBU2 | Glycerophosphodiester phosphodiesterase GDPDL3 | 81245.8 | 750 |
| A0A1D6GWP6 | 14-alpha-glucan-branching enzyme 2-2 chloroplastic/amyloplastic | 85363.4 | 751 |
| A0A1D6MXN6 | Golgin candidate 6 | 83286.4 | 751 |
| A0A1D6DVL4 | Plasma membrane ATPase | 81922.4 | 752 |
| A0A1D6G5A0 | GYF domain-containing protein | 82631.3 | 752 |
| A0A1D6JT90 | Methionine--tRNA ligase cytoplasmic | 83338.9 | 752 |
| A0A1D6QH74 | ARM repeat superfamily protein | 82913.6 | 752 |
| A0A317YEU3 | Vacuolar protein sorting-associated protein 35 | 84898.8 | 753 |
| B8A2F4 | Uncharacterized protein | 83691.8 | 753 |
| C0PAH4 | Transducin family protein / WD-40 repeat family protein | 82410.5 | 755 |
| A0A1D6E768 | LETM1-like protein | 85809.1 | 756 |
| B6SXZ1 | Uncharacterized protein | 84321 | 756 |
| A0A1D6K4K6 | Apoptotic chromatin condensation inducer in the nucleus | 82445.8 | 757 |
| A0A1D6PAF1 | Putative jumonji-like transcription factor family protein | 84395.4 | 757 |
| Q6QP44 | Putative aminoalcoholphosphotransferase | 84454.4 | 757 |
| A0A1D6GNU9 | Flowering locus K homology domain | 83352.3 | 759 |
| A0A1D6ERH9 | Protein transport protein SEC23 | 84434 | 760 |
| A0A317Y9V5 | Uncharacterized protein | 83657.8 | 760 |
| A0A317YGK3 | Conserved oligomeric Golgi complex subunit 4 | 84129.1 | 760 |
| B6U6J4 | ATP binding protein | 83751.4 | 760 |
| A0A1D6G977 | Protein kinase family protein with ARM repeat domain | 82695.1 | 761 |
| B4FW76 | Division protein | 83468.8 | 761 |
| A0A1D6ERI0 | Protein transport protein SEC23 | 84662.2 | 762 |
| A0A1D6L6V7 | Cullin-4 | 86095.3 | 762 |
| K7VRI2 | Vacuolar proton pump homolog1 | 79579.7 | 762 |
| A0A1D6FF44 | Ubiquitinyl hydrolase 1 | 84979 | 763 |
| B6U0R8 | Subtilisin-like protease | 78277.2 | 764 |
| A0A1D6LHW5 | Putative serine/threonine-protein kinase WNK3 | 85433.8 | 765 |
| A0A1D6LJ42 | Protein MEI2-like 5 | 84889.9 | 765 |
| B6UEE8 | Pyrophosphate-energized vacuolar membrane proton pump | 78946.2 | 765 |
| B6UF55 | 5-methyltetrahydropteroyltriglutamate--homocysteine methyltransferase | 84469.5 | 765 |
| B8A1R8 | 5-methyltetrahydropteroyltriglutamate--homocysteine methyltransferase 1 | 84617.8 | 765 |
| A0A1D6E4T9 | Putative beta-D-xylosidase 6 | 82589.9 | 766 |
| A0A1D6J073 | Protein PAF1-like protein | 86522.5 | 766 |
| A0A1D6KPN2 | Aminopeptidase | 85676.6 | 766 |
| A0A1D6NN32 | Cleavage and polyadenylation specificity factor subunit 2 | 85464.5 | 766 |
| A0A317Y087 | Uncharacterized protein | 84505.7 | 766 |
| C0P5Y3 | 5-methyltetrahydropteroyltriglutamate--homocysteine methyltransferase 1 | 84491.6 | 766 |
| C4J0U7 | LETM1-like protein | 87227.5 | 766 |
| Q5K3Q7 | Vacuolar H+-translocating inorganic pyrophosphatase | 80115.4 | 766 |
| Q8W529 | Methionine synthase (Fragment) | 84452.7 | 766 |
| A0A1D6LZ96 | Kinesin-like protein | 85671.9 | 767 |
| A0A1D6KTS3 | Nucleolin 2 | 81280.7 | 768 |
| K7UKK5 | Elongation factor G, chloroplastic | 84299.8 | 768 |
| A0A1D6MEK5 | Argonaute104 | 85870.5 | 769 |
| A0A1D6EHI0 | Protein TPLATE | 86336.5 | 770 |
| A0A1D6NNR1 | Pre-mRNA-processing protein 40A | 89507.2 | 771 |
| K7UB79 | Myosin heavy chain-related protein | 87628.3 | 771 |
| A0A1D6E491 | RNA recognition water-stress protein1 | 86487.6 | 772 |
| A0A096RKQ9 | Phosphoribosylanthranilate transferase isoform 1 | 89459.1 | 774 |
| A0A1D6KV27 | Acylamino-acid-releasing enzyme | 84926.6 | 774 |
| A0A1D6NNI0 | Pre-mRNA-processing protein 40A | 90070.8 | 774 |
| B6U4F7 | Acylamino-acid-releasing enzyme | 84923.7 | 774 |
| A0A1D6JV44 | Coatomer subunit gamma | 86104.5 | 775 |
| K7VBA9 | Calcium-dependent lipid-binding (CaLB domain) plant phosphoribosyltransferase family protein | 89153.6 | 775 |
| A0A1D6HC08 | Protein NBR1-like protein | 85954.6 | 776 |
| A0A317YDC6 | Phospholipase D beta 1 | 85377.1 | 776 |
| A0A1D6EVK6 | Protein GRIP | 88494.7 | 777 |
| A0A1D6HD58 | Polyribonucleotide nucleotidyltransferase 2 mitochondrial | 85686.4 | 777 |
| A0A1D6KT35 | Transportin MOS14 | 87153.9 | 777 |
| A0A1D6I6A0 | Isoamylase-type starch debranching enzyme3 | 87132.6 | 778 |
| A0A1D6M6I3 | DNA helicase | 86270.5 | 778 |
| C0HF77 | Subtilisin2 | 82633.4 | 778 |
| A0A1D6N9X4 | Insulin-degrading enzyme-like 1 peroxisomal | 90254.1 | 779 |
| C4J473 | Uncharacterized protein | 87359.6 | 779 |
| A0A096SGQ3 | Amine oxidase | 86434 | 780 |
| A0A1D6IXT7 | Bifunctional dethiobiotin synthetase/78-diamino-pelargonic acid aminotransferase mitochondrial | 85706.8 | 780 |
| B4F8R5 | Putative beta-D-xylosidase 7 | 83942.3 | 780 |
| A0A096PY84 | Octicosapeptide/Phox/Bem1p (PB1) domain-containing protein / tetratricopeptide repeat (TPR)-containing protein | 85892.4 | 781 |
| A0A1D6KJ43 | UvrB/uvrC motif family protein | 85485.7 | 781 |
| A0A1D6JW70 | V-type proton ATPase subunit a | 88764.9 | 782 |
| A0A1D6MK68 | Phospholipase D | 89283.3 | 783 |
| A0A1D6N215 | Potassium transporter | 87512.8 | 783 |
| C0PGM7 | Uncharacterized protein | 88767.1 | 783 |
| Q1A5Y4 | Phytochrome B1 (Fragment) | 85357 | 783 |
| A0A1D6FY00 | Protein ROOT HAIR DEFECTIVE 3 homolog | 87997.4 | 784 |
| A0A1D6G5W6 | Dynamin-related protein 3A | 86083 | 784 |
| A0A1D6J619 | Double Clp-N motif-containing P-loop nucleoside triphosphate hydrolase superfamily protein | 87139.8 | 784 |
| A0A1D6NK31 | Protein ROOT HAIR DEFECTIVE 3 homolog | 87767 | 784 |
| A0A317Y904 | Uncharacterized protein | 88297.7 | 784 |
| Q5GAP5 | Putative paramyosin | 86904.2 | 784 |
| A0A096T3A1 | Neutral/alkaline non-lysosomal ceramidase | 86318.8 | 785 |
| A0A1D6H4I9 | Putative pre-mRNA-splicing factor ATP-dependent RNA helicase DEAH5 | 87810.6 | 785 |
| K7TQJ7 | Eukaryotic translation initiation factor isoform 4G-2 | 86701.2 | 785 |
| A0A1D6E703 | Eukaryotic translation initiation factor isoform 4G-2 | 86463 | 786 |
| A0A1D6FNA7 | Heat shock 70 kDa protein 14 | 86994 | 786 |
| A0A1D6G4H3 | Villin-2 | 86963.8 | 786 |
| A0A1D6K4W3 | F-box protein | 89654.6 | 786 |
| A0A1D6PNM2 | AAA-type ATPase family protein | 84569.7 | 786 |
| A0A1D6GJ34 | Receptor-like kinase TMK2 | 83284.2 | 787 |
| A0A1D6HKI2 | RINT1-like protein MAG2 | 88321.7 | 787 |
| A0A1D6QK75 | Heat shock protein 90-5 chloroplastic | 88668.5 | 787 |
| A0A1D6QCI3 | Trehalose-6-phosphate synthase10 | 87072.6 | 788 |
| C0PEI1 | Exocyst complex component | 88222.2 | 788 |
| A0A1D6M432 | Pyruvate orthophosphate dikinase1 | 86199.1 | 789 |
| O22637 | SU1 isoamylase | 88352.1 | 789 |
| A0A1D6JRX9 | Calcium-transporting ATPase | 85019.5 | 790 |
| K7UQK1 | Protein transport protein SEC23 | 86339 | 791 |
| A0A1X8DP21 | Uncharacterized protein | 87127.2 | 792 |
| A0A1D6NXQ2 | Protein MEI2-like 5 | 87273.2 | 793 |
| A0A1D6P848 | Sucrose synthase | 90537.8 | 793 |
| A0A1D6I6D9 | Uncharacterized protein | 88697.5 | 794 |
| A0A1D6FTG1 | Trehalose-6-phosphate synthase1 | 88949.1 | 795 |
| A0A1D6PJK9 | Aconitate hydratase | 86398.7 | 795 |
| A0A1D6H4R4 | Prolyl oligopeptidase family protein | 88921.2 | 796 |
| A0A1D6MID5 | Glutamine--tRNA ligase cytoplasmic | 90294.3 | 796 |
| B6UC34 | Glutaminyl-tRNA synthetase | 90069.2 | 796 |
| Q93WS3 | Sucrose synthase (Fragment) | 90452.6 | 796 |
| A0A1D6HDS7 | Regulator of nonsense transcripts UPF2 | 90570.1 | 797 |
| A0A1D6I6H5 | Heat shock protein 90-5 chloroplastic | 90201 | 797 |
| A0A317Y361 | Glycerophosphodiester phosphodiesterase GDPDL4 | 85339.3 | 797 |
| A0A1D6LBS5 | Putative acyl-activating enzyme 16 chloroplastic | 88561.9 | 798 |
| A4GUI1 | Starch branching enzyme IIb | 90556.2 | 799 |
| Q08047 | 1,4-alpha-glucan-branching enzyme 2, chloroplastic/amyloplastic | 90517.2 | 799 |
| Q84UE6 | Isoamylase-type starch debranching enzyme ISO2 | 86920 | 799 |
| A0A1D6H2I0 | Protein RRC1 | 89515.8 | 800 |
| A0A1D6I2L6 | Retinol dehydrogenase 13 | 89755.8 | 800 |
| A0A1D6IQN9 | Ubiquitin-activating enzyme E1 2 | 89436.1 | 800 |
| A0A317YIN0 | WD repeat-containing protein 75 | 87090.1 | 800 |
| A0A1D6EBS5 | Starch branching enzyme IIa | 90363.6 | 801 |
| A0A1D6FAZ0 | Coatomer subunit beta | 88712.1 | 801 |
| A0A1D6FQV5 | Uncharacterized protein | 88868.8 | 801 |
| A0A1D6GG25 | Cullin-4 | 90505.6 | 801 |
| A0A1D6KPU8 | Putative alpha,alpha-trehalose-phosphate synthase [UDP-forming] 9 | 90691.7 | 801 |
| A0A1D6LNB9 | Leucine-rich repeat (LRR) family protein | 80044.2 | 801 |
| A0A1D6QL53 | Protein ROOT HAIR DEFECTIVE 3 homolog | 89840.4 | 801 |
| A0A1D6QRE5 | Uncharacterized protein | 88051.7 | 801 |
| A0A1D6GWP4 | 14-alpha-glucan-branching enzyme 2-2 chloroplastic/amyloplastic | 90570.3 | 802 |
| A0A1D6HST1 | Ubiquitin carboxyl-terminal hydrolase 13 | 94924 | 802 |
| A0A1D6NMP6 | DEAD-box ATP-dependent RNA helicase 14 | 88951.9 | 802 |
| K7VDR8 | Sucrose synthase | 91730.2 | 802 |
| A0A1D6EWP2 | Pre-mRNA-splicing factor ATP-dependent RNA helicase DEAH7 | 90697.9 | 803 |
| A0A1D6NUH1 | AP-1 complex subunit gamma | 87993.2 | 803 |
| B6U2K5 | Vacuolar protein sorting-associated protein 35 | 90711.3 | 803 |
| A0A1D6EGT0 | Protein transport protein Sec24-like CEF | 89143.3 | 804 |
| A0A1D6K0Q7 | Uncharacterized protein | 90607.5 | 804 |
| A0A1D6KMT0 | Phosphatidylinositol-3-phosphatase myotubularin-1 | 89598 | 804 |
| A0A1D6L516 | Protein kinase superfamily protein | 87619.6 | 804 |
| A0A1D6LMW9 | Endoplasmin-like protein | 92571.3 | 804 |
| K7VQA7 | Cell division control protein 48 homolog D | 89480.9 | 804 |
| A0A1D6NA64 | Vps51/Vps67 family (Components of vesicular transport) protein | 88454.9 | 805 |
| K7USZ5 | Putative splicing factor 3A subunit 1 | 88143.5 | 806 |
| A0A1D6JNX1 | Cell division control protein 48 homolog D | 89683.1 | 807 |
| A0A1D6MW00 | H+-translocating pyrophosphatase | 85647 | 807 |
| A0A1D6GWE1 | Shepherd-like1 | 92873.6 | 808 |
| A0A1D6FHZ0 | Protein transport protein SEC23 | 86616.9 | 809 |
| A0A1D6HI27 | Protease Do-like 7 | 89492.2 | 809 |
| A0A1D6M5K5 | Phosphoribosylanthranilate transferase | 91096.1 | 809 |
| B6UEE3 | Phosphoribosylanthranilate transferase | 91140.2 | 809 |
| A0A1D6M436 | Pyruvate orthophosphate dikinase1 | 88315.6 | 810 |
| A0A1D6MU43 | RING-type E3 ubiquitin transferase | 89104.5 | 810 |
| A0A1D6NUG0 | AP-1 complex subunit gamma | 88651.6 | 810 |
| A0A1D6PH61 | Retrovirus-related Pol polyprotein LINE-1 | 92987.8 | 810 |
| A0A1D6QNE9 | Calcium-dependent lipid-binding family protein | 91255.6 | 810 |
| K7VC94 | Exocyst complex component | 88730.2 | 810 |
| A0A1D6G4G1 | Villin-2 | 89958.4 | 811 |
| A0A1D6GQ42 | Cell division cycle protein 48 | 90117.4 | 811 |
| A0A1D6JMI1 | Exosome complex exonuclease RRP44 homolog A | 90689.2 | 811 |
| B6SV61 | Brassinosteroid LRR receptor kinase | 84491.5 | 811 |
| K7VQG5 | Phospholipase D | 92284.7 | 812 |
| A0A1D6FWJ8 | Methionine S-methyltransferase | 89432.2 | 813 |
| A0A1D6H7D6 | Coatomer subunit beta' | 92188 | 813 |
| A0A1D6HSH5 | IAP-like protein 1 | 86445.2 | 813 |
| A0A1D6IP64 | AMP deaminase | 93596.2 | 813 |
| A0A1D6M2Q4 | Tetratricopeptide repeat (TPR)-like superfamily protein | 91752.5 | 813 |
| A0A317YIH1 | E3 ubiquitin-protein ligase hel2 | 89270.5 | 813 |
| C0PDG3 | Heat shock protein 90-6 mitochondrial | 91178.1 | 813 |
| A0A1D6IP65 | AMP deaminase | 93699.3 | 814 |
| A0A1D6JX05 | ARM repeat superfamily protein | 89154 | 814 |
| O24421 | Starch branching enzyme IIa (Fragment) | 91865.3 | 814 |
| A0A317Y0C7 | Alpha,alpha-trehalose-phosphate synthase [UDP-forming] 6 | 92780.5 | 815 |
| K7TLJ6 | Valine--tRNA ligase mitochondrial 1 | 92714 | 815 |
| K7VGG8 | ATP-dependent zinc metalloprotease FTSH 10 mitochondrial | 89105.8 | 815 |
| A0A1D6E458 | ARM repeat superfamily protein | 89156 | 816 |
| A0A1D6HIA5 | Clustered mitochondria protein | 88851.8 | 816 |
| A0A1D6INL9 | Exocyst complex component SEC8 | 90152.5 | 816 |
| B8A045 | Phospholipase D | 91124.9 | 816 |
| A0A1D6J4E3 | Vacuolar protein sorting-associated protein 54 chloroplastic | 88134.1 | 817 |
| A0A1D6JNG6 | Protein PIR | 93632.7 | 817 |
| A0A097PT11 | Embryo yellow protein (Fragment) | 90182.5 | 818 |
| A0A1D6LHE5 | E3 UFM1-protein ligase 1-like protein | 89990.4 | 818 |
| C0PCW9 | Uncharacterized protein | 89906.4 | 818 |
| A0A1D6F8F3 | DEAD-box ATP-dependent RNA helicase 40 | 89450.7 | 819 |
| A0A1D6PER2 | V-type proton ATPase subunit a | 92640.3 | 820 |
| K7WBS3 | Oligomeric Golgi complex component-related protein | 90410.7 | 820 |
| A0A1D6QT45 | 26S proteasome non-ATPase regulatory subunit 2 homolog | 89699.5 | 821 |
| K7VM86 | Dynamin-related protein 3A | 90179.4 | 821 |
| A0A1D6NT56 | Sucrose synthase | 94072 | 822 |
| C0P3T5 | Beta-galactosidase | 91140.8 | 822 |
| B8A0C1 | Protein kinase family protein with ARM repeat domain | 89433.5 | 823 |
| A0A1D6I9T1 | Pantothenate kinase 2 | 90459.1 | 824 |
| A0A1D6P5E8 | Villin-4 | 91911.3 | 824 |
| A0A1D6JXW3 | Protein TOC75-3 chloroplastic | 89152 | 825 |
| A0A1D6FFH0 | Eukaryotic translation initiation factor 3 subunit A | 96537.7 | 826 |
| A0A1D6KZN6 | Inositol hexakisphosphate and diphosphoinositol-pentakisphosphate kinase | 93273.4 | 826 |
| A0A1D6PDS3 | Protein TOC75-3 chloroplastic | 89242.2 | 826 |
| A0A1D6QT53 | L-arabinokinase | 90078.4 | 826 |
| A0A1D6ENN4 | Bifunctional aspartokinase/homoserine dehydrogenase 2, chloroplastic Aspartokinase Homoserine dehydrogenase | 89811.8 | 828 |
| A0A1D6K308 | Nuclear pore protein | 91691.7 | 828 |
[truncated: 146,741 more chars]
